# Base-mediated homologative rearrangement of nitrogen–oxygen bonds of *N*-methyl-*N*-oxyamides†

**DOI:** 10.1039/d3sc03216g

**Published:** 2023-08-29

**Authors:** Monika Malik, Raffaele Senatore, Thierry Langer, Wolfgang Holzer, Vittorio Pace

**Affiliations:** a Department of Pharmaceutical Sciences, Division of Pharmaceutical Chemistry, University of Vienna Josef-Holaubek-Platz 2 1090 Vienna Austria vittorio.pace@univie.ac.at; b Department of Chemistry, University of Turin Via Giuria 7 10125 Turin Italy

## Abstract

Due to the well known reactivity of C(O)–N functionalities towards canonical C1-homologating agents (*e.g.* carbenoids, diazomethane, ylides), resulting in the extrusion of the *N*-centered fragment *en route* to carbonyl compounds, formal C1-insertions within N–O bonds still remain obscure. Herein, we document the homologative transformation of *N*-methyl-*N*-oxyamides – with high tolerance for diverse *O*-substituents – into *N*-acyl-*N*,*O*-acetals. Under controlled basic conditions, the *N*-methyl group of the same starting materials acts as a competent precursor of the methylene synthon required for the homologation. The logic is levered on the formation of an electrophilic iminium ion (*via* N–O heterolysis) susceptible to nucleophilic attack by the alkoxide previously expulsed. The procedure documents genuine chemocontrol and flexibility, as judged by the diversity of substituents placed on both amide and nitrogen linchpins. The mechanistic rationale was validated through experiments conducted on D-labeled materials which unambiguously attributed the origin of the methylene fragment to the *N*-methyl group of the starting compounds.

## Abstract 2

Due to the well known reactivity of C(O)–N functionalities towards canonical C1-homologating agents (*e.g.* carbenoids, diazomethane, ylides), resulting in the extrusion of the *N*-centered fragment *en route* to carbonyl compounds, formal C1-insertions within N–O bonds still remain obscure.

## Graphical abstract

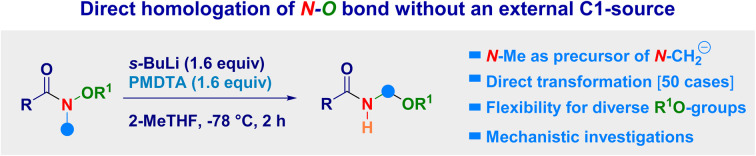

## Introduction

**Scheme 1:**
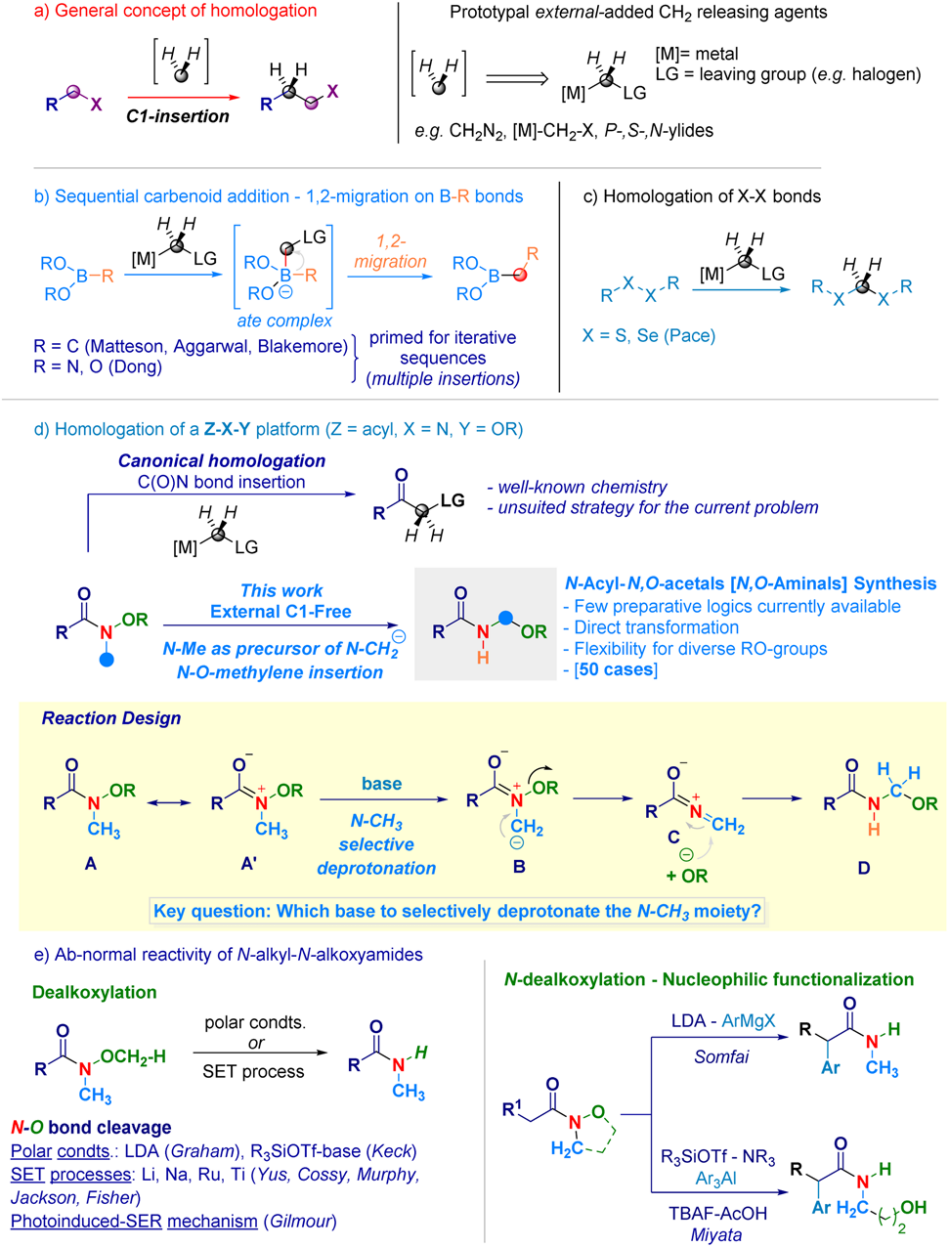
General context of the presented work.

Homologation techniques enable the progressive insertion of a constant unit (*e.g.* –*CH*_*2*_–) within a given chemical bond.^1^ These operations are playing nowadays a prominent role in the assembly of complex molecular entities and exhibit a significant potential in guiding the fine-tuning of crucial physical–chemical parameters.^2^ Since the introduction almost one century ago by Arndt and Eistert of the archetypal homologating agent^3^ – diazomethane (CH_2_N_2_) –, subsequent studies converged in individuating the LG-*CH*_*2*_-[M] motif as the general descriptor for C1-releasing agents (Scheme 1a). Mechanistically, the transformation can be represented as the formal delivery of the C1 unit from a donor – externally added – agent to a recipient linchpin which, through the triggering of 1,2-migration-type events furnishes the homologated skeleton.^4^ The venerable Matteson homologation of boronates with lithium carbenoids (nucleophilic C1-agents, Scheme 1b)^5^ elegantly extended to iterative processes by Aggarwal,^6^ and Blakemore^7^ – well illustrates the controlled insertions into C–B bonds and served as source of inspiration for the more recent work on N–B and O–B bond homologations by Dong.^8^ In the course of our studies on programmable LiCH_2_X-mediated homologation of carbon electrophiles,^9^ we validated the rationale for the selective insertion of methylene units into chalcogenide bonds (S–S and Se–Se analogues) to construct geminal disulfurated methanes (dithioacetal-like – Scheme 1c).^10^ One intriguing aspect is whether a C1-insertion could take place selectively on a more sophisticated Z–X–Y platform, where both Z–X and X–Y bonds might undergo homologation (Scheme 1d): to the best of our knowledge, these clusters have not been previously investigated in C1-insertion sequences. In this context, *N*-alkoxy-substituted amides^11^ constitute representative models since both the C(O)–N and the N–O fragments could be subjected to the methylene insertion: as documented in recent work by our group,^12^ amide-type substrates undergo homologation (employing [M]-CH_2_-LG reagents) at the C(O)–N linkage – yielding α-substituted ketones^11*b*,12*c*,13^ – thus, precluding *de facto* the use of such agents for inserting the C1-unit within the N–O bond (Scheme 1d – up). Beside introducing the concept of N–O homologation, the prospected transformation would provide a modular and rapid approach towards challenging – yet stable^14^ – *acyclic N*-acyl-*N*,*O*-acetals (*i.e. N*,*O*-aminals).^15^ Noteworthy, the preparation of these analogues – *i.e.* RN(H)–*CH*_*2*_–OR^1^ motif – has been much less explored compared to methods for assembling substituted congeners and, still remains circumscribed to the electrochemical oxidation of aliphatic amides introduced by Linstead,^16^ or to the Katritzky's amidoalkylation of alcohols.^17^

In this scenario, we wondered if the homologating element (–*CH*_*2*_–) could be generated from the constitutive *N*-methyl substituent of the *N*-alkoxy amide through a deprotonative event. Accordingly, a *N*-methyl-*N*-methoxyamide^13*a*^A represents a pertinent model to make productive the concept: as anticipated, it possesses – as judged by the NC(O) delocalization (A ↔ A′) – relatively acidic *N*-methyl protons amenable to removal with a base of adequate strength. In this circumstance, a ylide-type intermediate B would be formed prior to its collapse – *via* expulsion of the alkoxide – to a terminal, electrophilic iminium ion C,^18^ capable of intercepting the same nucleophile (RO^−^) and, thus yielding the homologated adduct D. A series of critical aspects required fine tuning, also in view of the hitherto explored chemistry of the O–*CH*_*3*_ moiety of Weinreb-like amides functional to access secondary amides (Scheme 1e). In this context, Graham pointed out that under polar conditions – the lithium amide base LDA (p*K*_a_ = 36)^19^ selectively deprotonates the O–*CH*_*3*_ group giving a demethoxylated amide (*via* E2 mechanism),^20^ thus predicting a critical role of the base for generating the requested ylide.^21^ To further complicate this base-mediated scenario, enolizable *N*-alkyl-*N*-alkoxyamides^22^ can serve as manifolds for sequential *N*-dealkoxylation – nucleophilic functionalization to gather α-substituted amides, as documented by Somfai^23^ and Miyata.^24^ Moreover, N–O breaking operations on *N*-alkyl-*N*-alkoxyamides benefited from adopting (metal) based single electron transfer logics,^25^ for which Gilmour in 2022 presented an elegant organocatalytic photoinduced alternative (Scheme 1e).^26^

Despite these precedents on the N–O bond cleavage, the seminal work by Beak^27^ on the lithiation of *N*,*N*-dimethylamides supported our initial hypothesis of selectively deprotonate the N–*CH*_*3*_ fragment. Herein, we report the homologative assembly of *N*-acyl-*N*,*O*-acetals starting from *N*-alkoxy-*N*-methyl-amides through the use of the same *N*-methyl group of alkoxyamide as a formal C1-delivering agent. Our findings shed light on a novel reactivity – beyond classical acylation and enolate chemistry – of *N*-alkoxyamides in which the C(O)N bond maintains its chemical integrity in a carbanion environment while, the labile N–O linkage (55–65 kcal mol^−1^)^26,28^ undergoes homologation.

## Results and discussion

The enantiomerically pure Weinreb amide 1 generated from the common nonsteroidal anti-inflammatory drug Ibuprofen was chosen as the model substrate (Table 1). Upon treatment with *s*-BuLi in THF at −78 °C (entry 1), the corresponding ketone 2b – derived from classical Weinreb amide chemistry – was obtained as the unique product (with traces of the demethoxylated adduct 2a). Pleasingly, by triggering the deagreggation (and thus the basicity)^29^ of *s*-BuLi through the use of the bidentate ligand TMEDA (*N*,*N*,*N*′,*N*′-tetramethylethylendiamine), the deprotonation of the N–Me group became evident, thus furnishing the desired *N*-acyl-*N*,*O*-acetal 2 in a promising 29% yield, though neither formation of the Graham^20*a*^ secondary amide 2a nor of *s*-butyl ketone 2b could be suppressed (entry 2). Running the reaction in diethyl ether (entry 3) or, increasing the loading of the additive resulted in no evident improvement (entry 4). The chemoselectivity slightly augmented in 2-methyltetrahydrofuran, probably, as a consequence of its higher stability under strongly basic conditions (entry 5).^30^ Considering Bauer's observation on the complete deaggregation of *s*-BuLi in the presence of the tridentate additive PMDTA (*N*,*N*,*N*′,*N*′′,*N*′′-pentamethyldiethylentriamine),^31^ we were pleased in noting the almost exclusive formation of the desired adduct 2, thus confirming the generation of the aminomethyl anion and the avoiding of Weinreb amides enolization phenomena (entry 6).^22*e*^ The noticeable dwindling of the yield in THF is presumably ascribed to the easier α-lithiation it undergoes – compared to 2-MeTHF – (entry 7),^30,32^ thus suggesting a strict dependance of the N–CH_3_ deprotonation with the overall basicity of the reaction medium. Moreover, conducting the process in 2-MeTHF guarantees the full integrity of the stereochemical information compared to THF in which racemization phenomena became apparent (entry 6 *vs.* 7).^32^ Some additional points merit mention: (a) reactions reach completion within 2 h, as judged by lowering the reaction time to 0.5 h (entry 8); (b) keeping the stoichiometric loading of *s*-BuLi and PMDTA at 1.6 equiv. enables to maximize the formation of the desired product 2, thus avoiding a detrimental effect on the chemoselectivity due to less controlled lithiations (entry 9); (c) replacing PMDTA with analogous polyamine ligands such as HMTETA, Me_6_TREN, BDMAEE or (−)-sparteine again affected the selectivity, thus confirming the unique beneficial effect displayed by PMDTA (entries 10–14); (d) switching to alkyllithiums of distinct basicity [*i.e.* more basic *t*-BuLi (p*K*_a_ = 53) and less alkaline *n*-BuLi (p*K*_a_ = 50)]^19^ resulted in substantial lack of control of the transformation (entries 14–15), while the lithium amide LTMP (p*K*_a_ = 35.7)^19^ shifted the distribution towards the demethoxylated product 2a (entry 16) in analogy to the previously used LDA;^20*a*^ (e) increasing the temperature to −40 °C lowered the reaction efficiency, as a consequence of the plausible diminished stability of the generated anion (entry 17).

**Table 1:** Reaction optimization^a^

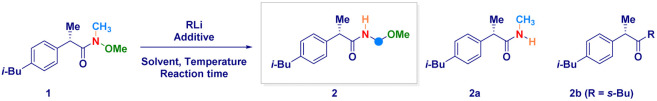				
Entry	RLi	Additive (equiv.)	Solvent	Product(s)^b^ (%)
1	*s*BuLi	—	THF	2 (—); 2a (6); 2b (90)
2	*s*BuLi	TMEDA (1.6)	THF	2 (29); 2a (17); 2b (34)
3	*s*BuLi	TMEDA (1.6)	Et_2_O	2 (23); 2a (20); 2b (31)
4	*s*BuLi	TMEDA (3.2)	THF	2 (30); 2a (24); 2b (27)
5	*s*BuLi	TMEDA (1.6)	MeTHF	2 (48); 2a (11); 2b (8)
6^c^	*s*BuLi	PMDTA (1.6)	MeTHF	2 (81)
7^d^	*s*BuLi	PMDTA (1.6)	THF	2 (59); 2a (15); 2b (12)
8^e^	*s*BuLi	PMDTA (1.6)	MeTHF	2 (62)
9^f^	*s*BuLi	PMDTA (3.2)	MeTHF	2 (64); 2a (15)
10	*s*BuLi	HMETA (1.6)	MeTHF	2 (43); 2a (22); 2b (30)
11	*s*BuLi	Me_6_TREN (1.6)	MeTHF	2 (35); 2a (16); 2b (24)
12	*s*BuLi	(−)-Spart. (1.6)	MeTHF	2 (51); 2a (23); 2b (18)
13	*s*BuLi	BDMAEE (1.6)	MeTHF	2 (47); 2a (18); 2b (14)
14	*t*BuLi	PMDTA (1.6)	MeTHF	2 (36); 2a (15); 2b (21)
15	*n*BuLi	PMDTA (1.6)	MeTHF	2 (12); 2a (19); 2b (37)
16	LTMP	—	MeTHF	2 (5); 2a (80)
17^g^	*s*BuLi	PMDTA (1.6)	MeTHF	2 (46); 2a (8); 2b (10)
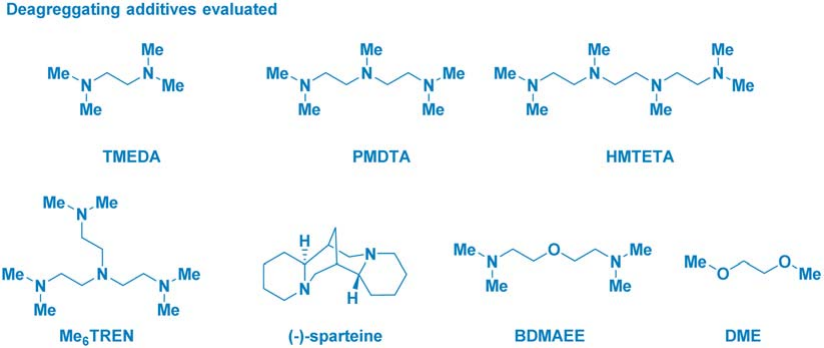				

a Unless otherwise stated, reactions were run at −78 °C for 2 h in the presence of 1.6 equiv. of the competent organolithium.

b Isolated yields.

c Compound 2 was obtained in 99 : 1 er.

d Compound 2 was obtained in 95 : 5 er.

e Reaction time 0.5 h.

f *s*-BuLi (3.2 equiv.) was used.

g Reaction run at −40 °C.

**Scheme 2:**
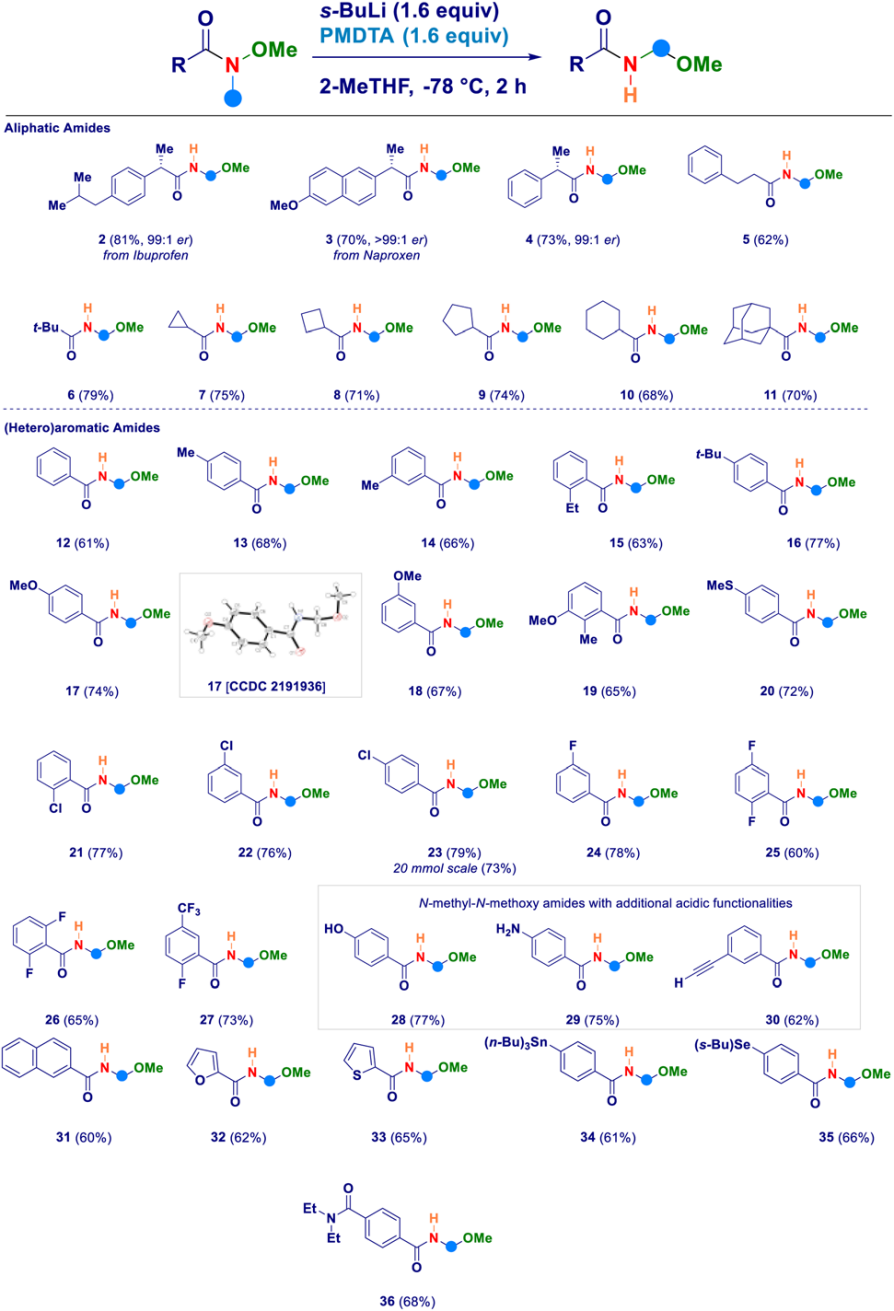
Scope of the method with *N*-methyl-*N*-methoxyamides.

Having established the four key elements governing the reaction (*s*-BuLi, PMDTA, 2-MeTHF, −78 °C), we then studied the scope of the intramolecular *N*-methyl-*N*-alkoxyamide homologation to *N*-acyl-*N*,*O*-acetals (Scheme 2). A series of Weinreb amides generated from aliphatic carboxylic acids were amenable substrates for the process: similarly to the ibuprofene analogue 2, also the aproxene derived species smoothly underwent the transformation (3) with full retention of the embodied stereochemical information. This latter aspect was also deducted in the case of compound 4, thus confirming that the employed strongly basic reaction conditions did not promote any racemization of the materials. Not only aliphatic Weinreb amides of different chemical hindrance [phenylethyl (5) and *tert*-butyl (6)] on the carbonyl carbon could be used, but also a series of alicyclic analogues of progressive ring size such as cyclopropyl (7), cyclobutyl (8), cyclopentyl (9), cyclohexyl (10) and the sterically demanding adamantyl derivative (11). Switching to aromatic Weinreb amides did not alter the validity of the general methodology. In this context, common aliphatic substituents could be conveniently placed at different positions of the phenyl ring [*e.g.* unsubstituted (12), *p*-methyl (13), *m*-methyl (14), *o*-ethyl (15), *p-tert*-butyl (16)]. The presence of elements modulating the electrophilicity of carbonyl carbon was tolerated, as documented in the cases of: (i) electron-donating substituents such as ethers (methoxy-, 17–19) or thioether (20) and, (ii) halogen atoms [chloro (21–23), fluoro (24–26), trifluoromethyl (27)]. The X-ray structural analysis of derivative 17 unambiguously confirmed the *N*-acyl-*N*,*O*-acetal cluster. Scaling up to 20 mmol scale did not influence the reaction yield, as shown in the case of compound 23. Notably, the inclusion of additional acidic functionalities [OH of a phenol (28), NH_2_ of an aniline residue (29) or C–H of a terminal alkyne (30)] were not detrimental for the homologative event whenever substrates were pretreated with MeLi (see ESI†). Moreover, polyaromatic [2-naphtyl (31)] and heteroaromatic [furyl (32) and thienyl (33)] systems further expanded the reaction scope. The deprotonation *en route* to the intermediate aminomethyl species did not suffer from the simultaneous presence of functionalities susceptible to lithiation, as deducted in the cases of the stannane (34) and the selenane (35). With much of our delight, the methodology enabled the selective transformation of a Weinreb amide featuring a carboxy-amide fragment (*N*,*N*-diethyl) into the corresponding *N*-acyl-*N*,*O*-acetal 36 in which no modification occurred to the functionalizing element. This is indeed an evident proof of the selective deprotonation occurring at the *N*-methyl group of the amides.

**Scheme 3:**
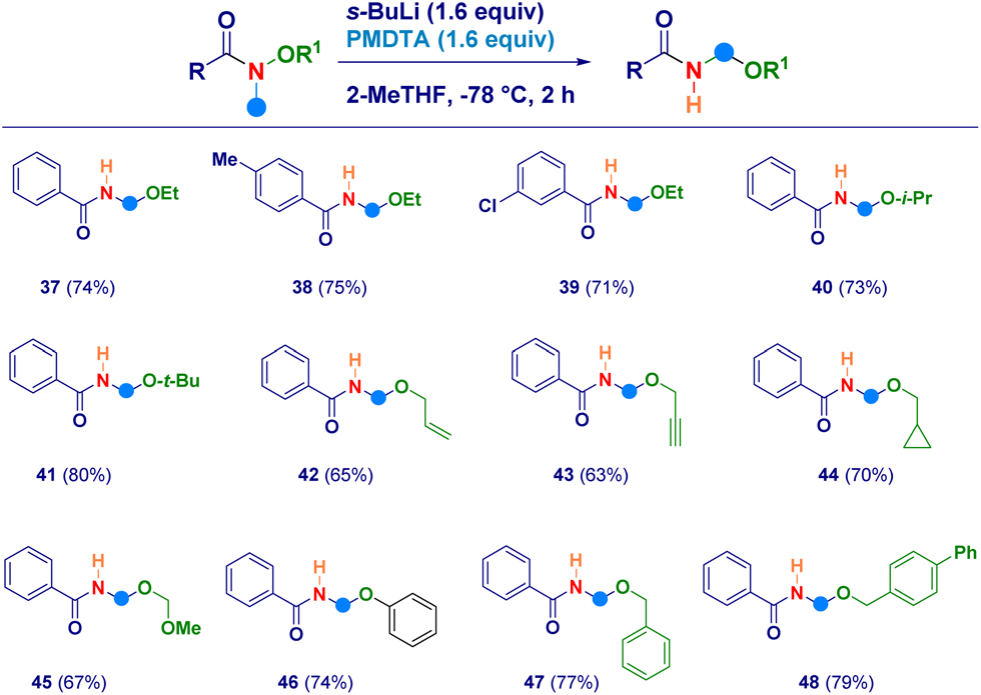
Synthesis of diverse *O*-substituted *N*-acyl-*N*,*O*-acetals.

With the aim to uncover the full potential of the reaction, the evaluation of different *O*-substituted *N*,*O*-alkoxyamides was performed (Scheme 3). In this sense, the scope of Weinreb amides discussed above was efficiently implemented by engaging as reaction partners not only the *O*-ethyl analogues (37–39) but also the more sterically hindered *O-i*-propyl (40) and *O-t*-butyl (41) ones. The initial N–CH_3_ deprotonation event was successfully realized also on the corresponding *O*-allyl (42) and the *O*-propargyl (43) systems, as well as, on the *O*-cyclopropylmethyl (44) and the *O*-methoxymethyl (45) analogues. The high electrophilic reactivity of the iminium species involved, enabled the smooth attack of a less nucleophilic element as the phenolate anion, thus furnishing derivative 46. Finally, also *N*-methyl-*O*-benzyl-type alkoxyamides yielded the expected *N*-acyl-*N*,*O*-acetals 47 and 48 in comparable efficiency.

**Scheme 4:**
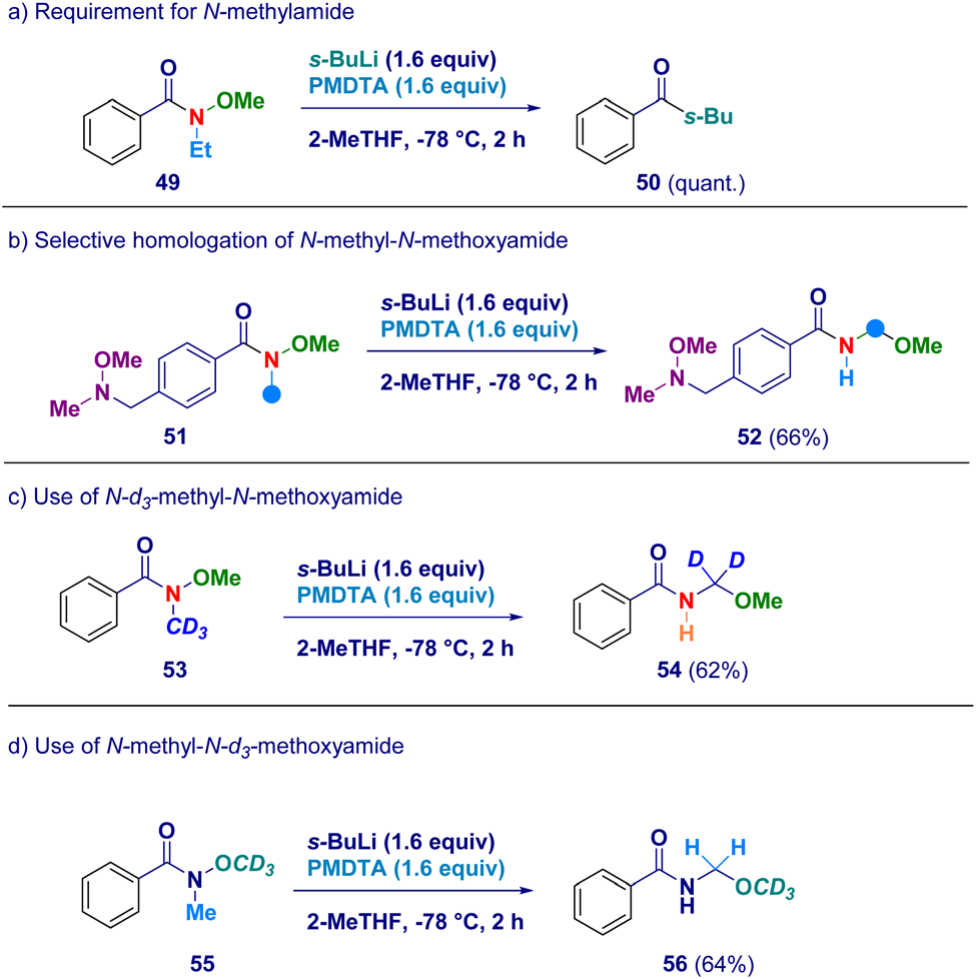
Control experiments for validating the mechanistic hypothesis.

In order to confirm the proposed rationale, a series of control experiments were performed (Scheme 4). The pivotal presence of the *N*-methyl group as the element furnishing the active methylene unit was deducted by attempting the transformation on the *N*-ethyl-*O*-methoxy analogue 49 which simply reacted *via* canonical Weinreb mechanism giving ketone 50 as the unique product (path a). It is likely that N–*C****H***_***2***_CH_3_ protons would exhibit lower acidity preventing the crucial deprotonation event with *s*-BuLi/PMDTA. Additionally, this result is also in agreement with the suitability of our conditions for preventing Graham demethoxylation.^20*a*^ The critical role displayed by the acyl moiety was evidenced by employing a Weinreb amide featuring a *N*-methoxy-*N*-methyl amine fragment (51) which yielded exclusively *N*-acyl-*N*,*O*-acetal 52 (path b), thus suggesting the requirement for an amide functionality to promote the reaction. The unambiguous deciphering of the origin of the constitutive methylene unit of *N*-acyl-*N*,*O*-acetals was gathered by applying the procedure to deuterium-labeled systems. Accordingly, *N-d*_3_-methyl-*N*-methoxy amide (53) was converted into the *d*_2_-*N*-acyl-*N*,*O*-acetal 54, thus demonstrating that the effective source for the homologated N–O linkage was the N–CD_3_ group (path c). *Ceteris paribus*, *N*-[^1^H]methyl-*N-d*_3_-methoxy amide (55) was homologated to the corresponding *N*-acyl-*N*,*O*-[^1^H]-acetal 56 featuring the labeled element exclusively at the alkoxy portion (OCD_3_ – path d).

**Scheme 5:**
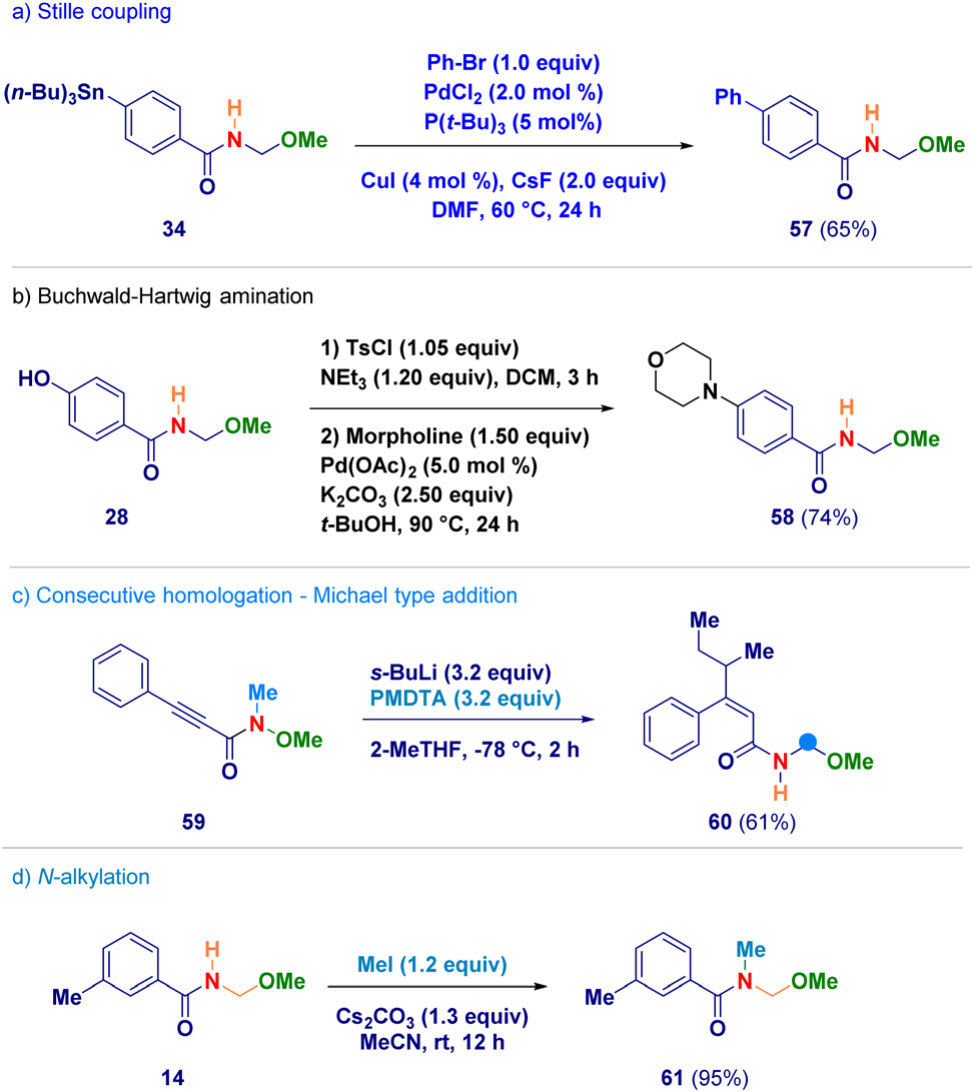
Synthetic manipulation of *N*-acyl-*N*,*O*-acetals.

Finally, selected *N*-acyl-*N*,*O*-acetals were employed as starting materials for standard derivatization chemistry (Scheme 5). The stannane containing analogue 34 was used in a Stille coupling furnishing the biphenyl adduct 57 (path a);^33^ upon tosylation of phenol-derivative 28, followed by a Buchwald–Hartwig amination with morpholine, compound 58 was obtained (path b);^34^ the alkynyl-type Michael acceptor 59 reacted – in the presence of an excess of *s*-BuLi – giving *N*-acyl-*N*,*O*-acetal 60 in which the excess of *s*-BuLi attacked the β-position (path c); the secondary amide functionality of adduct 14 could be efficiently alkylated under basic conditions generating 61 (path d).

In summary, we have introduced – through the homologative transformation of *N*-methyl-*N*-oxyamides to acyclic *N*-acyl-*N*,*O*-acetals – the concept of C1-installation within a N–O bond. The constitutive *N*-methyl moiety of the starting material acted as the source of the methylene unit. Because of the well-established reactivity of *N*-alkoxyamides in the presence of canonical homologating agents (*e.g.* [M]-CH_2_-LG), the tactic proposed herein appears an effective tool for inserting the CH_2_ group into formal oxy-amines functionalities. The controlled deprotonating event, conducted with deaggregated *s*-BuLi (PMDTA), guarantees the tailored genesis of a α-aminomethyl carbanion which spontaneously rearranges to an electrophilic iminium intercepting the released alkoxide. Almost no restriction on the substitution pattern across the whole amide functionality – and, in particular on the *N*-alkoxy element – was observed, thus making flexible the access to the targeted compounds. The crucial role displayed by the *N*-methyl group was definitively ascertained through the application of the protocol to deuterium-labeled analogues.

## Data availability

Further details of the experimental procedure, ^1^H, ^13^C, ^19^F, ^77^Se and ^119^Sn NMR spectra, HPLC analyses and X-ray crystallographic data for 17 are available in the ESI.†

## Author contributions

M. M. and R. S. performed the experiments. W. H. realized NMR analyses and elucidated the structures. V. P. conceived and directed the project and wrote the manuscript. All the authors discussed the results.

## Conflicts of interest

The authors declare no competing financial interests.

## Supplementary Material

SC-014-D3SC03216G-s001

SC-014-D3SC03216G-s002
