# Supplementary material for: Base-mediated homologative rearrangement of nitrogen–oxygen bonds of N-methyl-N-oxyamides
Source: Chem Sci. 2023 Aug 29;14(37):10140–6. doi: 10.1039/d3sc03216g (PMC10530184; doi:10.1039/d3sc03216g)
Supplement: SC-014-D3SC03216G-s001 [file SC-014-D3SC03216G-s001.pdf]

Electronic Supplementary Material (ESI) for Chemical Science.
This journal is © The Royal Society of Chemistry 2023

ELECTRONIC SUPPORTING INFORMATION

for

Base-mediated Homologative Rearrangement of Nitrogen-Oxygen Bonds of N-Methyl-N-oxyamides

Monika Malik,[a] Raffaele Senatore,[a] Thierry Langer,[a] Wolfgang Holzer[a] and Vittorio Pace *[a,b]

[a] University of Vienna - Department of Pharmaceutical Sciences, Division of Pharmaceutical Chemistry - Josef-Holaubek-Platz 2, 1090 Vienna, Austria.

[b] University of Turin - Department of Chemistry - Via P. Giuria 7, 10125 Turin, Italy.

e-mail: vittorio.pace@univie.ac.at; vittorio.pace@unito.it

TABLE OF CONTENTS

Instrumentation and general analytical methods	S2
Synthesis of starting materials and their characterization	S3
General procedure for the synthesis of *N*-(alkoxymethyl)amides	S14
N-(alkoxymethyl)amides: Experimental procedures and characterization	S15
Copies of ^{1}H- and ^{13}C-NMR spectra	S47
Copies of heteronuclei NMR spectra for relevant compounds (^{19}F, ^{119}Sn, ^{77}Se)	S121
X-Ray Analysis for compound **17**	S124
References	S128

Instrumentation and General Analytical Methods

Melting points were determined on a Reichert–Kofler hot-stage microscope and are uncorrected. Mass spectra were obtained on a Shimadzu QP 1000 instrument (EI, 70 eV) and on a Bruker maXis 4G instrument (ESI-TOF, HRMS). ^{1}H, ^{13}C, ^{19}F, ^{119}Sn and ^{77}Se NMR spectra were recorded with a Bruker Avance III 400 spectrometer (400 MHz for ^{1}H, 100 MHz for ^{13}C, 40 MHz for ^{15}N, 376 MHz for ^{19}F and 76 MHz for ^{77}Se) at 297 K using a directly detecting broadband observe (BBFO) probe. ^{1}H and ^{13}C NMR spectra were additionally recorded with a Bruker 200 spectrometer (200 MHz for ^{1}H, 50 MHz for ^{13}C). The center of the (residual) solvent signal was used as an internal standard which was related to TMS with δ 7.26 ppm (^{1}H in $CDCl_3$), 7.16 ppm (^{1}H in C_6D_6), 2.05 ppm (^{1}H in acetone-d_6) and δ 77.0 ppm (^{13}C in $CDCl_3$), 128.06 (^{13}C in C_6D_6), 202.26 – 29.84 ppm (^{13}C in acetone-d_6).[1] When recording ^{1}H spectra in acetone-d_6, occasionally it could be observed the water signal in this deuterated solvent at δ 2.84 ppm; in case of recording ^{13}C spectra in acetone-d_6 it could be noticed overlapping of alkyl-type signals at *ca.* δ 29.8 ppm. ^{15}N NMR (gs-HMBC) spectra were referenced against neat, external nitromethane. ^{19}F NMR spectra were referenced via the Ξ ratio (absolute referencing). ^{77}Se spectra were referenced against diphenyldiselane (δ Ph_2Se_2 463 ppm). ^{119}Sn NMR spectra were referenced against external Me_4Sn (0.0 ppm). Spin-spin coupling constants (*J*) are given in Hz.
In nearly all cases, full and unambiguous assignment of all resonances was performed by combined application of standard NMR techniques, such as APT, HSQC, HMBC, HSQCTOCSY, COSY and NOESY experiments.

Starting materials were prepared as detailed below; those ones not reported in the manuscript are indicated with the descriptor **nn-*sm***, being **nn** the corresponding final compound in the manuscript. Enantiopure and racemic compounds for obtaining (*S*)-**4** and *rac*-**4** were obtained according to our previous work.[2]

All reactions were performed under an inert atmosphere of argon using standard schlenk techniques. Solvents used for running reactions were distilled over Na/benzophenone; additives were redistilled prior to use. Organolithiums were titrated prior to use. Chemicals were purchased from SigmaAldrich, Acros, Alfa Aesar, Fluorochem and TCI Europe. Solutions were evaporated under reduced pressure with a rotary evaporator. For column chromathography, silica Gel 60 (0.04-0.063 mm) was used. TLC was carried out on aluminium sheets precoated with silica gel 60F254 (Merchery-Nagel, Merk); the spots were visualised under UV light (λ = 254 nm) and/or $KMnO_4$ (aq.) was used as revealing system.

Synthesis of Starting Materials

Most of *N*-alkyl-*N*-alkoxyamides employed as starting materials are known substrates and were prepared as indicated below.

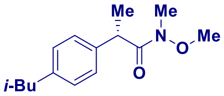

Gaunt, M. J. *et al.* *Angew. Chem. Int. Ed.* **2011**, *50*, 463

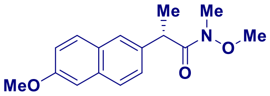

Gaunt, M. J. *et al.* *Angew. Chem. Int. Ed.* **2011**, *50*, 463

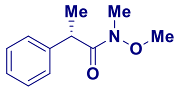

Pace, V. *et al.* *Adv. Synth. Catal.* **2020**, *362*, 5056

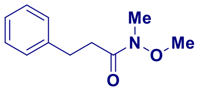

Pace, V. *et al.* *Adv. Synth. Catal.* **2020**, *362*, 5056

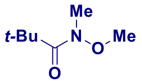

Pace, V. *et al.* *Org. Biomol. Chem.* **2015**, *13*, 1969

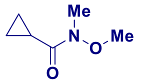

Pace, V. *et al.* *Adv. Synth. Catal.* **2020**, *362*, 5056

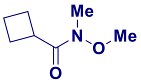

Alami, M. *et al.* *Org. Lett.* **2010**, *12*, 4042

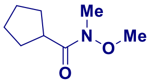

Gunda, G. I. *et al.* *Org. Lett.* **2000**, *2*, 4091

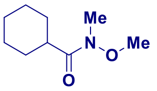

Pace, V. *et al.* *Adv. Synth. Catal.* **2020**, *362*, 5056

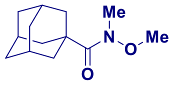

Pace, V. *et al.* *Org. Biomol. Chem.* **2015**, *13*, 1969

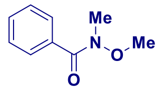

Pace, V. *et al.* *Chem. Commun.* **2016**, *52*, 7584

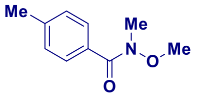

Pace, V. *et al.* *Chem. Commun.* **2016**, *52*, 7584

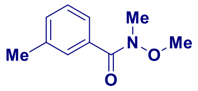

Pinney, K. G. *et al.* *Bioorg. Med. Chem. Lett.* **2010**, *20*, 1415

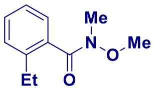

Pace, V. *et al.* *Org. Lett.* **2019**, *21*, 8261

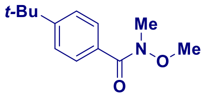

Pace, V. *et al.* *Chem. Commun.* **2016**, *52*, 7584

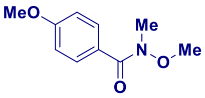

Pace, V. *et al.* *Chem. Commun.* **2016**, *52*, 7584

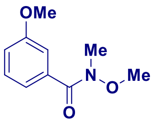

Pace, V. *et al.* *Chem. Commun.* **2016**, *52*, 7584

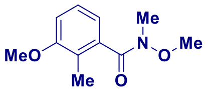

Pace, V. *et al.* *Chem. Commun.* **2016**, *52*, 7584

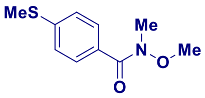

Pace, V. *et al.* *Org. Lett.* **2019**, *21*, 8261

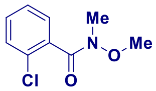

Pace, V. *et al.* *Org. Biomol. Chem.* **2015**, *13*, 1969

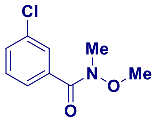

Pace, V. *et al.* *Org. Lett.* **2019**, *21*, 8261

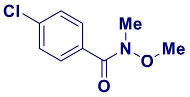

Pace, V. *et al.* *Org. Lett.* **2019**, *21*, 8261

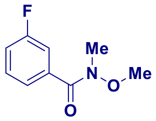

Pace, V. *et al.* *Org. Lett.* **2019**, *21*, 8261

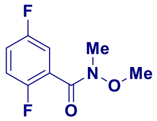

Buchwald, S. L.. *et al.* *Org. Lett.* **2006**, *8*, 4843

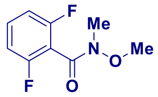

Pinney, K. G. *et al.* *Bioorg. Med. Chem. Lett.* **2010**, *20*, 1415

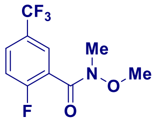

Pace, V. *et al.* *Chem. Commun.* **2016**, *52*, 7584

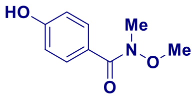

Hattori, K. *et al.* *J. Med. Chem.* **2008**, *51*, 1925

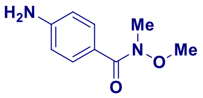

Doo, O. *et al.* *Bull. Chem. Korean Soc.* **2010**, *31*, 171

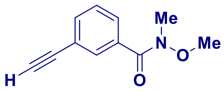

Pace, V. *et al.* *Chem. Commun.* **2016**, *52*, 7584

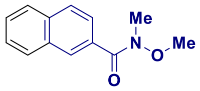

Pace, V. *et al.* *Org. Lett.* **2019**, *21*, 8261

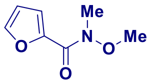

Pace, V. *et al.* *Chem. Commun.* **2017**, *53*, 9498

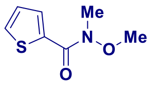

Pace, V. *et al.* *Chem. Commun.* **2017**, *53*, 9498

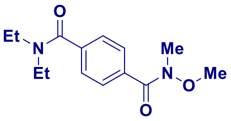

Pace, V. *et al.* *Adv. Synth. Catal.* **2020**, *362*, 5056

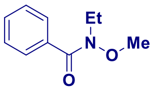

Ackermann, L. *et al.* *Org. Lett.* **2013**, *15*, 718

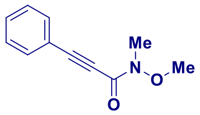

Pace, V. *et al.* *Org. Lett.* **2019**, *21*, 8261

2-(4-isobutylphenyl)-*N*-methoxy-*N*-methylpropanamide (1)

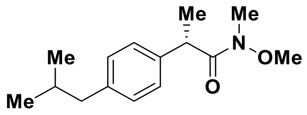

To a 50 mL round bottom flask equipped with stir bar was added (*S*)-(+)-2-(4-isobutylphenyl)propanoic acid ((*S*)-Ibuprofen) (1.00 g, 4.85 mmol, 1 equiv) and dichloromethane (13 mL, ≈ 0.3M). To this stirred solution was added 1,1′-carbonyldiimidazole (0.87 g, 5.34 mmol, 1.1 equiv) in one portion. The solution was allowed to stir for 45 minutes. At this time, *N,O*-dimethylhydroxylamine hydrochloride (0.52 g, 5.34 mmol, 1.1 equiv) was added and the reaction mixture was stirred for six hours. The reaction mixture was then quenched with 15 mL of 1 M HCl and stirred vigorously for 10 minutes. The layers were separated and the aqueous layer was extracted with DCM (2 x 30 mL). The combine organic layers were washed with 1 M HCl, deionized water and a 1:1 mixture of brine and a saturated sodium bicarbonate solution. The organic layer was dried over Na_2SO_4 and the solvent was removed under reduced pressure to afford the pure compound **1** in 85% yield (1.03 g) as a colorless oil.

The corresponding racemic sample has been prepared starting from racemic 2-(4-isobutylphenyl)propanoic acid and spectroscopic data match with those ones reported below.

^{1}H NMR (400 MHz, $CDCl_3$) δ: 7.18 (m, 2H, Ph H-2,6), 7.06 (m, 2H, Ph H-3,5), 4.10 (m, COC<u>H</u>CH_3), 3.36 (s, 3H, $NOCH_3$), 3.12 (s, 3H, NCH_3), 2.41 (d, *J* = 7.2 Hz, 2H, C<u>H</u>$_2$CH(CH_3)$_2$), 1.81 (m, 1H, CH_2C<u>H</u>(CH_3)$_2$), 1.41 (d, *J* = 7.0 Hz, 3H, CHC<u>H</u>$_3$), 0.86 (d, *J* = 6.6 Hz, 6H, CH_2CH(C<u>H</u>$_3$)$_2$).

^{13}C NMR (100 MHz, $CDCl_3$) δ: 175.4, 139.8, 138.9, 129.1, 127.1, 60.8, 44.8, 41.4, 32.1, 30.0, 22.2, 19.4.

^{15}N NMR (40 MHz, Acetone-d_6) δ: -260.6 (NH).

HRMS (ESI), *m/z*: calcd. for $C_{15}H_{23}NNaO_2^+$: 272.1621 $[M + Na]^+$; found: 272.1630.

HPLC analysis: Chiralpak IG Column, λ 254 nm, eluent: *n*-hexane / *i*-propanol 90:10. Flow 1 mL/min.

$[\alpha]_D^{25}$ = + 38 (*c* = 1, $CHCl_3$, lit.[3] + 40, *c* = 1.1, $CHCl_3$)

HPLC Charts for Compound 1

Racemate

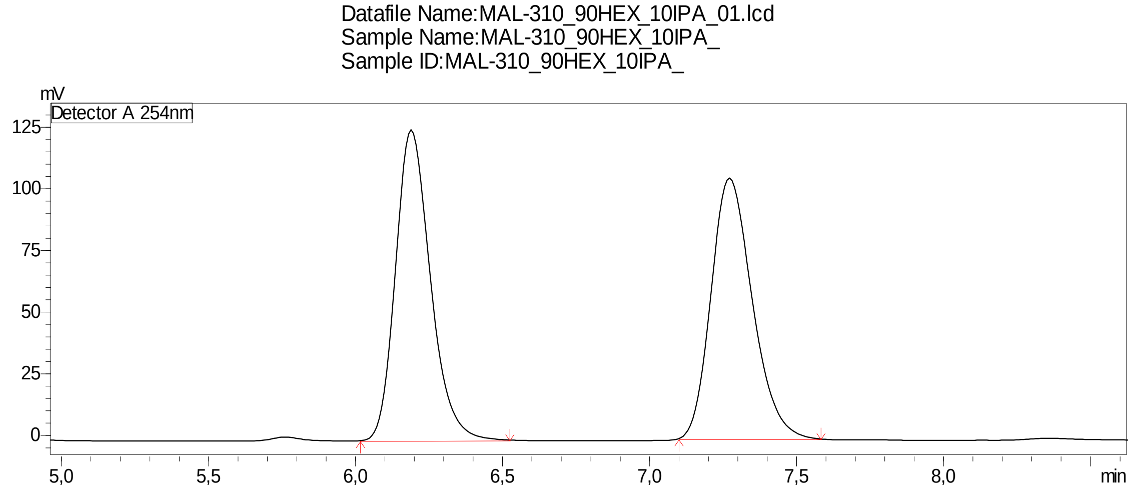

Peaks	Retention time (min)	Area	Area %
1	6,192	1039054	50,157
2	7,274	1032553	49,843
Total		2071607	100,000

Enantioenriched

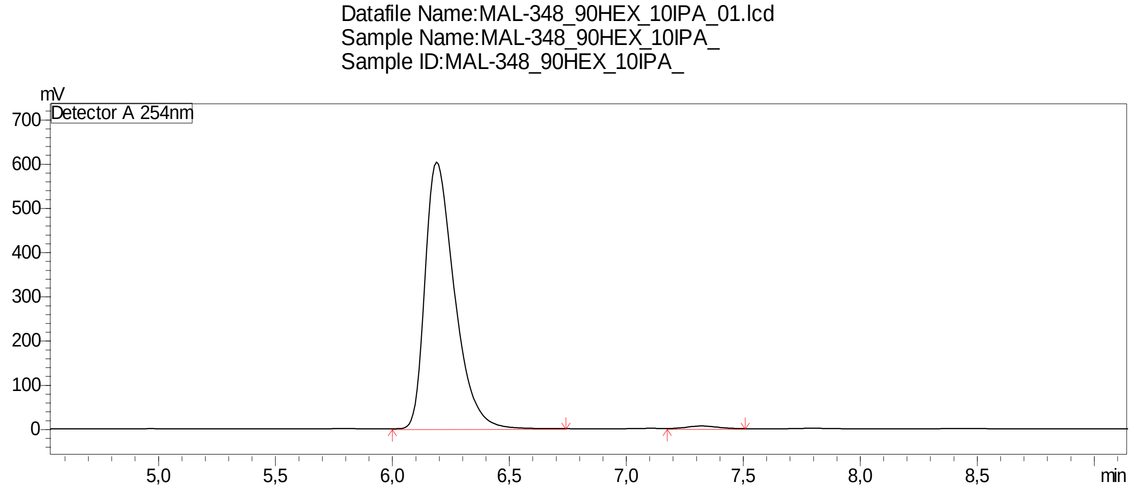

Peaks	Retention time (min)	Area	Area %
1	6,192	5261199	99,061
2	7,326	49896	0,939
Total		5311094	100,000

***N*-methoxy-2-(6-methoxynaphthalen-2-yl)-*N*-methylpropanamide (3-*sm*)**

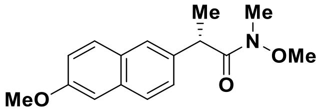

To a 50 mL round bottom flask equipped with stir bar was added (*S*)-(+)-2-(6-methoxy-2-naphthyl)propionic acid ((*S*)-naproxen) (1.00 g, 3.9 mmol, 1 equiv) and dichloromethane (13 mL, ≈ 0.3M). To this stirred solution was added 1,1'-carbonyl diimadazole (0.7 g, 4.3 mmol, 1.1 equiv) in one portion. The solution was allowed to stir for 45 minutes. At this time, N-O-dimethylhydroxylamine hydrochloride (0.42 g, 4.3 mmol, 1.1 equiv) was added and the reaction mixture was stirred for six hours. The reaction mixture was then quenched with 15 mL of 1 M HCl and stirred vigorously for 10 minutes. The layers were separated and the aqueous layer was extracted with DCM (2 x 30 mL). The combine organic layers were washed with 1 M HCl, deionized water and a 1:1 mixture of brine and a saturated sodium bicarbonate solution. The organic layer was dried over Na_2SO_4 and the solvent was removed under reduced pressure to afford the pure compound **3-*sm*** in 85% yield (0.9 g) as a white solid.

The corresponding racemic sample has been prepared starting from racemic 2-(6-methoxy-2-naphthyl)propionic acid. Spectroscopic data match with those ones reported below.

mp = 59-61 °C

^{1}H NMR (400 MHz, $CDCl_3$) δ: 7.69 (m, 3H), 7.43 (m, 1H), 7.11 (m, 2H), 4.27 (m, 1H), 3.90 (s, 3H), 3.40 (s, 3H), 3.17 (s, 3H), 1.52 (d, 3H).

^{13}C NMR (100 MHz, $CDCl_3$) δ: 175.2, 157.6, 136.9, 133.3, 129.2, 129.1, 127.2, 126.7, 125.9, 121.6, 118.7, 105.5, 61.3, 55.5, 41.9, 32.4, 19.6.

HRMS (ESI), *m/z*: calcd. for $C_{16}H_{19}NNaO_3^+$: 296.1257 $[M + Na]^+$; found: 296.1255.

HPLC analysis: Chiralpak IG Column, λ 254 nm, eluent: *n*-hexane / *i*-propanol 95:5. Flow 1 mL/min.

$[\alpha]_D^{25}$ = + 39 (*c* = 1, $CHCl_3$, lit.[3] + 43, *c* = 1, $CHCl_3$)

HPLC Charts for Compound 3-*sm*

Racemate

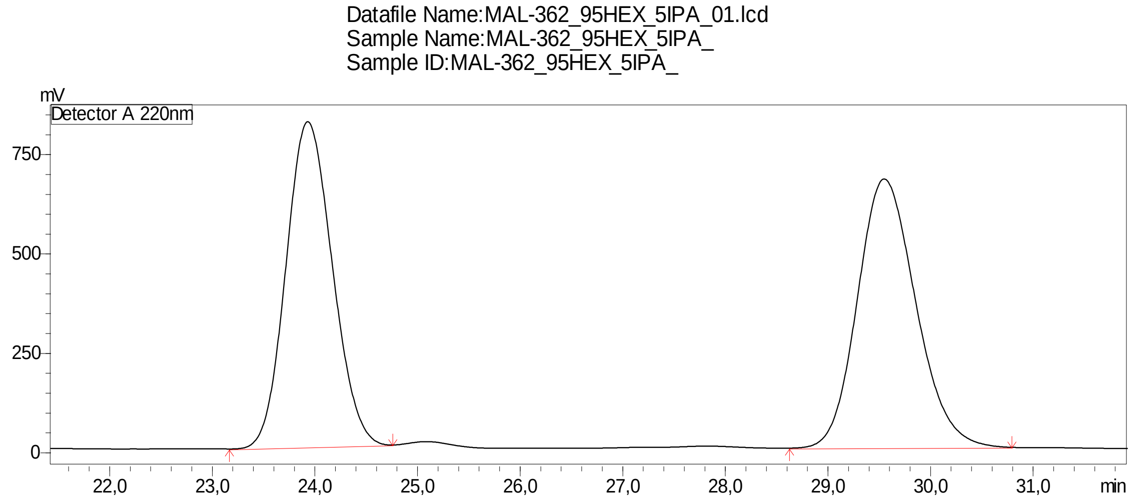

Peaks	Retention time (min)	Area	Area %
1	23,938	25704153	49,027
2	29,556	26724115	50,973
Total		52428268	100,000

Enantioenriched

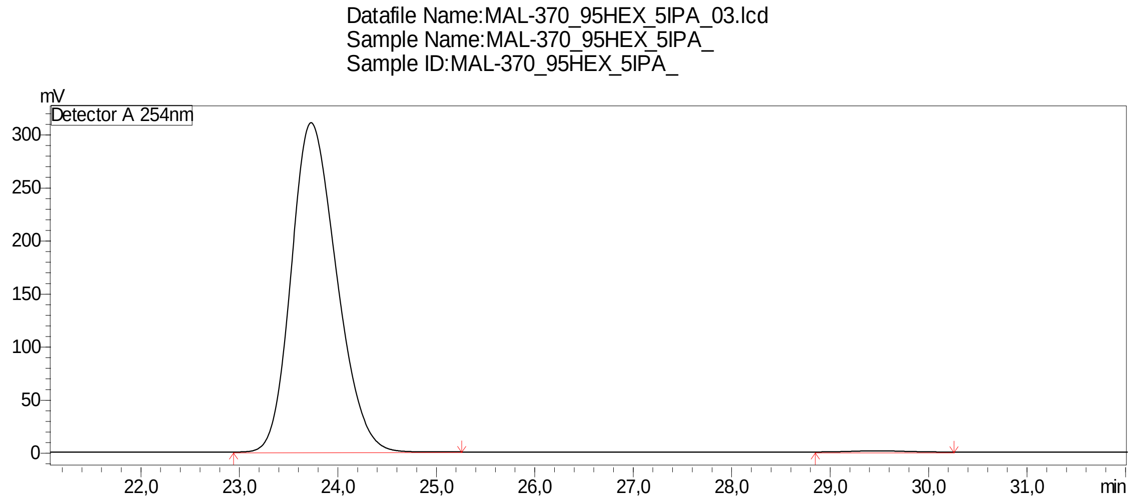

Peaks	Retention time (min)	Area	Area %
1	23,737	9903087	99,580
2	29,487	41770	0,420
Total		9944857	100,000

General procedures *for the preparation of N-alkyl-N-methoxyamides* (not previously reported)

General Procedure A for the preparation of N-alkyl-N-methoxyamides:

To a solution of *N*-alkoxyamide (1.0 equiv) and K_2CO_3 (2.0 equiv) in THF at room temperature alkyl halide (1.5 equiv) was added and the reaction was stirred overnight before quenching with $NH_4Cl_{(aq)}$. The two resulting phases were separated and the organic phase was dried over anhydrous Na_2SO_4 and evaporated under reduced pressure to give the desired compound without further purification unless otherwise stated.

General Procedure B for the preparation of N-alkoxy-N-methylamides:

To a solution of *N*-hydroxy-*N*-methylbenzamide (1.0 equiv), and Cs_2CO_3 (0.652 g, 2.0 mmol, 2.0 equiv) in MeCN (3.0 mL) at the room temperature alkyl halide (1.5 equiv) was added and the reaction was stirred overnight before quenching with $NH_4Cl_{(aq)}$. The two resulting phases were separated and the organic phase was dried over anhydrous Na_2SO_4 and evaporated under reduced pressure to give the desired compound without further purification unless otherwise stated.

_N_-ethoxy-_N_-methylbenzamide (37-_sm_)

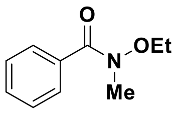

By following the general procedure A, starting from *N*-ethoxybenzamide[4] (0.165 g, 1.0 mmol, 1.0 equiv), methyl iodide (0.213 g, 1.5 mmol, 1.5 equiv) and K_2CO_3 (0.276 g, 2.0 mmol, 2.0 equiv) in THF (3.0 mL) **37-_sm_** was obtained in 98% yield (0.175 g) as a yellow oil.

^{1}H NMR (400 MHz, $CDCl_3$) δ: 7.66-7.68 (m, 2H, Ph), 7.36-7.44 (m, 3H, Ph), 4.80 (q, 2H, $C\underline{H}_2CH_3$), 3.36 (s, 3H, CH_3), 1.04 (t, 3H, $CH_2C\underline{H}_3$).

^{13}C NMR (100 MHz, $CDCl_3$) δ: 169.9, 134.2, 130.4, 128.2, 127.8, 69.4, 34.6, 13.2.

HRMS (ESI), *m/z*: calcd. for $C_{10}H_{14}NO_2^+$: 180.1025 $[M + H]^+$; found: 180.1031.

_N_-ethoxy-_N_-methyl-4-methylbenzamide (38-_sm_)

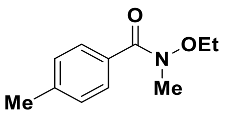

By following the general procedure A, starting from *N*-ethoxy-(4-methyl)benzamide[4] (0.179 g, 1.0 mmol, 1.0 equiv), methyl iodide (0.213 g, 1.5 mmol, 1.5 equiv) and K_2CO_3 (0.276 g, 2.0 mmol, 2.0 equiv) in THF (3.0 mL) **38-_sm_** was obtained in 97% yield (0.187 g) as a yellow oil.

^{1}H NMR (200 MHz, $CDCl_3$) δ: 7.61 (m, 2H, Ph), 7.18 (m, 2H, Ph), 3.77 (q, 2H, CH_2), 3.36 (s, 3H, CH_3), 2.37 (s, 3H, CH_3), 1.07 (t, 3H, CH_3).

^{13}C NMR (50 MHz, $CDCl_3$) δ: 169.8, 140.7, 131.2, 128.5, 69.3, 34.7, 21.4, 13.3.

HRMS (ESI), *m/z*: calcd. for $C_{11}H_{16}NO_2^+$: 194.1181 $[M + H]^+$; found: 194.1175.

_N_-ethoxy-_N_-methyl-3-chlorobenzamide (39-_sm_)

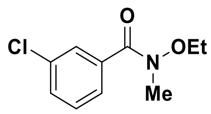

By following the general procedure A, starting from *N*-ethoxy-3-chlorobenzamide[5] (0.200 g, 1.0 mmol, 1.0 equiv), methyl iodide (0.213 g, 1.5 mmol, 1.5 equiv) and K_2CO_3 (0.276 g, 2.0 mmol, 2.0 equiv) in THF (3.0 mL) **39-_sm_** was obtained in 96% yield (0.187 g) as a yellow oil.

^{1}H NMR (200 MHz, $CDCl_3$) δ: 7.69 (m, 1H, Ph), 7.59 (m, 1H, Ph), 7.36 (m, 2H, Ph), 3.76 (q, 2H, CH_2), 3.37 (s, 3H, CH_3), 1.06 (t, 3H, CH_3).

^{13}C NMR (50 MHz, $CDCl_3$) δ: 168.2, 135.8, 133.8, 130.5, 129.2, 128.5, 126.5, 69.7, 34.4, 13.2.

HRMS (ESI), *m/z*: calcd. for $C_{10}H_{13}ClNO_2^+$: 214.0635 $[M + H]^+$; found: 214.0639.

N-isopropoxy-N-methylbenzamide (40-*sm*)

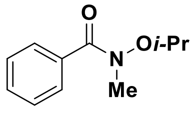

By following the general procedure B, starting from *N*-hydroxy-*N*-methylbenzamide[6] (0.151 g, 1.0 mmol, 1.0 equiv), isopropyl iodide (0.255 g, 1.5 mmol, 1.5 equiv) and Cs_2CO_3 (0.652 g, 2.0 mmol, 2.0 equiv) in THF (3.0 mL) **40-*sm*** was obtained in 75% yield (0.187 g) as an orange oil after purification by column chromatography on silica gel (Hex:EtOAc 8:2).

^{1}H NMR (200 MHz, $CDCl_3$) δ: 7.65-7.69 (m, 2H, Ph), 7.32-7.49 (m, 3H, Ph), 3.99 (sept, 1H, C$\underline{H}$(CH_3)$_2$), 3.38 (s, 3H, CH_3), 1,05 (d, 6H, CH(C$\underline{H}_3$)$_2$).

^{13}C NMR (50 MHz, $CDCl_3$) δ: 168.2, 135.8, 133.8, 130.5, 129.2, 128.5, 126.5, 69.7, 34.4, 13.2.

HRMS (ESI), *m/z*: calcd. for $C_{11}H_{16}NO_2^+$: 194.1181 [M + H]$^+$; found: 194.1174.

N-*tert*-butoxy-N-methylbenzamide (41-*sm*)

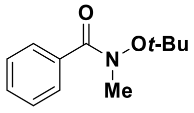

By following the general procedure A, starting from *N*-(*tert*-butoxy)benzamide[7] (0.193 g, 1.0 mmol, 1.0 equiv), methyl iodide (0.213 g, 1.5 mmol, 1.5 equiv) and K_2CO_3 (0.276 g, 2.0 mmol, 2.0 equiv) in THF (3.0 mL) **41-*sm*** was obtained in 97% yield (0.175 g) as a yellow oil.

^{1}H NMR (400 MHz, $CDCl_3$) δ: 7.67 (m, 2H), 7.39 (m, 3H), 3.39 (s, 3H), 1.14 (s, 9H).

^{13}C NMR (100 MHz, $CDCl_3$) δ: 172.8, 135.4, 130.3, 128.8, 127.8, 83.4, 27.3.

HRMS (ESI), *m/z*: calcd. for $C_{12}H_{18}NO_2^+$: 208.1338 [M + H]$^+$; found: 208.1347.

N-allyloxy-N-methylbenzamide (42-*sm*)

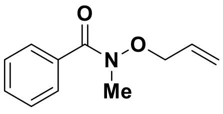

By following the general procedure A, starting from *N*-allyloxybenzamide[5] (0.177 g, 1.0 mmol, 1.0 equiv), methyl iodide (0.213 g, 1.5 mmol, 1.5 equiv) and K_2CO_3 (0.276 g, 2.0 mmol, 2.0 equiv) in THF (3.0 mL) **42-*sm*** was obtained in 98% yield (0.187 g) as a colorless oil.

^{1}H NMR (200 MHz, $CDCl_3$) δ: 9.55 (brs, 1H, NH), 7.73 (m, 2H, Ph), 7.33-7.48 (m, 3H, Ph), 5.72 (m, 1H), 5.21 (m, 2H), 4.22 (m, 2H), 3.38 (s, 3H).

^{13}C NMR (50 MHz, $CDCl_3$) δ: 170.0, 134.2, 131.4, 130.5, 128.3, 127.9, 120.7, 75.0, 35.3.

HRMS (ESI), *m/z*: calcd. for $C_{11}H_{14}NO_2^+$: 192.1025 [M + H]$^+$; found: 192.1032.

_N_-methyl-_N_-(prop-2-yn-1-yloxy)benzamide (43-_sm_)

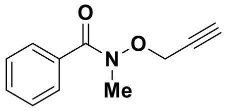

By following the general procedure B, starting from *N*-hydroxy-*N*-methylbenzamide[6] (0.151 g, 1.0 mmol, 1.0 equiv), propargyl bromide (0.178 g, 1.5 mmol, 1.5 equiv) and Cs_2CO_3 (0.652 g, 2.0 mmol, 2.0 equiv) in THF (3.0 mL) **43-*sm*** was obtained in 96% yield (0.181 g) as an orange oil with no need of purification.

^{1}H NMR (200 MHz, $CDCl_3$) δ: 7.64-7.68 (m, 2H, Ph), 7.36-7.49 (m, 3H, Ph), 4.40 (d, 2H, CH_2), 3.45 (s, 3H, CH_3), 2.49 (t, 1H, CH).

^{13}C NMR (50 MHz, $CDCl_3$) δ: 134.0, 130.7, 128.3, 128.1, 76.6, 61.6, 36.8.

HRMS (ESI), *m/z*: calcd. for $C_{11}H_{12}NO_2^+$: 190.0868 $[M + H]^+$; found: 190.0873.

_N_-(cyclopropylmethoxy)-_N_-methylbenzamide (44-_sm_)

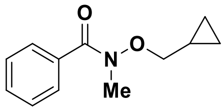

By following the general procedure B, starting from *N*-hydroxy-*N*-methylbenzamide[6] (0.151 g, 1.0 mmol, 1.0 equiv), cyclopropylmethyl bromide (0.203 g, 1.5 mmol, 1.5 equiv) and Cs_2CO_3 (0.652 g, 2.0 mmol, 2.0 equiv) in THF (3.0 mL) **44-*sm*** was obtained in 95% yield (0.195 g) as a colorless oil with no need of purification.

^{1}H NMR (200 MHz, $CDCl_3$) δ: 7.67-7.71 (m, 2H, Ph), 7.35-7.45 (m, 3H, Ph), 3.50 (d, 2H, CH_2), 3.39 (s, 3H, CH_3), 0.85 (m, 1H, CH), 0.46 (m, 2H), 0.03 (m, 2H).

^{13}C NMR (50 MHz, $CDCl_3$) δ: 168.2, 135.8, 133.8, 130.5, 129.2, 128.5, 126.5, 69.7, 34.4, 13.2.

HRMS (ESI), *m/z*: calcd. for $C_{12}H_{16}NO_2^+$: 206.1181 $[M + H]^+$; found: 206.1173.

_N_-(methoxymethoxy)-_N_-methylbenzamide (45-_sm_)

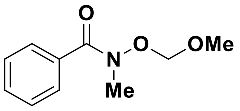

By following the general procedure B, starting from *N*-hydroxy-*N*-methylbenzamide[6] (0.151 g, 1.0 mmol, 1.0 equiv), bromomethyl methyl ether (0.188 g, 1.5 mmol, 1.5 equiv) and Cs_2CO_3 (0.652 g, 2.0 mmol, 2.0 equiv) in THF (3.0 mL) **45-*sm*** was obtained in 85% yield (0.166 g) as a colorless oil after purification by column chromatography on silica gel (Hex:EtOAc 7:3).

^{1}H NMR (200 MHz, $CDCl_3$) δ: 7.61 (m, 2H, Ph), 7.42 (m, 3H, Ph), 4.92 (s, 2H, CH_2), 3.44(s, 3H, CH_3), 3.27 (s, 3H, CH_3).

^{13}C NMR (50 MHz, $CDCl_3$) δ: 171.0, 133.7, 131.0, 128.3, 128.0, 108.2, 103.7, 39.5

HRMS (ESI), *m/z*: calcd. for $C_{10}H_{14}NO_3^+$: 196.0968 $[M + H]^+$; found: 196.0954.

***N*-phenoxy-*N*-methylbenzamide (46-*sm*)**

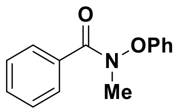

By following the general procedure A, starting from *N*-phenoxybenzamide[8] (0.213 g, 1.0 mmol, 1.0 equiv), methyl iodide (0.213 g, 1.5 mmol, 1.5 equiv) and K_2CO_3 (0.276 g, 2.0 mmol, 2.0 equiv) in THF (3.0 mL) **46-*sm*** was obtained in 96% yield (0.218 g) as a yellow oil.

^{1}H NMR (200 MHz, $CDCl_3$) δ: 7.62 (m, 2H, Ph), 7.26-7.45 (m, 5H, Ph), 6.97-7.11 (m, 3H, Ph), 3.44 (s, 3H, CH_3).

^{13}C NMR (50 MHz, $CDCl_3$) δ: 157.4, 133.3, 131.1, 129.9, 128.1, 128.0, 123.2, 113.3, 35.7.

HRMS (ESI), *m/z*: calcd. for $C_{14}H_{14}NO_2^+$: 228.1019 $[M + H]^+$; found: 228.1023.

***N*-benzyloxy-*N*-methylbenzamide (47-*sm*)[9]**

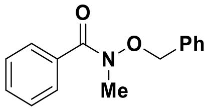

To a solution of benzoyl chloride (1.0 equiv, 3.6 mmol, 0.500 g) in Et_2O was added *O*-benzylhydroxylamine hydrochloride (1.1 equiv, 3.9 mmol, 0.624 g) and the resulting suspension was cooled to 0°C. Afterwards, an aqueous solution of potassium carbonate (2.2 equiv, 7.8 mmol, 1.082 g) was added during 2 min. The resulting mixture was stirred at room temperature overnight and subsequently H_2O was added. The two resulting phases were separated and, the organic phase was dried over anhydrous Na_2SO_4, filtered and removed in vacuo to give *N*-benzyloxybenzamide as an oil in 98% yield with no need of purification. **^{1}H NMR** (200 MHz, $CDCl_3$) δ: 8.51 (NH), 7.66 (m, 2H, Ph), 7.37-7.55 (m, 3H, Ph), 5,04 (s, 2H, CH_2). **^{13}C NMR** (50 MHz, $CDCl_3$) δ: 135.2, 132.1, 131.9, 129.3, 128.8, 128.7, 128.7, 127.0, 78.4.

By following the general procedure A, the above prepared *N*-benzyloxybenzamide (0.227 g, 1.0 mmol, 1.0 equiv) was reacted with methyl iodide (0.213 g, 1.5 mmol, 1.5 equiv) and K_2CO_3 (0.276 g, 2.0 mmol, 2.0 equiv) in THF (3.0 mL), furnishing **47-*sm*** in 98% yield (0.236 g) as a yellow oil.

^{1}H NMR (400 MHz, $CDCl_3$) δ: 7.53 (m, 2H, Ph), 7.37 (m, 1H, Ph), 7.31 (m, 2H, Ph), 7.21 (m, 3H, Ph), 7.00 (m, 2H, Ph), 4.60 (s, 2H, CH_2), 3.28 (s, 3H, CH_3).

^{13}C NMR (100 MHz, $CDCl_3$) δ: 170.1, 134.3, 134.1, 130.4, 129.4, 128.8, 128.4, 128.2, 127.9.

HRMS (ESI), *m/z*: calcd. for $C_{15}H_{16}NO_2^+$: 242.1181 $[M + H]^+$; found: 242.1185.

***N*-([1,1'-biphenyl]-4-ylmethoxy)-*N*-methylbenzamide (48-*sm*)**

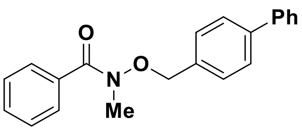

By following the general procedure B, starting from *N*-hydroxy-*N*-methylbenzamide[6] (0.151 g, 1.0 mmol, 1.0 equiv), 4-(bromomethyl)-1,1'-biphenyl (0.371 g, 1.5 mmol, 1.5 equiv) and Cs_2CO_3 (0.652 g, 2.0 mmol, 2.0 equiv) in THF (3.0 mL), **48-*sm*** was obtained in 90% yield (0.285 g) as a colorless oil after purification by column chromatography on silica gel (Hex:EtOAc 7:3).

^{1}H NMR (200 MHz, $CDCl_3$) δ: 7.32-7.66 (m, 12 H, Ph), 7.12-7.16 (m, 2H, Ph), 4.72 (s, 2H, CH_2), 3.41 (s, 3H, CH_3).

^{13}C NMR (50 MHz, $CDCl_3$) δ: 170.2, 14.7, 140.4, 134.3, 133.0, 130.4, 129.9, 128.8, 128.3, 127.9, 127.5, 127.2, 127.1, 75.8, 35.0.

HRMS (ESI), *m/z*: calcd. for $C_{21}H_{20}NO_2^+$: 318.1489 $[M + H]^+$; found: 318.1485.

General procedure for the synthesis of N-(alkoxymethyl)amides

To a solution of the starting *N*-alkoxy-*N*-methylamide (1.0 equiv) in dry 2-MeTHF (3 mL) PMDTA (1.6 equiv) was added. Then, *s*-BuLi (1.4 M in cyclohexane, 1.6 equiv) was added dropwise at -78 °C. After stirring for 2 hours at -78 °C, the reaction was quenched with sat. aqueous NH_4Cl. The reaction mixture was allowed to reach room temperature and was exhaustively extracted with Et_2O (3 x 10 ml). The combined organic extracts were dried over Na_2SO_4 and the solvent was removed under reduced pressure. The final products were obtained after purification by column chromatography on silica gel.

N-(alkoxymethyl)amides: Experimental procedures and characterization

2-(4-isobutylphenyl)-*N*-(methoxymethyl)propanamide (2)

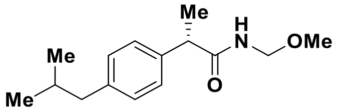

By following the general procedure, starting from *S*-(+)-2-(4-isobutylphenyl)-*N*-methoxy-*N*-methylpropanamide **1** (0.249 g, 1.0 mmol, 1.0 equiv), PMDTA (0.277 g, 0.33 mL, 1.6 mmol, 1.6 equiv) and *s*-BuLi (2.2 M in Et_2O, 0.73 mL, 1.6 mmol, 1.6 equiv) in dry 2-MeTHF (3 mL), compound **2** was obtained in 81% yield (0.201 g – *entry 6, Table 1 manuscript*) as a colourless oil after purification by column chromatography on silica gel (*n*-hexane:EtOAc 7:3).

The corresponding racemic sample has been prepared starting from racemic 2-(4-isobutylphenyl)-*N*-methoxy-*N*-methylpropanamide and spectroscopic data match with those ones reported below.

^{1}H NMR (400 MHz, Acetone-d_6) δ: 7.73 (brs, 1H, NH), 7.27 (m, 2H, Ph H-2,6), 7.10 (m, 2H, Ph H-3,5), 4.55 (d of an AB system, $^2J_{AB}$ = 10.2 Hz, $^3J_{H,NH}$ = 6.8 Hz, 1H, CH_2), 4.53 (d of an AB system, $^2J_{AB}$ = 10.2 Hz, $^3J_{H,NH}$ = 6.8 Hz, 1H, CH_2), 3.64 (q, ^3J = 7.1 Hz, 1H, $C\underline{H}CH_3$), 3.12 (s, 3H, OCH_3), 2.44 (d, J = 7.1 Hz, 2H, $C\underline{H}_2CH(CH_3)_2$), 1.84 (m, 1H, $CH_2C\underline{H}(CH_3)_2$), 1.40 (d, ^3J = 7.1 Hz, 3H, $CHC\underline{H}_3$), 0.88 (d, J = 6.6 Hz, 6H, $CH_2CH(C\underline{H}_3)_2$).

^{13}C NMR (100 MHz, Acetone-d_6) δ: 175.3 (C=O), 140.8 (Ph C-4), 140.5 (Ph C-1), 129.9 (Ph C-3,5), 128.1 (Ph C-2,6), 71.7 (CH_2), 55.4 (OCH_3), 47.0 ($\underline{C}HCH_3$), 45.6 ($\underline{C}H_2CH(CH_3)_2$), 31.0 ($CH_2\underline{C}H(CH_3)_2$), 22.60 ($CH_2CH(\underline{C}H_3)_2$), 22.59 ($CH_2CH(\underline{C}H_3)_2$), 19.2 ($CH\underline{C}H_3$).

^{15}N NMR (40 MHz, Acetone-d_6) δ: -260.6 (NH).

HRMS (ESI), *m/z*: calcd. for $C_{15}H_{23}NNaO_2^+$: 272.1621 $[M + Na]^+$; found: 272.1630.

HPLC analysis: Chiralpak IG Column, λ 254 nm, eluent: *n*-hexane / *i*-propanol 80:20. Flow 1 mL/min.

$[\alpha]_D^{25}$ = + 13 (c = 1, $CHCl_3$).

Racemate

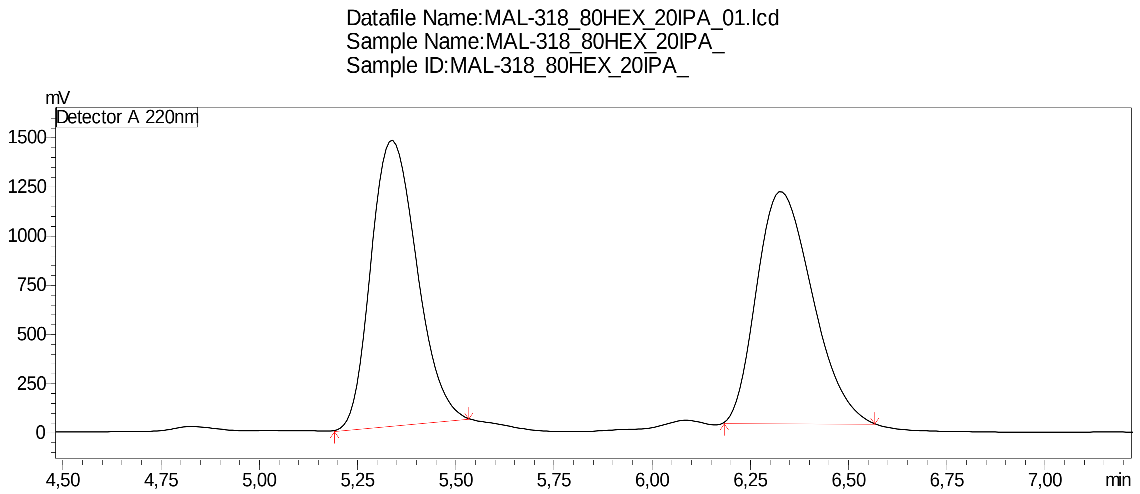

Peaks	Retention time (min)	Area	Area %
1	5,339	11766643	50,340
2	6,329	11607588	49,660
Total		23374232	100,000

Enantioenriched

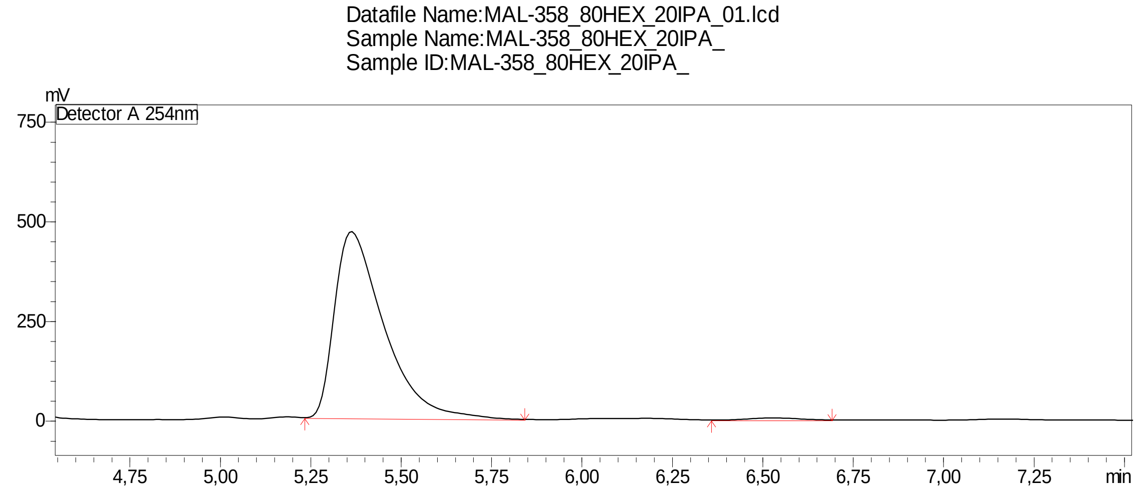

Peaks	Retention time (min)	Area	Area %
1	5,364	4458612	98,980
2	6,531	45931	1,020
Total		4504543	100,000

2-(4-isobutylphenyl)-N-methylpropanamide (2a)

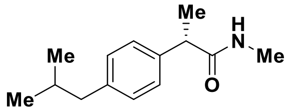

By following the general procedure, starting from *S*-(+)-2-(4-isobutylphenyl)-*N*-methoxy-*N*-methylpropanamide **1** (0.249 g, 1.0 mmol, 1.0 equiv), TMEDA (0.186 g, 0.24 mL, 1.6 mmol, 1.6 equiv) and *s*-BuLi (2.2 M in Et_2O, 0.73 mL, 1.6 mmol, 1.6 equiv) in dry 2-MeTHF (3 mL), compound **2a[10]** was obtained in 11% yield (0.024 g – *entry 5, Table 1 manuscript*) as a white solid after purification by column chromatography on silica gel (*n*-hexane:EtOAc 7:3).

The corresponding racemic sample has been prepared starting from racemic *N*-methoxy-2-(6-methoxynaphthalen-2-yl)-*N*-methylpropanamide and spectroscopic data match with those ones reported below.

mp = 106 °C (lit.[10] 104-106 °C)

^{1}H NMR (200 MHz, $CDCl_3$) δ: 7.19 (m, 2H), 7.11 (m, 2H), 5.39 (brs, 1H), 3.53 (q, 1H), 2.73 (d, 3H), 2.45 (d, 2H), 1.85 (m, 1H), 1.51 (d, 3H), 0.90 (d, 6H).

^{13}C NMR (100 MHz, $CDCl_3$) δ: 175.1, 140.7, 138.5, 129.7, 129.6, 127.4, 46.7, 45.0, 30.1, 26.5, 22.4, 18.5.

HRMS (ESI), *m/z*: calcd. for $C_{14}H_{21}NNaO^+$: 242.1521 $[M + Na]^+$; found: 242.1526.

$[\alpha]_D^{25}$ = + 40 (*c* = 1, EtOH).

2-(4-isobutylphenyl)-4-methylhexan-3-one (2b)

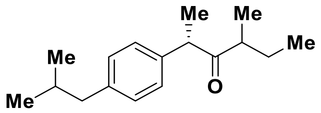

By following the general procedure, starting from *S*-(+)-2-(4-isobutylphenyl)-*N*-methoxy-*N*-methylpropanamide **1** (0.249 g, 1.0 mmol, 1.0 equiv), TMEDA (0.186 g, 0.24 mL, 1.6 mmol, 1.6 equiv) and *s*-BuLi (2.2 M in Et_2O, 0.73 mL, 1.6 mmol, 1.6 equiv) in dry 2-MeTHF (3 mL), compound **2b** was obtained in 8% yield (0.020 g – *entry 5, Table 1 manuscript*) as a colourless oil after purification by column chromatography on silica gel (*n*-hexane:EtOAc 7:3).

The corresponding racemic sample has been prepared starting from racemic *N*-methoxy-2-(6-methoxynaphthalen-2-yl)-*N*-methylpropanamide and spectroscopic data match with those ones reported below.

^{1}H NMR (400 MHz, $CDCl_3$) δ: 7.10 (m, 4H), 3.83 (m, 1H), 2.55 (m, 1H), 2.44 (d, 2H), 1.83 (m, 1H), 1,59 (m, 1H), 1.37 (m, 3H), 1.25 (m, 1H), 1.03- 0.53 (m, 3H), 0.90 (d, 3H), 0.90-0.80 (m, 9H).

^{13}C NMR (50 MHz, $CDCl_3$) δ: 215.1, 214.4, 140.6, 137.8, 137.4, 129.6, 129.6, 128.0, 127.9, 52.3, 51.3, 46.3, 46.1, 45.1, 30.3, 27.0, 25.6, 22.5, 22.5, 22.4, 17.9, 17.9, 17.5, 16.3, 11.8, 11.7.
HRMS (ESI), *m/z*: calcd. for $C_{17}H_{26}NaO^+$: 269.1881 $[M + Na]^+$; found: 269.1885.

$[\alpha]_D^{25}$ = + 76 (*c* = 1, EtOH).

N-(methoxymethyl)-2-(6-methoxy-2-naphthyl)propenamide (3)

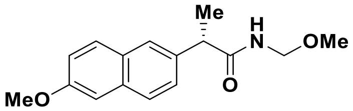

By following the general procedure, starting from (*S*)-(+)-*N*-methoxy-2-(6-methoxynaphthalen-2-yl)-*N*-methylpropanamide **1** (0.273 g, 1.0 mmol, 1.0 equiv), PMDTA (0.277 g, 0.33 mL, 1.6 mmol, 1.6 equiv) and *s*-BuLi (2.2 M in Et_2O, 0.73 mL, 1.6 mmol, 1.6 equiv) in dry 2-MeTHF (3 mL), compound **3** was obtained in 70% yield (0.191 g) as a white solid after purification by column chromatography on silica gel (*n*-hexane:EtOAc 7:3).

The corresponding racemic sample has been prepared starting from racemic *N*-methoxy-2-(6-methoxynaphthalen-2-yl)-*N*-methylpropanamide and spectroscopic data match with those ones reported below.

mp = 70-75 °C (enantiomer); 83-86 °C (racemate)

^{1}H NMR (400 MHz, Acetone-d_6) δ: 7.78 (m, 1H, NH), 7.75 (m, 3H, Naph H-1,4,8), 7.47 (dd, ^3J = 8.5 Hz, ^4J = 1.8 Hz, 1H, Naph H-3), 7.26 (d, ^4J = 2.6 Hz, 1H, Naph H-5), 7.13 (dd, ^3J = 9.0 Hz, ^4J = 2.6 Hz, 1H, Naph H-7), 4.56 (d of an AB-system, $^2J_{AB}$ = 12.8 Hz, ^3J = 6.8 Hz, 1 H, CH_2), 4.55 (d of an AB-system, $^2J_{AB}$ = 12.8 Hz, ^3J = 6.8 Hz, 1 H, CH_2), 3.90 (s, 3H, OCH_3), 3.81 (q, ^3J = 7.1 Hz, 1H, CH), 3.12 (s, 3H, $CH_2OC\underline{H}_3$), 1.49 (d, ^3J = 7.1 Hz, 3H, $CHC\underline{H}_3$).

^{13}C NMR (100 MHz, Acetone-d_6) δ: 175.2 (C=O), 158.6 (Naph C-6), 138.3 (Naph C-2), 134.7 (Naph C-4a), 130.0 (Naph C-8), 129.9 (Naph C-8a), 127.9 (Naph C-4), 127.2 (Naph C-3), 126.8 (Naph C-1), 106.5 (Naph C-5), 71.8 (CH_2), 55.6 (OCH_3) , 55.5 ($CH_2O\underline{C}H_3$), 47.3 (CH), 19.2 ($CH\underline{C}H_3$).

^{15}N NMR (40 MHz, Acetone-d_6) δ: -260.3 (NH).

HRMS (ESI), *m/z*: calcd. for $C_{16}H_{19}NNaO_3^+$: 296.1257 $[M + Na]^+$; found: 296.1259.

HPLC analysis: Chiralpak IG Column, λ 254 nm, eluent: *n*-hexane / *i*-propanol 80:20. Flow 1 mL/min.

$[\alpha]_D^{25}$ = + 21 (*c* = 1, $CHCl_3$).

Racemate

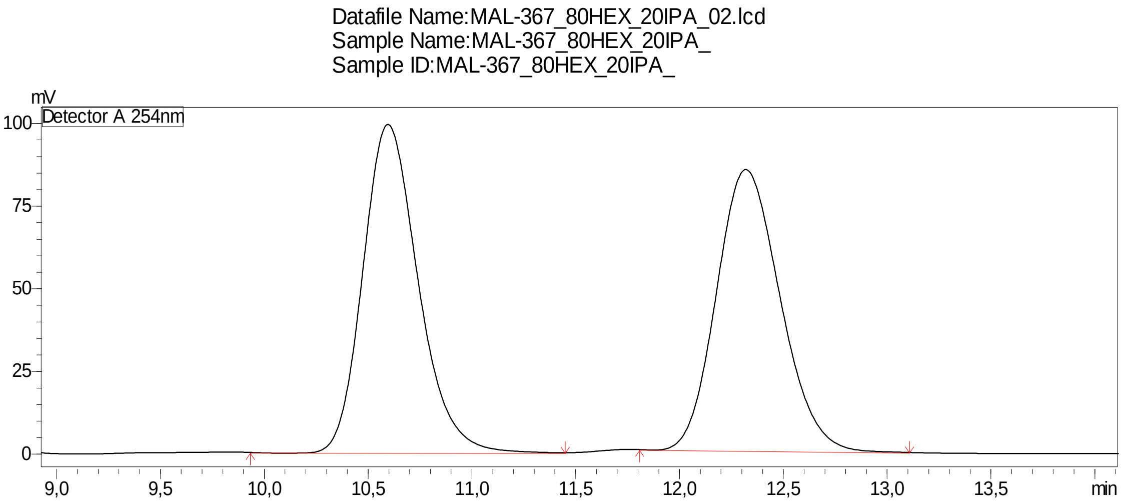

Peaks	Retention time (min)	Area	Area %
1	10,599	1827233	50,212
2	12,323	1811835	49,788
Total		3639068	100,000

Enantioenriched

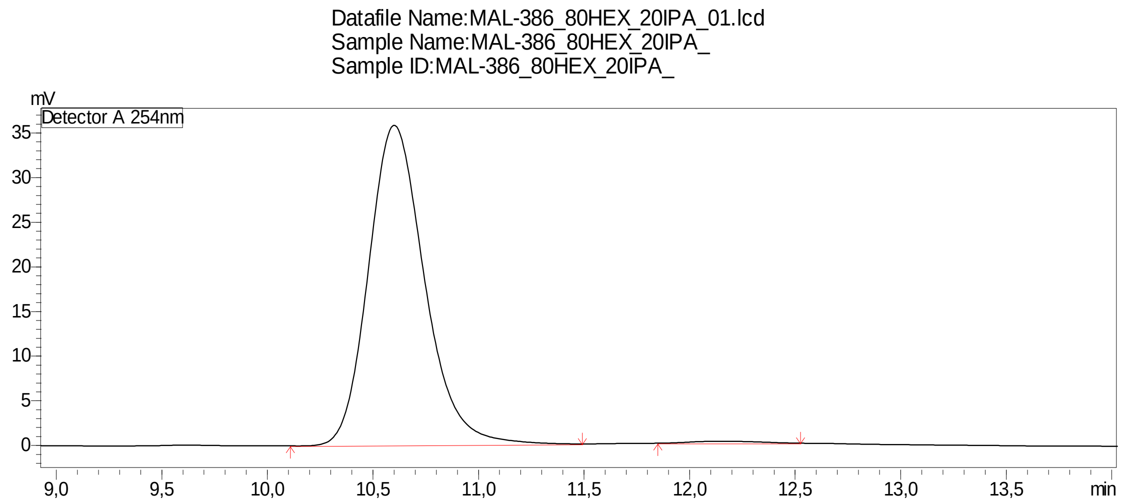

Peaks	Retention time (min)	Area	Area %
1	10,605	660713	99,255
2	12,144	4957	0,745
Total		665670	100,000

***N*-(methoxymethyl)-2-phenylpropanamide (4)**

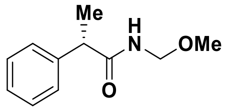

By following the general procedure, starting from (S)-(+)-*N*-methoxy-*N*-methyl-2-phenylpropanamide (0.193 g, 1.0 mmol, 1.0 equiv), PMDTA (0.277 g, 0.33 mL, 1.6 mmol, 1.6 equiv) and *s*-BuLi (2.2 M in Et_2O, 0.73 mL, 1.6 mmol, 1.6 equiv) in dry 2-MeTHF (3 mL), compound **4** was obtained in 73% yield (0.164 g) as a colourless oil after purification by column chromatography on silica gel (*n*-hexane:EtOAc 6:4).

The corresponding racemic sample has been prepared starting from racemic *N*-methoxy-*N*-methyl-2-phenylpropanamide and spectroscopic data match with those ones reported below.

^{1}H NMR (400 MHz, C_6D_6) δ: 7.18 (m, 2H, Ph H-2,6), 7.11 (m, 2H, Ph H-3,5), 7.03 (m, 1H, Ph H-4), 5.63 (brs, 1H, NH), 4.38 (d, J = 6.7 Hz, 2H, CH_2), 3.15 (q, ^3J = 7.1 Hz, 1H, CH), 3.05 (s, 3H, OCH_3), 1.44 (d, ^3J = 7.1 Hz, 3H, CH_3).

^{13}C NMR (100 MHz, C_6D_6) δ: 174.2 (C=O), 142.1 (Ph C-1), 129.0 (Ph C-3,5), 127.9 (Ph C-2,6), 127.3 (Ph C-4), 71.4 (CH_2), 55.5 (OCH_3), 47.4 (CH), 19.02 (CH_3).

^{15}N NMR (40 MHz, C_6D_6) δ: -260.8 (NH).

HRMS (ESI), *m/z*: calcd. for $C_{11}H_{15}NNaO_2^+$: 216.0995 $[M + Na]^+$; found: 216.1005.

HPLC analysis: Chiralpak IG Column, λ 254 nm, eluent: *n*-hexane / *i*-propanol 80:20. Flow 1 mL/min.

$[\alpha]_D^{25}$ = + 49 (*c* = 1, $CHCl_3$).

Racemate

Datafile Name:MAL-320_80HEX_20IPA_01.lcd
Sample Name:MAL-320_80HEX_20IPA_
Sample ID:MAL-320_80HEX_20IPA_

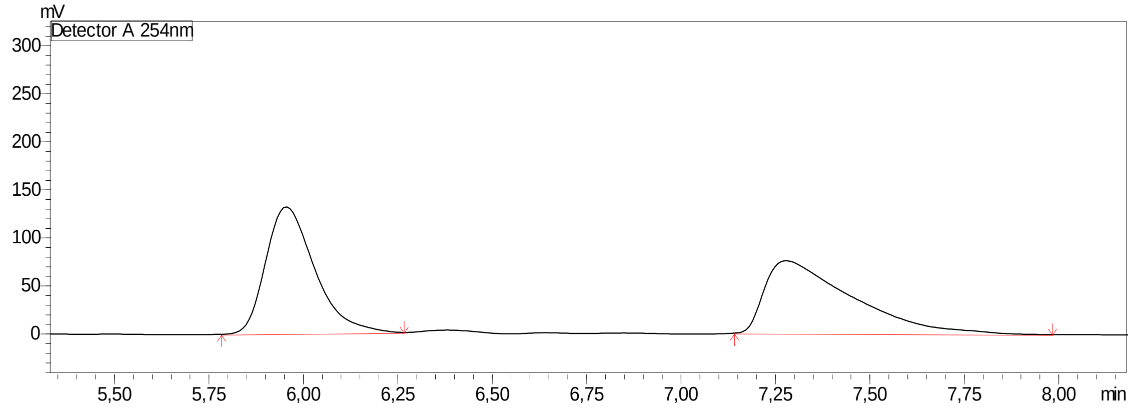

Peaks	Retention time (min)	Area	Area %
1	5,957	1189450	49,555
2	7,280	1210834	50,445
Total		2400285	100,000

Enantioenriched

Datafile Name:MAL-336_80HEX_20IPA_02.lcd
Sample Name:MAL-336_80HEX_20IPA_
Sample ID:MAL-336_80HEX_20IPA_

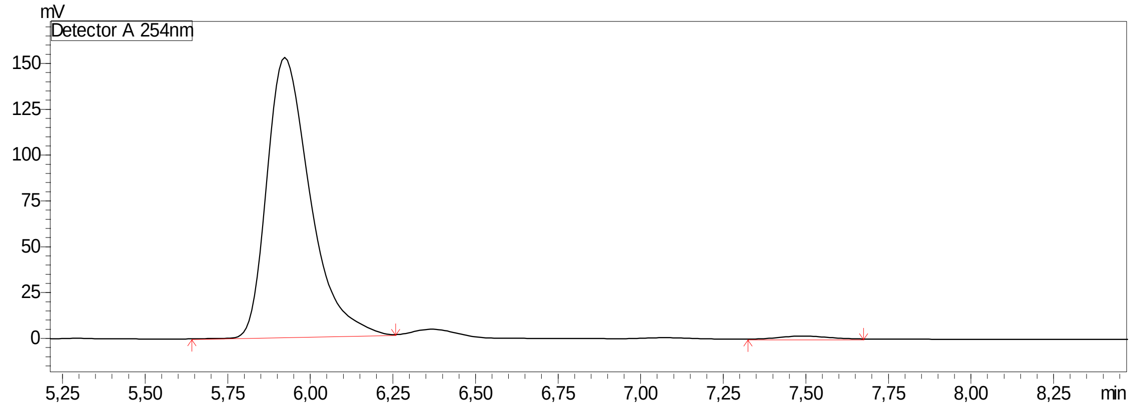

Peaks	Retention time (min)	Area	Area %
1	5,925	1383247	98,862
2	7,492	15916	1,138
Total		1399163	100,000

***N*-(methoxymethyl)-3-phenylpropanamide (5)**

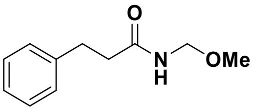

By following the general procedure, starting from *N*-methoxy-*N*-methyl-3-phenylpropanamide (0.193 g, 1.0 mmol, 1.0 equiv), PMDTA (0.277 g, 0.33 mL, 1.6 mmol, 1.6 equiv) and *s*-BuLi (2.2 M in Et_2O, 0.73 mL, 1.6 mmol, 1.6 equiv) in dry 2-MeTHF (3 mL), compound **5** was obtained in 62% yield (0.120 g) as a colourless oil after purification by column chromatography on silica gel (*n*-hexane:EtOAc 5:5).

^{1}H NMR (400 MHz, Acetone-d_6) δ: 7.74 (brs, 1H, NH), 7.26 (m, 2H, Ph H-3,5), 7.24 (m, 2H, Ph H-2,6), 7.16 (m, 1H, Ph H-4), 4.56 (d, *J* = 6.8 Hz, 2H, $C\underline{H}_2OCH_3$), 3.16 (s, 3H, OCH_3), 2.92 (m, 2H, $C\underline{H}_2CH_2CO$), 2.52 (m, 2H, $CH_2C\underline{H}_2CO$).

^{13}C NMR (100 MHz, Acetone-d_6) δ: 173.0 (C=O), 142.4 (Ph C-1), 129.2 (Ph C-2,6), 129.15 (Ph C-3,5), 126.8 (Ph C-4), 71.6 ($\underline{C}H_2OCH_3$), 55.5 (OCH_3), 38.5 ($CH_2\underline{C}H_2CO$), 32.0 ($\underline{C}H_2CH_2CO$).

^{15}N NMR (40 MHz, Acetone-d_6) δ: -259.3 (NH).

HRMS (ESI), *m/z*: calcd. for $C_{11}H_{15}NNaO_2^+$: 216.0995 $[M + Na]^+$; found: 216.0999.

***N*-(methoxymethyl)-2,2-dimethylpropanamide (6)**

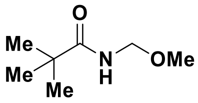

By following the general procedure, starting from *N*-methoxy-*N*-methylpivalamide (0.145 g, 1.0 mmol, 1.0 equiv), PMDTA (0.277 g, 0.33 mL, 1.6 mmol, 1.6 equiv) and *s*-BuLi (2.2 M in Et_2O, 0.73 mL, 1.6 mmol, 1.6 equiv) in dry 2-MeTHF (3 mL), compound **6** was obtained in 79% yield (0.114 g) as a colourless oil after purification by column chromatography on silica gel (*n*-hexane:EtOAc 6:4).

^{1}H NMR (400 MHz, Acetone-d_6) δ: 7.57 (brs, 1H, NH), 4.57 (d, *J* = 6.7 Hz, 2H, CH_2), 3.19 (s, 3H, OCH_3), 1.18 (s, 9H, CH_3).

^{13}C NMR (100 MHz, Acetone-d_6) δ: 179.1 (C=O), 71.9 (CH_2), 55.4 (OCH_3), 39.3 ($\underline{C}(CH_3)_3$), 27.7 (CH_3).

^{15}N NMR (40 MHz, Acetone-d_6) δ: -267.5 (NH).

HRMS (ESI), *m/z*: calcd. for $C_7H_{15}NNaO_2^+$: 168.0995 $[M + Na]^+$; found: 168.0997.

***N*-(methoxymethyl)cyclopropanecarboxamide (7)**

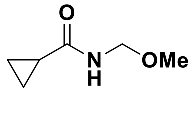

By following the general procedure, starting from *N*-methoxy-*N*-methylcyclopropanecarboxamide (0.129 g, 1.0 mmol, 1.0 equiv), PMDTA (0.277 g, 0.33 mL, 1.6 mmol, 1.6 equiv) and *s*-BuLi (2.2 M in Et_2O, 0.73 mL, 1.6 mmol, 1.6 equiv) in dry 2-MeTHF (3 mL), compound **7** was obtained in 75% yield (0.097 g) as a colourless oil after purification by column chromatography on silica gel (*n*-hexane:EtOAc 6:4).

^{1}H NMR (400 MHz, C_6D_6) δ: 6.31 (brs, 1H, NH), 4.53 (d, J = 6.9 Hz, 2H, CH_2), 3.16 (s, 3H, OCH_3), 1.07 (m, 2H, cyclopropane H-2,3), 1.00 (m, 1H, cyclopropane H-1), 0.43 (m, 2H, cyclopropane H-2,3).

^{13}C NMR (100 MHz, C_6D_6) δ: 174.2 (C=O), 71.5 (CH_2), 55.6 (OCH_3), 14.6 (cyclopropane C-1), 7.5 (cyclopropane C-2,3).

^{15}N NMR (40 MHz, C_6D_6) δ: -259.9 (NH).

HRMS (ESI), *m/z*: calcd. for $C_6H_{11}NNaO_2^+$: 152.0682 $[M + Na]^+$; found: 152.0687.

***N*-(methoxymethyl)cyclobutanecarboxamide (8)**

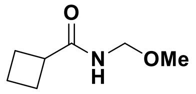

By following the general procedure, starting from *N*-methoxy-*N*-methylcyclobutanecarboxamide (0.143 g, 1.0 mmol, 1.0 equiv), PMDTA (0.277 g, 0.33 mL, 1.6 mmol, 1.6 equiv) and *s*-BuLi (2.2 M in Et_2O, 0.73 mL, 1.6 mmol, 1.6 equiv) in dry 2-MeTHF (3 mL), compound **8** was obtained in 71% yield (0.101 g) as a colourless oil after purification by column chromatography on silica gel (*n*-hexane:EtOAc 6:4).

^{1}H NMR (400 MHz, C_6D_6) δ: 5.59 (brs, 1H, NH), 4.51 (d, J = 6.9 Hz, 2H, CH_2), 3.16 (s, 3H, OCH_3), 2.57 (m, 1H, cyclobutane H-1), 2.29 (m, 2H, cyclobutane H-2,4), 1.88 (m, 2H, cyclobutane H-2,4), 1.71 (m, 2H, cyclobutane H-3).

^{13}C NMR (100 MHz, C_6D_6) δ: 175.0 (C=O), 71.3 (CH_2), 55.6 (OCH_3), 40.0 (cyclobutane C-1), 25.4 (cyclobutane C-2,4), 18.6 (cyclobutane C-3).

^{15}N NMR (40 MHz, C_6D_6) δ: -263.9 (NH).

HRMS (ESI), *m/z*: calcd. for $C_7H_{13}NNaO_2^+$: 166.0838 $[M + Na]^+$; found: 166.0847.

***N*-(methoxymethyl)cyclopentanecarboxamide (9)**

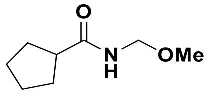

By following the general procedure, starting from *N*-methoxy-*N*-methylcyclopentanecarboxamide (0.157 g, 1.0 mmol, 1.0 equiv), PMDTA (0.277 g, 0.33 mL, 1.6 mmol, 1.6 equiv) and *s*-BuLi (2.2 M in Et_2O, 0.73 mL, 1.6 mmol, 1.6 equiv) in dry 2-MeTHF (3 mL), compound **9** was obtained in 74% yield (0.116 g) as a white solid after purification by column chromatography on silica gel (*n*-hexane:EtOAc 6:4).

mp = 35-40 °C

^{1}H NMR (400 MHz, C_6D_6) δ: 5.96 (brs, 1H, NH), 4.54 (d, *J* = 6.9 Hz, 2H, CH_2), 3.18 (s, 3H, OCH_3), 2.18 (m, 1H, cyclopentane H-1), 1.85 (m, 2H, cyclopentane H-2,5), 1.64 (m, 4H, cyclopentane H-2,3,4,5), 1.39 (m, 2H, cyclopentane C-3,4).

^{13}C NMR (100 MHz, C_6D_6) δ: 176.5 (C=O), 71.4 (CH_2), 55.6 (OCH_3), 45.8 (cyclopentane C-1), 30.6 (cyclopentane C-2,5), 26.3 (cyclopentane C-3,4).

^{15}N NMR (40 MHz, C_6D_6) δ: -261.4 (NH).

HRMS (ESI), *m/z*: calcd. for $C_8H_{15}NNaO_2^+$: 180.0995 $[M + Na]^+$; found: 180.0998.

N-(methoxymethyl)cyclohexanecarboxamide (10)

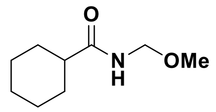

By following the general procedure, starting from *N*-methoxy-*N*-methylcyclohexanecarboxamide (0.171 g, 1.0 mmol, 1.0 equiv), PMDTA (0.277 g, 0.33 mL, 1.6 mmol, 1.6 equiv) and *s*-BuLi (2.2 M in Et_2O, 0.73 mL, 1.6 mmol, 1.6 equiv) in dry 2-MeTHF (3 mL), compound **10** was obtained in 68% yield (0.116 g) as a white solid after purification by column chromatography on silica gel (*n*-hexane:EtOAc 6:4).

mp = 69-71 °C

^{1}H NMR (400 MHz, C_6D_6) δ: 5.42 (brs, 1H, NH), 4.54 (d, *J* = 6.9 Hz, 2H, CH_2), 3.18 (s, 3H, OCH_3), 1.66 (m, 3H, cyclohexane H-1, 2,6), 1.61 (m, 2H, cyclohexane H-3,5), 1.49 (m, 2H, cyclohexane H-4), 1.47 (m, 2H, cyclohexane H-2,6), 1.06 (m, 2H, cyclohexane H-3,5).

^{13}C NMR (100 MHz, C_6D_6) δ: 175.9 (C=O), 71.2 (CH_2), 55.6 (OCH_3), 45.5 (cyclohexane C-1), 29.8 (cyclohexane C-2,6), 26.1 (cyclohexane C-4), 26.0 (cyclohexane C-3,5).

^{15}N NMR (40 MHz, C_6D_6) δ: -263.5 (NH).

HRMS (ESI), *m/z*: calcd. for $C_9H_{17}NNaO_2^+$: 194.1151 $[M + Na]^+$; found: 194.1152.

N-(methoxymethyl)-1-adamantanecarboxamide (11)

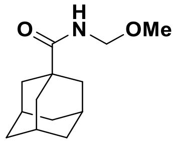

By following the general procedure, starting from *N*-methoxy-*N*-methyladamantane-1-carboxamide (0.223 g, 1.0 mmol, 1.0 equiv), PMDTA (0.277 g, 0.33 mL, 1.6 mmol, 1.6 equiv) and *s*-BuLi (2.2 M in Et_2O, 0.73 mL,

1.6 mmol, 1.6 equiv) in dry 2-MeTHF (3 mL), compound **11** was obtained in 70% yield (0.156 g) as a white solid after purification by column chromatography on silica gel (*n*-hexane:EtOAc 7:3).

mp = 110 °C

^{1}H NMR (400 MHz, C_6D_6) δ: 5.60 (brs, 1H, NH), 4.62 (d, *J* = 6.8 Hz, 2H, CH_2), 3.21 (s, 3H, OCH_3), 1.81 (m, 3H, Adamantane H-3,5,7), 1.70 (m, 6H, Adamantane H-2,8,9), 1.53 (m, 6H, Adamantane H-4,6,10).

^{13}C NMR (100 MHz, C_6D_6) δ: 177.5 (C=O), 71.3 (CH_2), 55.6 (OCH_3), 40.9 (Adamantane C-1), 39.3 (Adamantane C-2,8,9), 36.7 (Adamantane C-4,6,10), 28.5 (Adamantane C-3,5,7).

^{15}N NMR (40 MHz, C_6D_6) δ: -269.4 (NH).

HRMS (ESI), *m/z*: calcd. for $C_{13}H_{21}NNaO_2^+$: 246.1464 $[M + Na]^+$; found: 246.1471.

N-(methoxymethyl)benzamide (12)

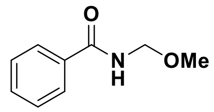

By following the general procedure, starting from *N*-methoxy-*N*-methylbenzamide (0.165 g, 1.0 mmol, 1.0 equiv), PMDTA (0.277 g, 0.33 mL, 1.6 mmol, 1.6 equiv) and *s*-BuLi (2.2 M in Et_2O, 0.73 mL, 1.6 mmol, 1.6 equiv) in dry 2-MeTHF (3 mL), compound **12** was obtained in 61% yield (0.101 g) as a colourless oil after purification by column chromatography on silica gel (*n*-hexane:EtOAc 7:3).

^{1}H NMR (400 MHz, Acetone-d_6) δ: 8.48 (brs, 1H, NH), 7.95 (m, 2H, Ph H-2,6), 7.55 (m, 1H, Ph H-4), 7.48 (m, 2H, Ph H-3,5), 4.80 (d, *J* = 6.6 Hz, 2H, CH_2), 3.30 (s, 3H, OCH_3).

^{13}C NMR (100 MHz, Acetone-d_6) δ: 168.0 (C=O), 135.4 (Ph C-1), 132.4 (Ph C-4), 129.3 (Ph C-3,5), 128.2 (Ph C-2,6), 72.3 (CH_2), 55.8 (CH_3).

HRMS (ESI), *m/z*: calcd. for $C_9H_{11}NNaO_2^+$: 188..0682 $[M + Na]^+$; found: 188.0680.

N-(methoxymethyl)-4-methylbenzamide (13)

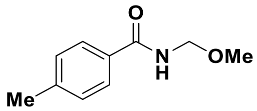

By following the general procedure, starting from *N*-methoxy-*N*,4-dimethylbenzamide (0.179 g, 1.0 mmol, 1.0 equiv), PMDTA (0.277 g, 0.33 mL, 1.6 mmol, 1.6 equiv) and *s*-BuLi (2.2 M in Et_2O, 0.73 mL, 1.6 mmol, 1.6 equiv) in dry 2-MeTHF (3 mL), compound **13** was obtained in 68% yield (0.121 g) as a white solid after purification by column chromatography on silica gel (*n*-hexane:EtOAc 7:3).

mp = 72-74 °C

^{1}H NMR (400 MHz, C_6D_6) δ: 7.59 (m, 2H, Ph H-2,6), 6.87 (m, 2H, Ph H-3,5), 6.29 (brs, 1H, NH), 4.72 (d, *J* = 6.8 Hz, 2H, CH_2), 3.22 (s, 3H, OCH_3), 2.01 (s, 3H, CH_3).

^{13}C NMR (100 MHz, C_6D_6) δ: 167.2 (C=O), 141.9 (Ph C-4), 131.9 (Ph C-1), 129.3 (Ph C-3,5), 127.7 (Ph C-2,6), 71.8 (CH_2), 55.9 (OCH_3), 21.2 (CH_3).

HRMS (ESI), *m/z*: calcd. for $C_{10}H_{13}NNaO_2^+$: 202.0838 $[M + Na]^+$; found: 202.0838.

N-(methoxymethyl)-3-methylbenzamide (14)

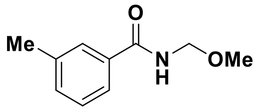

By following the general procedure, starting from *N*-methoxy-*N*,3-dimethylbenzamide (0.179 g, 1.0 mmol, 1.0 equiv), PMDTA (0.277 g, 0.33 mL, 1.6 mmol, 1.6 equiv) and *s*-BuLi (2.2 M in Et_2O, 0.73 mL, 1.6 mmol, 1.6 equiv) in dry 2-MeTHF (3 mL), compound **14** was obtained in 66% yield (0.118 g) as a colourless oil after purification by column chromatography on silica gel (*n*-hexane:EtOAc 7:3).

^{1}H NMR (400 MHz, Acetone-d_6) δ: 8.42 (brs, 1H, NH), 7.76 (m, 1H, Ph H-2), 7.73 (m, 1H, Ph H-6), 7.36 (m, 2H, Ph H-4,5), 4.79 (d, 2H, CH_2), 3.30 (s, 3H, OCH_3), 2.38 (s, 3H, CH_3).

^{13}C NMR (100 MHz, Acetone-d_6) δ: 168.1 (C=O), 138.9 (Ph C-3), 135.4 (Ph C-1), 133.0 (Ph C-4), 129.2 (Ph C-5), 128.8 (Ph C-2), 125.3 (Ph C-6), 72.2 (CH_2), 55.8 (OCH_3), 21.3 (CH_3).

^{15}N NMR (40 MHz, Acetone-d_6) δ: -265.9 (NH).

HRMS (ESI), *m/z*: calcd. for $C_{10}H_{13}NNaO_2^+$: 202.0838 $[M + Na]^+$; found: 202.0841.

2-ethyl-*N*-(methoxymethyl)benzamide (15)

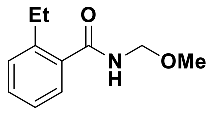

By following the general procedure, starting from 2-ethyl-*N*-methoxy-*N*-methylbenzamide (0.193 g, 1.0 mmol, 1.0 equiv), PMDTA (0.277 g, 0.33 mL, 1.6 mmol, 1.6 equiv) and *s*-BuLi (2.2 M in Et_2O, 0.73 mL, 1.6 mmol, 1.6 equiv) in dry 2-MeTHF (3 mL), compound **15** was obtained in 63% yield (0.121 g) as a colourless oil after purification by column chromatography on silica gel (*n*-hexane:EtOAc 7:3).

^{1}H NMR (400 MHz, $CDCl_3$) δ: 7.37 (m, 1H, Ph H-6), 7.36 (m, 1H, Ph H-4), 7.28 (m, 1H, Ph H-3), 7.21 (m, 1H, Ph H-5), 6.43 (brs, 1H, NH), 4.86 (d, J = 6.9 Hz, 2H, CH_2), 3.43 (s, 3H, OCH_3), 2.82 (q, ^3J = 7.5 Hz, 2H, $C\underline{H}_2CH_3$), 1.25 (t, ^3J = 7.5 Hz, 3H, $CH_2C\underline{H}_3$).

^{13}C NMR (100 MHz, $CDCl_3$) δ: 170.7 (C=O), 142.6 (Ph C-2), 135.5 (Ph C-1), 130.4 (Ph C-6), 129.6 (Ph C-3), 126.5 (Ph C-4), 125.8 (Ph C-5), 71.6 (CH_2), 56.2 (OCH_3), 26.3 ($\underline{C}H_2CH_3$), 15.9 ($CH_2\underline{C}H_3$).

HRMS (ESI), *m/z*: calcd. for $C_{11}H_{15}NNaO_2^+$: 216.0995 $[M + Na]^+$; found: 216.0993.

N-(methoxymethyl)-4-(2-methyl-2-propanyl)benzamide (16)

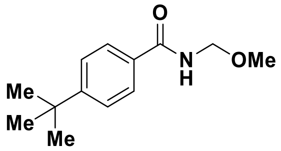

By following the general procedure, starting from 4-(*tert*-butyl)-*N*-methoxy-*N*-methylbenzamide (0.221 g, 1.0 mmol, 1.0 equiv), PMDTA (0.277 g, 0.33 mL, 1.6 mmol, 1.6 equiv) and *s*-BuLi (2.2 M in Et_2O, 0.73 mL, 1.6 mmol, 1.6 equiv) in dry 2-MeTHF (3 mL), compound **16** was obtained in 77% yield (0.170 g) as a colourless oil after purification by column chromatography on silica gel (*n*-hexane:EtOAc 7:3).

^{1}H NMR (400 MHz, Acetone-d_6) δ: 8.41 (brs, 1H, NH), 7.89 (m, 2H, Ph H-2,6), 7.52 (m, 2H, Ph H-3,5), 4.79 (d, *J* = 6.7 Hz, 2H, CH_2), 3.29 (s, 3H, OCH_3), 1.33 (s, 9H, CH_3).

^{13}C NMR (100 MHz, Acetone-d_6) δ: 167.8 (C=O), 155.6 (Ph C-4), 132.6 (Ph C-1), 128.1 (Ph C-2,6), 126.1 (Ph C-3,5), 72.2 (CH_2), 55.8 (OCH_3), 35.4 ($\underline{C}(CH_3)_3$), 31.4 (CH_3).

^{15}N NMR (40 MHz, Acetone-d_6) δ: -265.9 (NH).

HRMS (ESI), *m/z*: calcd. for $C_{13}H_{19}NNaO_2^+$: 244.1308 $[M + Na]^+$; found: 244.1312.

4-methoxy-*N*-(methoxymethyl)benzamide (17)

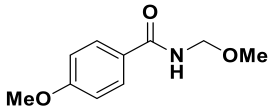

By following the general procedure, starting from *N*,4-dimethoxy-*N*-methylbenzamide (0.195 g, 1.0 mmol, 1.0 equiv), PMDTA (0.277 g, 0.33 mL, 1.6 mmol, 1.6 equiv) and *s*-BuLi (2.2 M in Et_2O, 0.73 mL, 1.6 mmol, 1.6 equiv) in dry 2-MeTHF (3 mL), compound **17** was obtained in 74% yield (0.144 g) as a white solid after purification by column chromatography on silica gel (*n*-hexane:EtOAc 6:4).

mp = 59-62 °C

^{1}H NMR (400 MHz, Acetone-d_6) δ: 8.37 (brs, 1H, NH), 7.93 (m, 2H, H-2,6), 7.00 (m, 2H, H-3,5), 4.78 (d, *J* = 6.7 Hz, 2H, CH_2), 3.29 (s, 3H, $CH_2O\underline{C}H_3$), 3.85 (s, 3H, OCH_3).

^{13}C NMR (100 MHz, Acetone-d_6) δ: 167.5 (C=O), 163.3 (Ph C-4), 130.0 (Ph C-2,6), 127.5 (Ph C-1), 114.4 (Ph C-3,5), 72.2 (CH_2), 55.79 (OCH_3), 55.74 ($CH_2O\underline{C}H_3$).

^{15}N NMR (40 MHz, Acetone-d_6) δ: -266.9 (NH).

HRMS (ESI), *m/z*: calcd. for $C_{10}H_{13}NNaO_3^+$: 218.0788 $[M + Na]^+$; found: 218.0779.

3-methoxy-*N*-(methoxymethyl)benzamide (18)

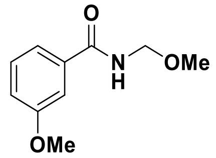

By following the general procedure, starting from *N*,3-dimethoxy-*N*-methylbenzamide (0.195 g, 1.0 mmol, 1.0 equiv), PMDTA (0.277 g, 0.33 mL, 1.6 mmol, 1.6 equiv) and *s*-BuLi (2.2 M in Et_2O, 0.73 mL, 1.6 mmol, 1.6 equiv) in dry 2-MeTHF (3 mL), compound **18** was obtained in 67% yield (0.130 g) as a colourless oil after purification by column chromatography on silica gel (*n*-hexane:EtOAc 6:4).

^{1}H NMR (400 MHz, $CDCl_3$) δ: 7.39 (m, 1H, Ph H-2), 7.36 (m, 1H, Ph H-5), 7.31 (m, 1H, Ph H-6), 7.07 (ddd, ^3J = 7.8 Hz, ^4J = 2.6 Hz, ^5J = 1.4 Hz, 1H, Ph H-4), 6.77 (brs, 1H, NH), 4.90 (d, *J* = 6.8 Hz, 2H, CH_2), 3.85 (s, 3H, OCH_3), 3.41 (s, 3H, $CH_2OC\underline{H}_3$).

^{13}C NMR (100 MHz, $CDCl_3$) δ: 167.8 (C=O), 159.9 (Ph C-3), 135.3 (Ph C-1), 129.7 (Ph C-5), 118.7 (Ph C- 6), 118.2 (Ph C-4), 112.4 (Ph C-2), 71.9 (CH_2), 56.2 ($CH_2O\underline{C}H_3$), 55.5 (OCH_3).

HRMS (ESI), *m/z*: calcd. for $C_{10}H_{13}NNaO_3^+$: 218.0788 $[M + Na]^+$; found: 218.0788.

3-methoxy-*N*-(methoxymethyl)-2-methylbenzamide (19)

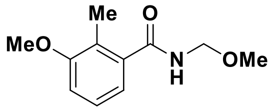

By following the general procedure, starting from *N*,3-dimethoxy-*N*,2-dimethylbenzamide (0.209 g, 1.0 mmol, 1.0 equiv), PMDTA (0.277 g, 0.33 mL, 1.6 mmol, 1.6 equiv) and *s*-BuLi (2.2 M in Et_2O, 0.73 mL, 1.6 mmol, 1.6 equiv) in dry 2-MeTHF (3 mL), compound **19** was obtained in 65% yield (0.135 g) as a pale yellow solid after purification by column chromatography on silica gel (*n*-hexane:EtOAc 6:4).

mp = 32-35 °C

^{1}H NMR (400 MHz, Acetone-d_6) δ: 8.03 (brs, 1H, NH), 7.20 (m, 1H, Ph H-5), 7.00 (m, 1H, Ph H-4), 6.92 (m, 1H, Ph H-6), 4.76 (d, *J* = 6.8 Hz, 2H, CH_2), 3.85 (s, 3H, OCH_3), 3.34 (s, 3H, $CH_2OC\underline{H_3}$) 2.23 (s, 3H, CH_3).

^{13}C NMR (100 MHz, Acetone-d_6) δ: 170.8 (C=O), 139.5 (Ph C-1), 158.8 (Ph C-3), 127.3 (Ph C-5), 124.9 (Ph C-2), 119.6 (Ph C-6), 112.1 (Ph C-4), 71.9 (CH_2), 56.0 (OCH_3), 55.9 ($CH_2O\underline{C}H_3$), 12.7 (CH_3).

^{15}N NMR (40 MHz, Acetone-d_6) δ: -256.3 (NH).

HRMS (ESI), *m/z*: calcd. for $C_{11}H_{15}NNaO_3^+$: 232.0944 $[M + Na]^+$; found: 232.0933.

(*N*-methoxymethyl)-4-(methylsulfanyl)benzamide (20)

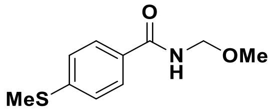

By following the general procedure, starting from *N*-methoxy-*N*-methyl-4-(methylthio)benzamide (0.211 g, 1.0 mmol, 1.0 equiv), PMDTA (0.277 g, 0.33 mL, 1.6 mmol, 1.6 equiv) and *s*-BuLi (2.2 M in Et_2O, 0.73 mL, 1.6 mmol, 1.6 equiv) in dry 2-MeTHF (3 mL), compound **20** was obtained in 72% yield (0.152 g) as a pale yellow solid after purification by column chromatography on silica gel (*n*-hexane:EtOAc 6:4).

mp = 87-90 °C

^{1}H NMR (400 MHz, Acetone-d$_6$) δ: 8.42 (brs, 1H, NH), 7.88 (m, 2H, Ph H-2,6), 7.34 (m, 2H, Ph H-3,5), 4.79 (d, *J* = 6.6 Hz, 2H, CH$_2$), 3.29 (s, 3H, OCH$_3$), 2.54 (s, 3H, SCH$_3$).

^{13}C NMR (100 MHz, Acetone-d$_6$) δ: 167.4 (C=O), 144.5 (Ph C-4), 131.4 (Ph C-1), 128.7 (Ph C-2,6), 125.9 (Ph C-3,5), 72.3 (CH$_2$), 55.8 (OCH$_3$), 14.7 (SCH$_3$).

^{15}N NMR (40 MHz, Acetone-d$_6$) δ: -266.2 (NH).

HRMS (ESI), *m/z*: calcd. for $C_{10}H_{13}NNaO_2S^+$: 234.0559 [M + Na]$^+$; found: 234.0562.

2-chloro-*N*-(methoxymethyl)benzamide (21)

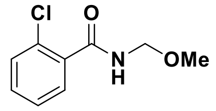

By following the general procedure, starting from 2-chloro-*N*-methoxy-*N*-methylbenzamide (0.199 g, 1.0 mmol, 1.0 equiv), PMDTA (0.277 g, 0.33 mL, 1.6 mmol, 1.6 equiv) and *s*-BuLi (2.2 M in Et$_2$O, 0.73 mL, 1.6 mmol, 1.6 equiv) in dry 2-MeTHF (3 mL), compound **21** was obtained in 77% yield (0.153 g) as a white solid after purification by column chromatography on silica gel (*n*-hexane:EtOAc 7:3).

mp = 65-67 °C

^{1}H NMR (400 MHz, Acetone-d$_6$) δ: 8.21 (brs, 1H, NH), 7.52 (m, 1H, Ph H-6), 7.47 (m, 1H, Ph H-4), 7.46 (m, 1H, Ph H-3), 7.39 (m, 1H, Ph H-5), 4.79 (d, *J* = 6.7 Hz, 2H, CH$_2$), 3.36 (s, 3H, OCH$_3$).

^{13}C NMR (100 MHz, Acetone-d$_6$) δ: 168.0 (C=O), 137.7 (Ph C-2), 131.8 (Ph C-4), 131.2 (Ph C-1), 130.7 (Ph C-3), 129.9 (Ph C-6), 127.9 (Ph C-5), 71.9 (CH$_2$), 56.0 (OCH$_3$).

^{15}N NMR (40 MHz, Acetone-d$_6$) δ: -255.7 (NH).

HRMS (ESI), *m/z*: calcd. for $C_9H_{10}ClNNaO_2^+$: 222.0292 [M + Na]$^+$; found: 222.0291.

3-chloro-*N*-(methoxymethyl)benzamide (22)

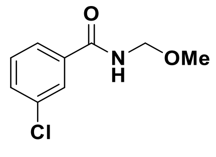

By following the general procedure, starting from 3-chloro-*N*-methoxy-*N*-methylbenzamide (0.199 g, 1.0 mmol, 1.0 equiv), PMDTA (0.277 g, 0.33 mL, 1.6 mmol, 1.6 equiv) and *s*-BuLi (2.2 M in Et$_2$O, 0.73 mL, 1.6 mmol, 1.6 equiv) in dry 2-MeTHF (3 mL), compound **22** was obtained in 76% yield (0.151 g) as a colourless oil after purification by column chromatography on silica gel (*n*-hexane:EtOAc 7:3).

^{1}H NMR (400 MHz, C_6D_6) δ: 7.58 (m, 1H, Ph H-2), 7.39 (m, 1H, Ph H-6), 7.05 (m, 1H, Ph H-4), 6.71 (m, 1H, Ph H-5), 6.04 (brs, 1H, NH), 4.60 (d, *J* = 6.8 Hz, 2H, CH$_2$), 3.16 (s, 3H, OCH$_3$).

^{13}C NMR (100 MHz, C_6D_6) δ: 166.0 (C=O), 136.4 (Ph C-1), 134.7 (Ph C-3), 131.6 (Ph C-4), 130.0 (Ph C-5), 127.5 (Ph C-2), 125.9 (Ph C-6), 71.8 (CH_2), 56.0 (OCH_3).

HRMS (ESI), *m/z*: calcd. for $C_9H_{10}ClNNaO_2^+$: 222.0292 $[M + Na]^+$; found: 222.0289.

4-chloro-*N*-(methoxymethyl)benzamide (23)

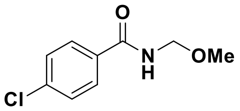

By following the general procedure, starting from 4-chloro-*N*-methoxy-*N*-methylbenzamide (0.199 g, 1.0 mmol, 1.0 equiv), PMDTA (0.277 g, 0.33 mL, 1.6 mmol, 1.6 equiv) and *s*-BuLi (2.2 M in Et_2O, 0.73 mL, 1.6 mmol, 1.6 equiv) in dry 2-MeTHF (3 mL), compound **23** was obtained in 79% yield (0.157 g) as a yellow solid after purification by column chromatography on silica gel (*n*-hexane:EtOAc 7:3).

Scale up of the reaction using 20 mmol of starting material.

By following the general procedure, starting from 4-chloro-*N*-methoxy-*N*-methylbenzamide (3.992 g, 20.0 mmol, 1.0 equiv), PMDTA (5.440 g, 6.60 mL, 32 mmol, 1.6 equiv) and *s*-BuLi (2.2 M in Et_2O, 14.6 mL, 32 mmol, 1.6 equiv) in dry 2-MeTHF (60 mL), compound **23** was obtained in 73% yield (2.914 g) as a yellow solid after purification by column chromatography on silica gel (*n*-hexane:EtOAc 7:3).

Spectroscopic and spectrometric data match with those ones reported for running the reaction at 1 mmol scale.

mp = 65-70 °C

^{1}H NMR (400 MHz, Acetone-d$_6$) δ: 8.53 (brs, 1H, NH), 7.96 (m, 2H, Ph H-2,6), 7.52 (m, 2H, Ph H-3,5), 4.80 (d, *J* = 6.6 Hz, 2H, CH_2), 3.30 (s, 3H, OCH_3).

^{13}C NMR (100 MHz, Acetone-d$_6$) δ: 166.9 (C=O), 138.0 (Ph C-4), 134.1 (Ph C-1), 130.0 (Ph C-2,6), 129.4 (Ph C-3,5), 72.3 (CH_2), 55.9 (OCH_3).

^{15}N NMR (40 MHz, Acetone-d$_6$) δ: -265.3 (NH).

HRMS (ESI), *m/z*: calcd. for $C_9H_{10}ClNNaO_2^+$: 222.0292 $[M + Na]^+$; found: 222.0280.

3-fluoro-*N*-(methoxymethyl)benzamide (24)

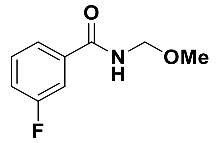

By following the general procedure, starting from 3-fluoro-*N*-methoxy-*N*-methylbenzamide (0.183 g, 1.0 mmol, 1.0 equiv), PMDTA (0.277 g, 0.33 mL, 1.6 mmol, 1.6 equiv) and *s*-BuLi (2.2 M in Et_2O, 0.73 mL, 1.6

mmol, 1.6 equiv) in dry 2-MeTHF (3 mL), compound **24** was obtained in 78% yield (0.143 g) as a pale yellow solid after purification by column chromatography on silica gel (*n*-hexane:EtOAc 7:3).

mp = 42-45 °C

^{1}H NMR (400 MHz, $CDCl_3$) δ: 7.56 (m, 1H, Ph H-6), 7.54 (m, 1H, Ph H-2), 7.42 (m, 1H, Ph H-5), 7.22 (m, 1H, Ph H-4), 6.92 (brs, 1H, NH), 4.89 (d, *J* = 6.8 Hz, 2H, CH_2), 3.40 (s, 3H, OCH_3).

^{13}C NMR (100 MHz, $CDCl_3$) δ: 166.7 (C=O), 162.8 (d, $^{1}J_{C,F}$ = 248.1 Hz, Ph C-3), 136.1 (d, $^{3}J_{C,F}$ = 6.8 Hz, Ph C-1), 130.9 (d, $^{3}J_{C,F}$ = 7.9 Hz, Ph C-5), 122.5 (d, $^{4}J_{C,F}$ = 3.1 Hz, Ph C-6), 119.0 (d, $^{2}J_{C,F}$ = 21.3 Hz, Ph C-4), 114.6 (d, $^{2}J_{C,F}$ = 23.0 Hz, Ph C-2), 71.9 (CH_2), 56.3 (CH_3).

^{15}N NMR (40 MHz, $CDCl_3$) δ: -264.5 (NH).

^{19}F NMR (376 MHz, $CDCl_3$) δ: -111.5 (m).

HRMS (ESI), *m/z*: calcd. for $C_9H_{10}FNNaO_2^+$: 206.0588 $[M + Na]^+$; found: 206.0590.

2,5-difluoro-*N*-(methoxymethyl)benzamide (25)

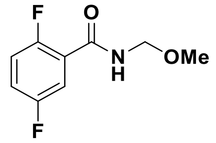

By following the general procedure, starting from 2,5-difluoro-*N*-methoxy-*N*-methylbenzamide (0.201 g, 1.0 mmol, 1.0 equiv), PMDTA (0.277 g, 0.33 mL, 1.6 mmol, 1.6 equiv) and *s*-BuLi (2.2 M in Et_2O, 0.73 mL, 1.6 mmol, 1.6 equiv) in dry 2-MeTHF (3 mL), compound **25** was obtained in 60% yield (0.120 g) as a yellowish oil after purification by column chromatography on silica gel (*n*-hexane:EtOAc 8:2).

^{1}H NMR (400 MHz, $CDCl_3$) δ: 7.81 (m, 1H, Ph), 7.33 (brs, 1H, NH), 7.20 (m, 1H, Ph), 7.13 (m, 1H, Ph), 4.92 (dd, *J* = 6.6 Hz, *J* = 1.6 Hz, 2H, CH_2), 3.41 (s, 3H, OCH_3).

^{13}C NMR (100 MHz, $CDCl_3$) δ: 162.8 (m, C=O), 158.8 (m, Ph), 156.5 (m, Ph), 121.9 (dd, $^{2}J_{C,F}$ = 14.1 Hz, $^{3}J_{C,F}$ = 7.4 Hz, Ph C-1), 120.5 (dd, *J* = 24.6 Hz, *J* = 9.9 Hz, Ph), 118.4 (dd, *J* = 25.9 Hz, *J* = 2.8 Hz, Ph), 117.6 (dd, *J* = 28.2 Hz, *J* = 8.0 Hz, Ph), 71.9 (CH_2), 56.3 (OCH_3).

^{19}F NMR (376 MHz, $CDCl_3$) δ: -119.1 (m), -116.7 (m).

HRMS (ESI), *m/z*: calcd. for $C_9H_9F_2NNaO_2^+$: 224.0494 $[M + Na]^+$; found: 224.0492.

2,6-difluoro-*N*-(methoxymethyl)benzamide (26)

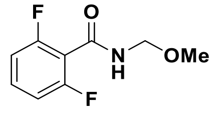

By following the general procedure, starting from 2,6-difluoro-*N*-methoxy-*N*-methylbenzamide (0.201 g, 1.0 mmol, 1.0 equiv), PMDTA (0.277 g, 0.33 mL, 1.6 mmol, 1.6 equiv) and *s*-BuLi (2.2 M in Et_2O, 0.73 mL, 1.6

mmol, 1.6 equiv) in dry 2-MeTHF (3 mL), compound **26** was obtained in 65% yield (0.131 g) as a white solid after purification by column chromatography on silica gel (*n*-hexane:EtOAc 6:4).

mp = 52-55 °C

^{1}H NMR (400 MHz, Acetone-d$_6$) δ: 8.44 (brs, 1H, NH), 7.52 (m, 1H, Ph H-4), 7.09 (m, 2H, Ph H-3,5), 4.80 (d, *J* = 6.7 Hz, 2H, CH$_2$), 3.35 (s, 3H, OCH$_3$).

^{13}C NMR (100 MHz, Acetone-d$_6$) δ: 161.1 (C=O), 160.4 (dd, $^{1}J_{C,F}$ = 249.3 Hz, $^{3}J_{C,F}$ = 7.8 Hz, Ph C-2,6), 132.6 (t, $^{3}J_{C,F}$ = 10.0 Hz, Ph C-4), 112.6 (m, Ph C-3,5), 71.8 (CH$_2$), 55.9 (OCH$_3$).

^{19}F NMR (376 MHz, Acetone-d$_6$) δ: -115.0 (m).

HRMS (ESI), *m/z*: calcd. for $C_9H_9F_2NNaO_2^+$: 224.0494 [M + Na]$^+$; found: 224.0498.

2-fluoro-*N*-(methoxymethyl)-5-(trifluoromethyl)benzamide (27)

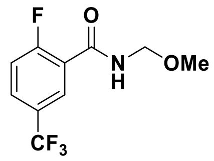

By following the general procedure, starting from 2-fluoro-*N*-methoxy-*N*-methyl-5-(trifluoromethyl)benzamide (0.251 g, 1.0 mmol, 1.0 equiv), PMDTA (0.277 g, 0.33 mL, 1.6 mmol, 1.6 equiv) and *s*-BuLi (2.2 M in Et$_2$O, 0.73 mL, 1.6 mmol, 1.6 equiv) in dry 2-MeTHF (3 mL), compound **27** was obtained in 73% yield (0.183 g) as a yellow solid after purification by column chromatography on silica gel (*n*-hexane:EtOAc 8:2).

mp: 57 °C

^{1}H NMR (400 MHz, C$_6$D$_6$) δ: 8.43 (dd, ^{4}J = 7.0 Hz, ^{4}J = 2.5 Hz, 1 H, Ph H-6), 6.99 (m, 1H, Ph H-4), 6.47 (brs, 1H, NH), 6.32 (dd, *J* = 10.9 Hz, *J* = 8.8 Hz, 1H, Ph H-3), 4.56 (dd, *J* = 6.7 Hz, *J* = 1.4 Hz, 2H, CH$_2$), 3.16 (s, 3H, OCH$_3$).

^{13}C NMR (100 MHz, C$_6$D$_6$) δ: 162.0 (d, $^{3}J_{C,F}$ = 3.1 Hz, C=O), 162.0 (d, $^{1}J_{C,F}$ = 253.0 Hz, Ph C-2), 130.3 (m, Ph C-4), 130.1 (m, Ph C-6), 127.6 (m, Ph C-5), 124.0 (q, $^{1}J_{C,F}$ = 272.2 Hz, CF$_3$), 122.5 (d, $^{2}J_{C,F}$ = 13.8 Hz, Ph C-1), 116.9 (d, $^{3}J_{C,F}$ = 26.2 Hz, Ph C-3), 71.8 (CH$_2$), 56.1 (OCH$_3$).

^{15}N NMR (40 MHz, C$_6$D$_6$) δ: -259.0 (NH).

^{19}F NMR (376 MHz, C$_6$D$_6$) δ: -109.0 (m, F), -62.1 (d, *J* = 1.3 Hz, CF$_3$).

HRMS (ESI), *m/z*: calcd. for $C_{10}H_9F_4NNaO_2^+$: 274.0462 [M + Na]$^+$; found: 274.0461.

4-hydroxy-*N*-(methoxymethyl)benzamide (28)

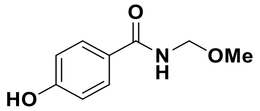

4-Hydroxy-*N*-methoxy-*N*-methylbenzamide (0.181 g, 1.0 mmol, 1.0 equiv) was pretreated in dry 2-MeTHF (3 mL) at -78 °C with MeLi (1.6 M Et_2O, 0.61 mL, 0.98 mmol) for 5 min. Then, by following the general procedure, PMDTA (0.277 g, 0.33 mL, 1.6 mmol, 1.6 equiv) and *s*-BuLi (2.2 M in Et_2O, 0.73 mL, 1.6 mmol, 1.6 equiv) were added, obtaining compound **28** in 77% yield (0.139 g) as colourless cristals after purification by column chromatography on silica gel (DCM:EtOAc 6:4).

mp = 156 °C

^{1}H NMR (400 MHz, Acetone-d_6) δ: 9.01 (s, 1H, OH), 8.27 (brs, 1H, NH), 7.84 (m, 2H, Ph H-2,6), 6.89 (m, 2H, Ph H-3,5), 4.77 (d, *J* = 6.7 Hz, 2H, CH_2), 3.28 (s, 3H, OCH_3).

^{13}C NMR (100 MHz, Acetone-d_6) δ: 167.5 (C=O), 161.4 (Ph C-4), 130.2 (Ph C-2,6), 126.5 (Ph C-1), 115.8 (Ph C-3,5), 72.2 (CH_2), 55.7 (OCH_3).

^{15}N NMR (40 MHz, Acetone-d_6) δ: -267.5 (NH).

HRMS (ESI), *m/z*: calcd. for $C_9H_{11}NNaO_3^+$: 204.0631 $[M + Na]^+$; found: 204.0623.

4-amino-*N*-(methoxymethyl)benzamide (29)

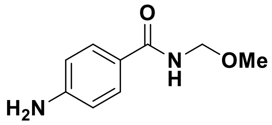

4-Amino-*N*-methoxy-*N*-methylbenzamide (0.180 g, 1.0 mmol, 1.0 equiv) was pretreated in dry 2-MeTHF (3 mL) at -78 °C with MeLi (1.6 M Et_2O, 0.61 mL, 0.98 mmol) for 5 min. Then, by following the general procedure, PMDTA (0.277 g, 0.33 mL, 1.6 mmol, 1.6 equiv) and *s*-BuLi (2.2 M in Et_2O, 0.73 mL, 1.6 mmol, 1.6 equiv) were added, obtaining compound **29** in 75% yield (0.135 g) as a yellow solid after purification by column chromatography on silica gel (DCM:EtOAc 6:4).

mp = 123 °C

^{1}H NMR (400 MHz, Acetone-d_6) δ: 8.08 (brs, 1H, NH), 7.71 (m, 2H, Ph H-2,6), 6.68 (m, 2H, Ph H-3,5), 5.18 (brs, 2H, NH_2), 4.75 (d, *J* = 6.7 Hz, 2H, CH_2), 3.26 (s, 3H, OCH_3).

^{13}C NMR (100 MHz, Acetone-d_6) δ: 167.8 (C=O), 152.7 (Ph C-4), 129.9 (Ph C-2,6), 122.9 (Ph C-1), 114.0 (Ph C-3,5), 72.1 (CH_2), 55.6 (OCH_3).

^{15}N NMR (40 MHz, Acetone-d_6) δ: -320.3 (NH_2), -269.2 (NH).

HRMS (ESI), *m/z*: calcd. for $C_9H_{12}N_2NaO_2^+$: 203.0791 $[M + Na]^+$; found: 203.0791.

3-ethynyl-*N*-(methoxymethyl)benzamide (30)

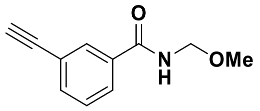

3-Ethynyl-*N*-methoxy-*N*-methylbenzamide (0.189 g, 1.0 mmol, 1.0 equiv) was pretreated in dry 2-MeTHF (3 mL) at -78 °C with MeLi (1.6 M Et_2O, 0.61 mL, 0.98 mmol) for 15 min. Then, by following the general procedure, PMDTA (0.277 g, 0.33 mL, 1.6 mmol, 1.6 equiv) and *s*-BuLi (2.2 M in Et_2O, 0.73 mL, 1.6 mmol, 1.6 equiv) were added, obtaining compound **30** in 62% yield (0.117 g) as a yellow solid after purification by column chromatography on silica gel (*n*-hexane:EtOAc 7:3).

mp = 58-61 °C

^{1}H NMR (400 MHz, Acetone-d_6) δ: 8.56 (brs, 1H, NH), 8.03 (m, 1H, Ph H-2), 7.96 (m, 1H, Ph H-6), 7.66 (m, 1H, Ph H-4), 7.51 (m, 1H, Ph H-5), 4.81 (d, *J* = 6.6 Hz, 2H, CH_2), 3.74 (s, 1H, CCH), 3.31 (s, 3H, OCH_3).

^{13}C NMR (100 MHz, Acetone-d_6) δ: 167.1 (C=O), 135.8 (Ph C-1), 135.5 (Ph C-4), 131.6 (Ph C-2), 129.7 (Ph C-5), 128.7 (Ph C-6), 123.5 (Ph C-3), 83.4 (<u>C</u>CH), 80.0 (C<u>C</u>H), 72.3 (CH_2), 55.9 (OCH_3).

^{15}N NMR (40 MHz, Acetone-d_6) δ: -264.8 (NH).

HRMS (ESI), *m/z*: calcd. for $C_{11}H_{11}NNaO_2^+$: 212.0682 $[M + Na]^+$; found: 212.0688.

N-(methoxymethyl)-2-naphthamide (31)

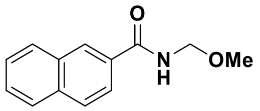

By following the general procedure, starting from *N*-methoxy-*N*-methyl-2-naphthamide (0.215 g, 1.0 mmol, 1.0 equiv), PMDTA (0.277 g, 0.33 mL, 1.6 mmol, 1.6 equiv) and *s*-BuLi (2.2 M in Et_2O, 0.73 mL, 1.6 mmol, 1.6 equiv) in dry 2-MeTHF (3 mL), compound **31** was obtained in 60% yield (0.129 g) as a colourless oil after purification by column chromatography on silica gel (*n*-hexane:EtOAc 7:3).

^{1}H NMR (400 MHz, Acetone-d_6) δ: 8.63 (brs, 1H, NH), 8.52 (m, 1H, Naph H-1), 7.96–8.05 (m, 4H), 7.57–7.65 (m, 2H), 4.87 (d, *J* = 6.7 Hz, 2H, CH_2), 3.34 (s, 3H, OCH_3).

^{13}C NMR (100 MHz, Acetone-d_6) δ: 168.0 (C=O), 135.8 (Naph C-4a), 133.6 (Naph), 132.7 (Naph), 129.8 (Naph C-3), 129.0 (Naph), 128.59 (Naph), 128.58 (Naph), 128.54 (Naph C-1), 127.6 (Naph), 125.0 (Naph), 72.4 (CH_2), 55.9 (OCH_3).

HRMS (ESI), *m/z*: calcd. for $C_{13}H_{13}NNaO_2^+$: 238.0838 $[M + Na]^+$; found: 238.0841.

N-(methoxymethyl)-2-furamide (32)

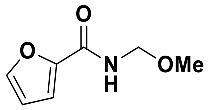

By following the general procedure, starting from *N*-methoxy-*N*-methylfuran-2-carboxamide (0.155 g, 1.0 mmol, 1.0 equiv), PMDTA (0.277 g, 0.33 mL, 1.6 mmol, 1.6 equiv) and *s*-BuLi (2.2 M in Et_2O, 0.73 mL, 1.6 mmol, 1.6 equiv) in dry 2-MeTHF (3 mL), compound **32** was obtained in 62% yield (0.096 g) as a yellowish oil after purification by column chromatography on silica gel (*n*-hexane:EtOAc 6:4).

^{1}**H NMR** (400 MHz, C_6D_6) δ: 7.03 (dd, ^3J = 3.5 Hz, ^4J = 0.8 Hz, 1H, furan H-3), 6.72 (dd, ^3J = 1.8 Hz, ^4J = 0.8 Hz, 1H, furan H-5), 6.29 (brs, 1H, NH), 5.86 (dd, ^3J = 3.5 Hz, ^3J = 1.8 Hz, 1H, furan H-4), 4.55 (d, J = 7.0 Hz, 2H, CH_2), 3.14 (s, 3H, OCH_3).

^{13}**C NMR** (100 MHz, C_6D_6) δ: 158.3 (C=O), 148.4 (furan C-2), 143.6 (furan C-5), 115.0 (furan C-3), 112.3 (furan C-4), 71.0 (CH_2), 55.8 (OCH_3).

^{15}**N NMR** (40 MHz, C_6D_6) δ: -268.3 (NH).

HRMS (ESI), *m/z*: calcd. for $C_7H_9NNaO_3^+$: 178.0475 $[M + Na]^+$; found: 178.0472.

N-(methoxymethyl)-2-tiophencarboxamide (33)

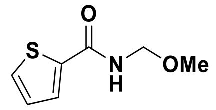

By following the general procedure, starting from *N*-methoxy-*N*-methylthiophene-2-carboxamide (0.171 g, 1.0 mmol, 1.0 equiv), PMDTA (0.277 g, 0.33 mL, 1.6 mmol, 1.6 equiv) and *s*-BuLi (2.2 M in Et_2O, 0.73 mL, 1.6 mmol, 1.6 equiv) in dry 2-MeTHF (3 mL), compound **33** was obtained in 65% yield (0.111 g) as a yellow solid after purification by column chromatography on silica gel (*n*-hexane:EtOAc 7:3).

mp = 63 °C

^{1}**H NMR** (400 MHz, Acetone-d$_6$) δ: 8.45 (brs, 1H, NH), 7.79 (dd, ^3J = 3.7 Hz, ^4J = 1.1 Hz, 1H, thiophen H-3), 7.72 (dd, ^3J = 5.0 Hz, ^4J = 1.1 Hz, 1H, thiophen H-5), 7.14 (dd, ^3J = 5.0 Hz, ^3J = 3.7 Hz, 1H, thiophen H-4), 4.76 (d, J = 6.7 Hz, 2H, CH_2), 3.29 (s, 3H, OCH_3).

^{13}**C NMR** (100 MHz, Acetone-d$_6$) δ: 162.8 (C=O), 140.7 (thiophen C-2), 131.8 (thiophen C-5), 129.0 (thiophen C-3), 128.6 (thiophen C-4), 72.1 (CH_2), 55.8 (OCH_3).

^{15}**N NMR** (40 MHz, Acetone-d$_6$) δ: -266.4 (NH).

HRMS (ESI), *m/z*: calcd. for $C_7H_9NNaO_2S^+$: 194.0246 $[M + Na]^+$; found: 194.0239.

N-(methoxymethyl)-4-(tributylstannyl)benzamide (34)

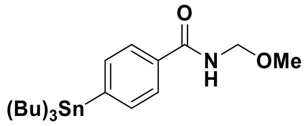

By following the general procedure, starting from *N*-methoxy-*N*-methyl-4-(tributylstannyl)benzamide (0.455 g, 1.0 mmol, 1.0 equiv), PMDTA (0.277 g, 0.33 mL, 1.6 mmol, 1.6 equiv) and *s*-BuLi (2.2 M in Et_2O, 0.73 mL, 1.6 mmol, 1.6 equiv) in dry 2-MeTHF (3 mL), compound **34** was obtained in 61% yield (0.278 g) as a colourless oil after purification by column chromatography on silica gel (*n*-hexane:EtOAc 7:3).

^{1}H NMR (400 MHz, C_6D_6) δ: 7.67 (m, 2H, Ph H-2,6), 7.50 (m, 2H, Ph H-3,5), 6.07 (brt, *J* = 6.8 Hz, 1H, NH), 4.70 (d, *J* = 6.8 Hz, 2H, CH_2), 3.23 (s, 3H, OCH_3), 1.59 (m, 6H, Bu H-2), 1.36 (m, 6H, Bu H-3), 1.08 (m, 6H, Bu H-1), 0.91 (t, *J* = 7.3 Hz, 9H, CH_3).

^{13}C NMR (100 MHz, C_6D_6) δ: 167.4 (C=O), 147.1 (Ph C-4), 136.8 (Ph C-3,5), 134.4 (Ph C-1), 126.8 (Ph C-2,6), 71.8 (CH_2), 55.9 (OCH_3), 29.5 (Bu C-2), 27.7 (Bu C-3), 13.9 (Bu C-4), 9.9 (Bu C-1).

^{15}N NMR (40 MHz, C_6D_6) δ: -266.8 (NH).

^{119}Sn NMR (149 MHz, C_6D_6) δ: -42.3 (s).

HRMS (ESI), *m/z*: calcd. for $C_{21}H_{37}NNaO_2Sn^+$: 478.1739 $[M + Na]^+$; found: 478.1738.

4-(*sec*-butylselanyl)-*N*-(methoxymethyl)benzamide (35)

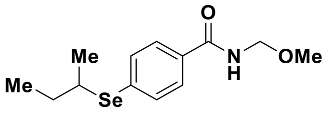

By following the general procedure, starting from *N*-methoxy-*N*-methyl-4-(phenylselanyl)benzamide (0.320 g, 1.0 mmol, 1.0 equiv), PMDTA (0.277 g, 0.33 mL, 1.6 mmol, 1.6 equiv) and *s*-BuLi (2.2 M in Et_2O, 0.73 mL, 1.6 mmol, 1.6 equiv) in dry 2-MeTHF (3 mL), compound **35** was obtained in 66% yield (0.211 g) as a colourless oil after purification by column chromatography on silica gel (*n*-hexane:EtOAc 7:3).

^{1}H NMR (400 MHz, Acetone-d$_6$) δ: 8.46 (brs, 1H, NH), 7.85 (m, 2H, Ph H-2,6), 7.61 (m, 2H, Ph H-3,5), 4.79 (d, *J* = 6.6 Hz, 2H, CH_2), 3.48 (sept., *J* = 6.7 Hz, 1H, CH), 3.30 (s, 3H, OCH_3), 1.69 (m, 2H, $C\underline{H}_2CH_3$), 1.43 (d, *J* = 6.9 Hz, 3H, $CHC\underline{H}_3$), 1.01 (t, *J* = 7.4 Hz, 3H, $CH_2C\underline{H}_3$),

^{13}C NMR (100 MHz, Acetone-d$_6$) δ: 167.5 (C=O), 135.9 (Ph C-1,4), 133.7 (Ph C-3,5), 128.7 (Ph C-2,6), 72.3 (CH_2), 55.9 (OCH_3), 41.7 (CH), 31.1 ($\underline{C}H_2CH_3$), 21.8 ($CH\underline{C}H_3$), 12.5 ($CH_2\underline{C}H_3$).

^{77}Se NMR (76 MHz, Acetone-d$_6$) δ: 394.3 (s).

HRMS (ESI), *m/z*: calcd. for $C_{13}H_{19}NNaO_2Se^+$: 324.0473 $[M + Na]^+$; found: 324.0476.

***N,N*-diethyl-*N'*-(methoxymethyl)terephthalamide (36)**

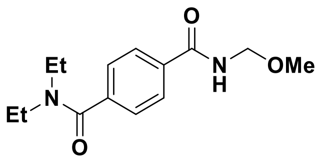

By following the general procedure, starting from *N,N*-diethyl-*N*′-methoxy-*N*′-methylterephthalamide (0.264 g, 1.0 mmol, 1.0 equiv), PMDTA (0.277 g, 0.33 mL, 1.6 mmol, 1.6 equiv) and *s*-BuLi (2.2 M in Et_2O, 0.73 mL, 1.6 mmol, 1.6 equiv) in dry 2-MeTHF (3 mL), compound **36** was obtained in 68% yield (0.179 g) as a pale yellow solid after purification by column chromatography on silica gel (DCM:EtOAc 6:4).

mp: 65-67 °C

^{1}H NMR (400 MHz, Acetone-d_6) δ: 8.54 (brs, 1H, NH), 7.99 (m, 2H, Ph H-3,5), 7.46 (m, 2H, Ph H-2,6), 4.81 (d, *J* = 6.6 Hz, 2H, CH_2), 3.51 (brs, 2H, $NC\underline{H}_2CH_3$), 3.31 (s, 3H, OCH_3), 3.27 (brs, 2H, $NC\underline{H}_2CH_3$), 1.18 (brs, 3H, $NCH_2C\underline{H}_3$), 1.13 (brs, 3H, $NCH_2C\underline{H}_3$).

^{13}C NMR (100 MHz, Acetone-d_6) δ: 170.4 (C=O), 167.4 (C=O), 141.9 (Ph C-1), 135.6 (Ph C-4), 128.3 (Ph C-3,5), 127.2 (Ph C-2,6), 72.3 (CH_2), 55.9 (OCH_3), 43.8 ($N\underline{C}H_2CH_3$), 39.8 ($N\underline{C}H_2CH_3$), 14.4 ($NCH_2\underline{C}H_3$), 13.2 ($NCH_2\underline{C}H_3$).

HRMS (ESI), *m/z*: calcd. for $C_{14}H_{21}N_2O_3^+$: 265.1547 [M + H]$^+$; found: 265.1534.

_N_-(ethoxymethyl)benzamide (37)

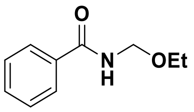

By following the general procedure, starting from *N*-ethoxy-*N*-methylbenzamide (0.179 g, 1.0 mmol, 1.0 equiv), PMDTA (0.277 g, 0.33 mL, 1.6 mmol, 1.6 equiv) and *s*-BuLi (2.2 M in Et_2O, 0.73 mL, 1.6 mmol, 1.6 equiv) in dry 2-MeTHF (3 mL), compound **37** was obtained in 74% yield (0.132 g) as a white solid after purification by column chromatography on silica gel (*n*-hexane:EtOAc 7:3).

mp = 33-36 °C

^{1}H NMR (400 MHz, Acetone-d_6) δ: 8.45 (brs, 1H, NH), 7.94 (m, 2H, Ph H-2,6), 7.55 (m, 1H, Ph H-4), 7.47 (m, 2H, Ph H-3,5), 4.86 (d, *J* = 6.6 Hz, 2H, CH_2), 3.56 (q, *J* = 7.0 Hz, 2H, $OC\underline{H}_2CH_3$), 1.13 (t, *J* = 7.0 Hz, 3H, $OCH_2C\underline{H}_3$).

^{13}C NMR (100 MHz, Acetone-d_6) δ: 167.8 (C=O), 135.5 (Ph C-1), 132.3 (Ph C-4), 129.2 (Ph C-3,5), 128.2 (Ph C-2,6), 70.7 (CH_2), 64.0 ($O\underline{C}H_2CH_3$), 15.5 ($OCH_2\underline{C}H_3$).

^{15}N NMR (40 MHz, Acetone-d_6) δ: -264.2 (NH).

HRMS (ESI), *m/z*: calcd. for $C_{10}H_{13}NNaO_2^+$: 202.0838 [M + Na]$^+$; found: 202.0850.

_N_-(ethoxymethyl)-4-methylbenzamide (38)

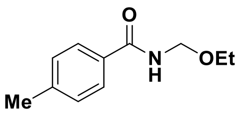

By following the general procedure, starting from *N*-ethoxy-*N*,4-dimethylbenzamide (0.193 g, 1.0 mmol, 1.0 equiv), PMDTA (0.277 g, 0.33 mL, 1.6 mmol, 1.6 equiv) and *s*-BuLi (2.2 M in Et_2O, 0.73 mL, 1.6 mmol, 1.6 equiv) in dry 2-MeTHF (3 mL), compound **38** was obtained in 75% yield (0.144 g) as a yellowish solid after purification by column chromatography on silica gel (*n*-hexane:EtOAc 7:3).

mp = 81-83 °C

^{1}H NMR (400 MHz, Acetone-d_6) δ: 8.44 (brs, 1H, NH), 7.84 (m, 2H, Ph H-2,6), 7.27 (m, 2H, Ph H-3,5), 4.83 (d, *J* = 6.6 Hz, 2H, CH_2), 3.54 (q, *J* = 7.0 Hz, 2H, $OC\underline{H}_2CH_3$), 2.37 (s, 3H, CH_3), 1.12 (t, *J* = 7.0 Hz, 3H, $OCH_2C\underline{H}_3$).

^{13}**C NMR** (100 MHz, Acetone-d$_6$) δ: 167.8 (C=O), 132.6 (Ph C-1), 142.7 (Ph C-4), 129.8 (Ph C-3,5), 128.2 (Ph C-2,6), 70.6 (CH$_2$), 64.0 (O$\underline{C}$H$_2$CH$_3$), 21.3 (CH$_3$), 15.5 (OCH$_2\underline{C}$H$_3$).

^{15}**N NMR** (40 MHz, Acetone-d$_6$) δ: -264.6 (NH).

HRMS (ESI), *m/z*: calcd. for $C_{11}H_{15}NNaO_2^+$: 216.0995 [M + Na]$^+$; found: 216.1011.

3-chloro-*N*-(ethoxymethyl)benzamide (39)

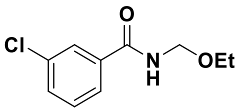

By following the general procedure, starting from 3-chloro-*N*-ethoxy-*N*-methylbenzamide (0.213 g, 1.0 mmol, 1.0 equiv), PMDTA (0.277 g, 0.33 mL, 1.6 mmol, 1.6 equiv) and *s*-BuLi (2.2 M in Et$_2$O, 0.73 mL, 1.6 mmol, 1.6 equiv) in dry 2-MeTHF (3 mL), compound **39** was obtained in 71% yield (0.151 g) as a white solid after purification by column chromatography on silica gel (*n*-hexane:EtOAc 7:3).

mp = 64-66 °C

^{1}**H NMR** (400 MHz, Acetone-d$_6$) δ: 8.65 (brs, 1H, NH), 7.94 (m, 1H, Ph H-6), 7.88 (m, 1H, Ph H-2), 7.57 (m, 1H, Ph H-4), 7.50 (m, 1H, Ph H-3), 4.85 (d, *J* = 6.6 Hz, 2H, CH$_2$), 3.56 (q, *J* = 7.0 Hz, 2H, OC$\underline{H}_2$CH$_3$), 1.12 (t, *J* = 7.0 Hz, 3H, OCH$_2$C$\underline{H}_3$).

^{13}**C NMR** (100 MHz, Acetone-d$_6$) δ: 166.6 (C=O), 137.3 (Ph C-1), 134.8 (Ph C-5), 132.2 (Ph C-4), 131.1 (Ph C-3), 128.2 (Ph C-6), 126.2 (Ph C-2), 70.7 (CH$_2$), 64.1 (O$\underline{C}$H$_2$CH$_3$), 15.5 (OCH$_2\underline{C}$H$_3$).

^{15}**N NMR** (40 MHz, Acetone-d$_6$) δ: -262.8 (NH).

HRMS (ESI), *m/z*: calcd. for $C_{10}H_{12}ClNNaO_2^+$: 236.0449 [M + Na]$^+$; found: 236.0447.

***N*-(isopropoxymethyl)benzamide (40)**

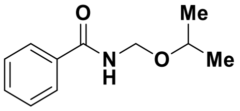

By following the general procedure, starting from *N*-isopropoxy-*N*-methylbenzamide (0.193 g, 1.0 mmol, 1.0 equiv), PMDTA (0.277 g, 0.33 mL, 1.6 mmol, 1.6 equiv) and *s*-BuLi (2.2 M in Et$_2$O, 0.73 mL, 1.6 mmol, 1.6 equiv) in dry 2-MeTHF (3 mL), compound **40** was obtained in 73% yield (0.140 g) as a colourless oil after purification by column chromatography on silica gel (*n*-hexane:EtOAc 7:3).

^{1}**H NMR** (400 MHz, Acetone-d$_6$) δ: 8.40 (brs, 1H, NH), 7.94 (m, 2H, Ph H-2,6), 7.54 (m, 1H, Ph H-4), 7.47 (m, 2H, Ph H-3,5), 4.88 (d, *J* = 6.6 Hz, 2H, CH$_2$), 3.86 (sept., *J* = 6.1 Hz, 1H, CH), 1.12 (d, *J* = 6.1 Hz, 6H, CH$_3$).

^{13}**C NMR** (100 MHz, Acetone-d$_6$) δ: 167.7 (C=O), 135.5 (Ph C-1), 132.3 (Ph C-4), 129.2 (Ph C-3,5), 128.2 (Ph C-2,6), 69.3 (CH), 68.7 (CH$_2$), 22.8 (CH$_3$).

^{15}**N NMR** (40 MHz, Acetone-d_6) δ: -263.0 (NH).

HRMS (ESI), *m/z*: calcd. for $C_{11}H_{15}NNaO_2^+$: 216.0995 $[M + Na]^+$; found: 216.0997.

N-{[(2-methyl-2-propanyl)oxy]methyl}benzamide (41)

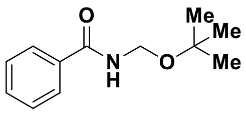

By following the general procedure, starting from *N*-(*tert*-butoxy)-*N*-methylbenzamide (0.207 g, 1.0 mmol, 1.0 equiv), PMDTA (0.277 g, 0.33 mL, 1.6 mmol, 1.6 equiv) and *s*-BuLi (2.2 M in Et_2O, 0.73 mL, 1.6 mmol, 1.6 equiv) in dry 2-MeTHF (3 mL), compound **41** was obtained in 80% yield (0.166 g) as a colourless oil after purification by column chromatography on silica gel (*n*-hexane:EtOAc 8:2).

^{1}**H NMR** (400 MHz, Acetone-d_6) δ: 8.23 (brs, 1H, NH), 7.93 (m, 2H, Ph H-2,6), 7.53 (m, 1H, Ph H-4), 7.46 (m, 2H, Ph H-3,5), 4.90 (d, *J* = 6.5 Hz, 2H, CH_2), 1.24 (s, 9H, CH_3).

^{13}**C NMR** (100 MHz, Acetone-d_6) δ: 167.1 (C=O), 135.8 (Ph C-1), 132.2 (Ph C-4), 129.2 (Ph C-3,5), 128.1 (Ph C-2,6), 73.7 ($\underline{C}(CH_3)_3$), 65.1 (CH_2), 28.4 (CH_3).

^{15}**N NMR** (40 MHz, Acetone-d_6) δ: -262.1 (NH).

HRMS (ESI), *m/z*: calcd. for $C_{12}H_{17}NNaO_2^+$: 230.1151 $[M + Na]^+$; found: 230.1154.

N-[(allyloxy)methyl]benzamide (42)

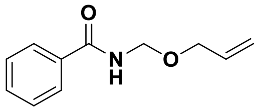

By following the general procedure, starting from *N*-(allyloxy)-*N*-methylbenzamide (0.191 g, 1.0 mmol, 1.0 equiv), PMDTA (0.277 g, 0.33 mL, 1.6 mmol, 1.6 equiv) and *s*-BuLi (2.2 M in Et_2O, 0.73 mL, 1.6 mmol, 1.6 equiv) in dry 2-MeTHF (3 mL), compound **42** was obtained in 65% yield (0.124 g) as a yellow oil after purification by column chromatography on silica gel (*n*-hexane:EtOAc 7:3).

^{1}**H NMR** (400 MHz, Acetone-d_6) δ: 8.50 (brs, 1H, NH), 7.95 (m, 2H, Ph H-2,6), 7.55 (m, 1H, Ph H-4), 7.48 (m, 2H, Ph H-3,5), 5.91 (m, 1H, $OCH_2C\underline{H}CH_2$), 5.27 (m, $^3J_{trans}$ = 17.3 Hz, 1H, $OCH_2CHC\underline{H_2}$), 5.09 (m, $^3J_{cis}$ = 10.5 Hz, 1H, $OCH_2CHC\underline{H_2}$), 4.89 (d, *J* = 6.7 Hz, 2H, CH_2), 4.07 (m, 2H, $OC\underline{H_2}CHCH_2$).

^{13}**C NMR** (100 MHz, Acetone-d_6) δ: 167.9 (C=O), 136.2 ($OCH_2\underline{C}HCH_2$), 135.4 (Ph C-1), 132.4 (Ph C-4), 129.3 (Ph C-3,5), 128.2 (Ph C-2,6), 116.2 ($OCH_2CH\underline{C}H_2$), 70.6 (CH_2), 69.6 ($O\underline{C}H_2CHCH_2$).

^{15}**N NMR** (40 MHz, Acetone-d_6) δ: -264.3 (NH).

HRMS (ESI), *m/z*: calcd. for $C_{11}H_{13}NNaO_2^+$: 214.0838 $[M + Na]^+$; found: 214.0844.

N-[(2-propyn-1-yloxy)methyl]benzamide (43)

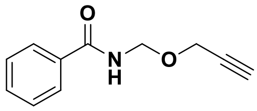

By following the general procedure, starting from *N*-methyl-*N*-(prop-2-yn-1-yloxy)benzamide (0.189 g, 1.0 mmol, 1.0 equiv), PMDTA (0.277 g, 0.33 mL, 1.6 mmol, 1.6 equiv) and *s*-BuLi (2.2 M in Et_2O, 0.73 mL, 1.6 mmol, 1.6 equiv) in dry 2-MeTHF (3 mL), compound **43** was obtained in 63% yield (0.119 g) as a colourless oil after purification by column chromatography on silica gel (*n*-hexane:EtOAc 8:2).

^{1}H NMR (400 MHz, Acetone-d_6) δ: 8.56 (brs, 1H, NH), 7.95 (m, 2H, Ph H-2,6), 7.56 (m, 1H, Ph H-4), 7.48 (m, 2H, Ph H-3,5), 4.94 (d, *J* = 6.7 Hz, 2H, CH_2), 4.24 (d, *J* = 2.4 Hz, 2H, C$\underline{H}_2$CCH), 2.90 (t, *J* = 2.4 Hz, 1H, CH_2CC$\underline{H}$).

^{13}C NMR (100 MHz, Acetone-d_6) δ: 168.0 (C=O), 135.2 (Ph C-1), 132.5 (Ph C-4), 129.3 (Ph C-3,5), 128.2 (Ph C-2,6), 81.1 ($CH_2$$\underline{C}$CH), 75.4 ($CH_2C\underline{C}$H), 70.1 ($CH_2$), 55.9 ($\underline{C}H_2$CCH).

^{15}N NMR (40 MHz, Acetone-d_6) δ: -265.7 (NH).

HRMS (ESI), *m/z*: calcd. for $C_{11}H_{11}NNaO_2^+$: 212.0682 [M + Na]$^+$; found: 212.0684.

N-[(cyclopropylmethoxy)methyl]benzamide (44)

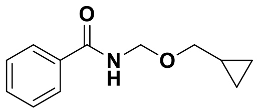

By following the general procedure, starting from *N*-(cyclopropylmethoxy)-*N*-methylbenzamide (0.205 g, 1.0 mmol, 1.0 equiv), PMDTA (0.277 g, 0.33 mL, 1.6 mmol, 1.6 equiv) and *s*-BuLi (2.2 M in Et_2O, 0.73 mL, 1.6 mmol, 1.6 equiv) in dry 2-MeTHF (3 mL), compound **44** was obtained in 70% yield (0.143 g) as a white solid after purification by column chromatography on silica gel (*n*-hexane:EtOAc 7:3).

mp = 53-55 °C

^{1}H NMR (400 MHz, Acetone-d_6) δ: 8.45 (brs, 1H, NH), 7.94 (m, 2H, Ph H-2,6), 7.54 (m, 1H, Ph H-4), 7.47 (m, 2H, Ph H-3,5), 4.88 (d, *J* = 6.6 Hz, 2H, NCH_2), 3.37 (d, *J* = 6.8 Hz, 2H, CH_2), 1.02 (m, 1H, cyclopropane H-1), 0.44 (m, 2H, cyclopropane H-2,3), 0.18 (m, 2H, cyclopropane H-2,3).

^{13}C NMR (100 MHz, Acetone-d_6) δ: 167.8 (C=O), 135.4 (Ph C-1), 132.3 (Ph C-4), 129.2 (Ph C-3,5), 128.2 (Ph C-2,6), 73.2 (CH_2), 70.7 (NCH_2), 11.3 (cyclopropane C-1), 3.2 (cyclopropane C-2,3).

^{15}N NMR (40 MHz, Acetone-d_6) δ: -263.9 (NH).

HRMS (ESI), *m/z*: calcd. for $C_{12}H_{15}NNaO_2^+$: 228.0995 [M + Na]$^+$; found: 228.0992.

N-[(methoxymethoxy)methyl]benzamide (45)

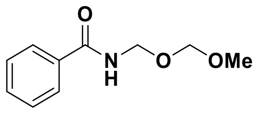

By following the general procedure, starting from *N*-(methoxymethoxy)-*N*-methylbenzamide (0.195 g, 1.0 mmol, 1.0 equiv), PMDTA (0.277 g, 0.33 mL, 1.6 mmol, 1.6 equiv) and *s*-BuLi (2.2 M in Et_2O, 0.73 mL, 1.6 mmol, 1.6 equiv) in dry 2-MeTHF (3 mL), compound **45** was obtained in 67% yield (0.130 g) as a white solid after purification by column chromatography on silica gel (*n*-hexane:EtOAc 6:4).

mp = 30-32 °C

^{1}H NMR (400 MHz, Acetone-d_6) δ: 8.56 (brs, 1H, NH), 7.95 (m, 2H, Ph H-2,6), 7.55 (m, 1H, Ph H-4), 7.47 (m, 2H, Ph H-3,5), 4.95 (d, *J* = 6.7 Hz, 2H, NCH_2), 4.71 (s, 2H C<u>H</u>$_2$OCH$_3$), 3.32 (s, 3H, CH_3).

^{13}C NMR (100 MHz, Acetone-d_6) δ: 167.9 (C=O), 135.3 (Ph C-1), 132.4 (Ph C-4), 129.3 (Ph C-3,5), 128.8 (Ph C-2,6), 95.1 (<u>C</u>H$_2$OCH$_3$), 68.3 (NCH_2), 55.4 (CH_3).

^{15}N NMR (40 MHz, Acetone-d_6) δ: -264.8 (NH).

HRMS (ESI), *m/z*: calcd. for $C_{10}H_{13}NNaO_3^+$: 218.0788 $[M + Na]^+$; found: 218.0790.

N-(phenoxymethyl)benzamide (46)

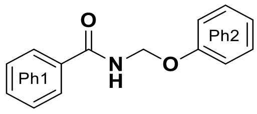

By following the general procedure, starting from *N*-methyl-*N*-phenoxybenzamide (0.227 g, 1.0 mmol, 1.0 equiv), PMDTA (0.277 g, 0.33 mL, 1.6 mmol, 1.6 equiv) and *s*-BuLi (2.2 M in Et_2O, 0.73 mL, 1.6 mmol, 1.6 equiv) in dry 2-MeTHF (3 mL), compound **46** was obtained in 74% yield (0.168 g) as a white solid after purification by column chromatography on silica gel (DCM).

mp = 110-115 °C

^{1}H NMR (400 MHz, Acetone-d_6) δ: 8.77 (brs, 1H, NH), 7.95 (m, 2H, Ph1 H-2,6), 7.56 (m, 1H, Ph1 H-4), 7.48 (m, 2H, Ph1 H-3,5), 7.28 (m, 2H, Ph2 H-3,5), 7.06 (m, 2H, Ph2 H-2,6), 6.94 (m, 1H, Ph2 H-4), 5.47 (d, *J* = 6.7 Hz, 2H, CH_2).

^{13}C NMR (100 MHz, Acetone-d_6) δ: 167.7 (C=O), 158.2 (Ph2 C-1), 135.0 (Ph1 C-1), 132.6 (Ph1 C-4), 130.3 (Ph2 C-3,5), 129.3 (Ph1 C-3,5), 128.3 (Ph1 C-2,6), 122.0 (Ph2 C-4), 116.4 (Ph2 C-2,6), 69.4 (CH_2).

^{15}N NMR (40 MHz, Acetone-d_6) δ: -266.8 (NH).

HRMS (ESI), *m/z*: calcd. for $C_{14}H_{13}NNaO_2^+$: 250.0838 $[M + Na]^+$; found: 250.0845.

***N*-[(benzyloxy)methyl]benzamide (47)**

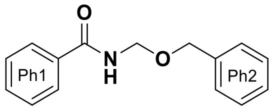

By following the general procedure, starting from *N*-(benzyloxy)-*N*-methylbenzamide (0.241 g, 1.0 mmol, 1.0 equiv), PMDTA (0.277 g, 0.33 mL, 1.6 mmol, 1.6 equiv) and *s*-BuLi (2.2 M in Et_2O, 0.73 mL, 1.6 mmol, 1.6 equiv) in dry 2-MeTHF (3 mL), compound **47** was obtained in 77% yield (0.186 g) as a white solid after purification by column chromatography on silica gel (*n*-hexane:EtOAc 7:3).

mp = 60-64 °C

^{1}H NMR (400 MHz, Acetone-d_6) δ: 8.58 (brs, 1H, NH), 7.96 (m, 2H, Ph1 H-2,6), 7.55 (m, 1H, Ph1 H-4), 7.48 (m, 2H, Ph1 H-3,5), 7.37 (m, 2H, Ph2 H-2,6), 7.32 (m, 2H, Ph2 H-3,5), 7.25 (m, 1H, Ph2 H-4), 4.98 (d, *J* = 6.6 Hz, 2H, NCH_2), 4.63 (s, 2H, C<u>H</u>$_2$Ph).

^{13}C NMR (100 MHz, Acetone-d_6) δ: 168.0 (C=O), 139.8 (Ph2 C-1), 135.4 (Ph1 C-1), 132.4 (Ph1 C-4), 129.3 (Ph1 C-3,5), 129.0 (Ph2 C-3,5), 128.4 (Ph2 C-2,6), 128.2 (Ph1 C-2,6), 128.1 (Ph2 C-4), 70.8 (NCH_2), 70.7 (<u>C</u>H$_2$Ph).

^{15}N NMR (40 MHz, Acetone-d_6) δ: -264.4 (NH).

HRMS (ESI), *m/z*: calcd. for $C_{15}H_{15}NNaO_2^+$: 264.0995 [M + Na]$^+$; found: 264.1009.

***N*-[(4-biphenylylmethoxy)methyl]benzamide (48)**

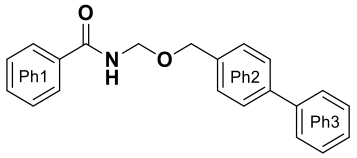

By following the general procedure, starting from *N*-([1,1'-biphenyl]-4-ylmethoxy)-*N*-methylbenzamide (0.317 g, 1.0 mmol, 1.0 equiv), PMDTA (0.277 g, 0.33 mL, 1.6 mmol, 1.6 equiv) and *s*-BuLi (2.2 M in Et_2O, 0.73 mL, 1.6 mmol, 1.6 equiv) in dry 2-MeTHF (3 mL), compound **48** was obtained in 79% yield (0.250 g) as a white solid after purification by column chromatography on silica gel (*n*-hexane:EtOAc 7:3).

mp = 134-136 °C

^{1}H NMR (400 MHz, Acetone-d_6) δ: 8.60 (brs, 1H, NH), 7.96 (m, 2H, Ph1 H-2,6), 7.64 (m, 2H, Ph3 H-2,6), 7.62 (m, 2H, Ph2 H-2,6), 7.56 (m, 1H, Ph1 H-4), 7.48 (m, 2H, Ph1 H-3,5), 7.47 (m, 2H, Ph2 H-3,5), 7.45 (m, 2H, Ph3 H-3,5), 7.35 (m, 1H, Ph3 H-4), 5.01 (d, *J* = 6.7 Hz, 2H, NCH_2), 4.69 (s, 2H, CH_2).

^{13}C NMR (100 MHz, Acetone-d_6) δ: 168.0 (C=O), 141.6 (Ph3 C-1), 140.9 (Ph2 C-1), 139.0 (Ph2 C-4), 135.4 (Ph1 C-1), 132.4 (Ph1 C-4), 129.7 (Ph3 C-3,5), 129.2 (Ph1 C-3,5), 129.0 (Ph2 C-3,5), 128.2 (Ph1 C-2,6), 128.1 (Ph3 C-4), 127.7 (Ph3 C-2,6), 127.6 (Ph2 C-2,6), 70.8 (NCH_2),70.4 (CH_2).

^{15}N NMR (40 MHz, Acetone-d_6) δ: -264.2 (NH).

HRMS (ESI), *m/z*: calcd. for $C_{21}H_{19}NNaO_2^+$: 340.1308 [M + Na]$^+$; found: 340.1306.

2-methyl-1-phenylbutan-1-one (50)

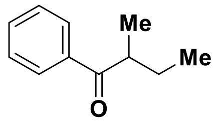

By following the general procedure, starting from *N*-ethyl-*N*-methoxybenzamide **49**[11] (0.179 g, 1.0 mmol, 1.0 equiv), PMDTA (0.277 g, 0.33 mL, 1.6 mmol, 1.6 equiv) and *s*-BuLi (2.2 M in Et_2O, 0.73 mL, 1.6 mmol, 1.6 equiv) in dry 2-MeTHF (3 mL), compound **50**[12] was obtained in quantitative yield (0.162 g) as a colorless oil which did not require purification.

^{1}H NMR (200 MHz, $CDCl_3$) δ: 7.97-7.94 (m, 2H), 7.55-7.42 (m, 3H), 3.45-3.35 (m, 1H), 1.87-1.77 (m, 1H), 1.56-1.46 (m, 1H), 1.19 (d, *J* = 6.8 Hz, 3H), 0.92 (t, *J* = 7.3 Hz, 3H).

^{13}C NMR (50 MHz, $CDCl_3$) δ: 204.1, 136.8, 132.9, 128.7, 128.3, 42.2, 26.8, 16.9, 11.9.

HRMS (ESI), *m/z*: calcd. for $C_{11}H_{14}NaO^+$: 185.0937 [M + Na]$^+$; found: 185.0932.

***N*-(methoxymethyl)-4-{[methoxy(methyl)amino]methyl}benzamine (52)**

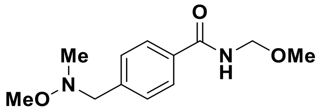

By following the general procedure, starting from *N*-methoxy-4-((methoxy(methyl)amino)methyl)-*N*-methylbenzamide (0.238 g, 1.0 mmol, 1.0 equiv), PMDTA (0.277 g, 0.33 mL, 1.6 mmol, 1.6 equiv) and *s*-BuLi (2.2 M in Et_2O, 0.73 mL, 1.6 mmol, 1.6 equiv) in dry 2-MeTHF (3 mL), compound **52** was obtained in 66% yield (0.157 g) as a white solid after purification by column chromatography on silica gel (DCM:EtOAc 6:4).

mp = 55-58 °C

^{1}H NMR (400 MHz, Acetone-d_6) δ: 8.44 (brs, 1H, NH), 7.90 (m, 2H, Ph H-2,6), 7.46 (m, 2H, Ph H-3,5), 4.80 (d, *J* = 6.6 Hz, 2H, CH_2), 3.81 (s, 2H, CH_2), 3.31 (s, 3H, $NOCH_3$), 3.30 (s, 3H, OCH_3), 2.58 (s, 3H, NCH_3).

^{13}C NMR (100 MHz, Acetone-d_6) δ: 167.8 (C=O), 143.0 (Ph C-4), 134.2 (Ph C-1), 130.1 (Ph C-3,5), 128.0 (Ph C-2,6), 72.3 (CH_2), 64.7 (CH_2), 59.8 ($NOCH_3$), 55.8 (OCH_3), 45.1 (NCH_3).

^{15}N NMR (40 MHz, Acetone-d_6) δ: -265.5 (NH), -218.8 (N).

HRMS (ESI), *m/z*: calcd. for $C_{12}H_{18}N_2NaO_3^+$: 261.1210 [M + Na]$^+$; found: 261.1222.

N-methoxy-N-($^2H_3$)methylbenzamide (53)

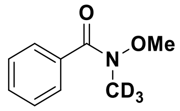

To a solution of *N*-methoxybenzamide[13] (0.200 g, 1.32 mmol, 1.0 equiv) and Cs_2CO_3 (0.873 g, 2.7 mmol, 2 equiv) in dry DMF (3 mL) Iodomethane-d_3 (0.287, 1.98 mmol, 1.5 equiv) was added and the reaction was stirred overnight at room temperature. The reaction was quenched with sat. aqueous NH_4Cl and the reaction mixture was extracted with Et_2O (3 x 10 ml). The combined organic extracts were dried over Na_2SO_4 and the solvent was removed under reduced pressure. Compound **53** was obtained without purification in 97% yield (0.212 g) as colorless oil.

^{1}H NMR (400 MHz, $CDCl_3$) δ: 7.67 (m, 2H, Ph H-2,6), 7.45 (m, 1H, Ph H-4), 7.40 (m, 2H, Ph H-3,5), 3.56 (s, 3H, OCH_3).

^{13}C NMR (100 MHz, $CDCl_3$) δ: 170.0 (C=O), 134.1 (Ph C-1), 130.5 (Ph H-4), 128.1 (Ph C-2,6), 128.0 (Ph C-3,5), 61.0 (OCH_3).

HRMS (ESI), *m/z*: calcd. for $C_9H_9D_3NO_2^+$: 169.1051 [M + H]$^+$; found: 169.1054.

N-[methoxy($^2H_2$)methyl)benzamide (54)

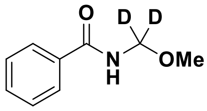

By following the general procedure, starting from *N*-methyl-*N*-[($^2H_3$)methyloxy]benzamide (compound **53**) (0.168 g, 1.0 mmol, 1.0 equiv), PMDTA (0.277 g, 0.33 mL, 1.6 mmol, 1.6 equiv) and *s*-BuLi (2.2 M in Et_2O, 0.73 mL, 1.6 mmol, 1.6 equiv) in dry 2-MeTHF (3 mL), compound **54** was obtained in 62% yield (0.104 g) as a white solid after purification by column chromatography on silica gel (*n*-hexane:EtOAc 7:3).

mp = 61-64 °C

^{1}H NMR (400 MHz, Acetone-d_6) δ: 8.44 (brs, 1H, NH), 7.94 (m, 2H, Ph H-2,6), 7.55 (m, 1H, Ph H-4), 7.48 (m, 2H, Ph H-3,5), 3.30 (s, 3H, CH_3).

^{13}C NMR (100 MHz, Acetone-d_6) δ: 167.9 (C=O), 135.4 (Ph C-1), 132.4 (Ph C-4), 129.3 (Ph C-3,5), 128.2 (Ph C-2,6), 55.8 (CH_3).

HRMS (ESI), *m/z*: calcd. for $C_9H_9D_2NNaO_2^+$: 190.0808 [M + Na]$^+$; found: 190.0807.

N-methyl-N-[($^2H_3$)methyloxy]benzamide (55)

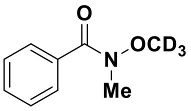

To a solution of *N*-hydroxy-*N*-methylbenzamide[6] (0.200 g, 1.32 mmol, 1.0 equiv) and Cs_2CO_3 (0.873 g, 2.68 mmol, 2 equiv) in dry DMF (3 mL) Iodomethane-d_3 (0.287g, 1.98 mmol, 2.0 equiv) was added and the reaction

was stirred overnight at room temperature. The reaction was quenched with sat. aqueous NH_4Cl and the reaction mixture was extracted with Et_2O (3 x 10 ml). The combined organic extracts were dried over Na_2SO_4 and the solvent was removed under reduced pressure. The compound **55** was obtained without purification in 95% yield (0.207 g) as colorless oil.

^{1}H NMR (400 MHz, $CDCl_3$) δ: 7.67 (m, 2H, Ph H-2,6), 7.45 (m, 1H, Ph H-4), 7.40 (m, 2H, Ph H-3,5), 3.36 (s, 3H, CH_3).

^{13}C NMR (100 MHz, $CDCl_3$) δ: 170.0 (C=O), 134.1 (Ph C-1), 130.5 (Ph H-4), 128.1 (Ph C-2,6), 128.0 (Ph C-3,5), 33.8 (CH_3).

HRMS (ESI), *m/z*: calcd. for $C_9H_8D_3NNaO_2^+$: 191.0870 $[M + Na]^+$; found: 191.0873.

N-{[($^2H_3$)methyloxy]methyl}benzamide (56)

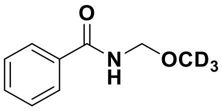

By following the general procedure, starting from *N*-methyl-*N*-[($^2H_3$)methyloxy]benzamide (compound **55**) (0.168 g, 1.0 mmol, 1.0 equiv), PMDTA (0.277 g, 0.33 mL, 1.6 mmol, 1.6 equiv) and *s*-BuLi (2.2 M in Et_2O, 0.73 mL, 1.6 mmol, 1.6 equiv) in dry 2-MeTHF (3 mL), compound **56** was obtained in 64% yield (0.107 g) as a white solid after purification by column chromatography on silica gel (*n*-hexane:EtOAc 6:4).

mp = 55-58 °C

^{1}H NMR (400 MHz, Acetone-d$_6$) δ: 8.45 (brs, 1H, NH), 7.95 (m, 2H, Ph H-2,6), 7.55 (m, 1H, Ph H-4), 7.48 (m, 2H, Ph H-3,5), 4.80 ((d, *J* = 6.6 Hz, 2H, CH_2).

^{13}C NMR (100 MHz, Acetone-d$_6$) δ: 167.9 (C=O), 135.4 (Ph C-1), 132.4 (Ph C-4), 129.3 (Ph C-3,5), 128.2 (Ph C-2,6), 72.2 (CH_2).

^{15}N NMR (40 MHz, Acetone-d$_6$) δ: -265.3 (NH).

HRMS (ESI), *m/z*: calcd. for $C_9H_9D_2NNaO_2^+$: 190.0808 $[M + Na]^+$; found: 190.0806.

N-(methoxymethyl)-4-biphenylcarboxamide (57)

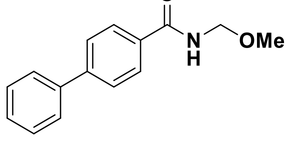

N-(methoxymethyl)-4-(tributylstannyl)benzamide **34** (0.60 mmol, 0.278 g, 1.0 equiv) was dissolved in dry DMF (5 mL) and to this solution were progressively added bromobenzene (0.60 mmol, 0.09 g, 0.06 mL, 1.0 equiv), $PdCl_2$ (0.01 mmol, 0.002 g, 0.02 equiv), P(*t*-Bu)$_3$ (0.03 mmol, 0.006 g, 0.007 mL, 0.05 equiv), CuI (0.024 mmol, 0.005 g, 0.04 equiv) and CsF (1.2 mmol, 0.182 mg, 2.0 equiv).[14] The mixture was heated at 60 °C for 24 h and, after cooling to rt, diethyl ether (10 mL) was added before flushing upon a plug of Celite. Water (1 mL) was added and the organic phase was rapidly extracted, dried over anhydrous sodium sulfate and the

solvent was removed under reduced pressure. The obtained crude was purified on silica gel using *n*-hexane: ethyl acetate (6:4 *v*/*v*) as the mobile phase, giving compound **57** in 65% yield (0.094 g) as a white solid.

mp = 134 °C

^{1}H NMR (400 MHz, Acetone-d$_6$) δ: 8.52 (brs, 1H, NH), 8.05 (m, 2H, Ph H-3,5), 7.78 (m, 2H, Ph H-2,6), 7.72 (m, 2H, Ph H-2',6'), 7.50 (m, 2H, Ph H-3',5'), 7.41 (m, 1H, Ph H-4'), 4.83 (d, *J* = 6.6 Hz, 2H, CH$_2$), 3.32 (s, 3H, OCH$_3$).

^{13}C NMR (100 MHz, Acetone-d$_6$) δ: 167.6 (C=O), 144.9 (Ph C-1), 140.8 (Ph C-1'), 134.2 (Ph C-4), 129.9 (Ph C-3',5'), 128.88 (Ph C-4'), 128.86 (Ph C-3,5), 127.9 (Ph C-2',6'), 127.7 (Ph C-2,6), 72.3 (CH$_2$), 55.9 (OCH$_3$).

^{15}N NMR (40 MHz, Acetone-d$_6$) δ: -265.3 (NH).

HRMS (ESI), *m/z*: calcd. for $C_{15}H_{15}NNaO_2^+$: 264.0995 [M + Na]$^+$; found: 264.0989.

N-(methoxymethyl)-4-(4-morpholinyl)benzamide (58)

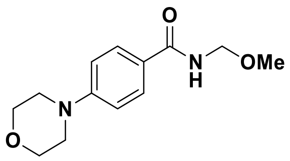

To the solution of 4-hydroxy-*N*-(methoxymethyl)benzamide (**28**, 0.77 mmol, 0.139 g, 1.0 equiv) in DCM (2mL), TsCl (0.08 mmol, 0.015 g, 1.05 equiv) and NEt$_3$ (0.92 mmol, 0.093 g, 0.13 mL,1.2 equiv) was added at rt and stirred for 3 h. After that the reaction was quenched with water and the organic phase extracted, dried over anhydrous sodium sulfate and the solvent was removed under reduced pressure. The so obtained intermediate *O*-Ts was directly used in the following step. Thus, it was dissolved in dry *t*-BuOH (5 mL) and consecutively were added Pd(OAc)$_2$ (0.039 mmol, 0.009 g, 0.05 equiv), K$_2$CO$_3$ (1.9 mmol, 0.262, 2.5 equiv) and morpholine (1.2 mmol, 0.104 g, 0.1 mL, 1.6 equiv). After degassing with Ar (x 3) the resulting mixture was heated at 90 °C for 24 h and then cooled at rt and diluted with diethyl ether (10 mL). The mixture was flushed upon a plug of Celite and washed with water (3 mL). The extracted organic phase was dried over anhydrous sodium sulfate and the solvent was removed under reduced pressure. The obtained crude was purified on silica gel using DCM:MeOH (98:2 *v*/*v*) as the mobile phase, giving compound **58** in 74% yield (0.142 g) as a yellow solid.

mp = 89-91 °C

^{1}H NMR (400 MHz, Acetone-d$_6$) δ: 8.22 (brs, 1H, NH), 7.86 (m, 2H, Ph H-2,6), 6.99 (m, 2H, Ph H-3,5), 4.77 (d, *J* = 6.7 Hz, 2H, CH$_2$), 3.78 (m, 4H, morpholine H-2,6), 3.27 (s, 3H, OCH$_3$), 3.26 (m, 4H, morpholine H-3,5).

^{13}C NMR (100 MHz, Acetone-d$_6$) δ: 167.5 (C=O), 154.6 (Ph C-4), 129.6 (Ph H-2,6), 125.1 (Ph C-1), 114.6 (Ph C-3,5), 72.2 (CH$_2$), 67.2 (morpholine C-2,6), 55.4 (OCH$_3$), 48.7 (morpholine C-3,5).

^{15}N NMR (40 MHz, Acetone-d$_6$) δ: -312.7 (N), -268.0 (NH).

HRMS (ESI), *m/z*: calcd. for $C_{13}H_{18}N_2NaO_3^+$: 273.1210 [M + Na]$^+$; found: 273.1215.

(2*E*)-*N*-(methoxymethyl)-4-methyl-3-phenyl-2-hexenamide (60)

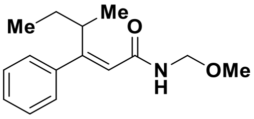

By following the general procedure, starting from *N*-methoxy-*N*-methyl-3-phenylpropiolamide (**59**, 0.189 g, 1.0 mmol, 1.0 equiv), PMDTA (0.554 g, 0.66 mL, 3.2 mmol, 3.2 equiv) and *s*-BuLi (2.2 M in Et_2O, 1.46 mL, 3.2 mmol, 3.2 equiv) in dry 2-MeTHF (3 mL), compound **60** was obtained in 61% yield (0.151 g) as a yellow solid after purification by column chromatography on silica gel (*n*-hexane:EtOAc 7:3).

mp = 52-55 °C

^{1}H NMR (400 MHz, C_6D_6) δ: 7.17 (m, 4H, Ph H-2,3,5,6), 7.07 (m, 1H, Ph H-4), 6.65 (d, *J* = 2.4 Hz, 1H, C=CH), 5.77 (brs, 1H, NH), 4.57 (d, *J* = 6.9 Hz, 2H, C<u>H</u>$_2$OCH$_3$), 3.22 (s, 3H, OCH$_3$), 2.85 (m, 1H, C<u>H</u>CH$_3$), 1.90 (m, 1H, CH$_2$), 1.58 (m, 1H, CH$_2$), 1.34 (d, *J* = 6.9 Hz, 3H, CH$_3$), 0.82 (t, *J* = 7.4 Hz, 3H, CH$_3$).

^{13}C NMR (100 MHz, C_6D_6) δ: 170.2 (C=O), 145.1 (<u>C</u>=CH), 131.0 (C=<u>C</u>H), 131.0 (Ph C-1), 129.1 (Ph C-2,6), 128.7 (Ph C-3,5), 127.7 (Ph C-4), 71.2 (<u>C</u>H$_2$OCH$_3$), 55.9 (OCH$_3$), 36.0 (<u>C</u>HCH$_3$), 28.5 (<u>C</u>H$_2$CH$_3$), 20.0 (CH<u>C</u>H$_3$), 12.9 (CH$_2$<u>C</u>H$_3$).

^{15}N NMR (40 MHz, C_6D_6) δ: -260.3 (NH).

HRMS (ESI), *m/z*: calcd. for $C_{15}H_{22}NO_2^+$: 248.1645 $[M + H]^+$; found: 248.1652.

N-(methoxymethyl)-N,3-dimethylbenzamide (61)

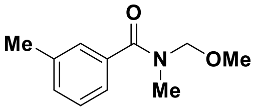

To the solution of *N*-(methoxymethyl)-3-methylbenzamide **14** (0.66 mmol, 0118 g, 1 equiv) and Cs_2CO_3 (0.86 mmol, 0.280 g, 1.3 equiv) in MeCN (2 mL), MeI (0.79 mmol, 0.112 g, 0.05 mL, 1.2 equiv) was added and reaction was stirred overnight before quenching with $NH_4Cl_{(aq.)}$. The reaction mixture was extracted 3x with EtOAc, dried over Na_2SO_4 and the solvent was removed under reduced pressure. Compound **561** was obtained in 95% (0.121 g) as yellow oil.

^{1}H NMR (400 MHz, Acetone-d$_6$) δ: 7.31-7.24 (m, 4H, Ph), 4.61 (brs, 2H, CH$_2$), 3.16 (brs, 3H, OCH$_3$), 3.02 (brs, 3H, CH$_3$), 2.82 (s, 3H, Ar-CH$_3$).

^{13}C NMR (100 MHz, Acetone-d$_6$) δ: 172.5 (C=O), 138.8 (Ph C-3), 135.4 (Ph C-1), 133.0 (Ph C-4), 129.2 (Ph C-5), 128.8 (Ph C-2), 125.3 (Ph C-6), 83.1 (CH$_2$), 55.2 (OCH$_3$), 21.3 (Ar-CH$_3$). The *N*-*C*H$_3$ signal is overlapped with the solvent residual peak at *ca.* 29.80 ppm.

HRMS (ESI), *m/z*: calcd. for $C_{11}H_{16}NO_2^+$: 194.1176 $[M + Na]^+$; found: 194.1156.

Copies of NMR spectra

Copies of ^{1}H and ^{13}C-NMR spectra of *N*-alkyl-*N*-alkoxyamides used in Schemes 3 and 4 of the manuscript.

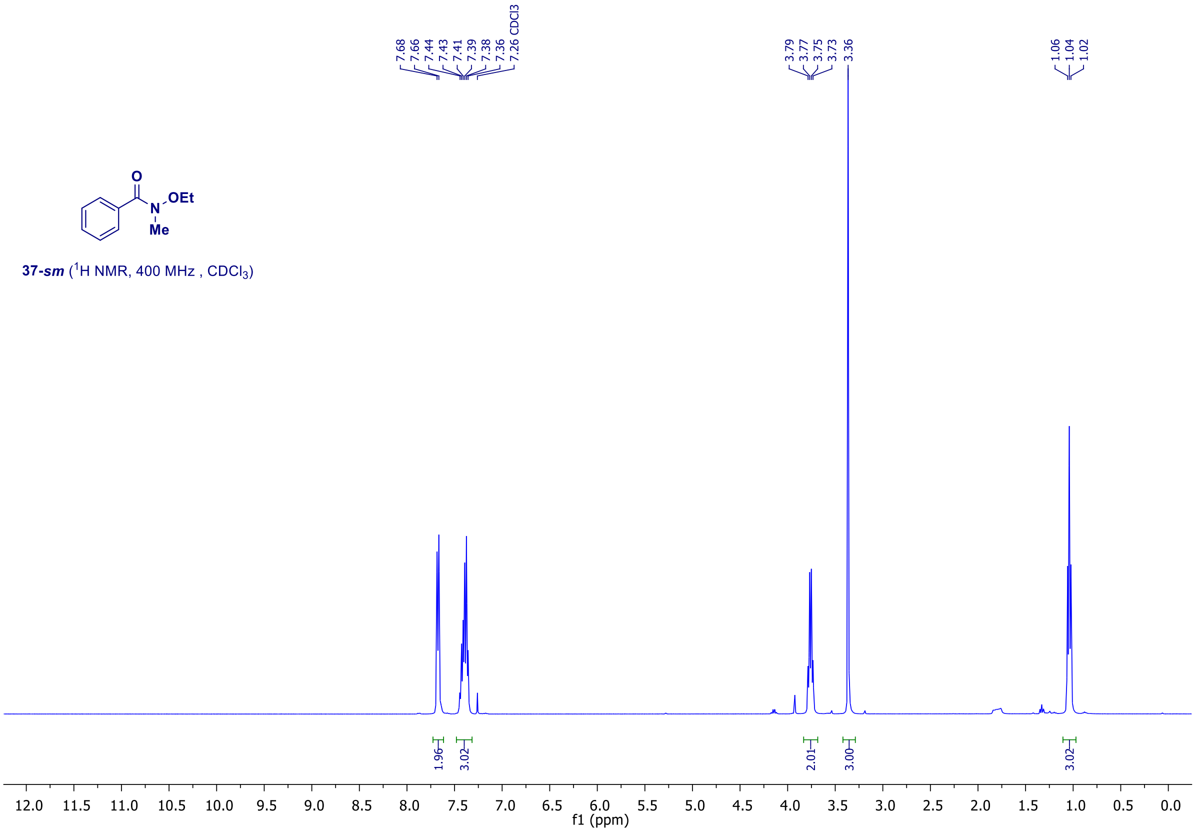

37-*sm* (^{1}H NMR, 400 MHz , $CDCl_3$)

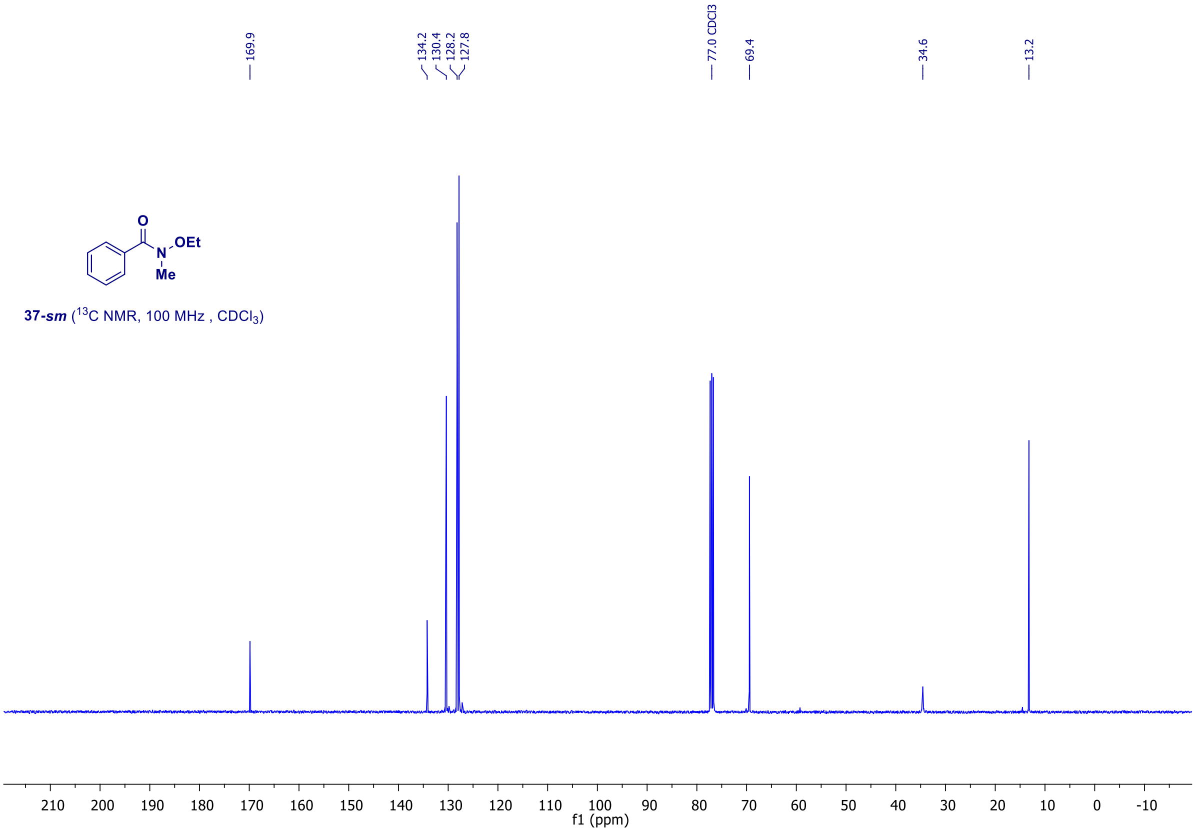

37-*sm* (^{13}C NMR, 100 MHz , $CDCl_3$)

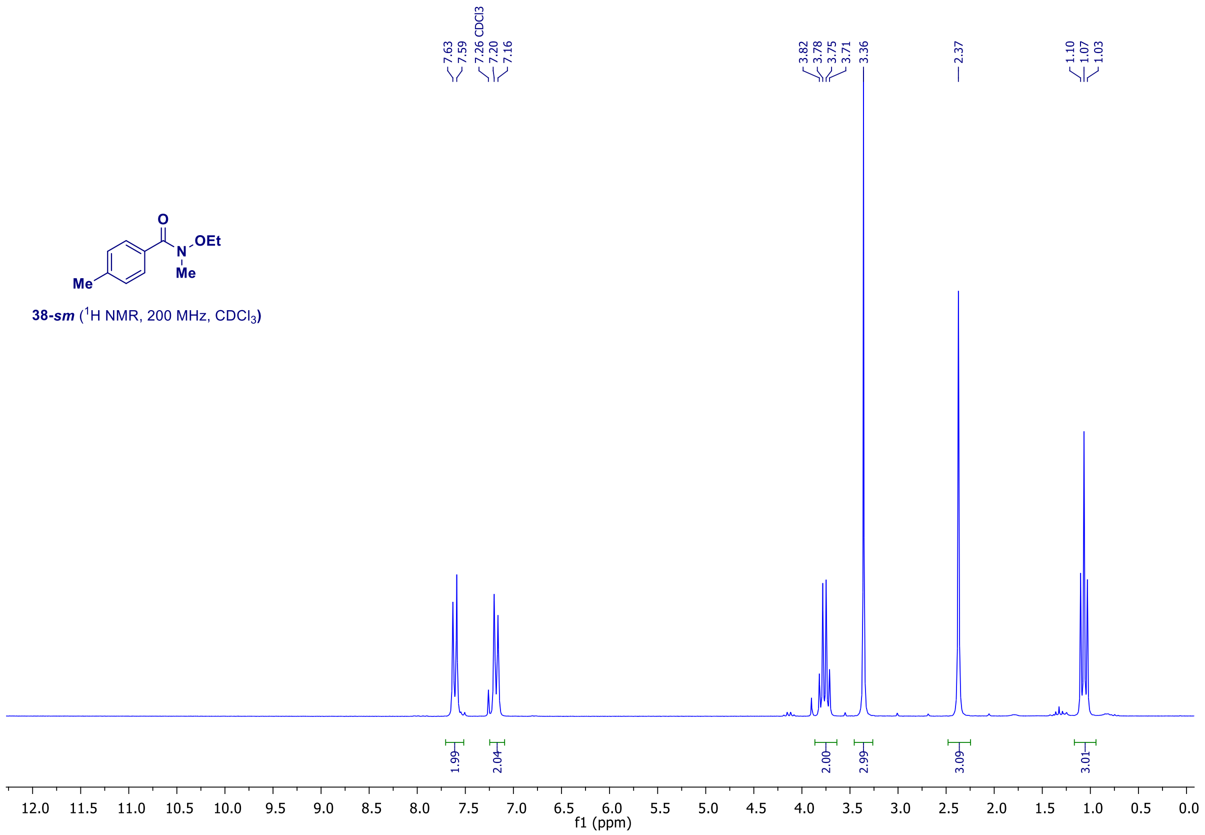
7.63
7.59
7.26 CDCl3
7.20
7.16
3.82
3.78
3.75
3.71
3.36
2.37
1.10
1.07
1.03
O
N
OEt
Me
Me
38-sm (1H NMR, 200 MHz, CDCl3)
1.99
2.04
2.00
2.99
3.09
3.01
f1 (ppm)

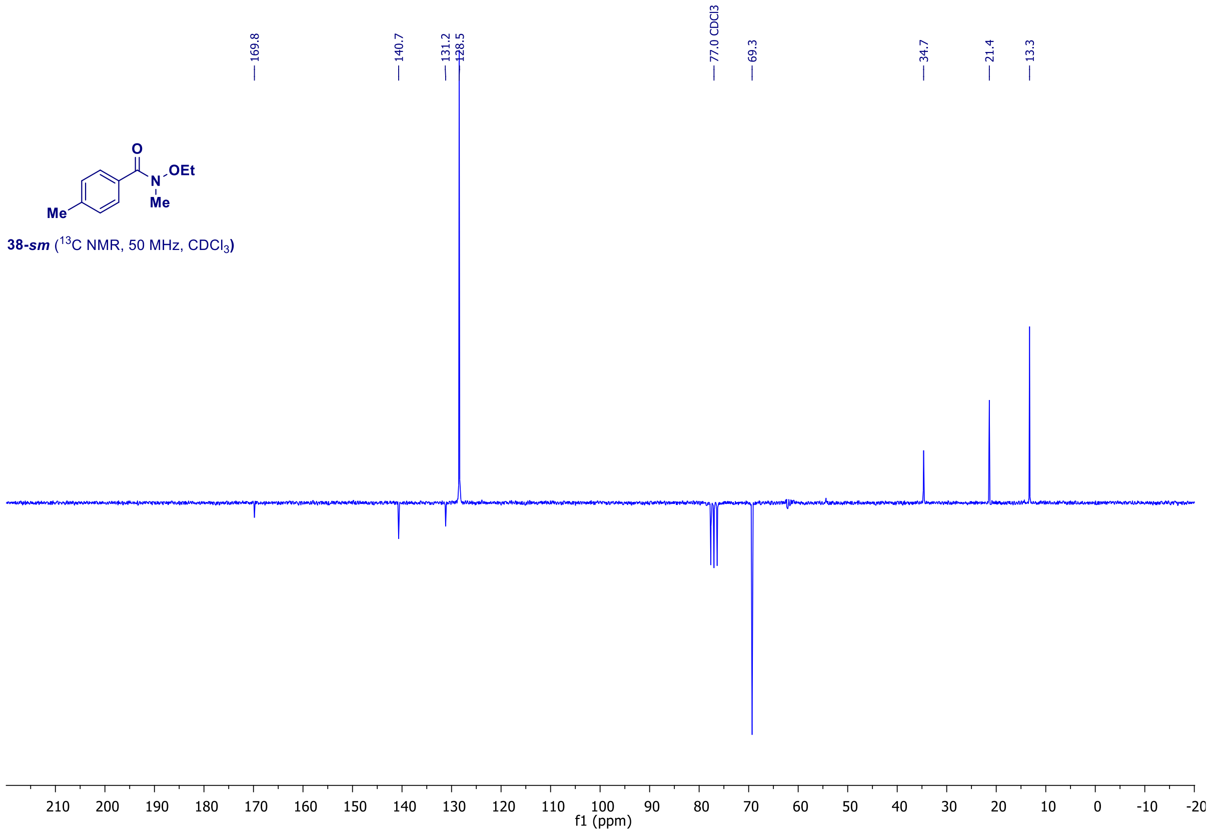
169.8
140.7
131.2
128.5
77.0 CDCl3
69.3
34.7
21.4
13.3
O
N
OEt
Me
Me
38-sm (13C NMR, 50 MHz, CDCl3)
f1 (ppm)

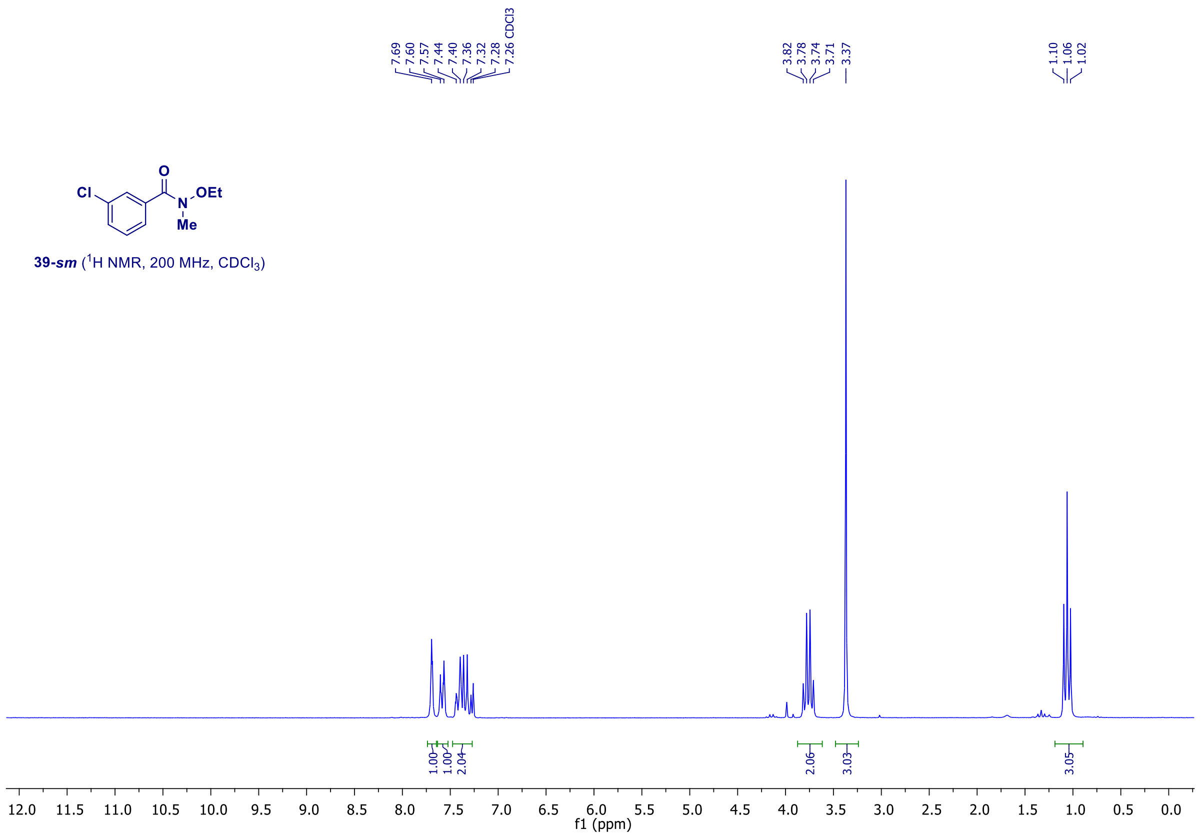

7.69
7.60
7.57
7.44
7.40
7.36
7.32
7.28
7.26 CDCl3
3.82
3.78
3.74
3.71
3.37
1.10
1.06
1.02
Cl
O
N
OEt
Me
39-sm (1H NMR, 200 MHz, CDCl3)
1.00
1.00
2.04
2.06
3.03
3.05
f1 (ppm)

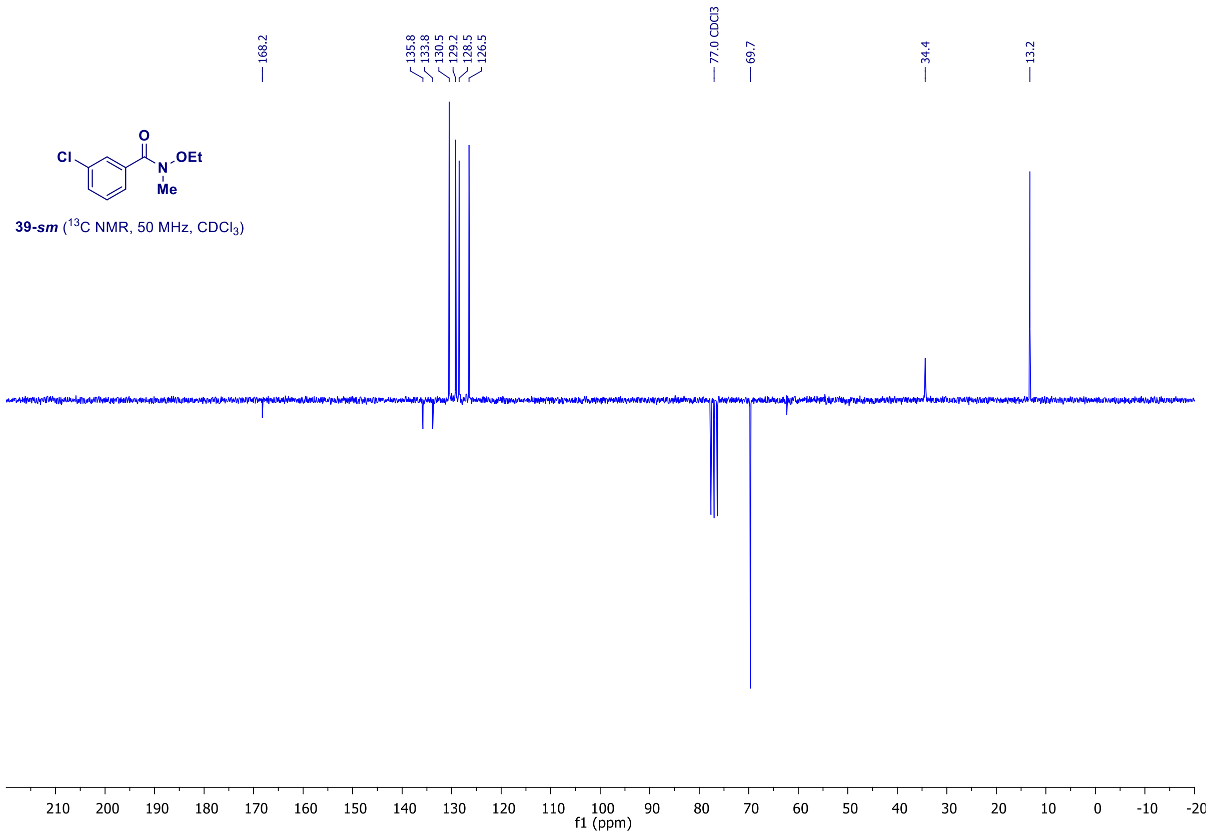

168.2
135.8
133.8
130.5
129.2
128.5
126.5
77.0 CDCl3
69.7
34.4
13.2
Cl
O
N
OEt
Me
39-sm (13C NMR, 50 MHz, CDCl3)
f1 (ppm)

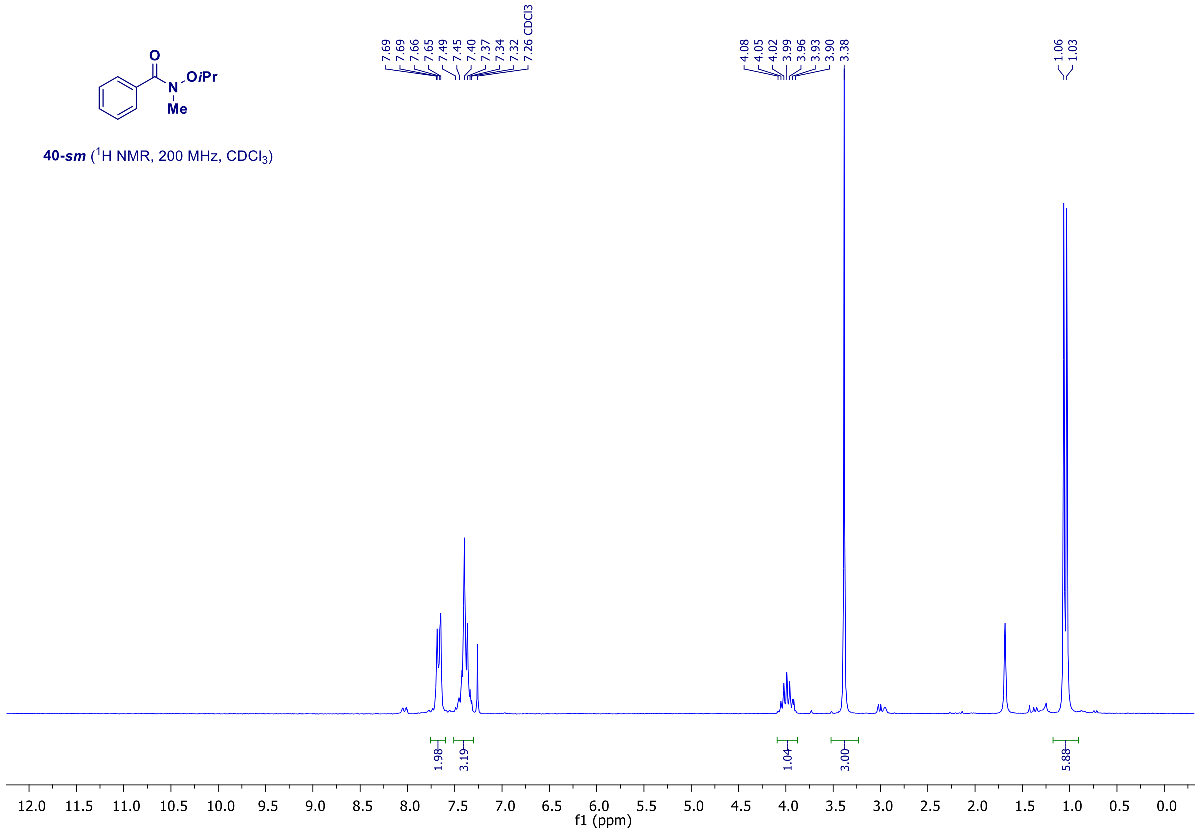

40-*sm* (^{1}H NMR, 200 MHz, $CDCl_3$)

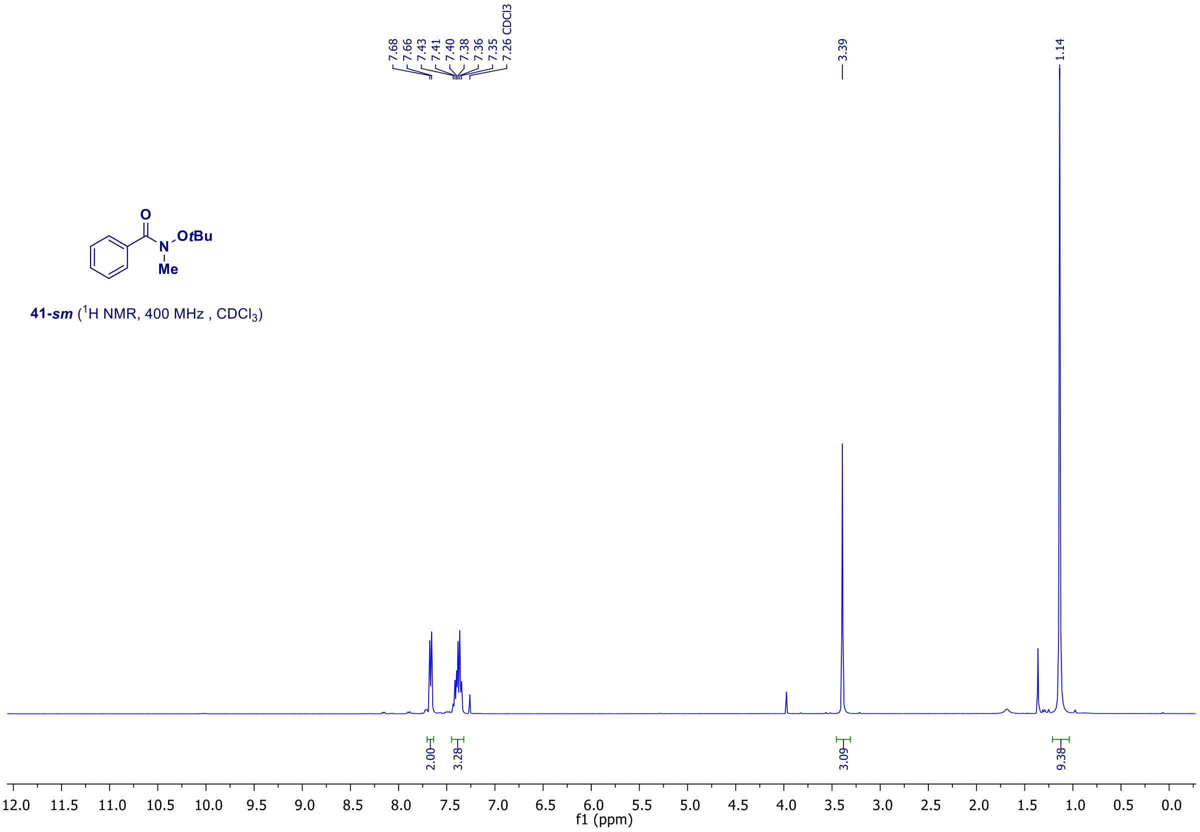

41-*sm* (^{1}H NMR, 400 MHz , $CDCl_3$)

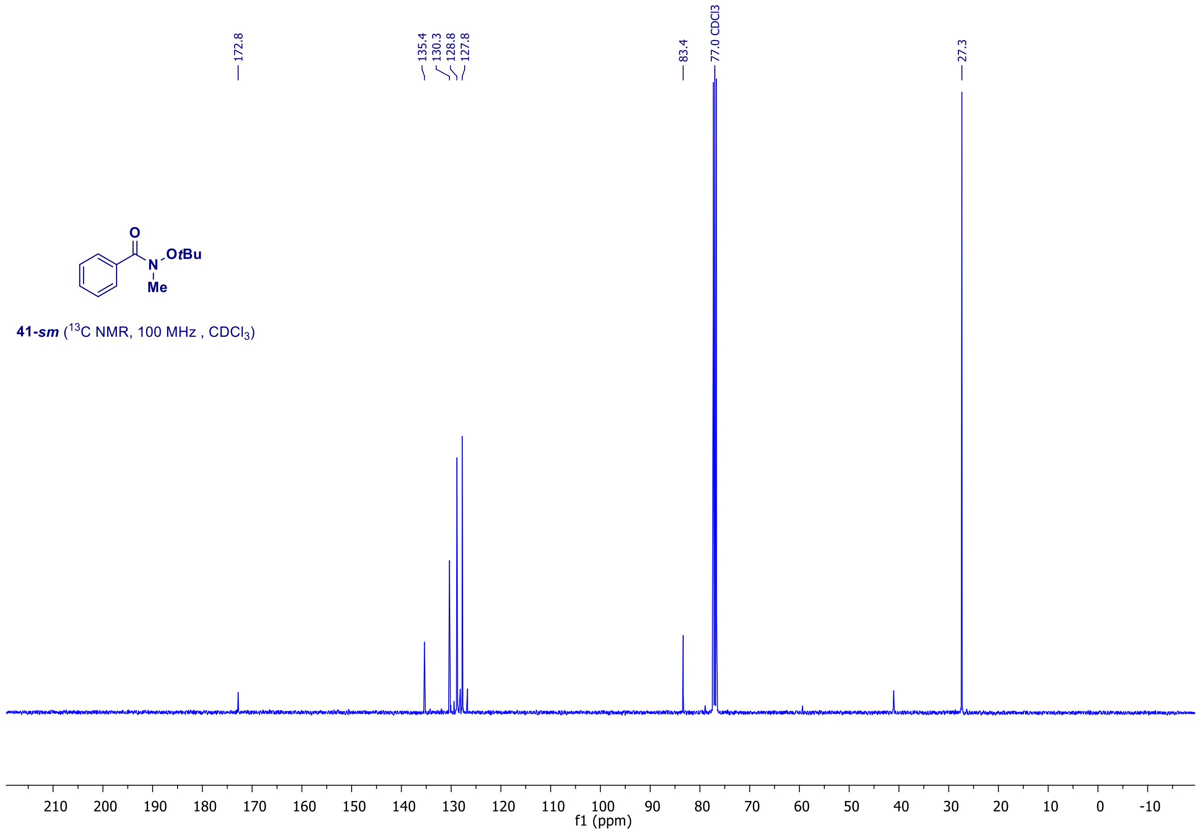

41-*sm* (^{13}C NMR, 100 MHz , $CDCl_3$)

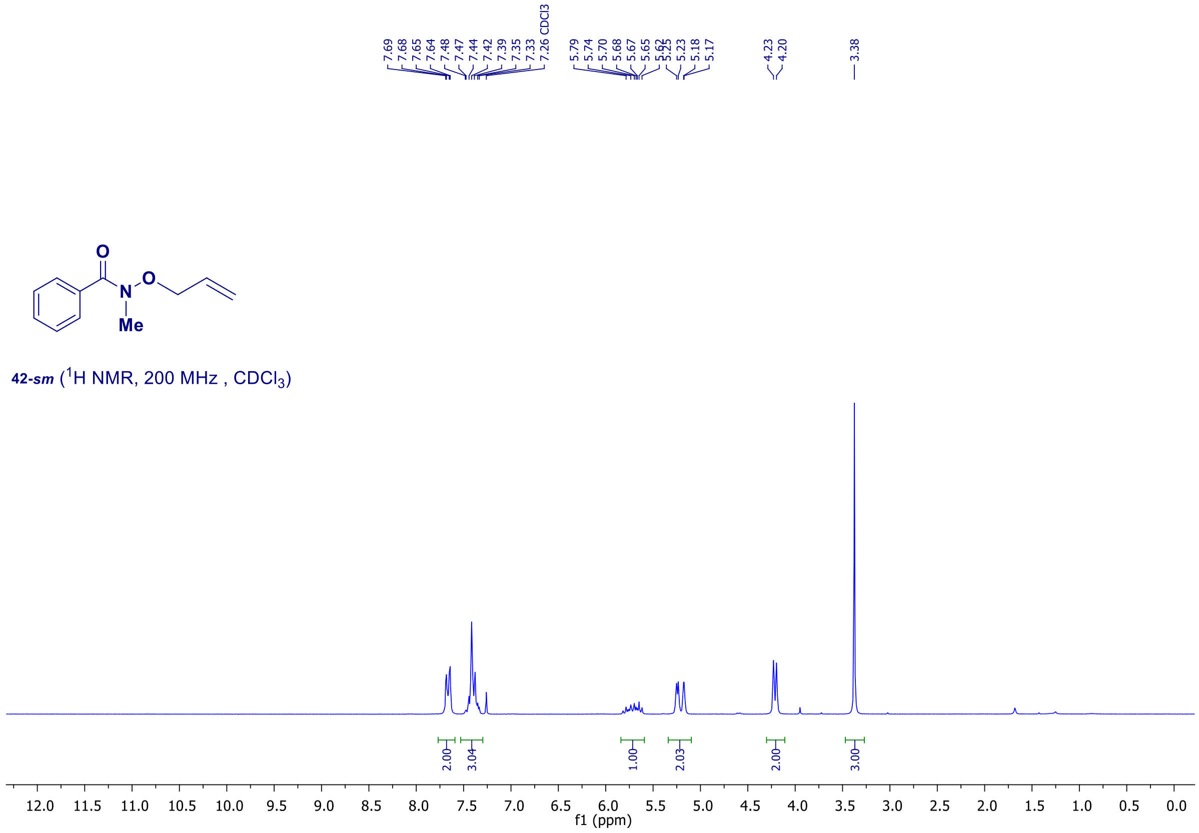

42-*sm* (^{1}H NMR, 200 MHz , $CDCl_3$)

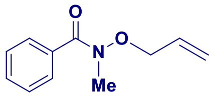

42-*sm* (^{13}C NMR, 50 MHz , $CDCl_3$)

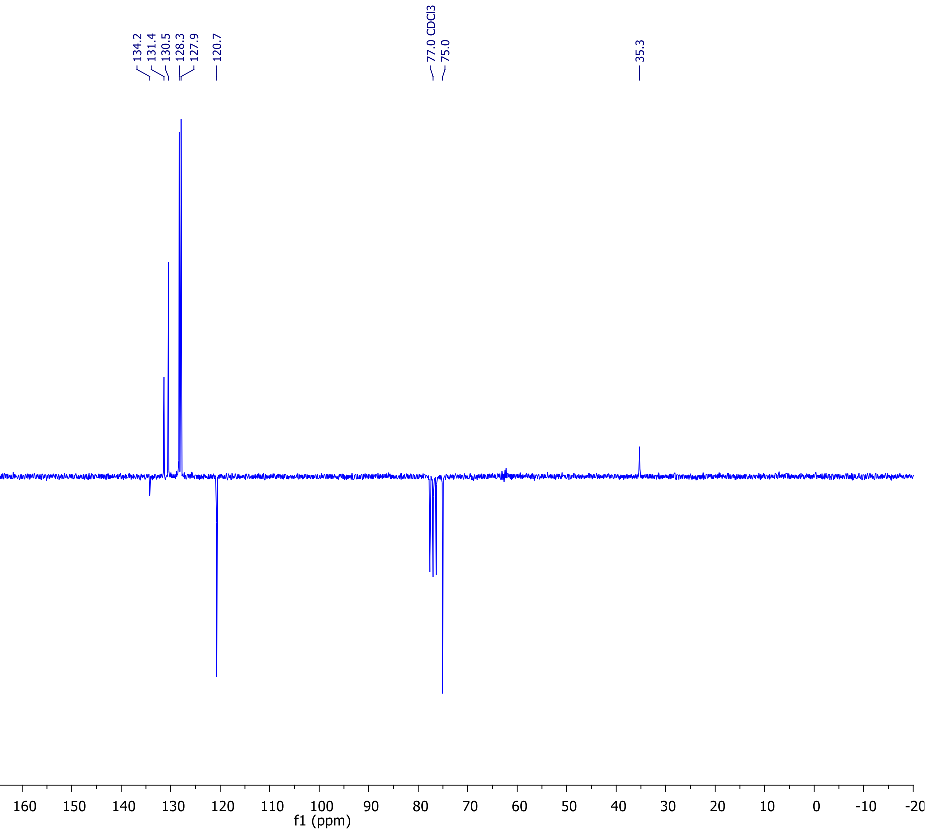

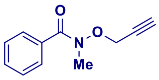

43-*sm* (^{1}H NMR, 200 MHz, $CDCl_3$)

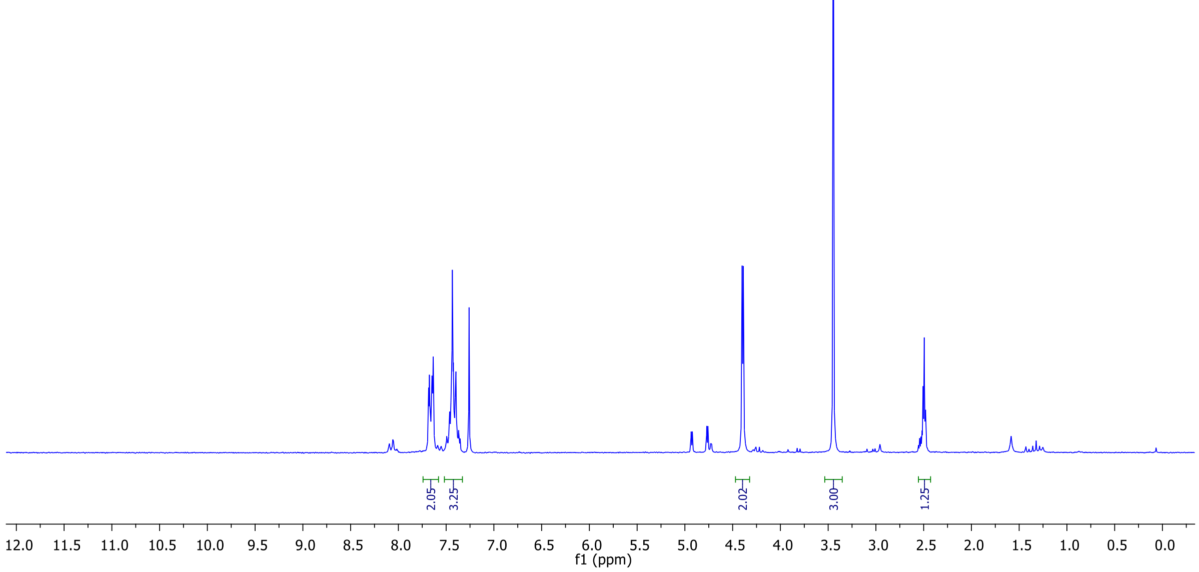

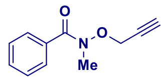

43-*sm* (^{13}C NMR, 50 MHz, $CDCl_3$)

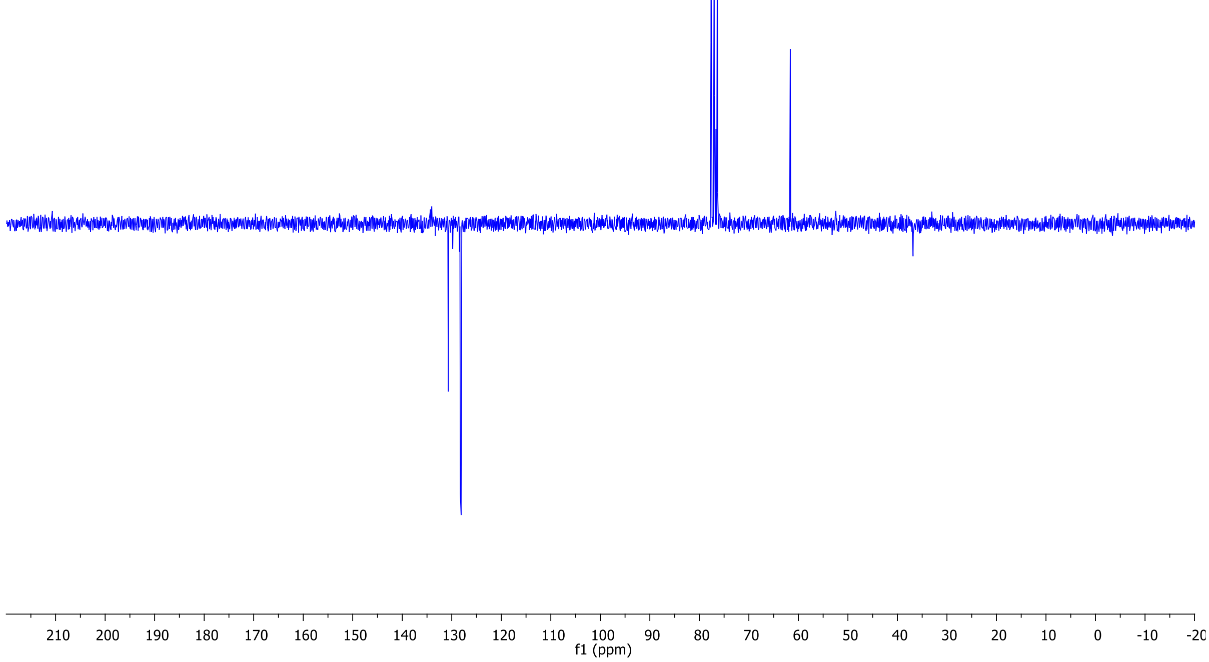

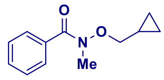

44-*sm* (^{1}H NMR, 200 MHz, $CDCl_3$)

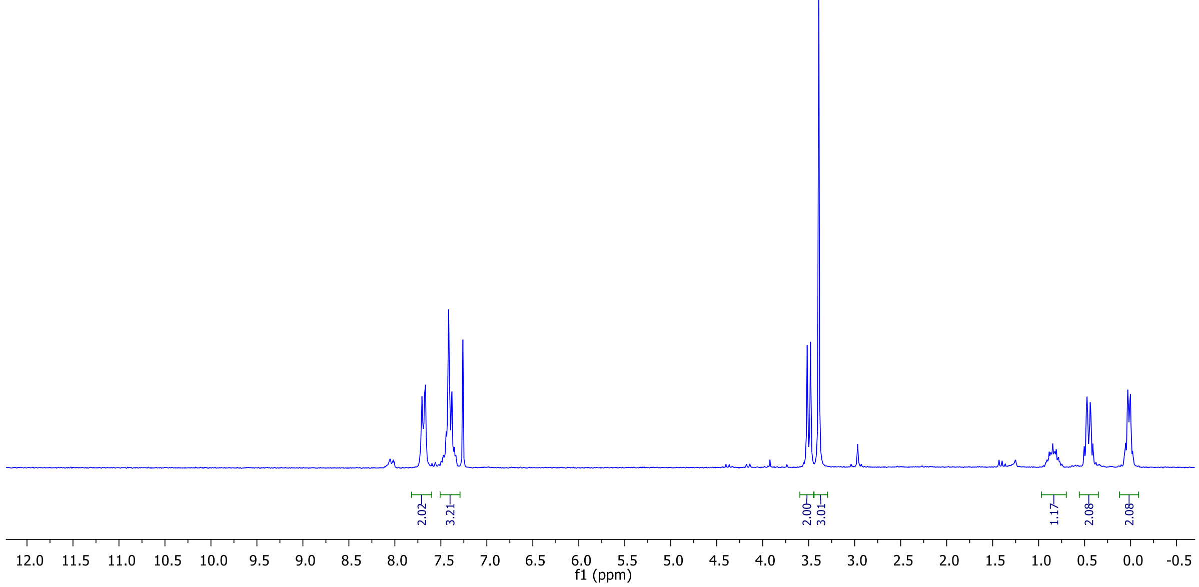

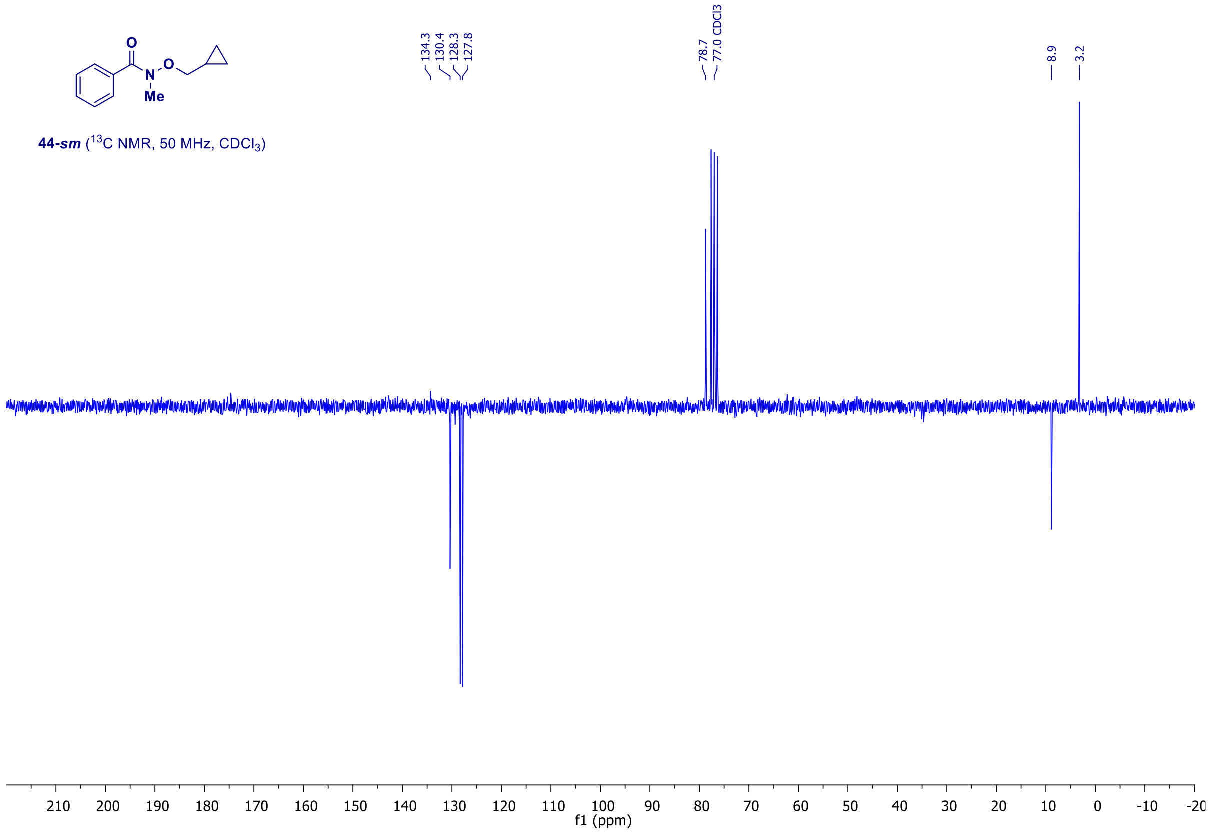

44-*sm* (^{13}C NMR, 50 MHz, $CDCl_3$)

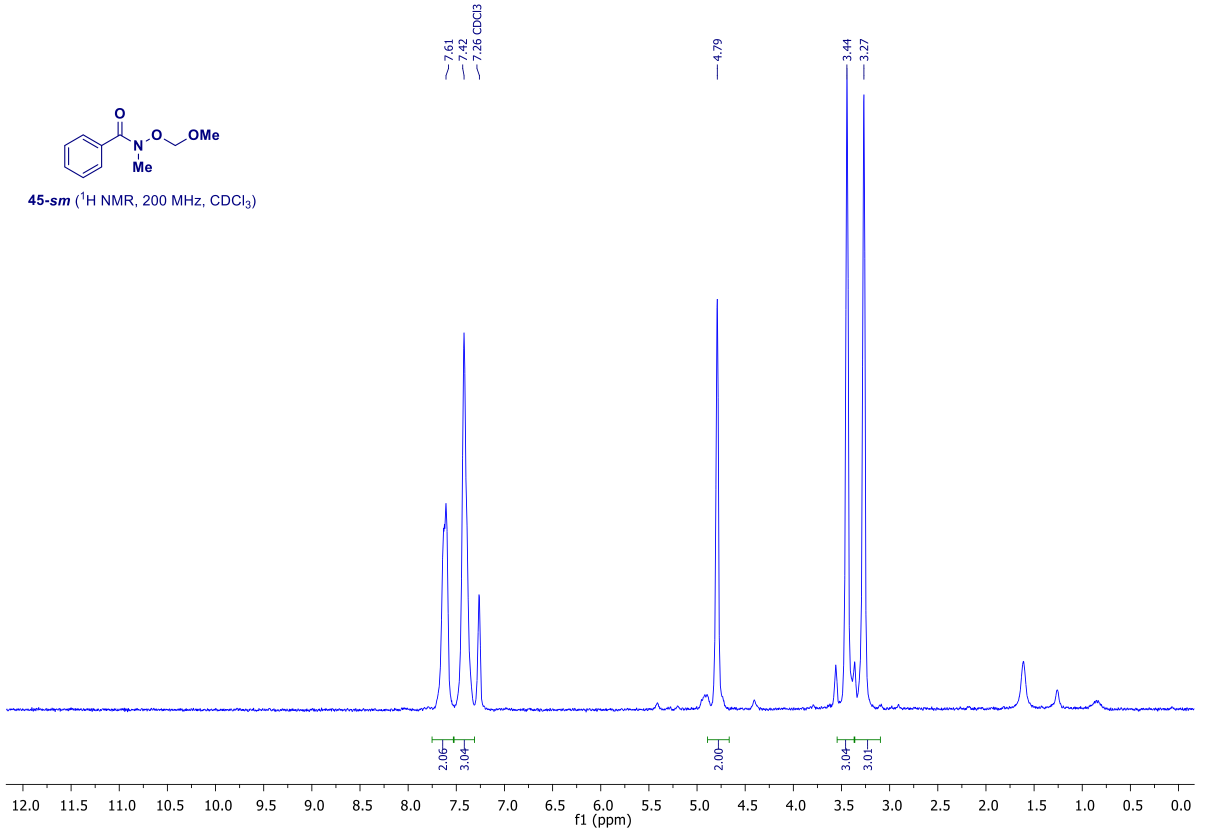

45-*sm* (^{1}H NMR, 200 MHz, $CDCl_3$)

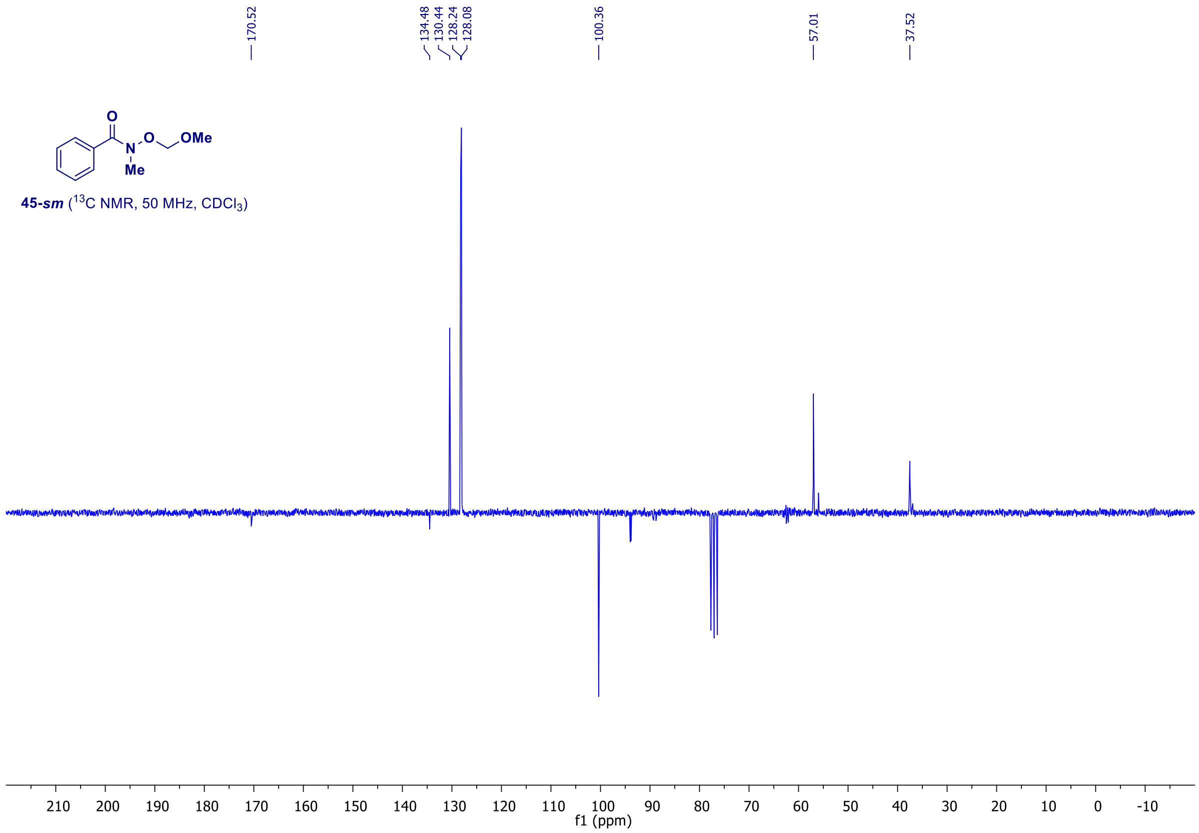
170.52
134.48
130.44
128.24
128.08
100.36
57.01
37.52
O
N
O
OMe
Me
45-*sm* (^{13}C NMR, 50 MHz, $CDCl_3$)
210 200 190 180 170 160 150 140 130 120 110 100 90 80 70 60 50 40 30 20 10 0 -10
f1 (ppm)

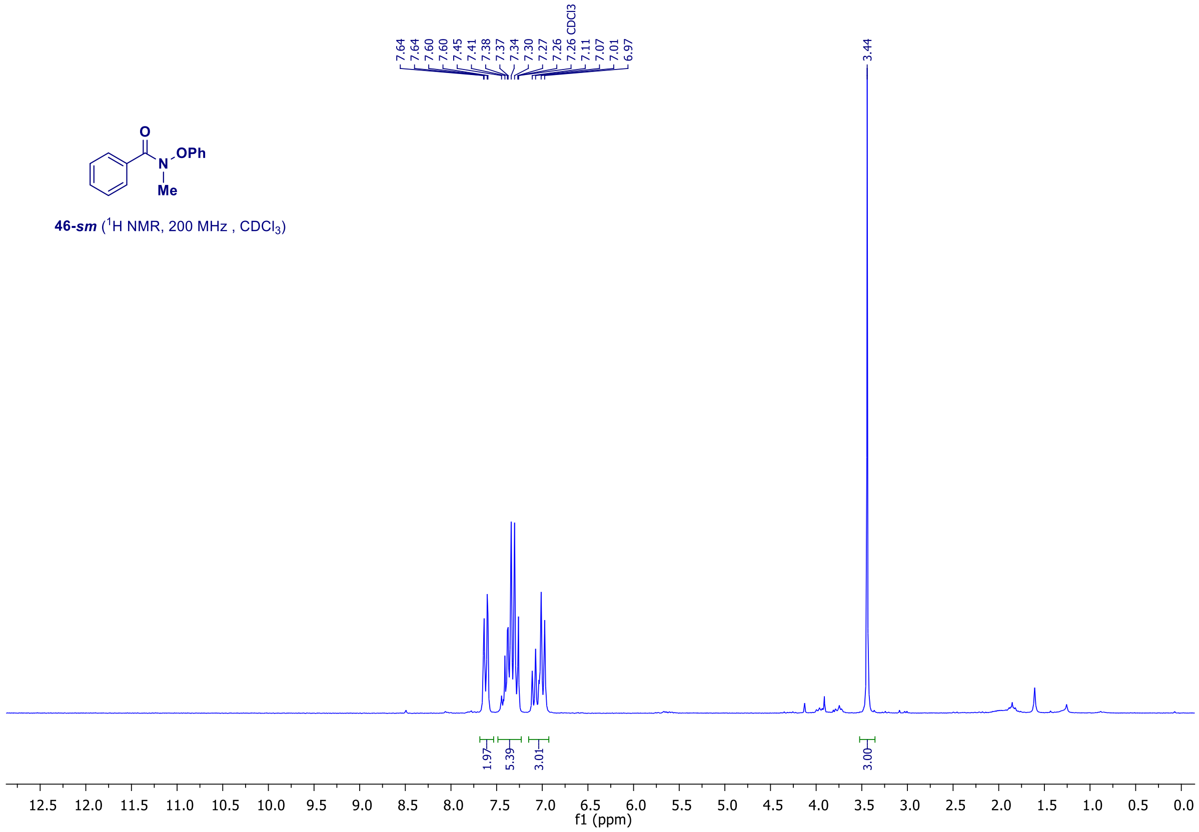
7.64
7.64
7.60
7.60
7.45
7.41
7.38
7.37
7.34
7.30
7.27
7.26 CDCl3
7.11
7.07
7.01
6.97
3.44
O
N
OPh
Me
46-*sm* (^{1}H NMR, 200 MHz , $CDCl_3$)
1.97
5.39
3.01
3.00
12.5 12.0 11.5 11.0 10.5 10.0 9.5 9.0 8.5 8.0 7.5 7.0 6.5 6.0 5.5 5.0 4.5 4.0 3.5 3.0 2.5 2.0 1.5 1.0 0.5 0.0
f1 (ppm)

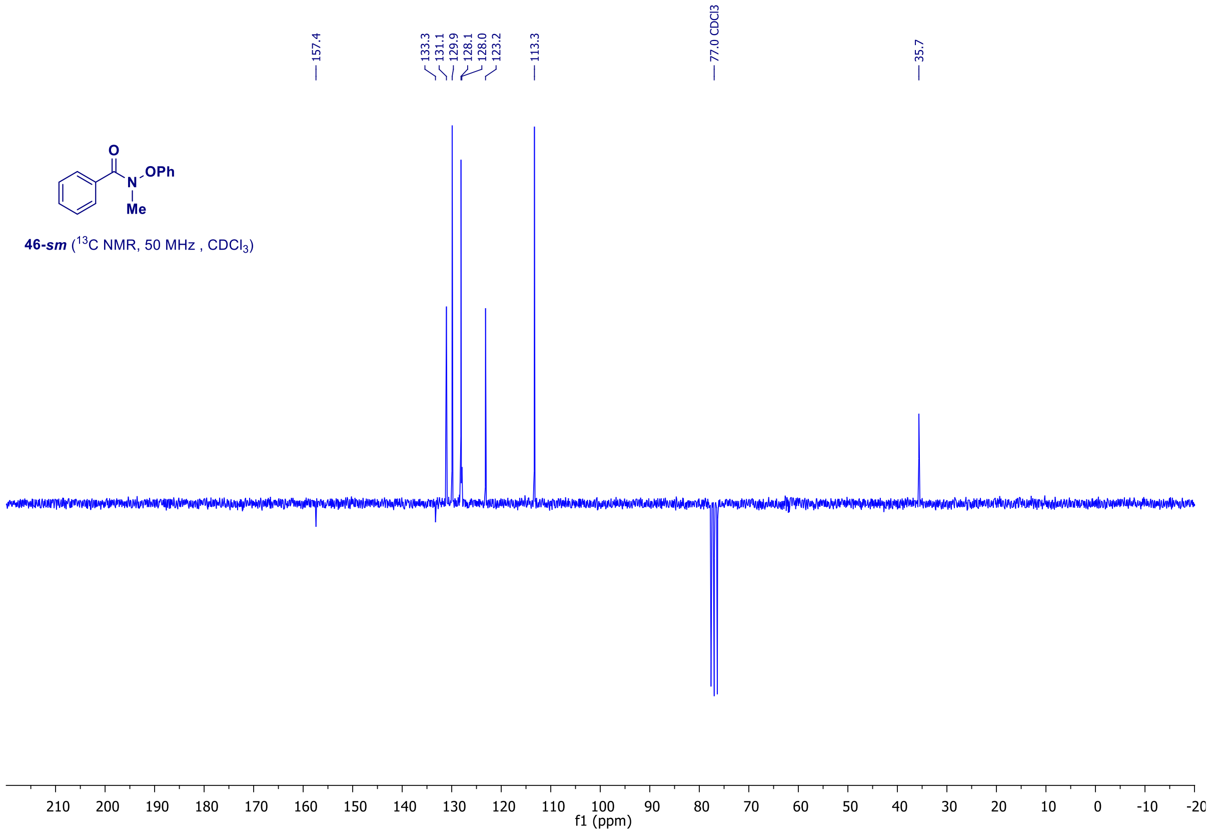

46-*sm* (^{13}C NMR, 50 MHz , $CDCl_3$)

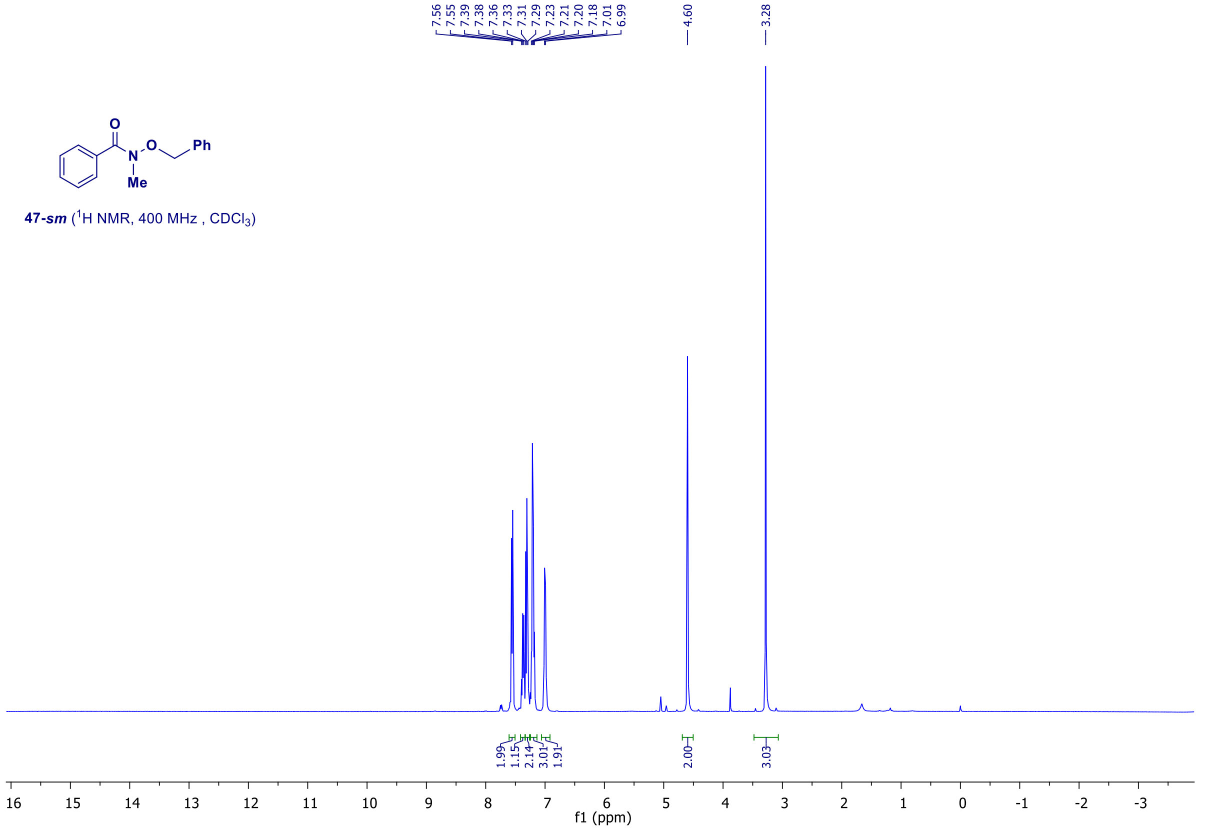

47-*sm* (^{1}H NMR, 400 MHz , $CDCl_3$)

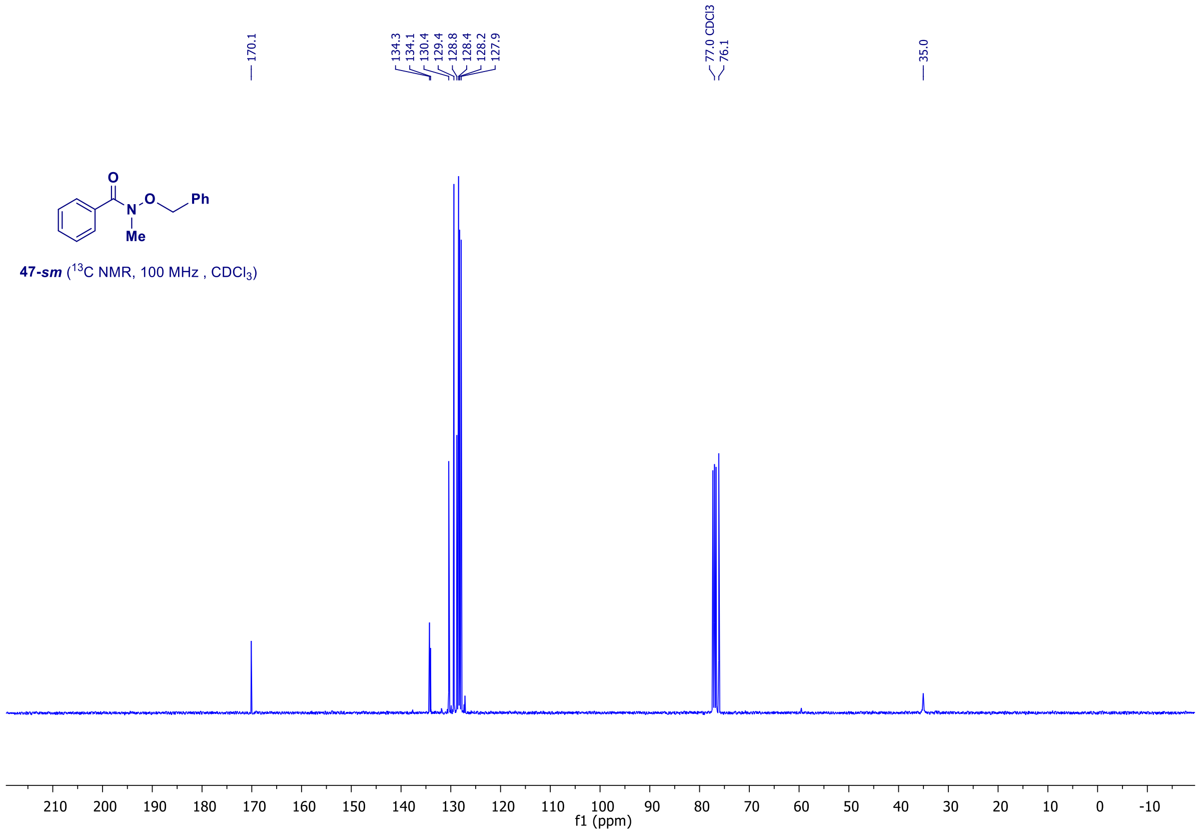

47-*sm* (^{13}C NMR, 100 MHz , $CDCl_3$)

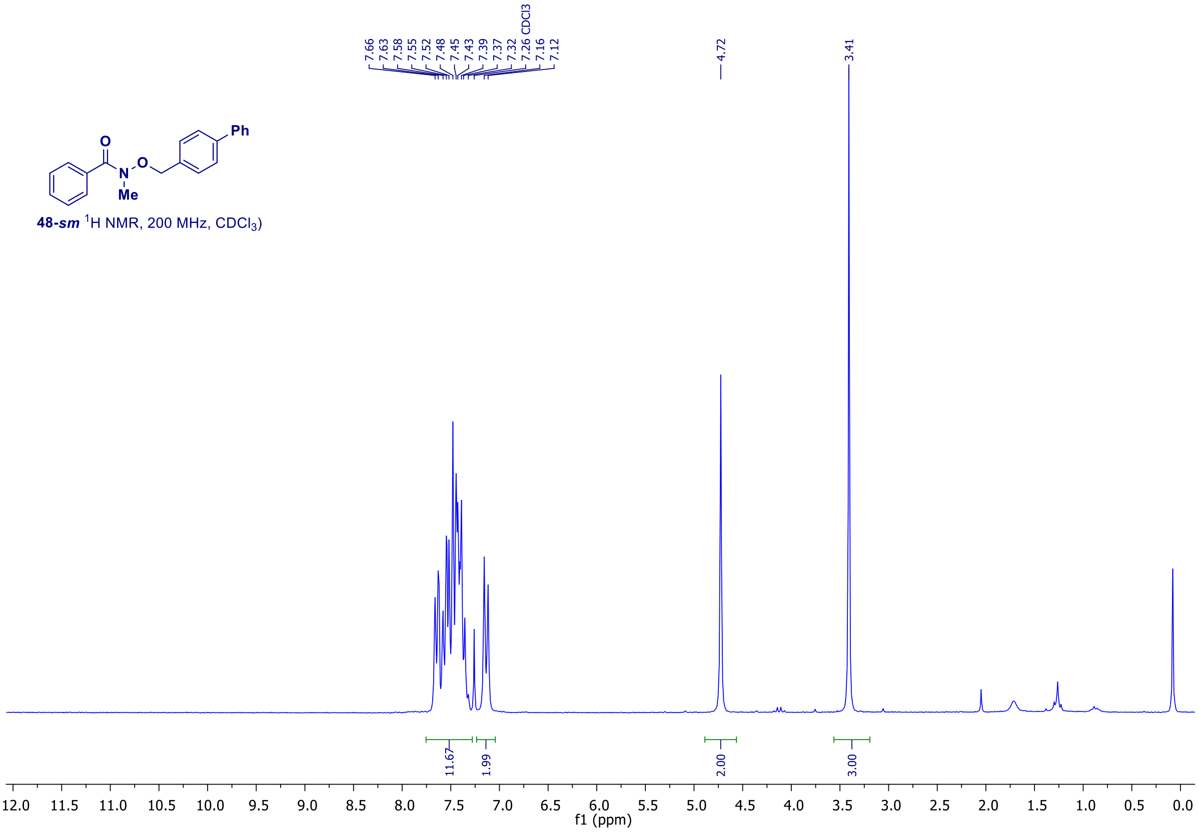

48-*sm* ^{1}H NMR, 200 MHz, $CDCl_3$)

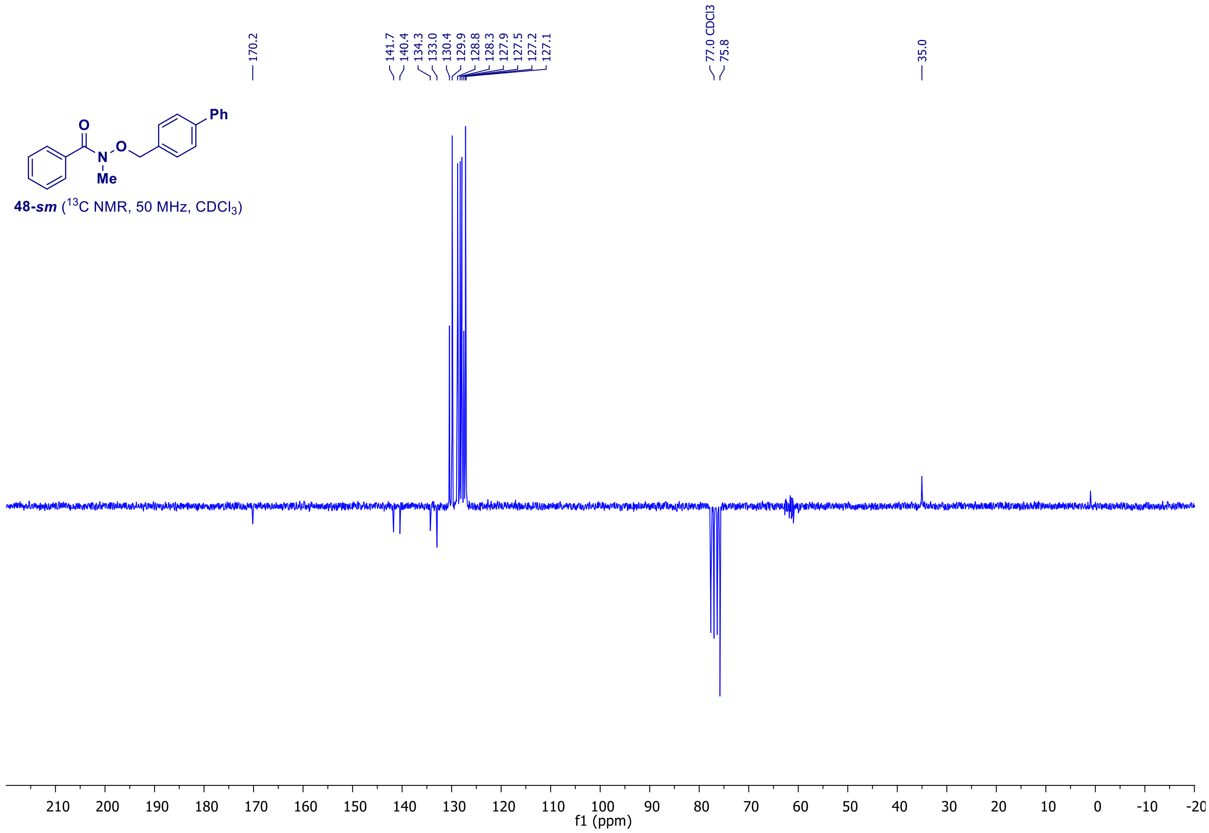

48-*sm* (^{13}C NMR, 50 MHz, $CDCl_3$)

Copies of ^{1}H and ^{13}C-NMR spectra of *N*-acyl-*N,O*-acetals

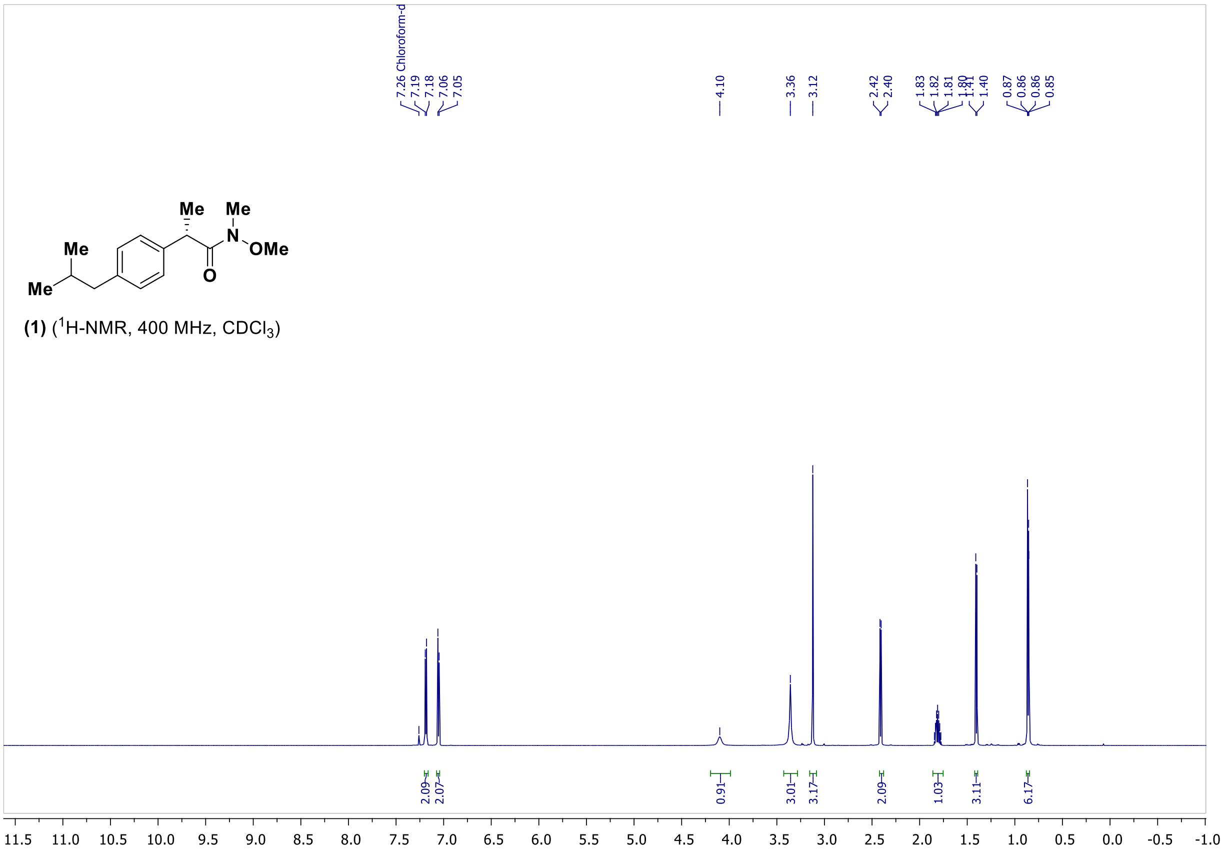

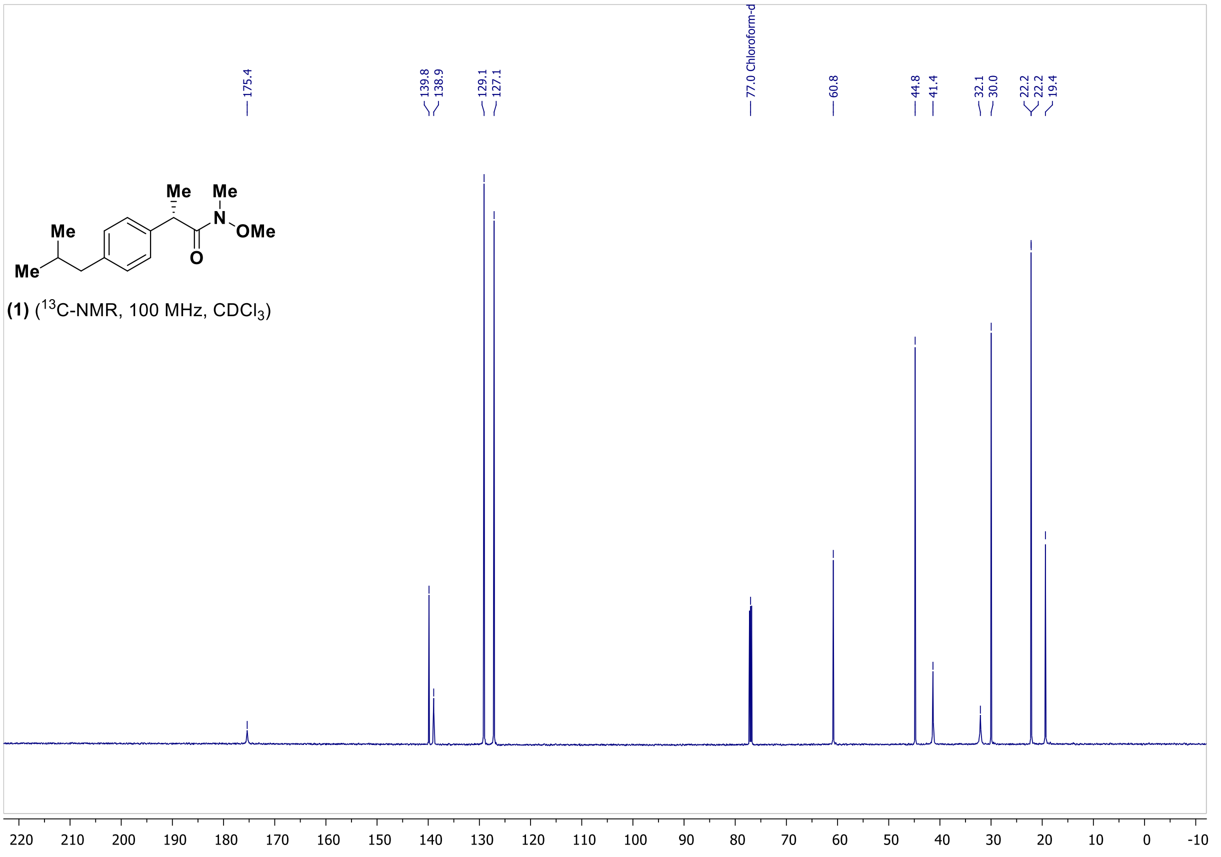

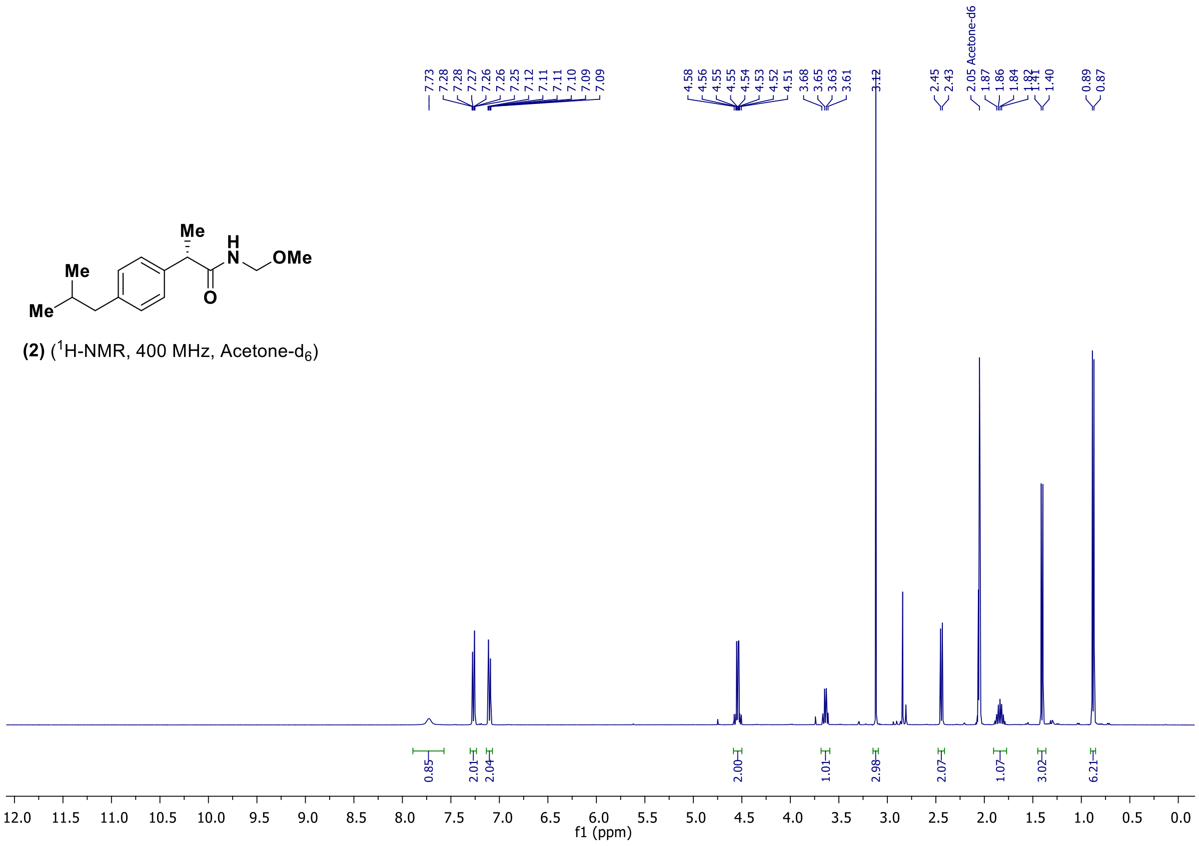
7.73 7.28 7.28 7.27 7.26 7.26 7.25 7.12 7.11 7.11 7.10 7.09 7.09
4.58 4.56 4.55 4.55 4.54 4.53 4.52 4.51 3.68 3.65 3.63 3.61 3.12
2.45 2.43 2.05 Acetone-d6 1.87 1.86 1.84 1.82 1.41 1.40 0.89 0.87
Me
H
N
OMe
Me
Me
O
(2) (^{1}H-NMR, 400 MHz, Acetone-d$_6$)
0.85 2.01 2.04 2.00 1.01 2.98 2.07 1.07 3.02 6.21
f1 (ppm)

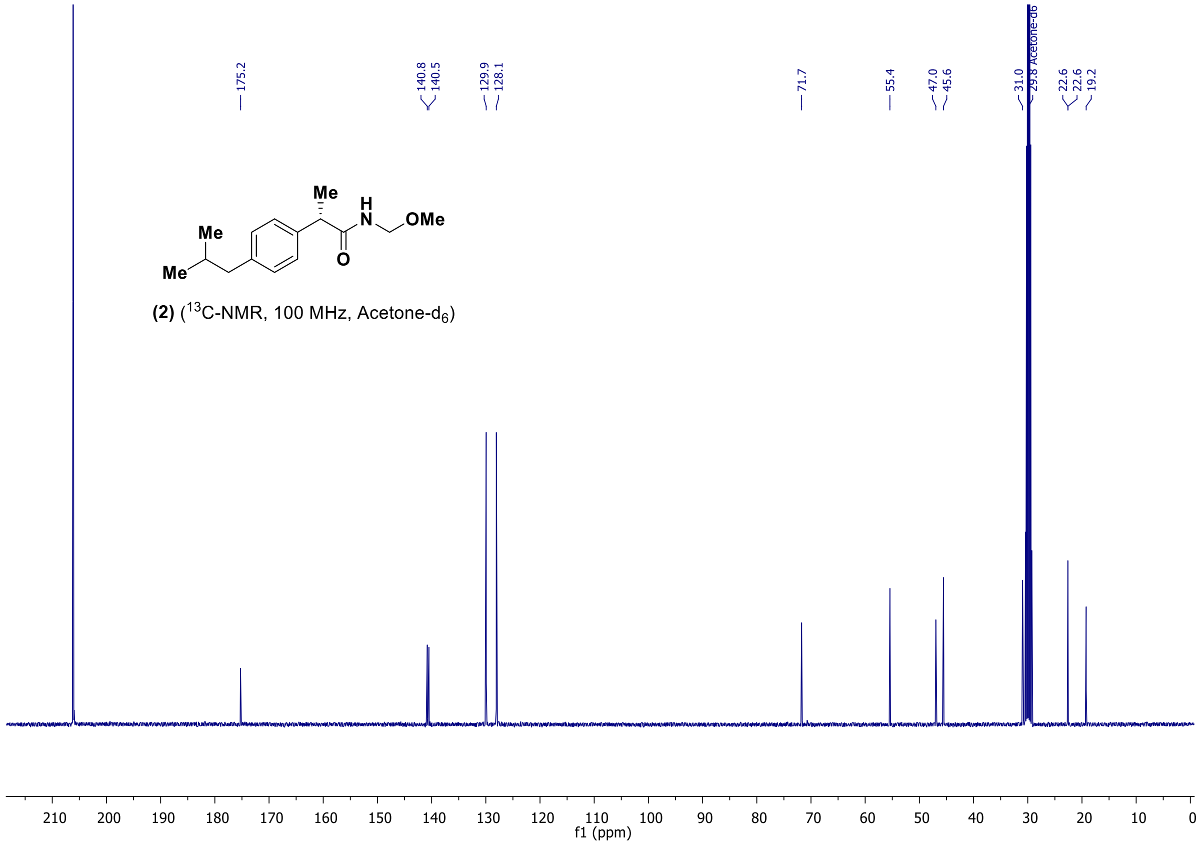
175.2 140.8 140.5 129.9 128.1 71.7 55.4 47.0 45.6 31.0 29.8 Acetone-d6 22.6 22.6 19.2
Me
H
N
OMe
Me
Me
O
(2) (^{13}C-NMR, 100 MHz, Acetone-d$_6$)
f1 (ppm)

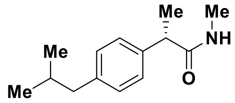

(2a) (^{1}H-NMR, 200 MHz, $CDCl_3$)

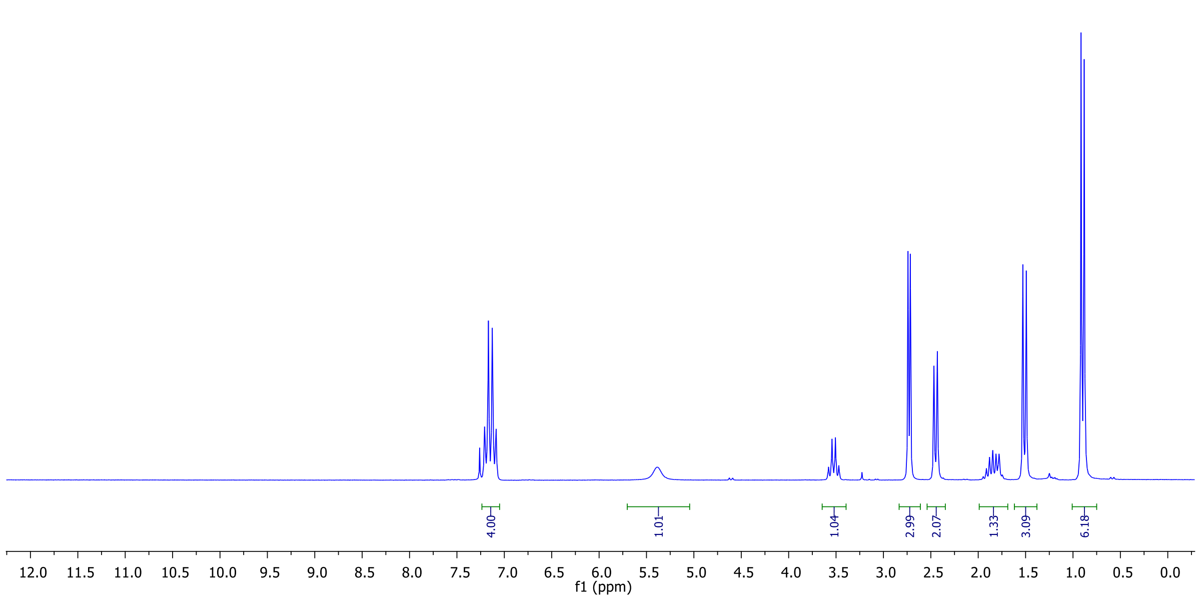

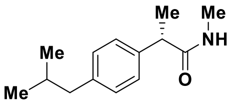

(2a) (^{13}C-NMR, 100 MHz, $CDCl_3$)

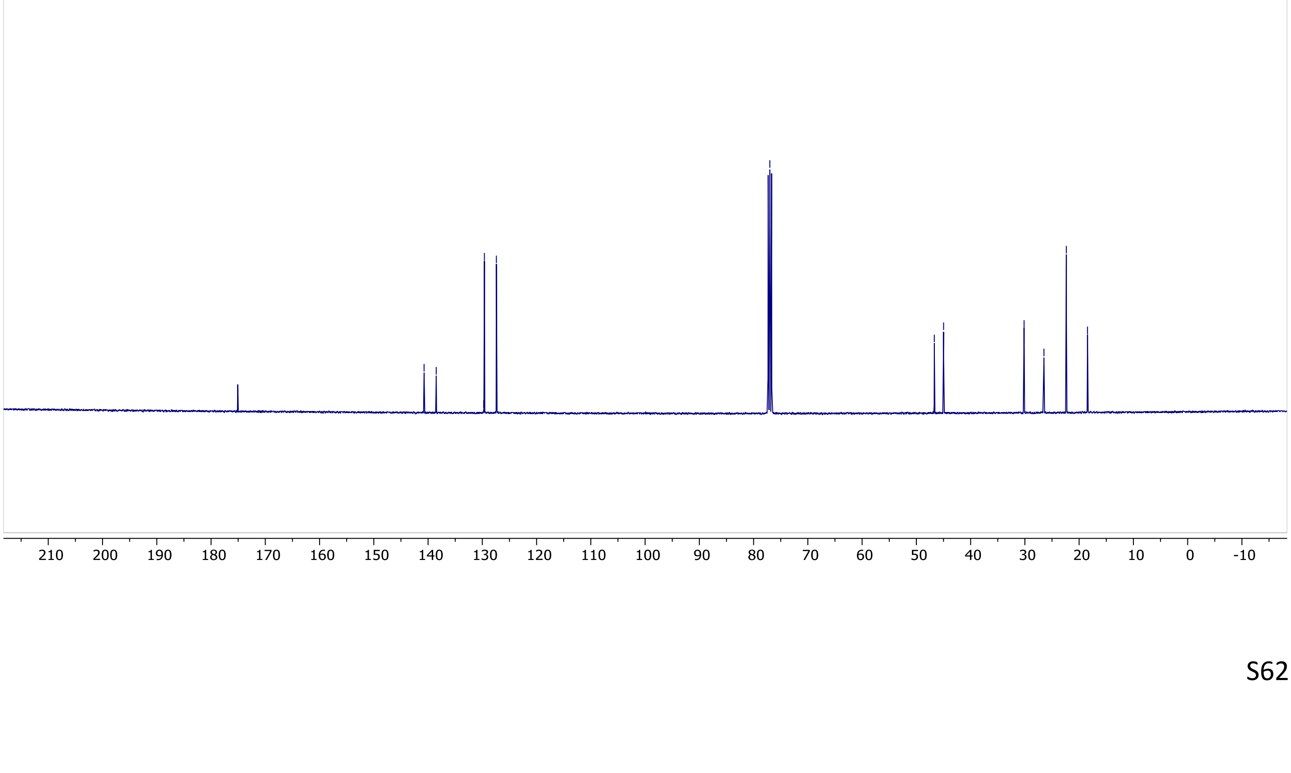

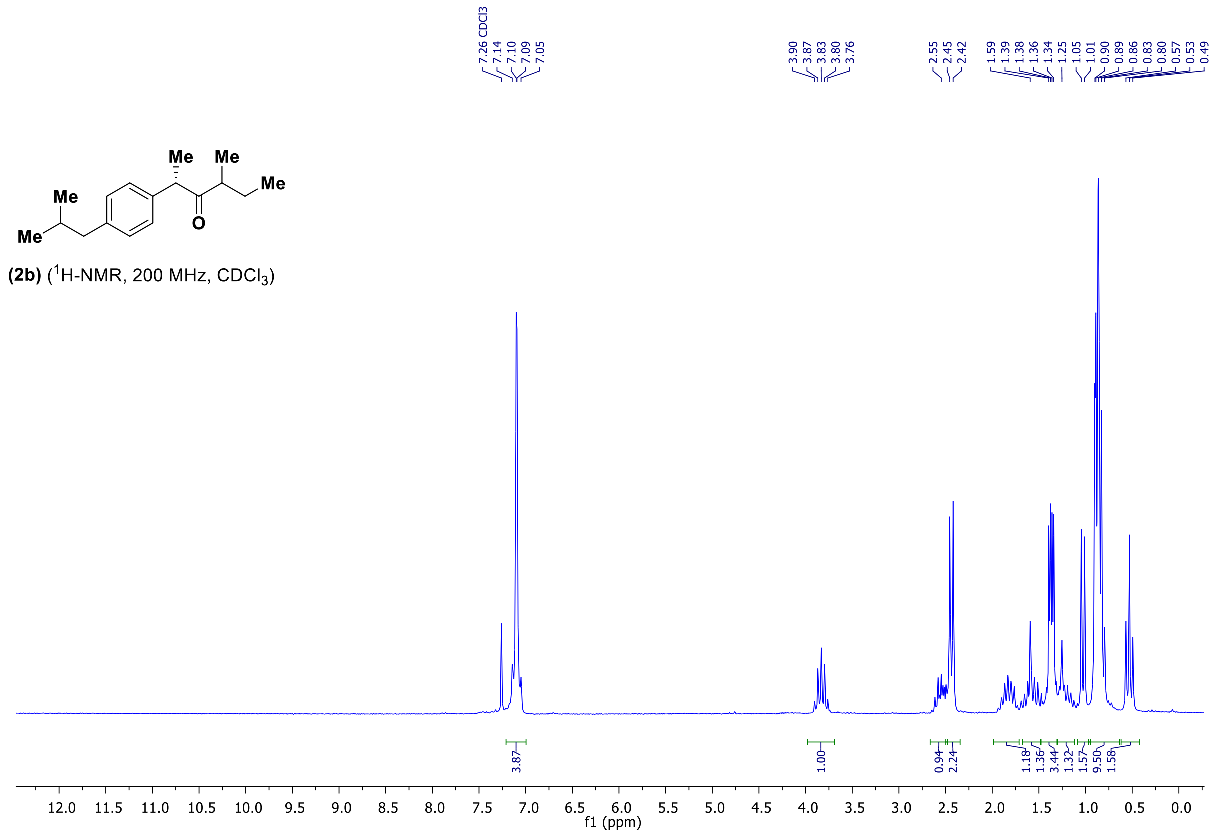
Me
Me
Me
Me
Me
O
(2b) (1H-NMR, 200 MHz, CDCl3)
7.26 CDCl3
7.14
7.10
7.09
7.05
3.90
3.87
3.83
3.80
3.76
2.55
2.45
2.42
1.59
1.39
1.38
1.36
1.34
1.25
1.05
1.01
0.90
0.89
0.86
0.83
0.80
0.57
0.53
0.49
3.87
1.00
0.94
2.24
1.18
1.36
3.44
1.32
1.57
9.50
1.58
f1 (ppm)

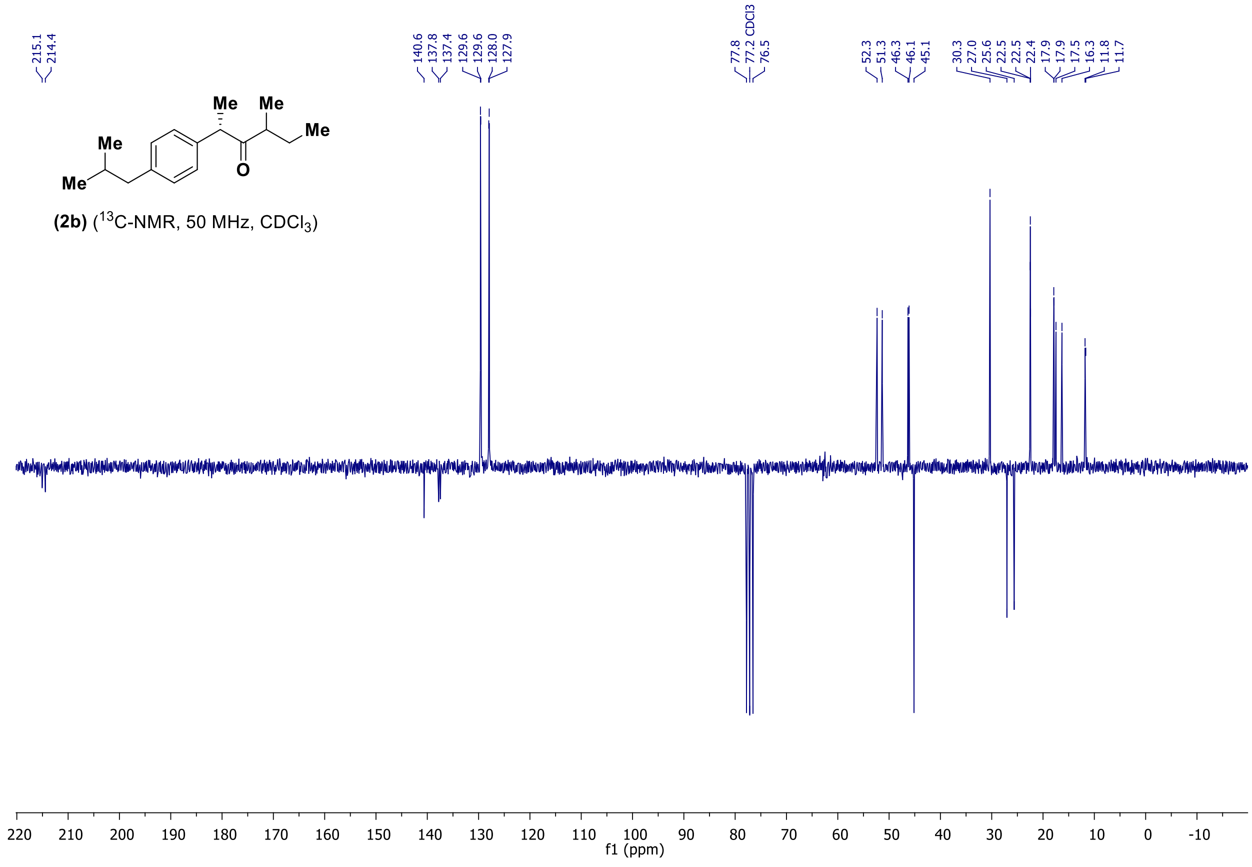
Me
Me
Me
Me
Me
O
(2b) (13C-NMR, 50 MHz, CDCl3)
215.1
214.4
140.6
137.8
137.4
129.6
129.6
128.0
127.9
77.8
77.2 CDCl3
76.5
52.3
51.3
46.3
46.1
45.1
30.3
27.0
25.6
22.5
22.5
22.4
17.9
17.9
17.5
16.3
11.8
11.7
f1 (ppm)

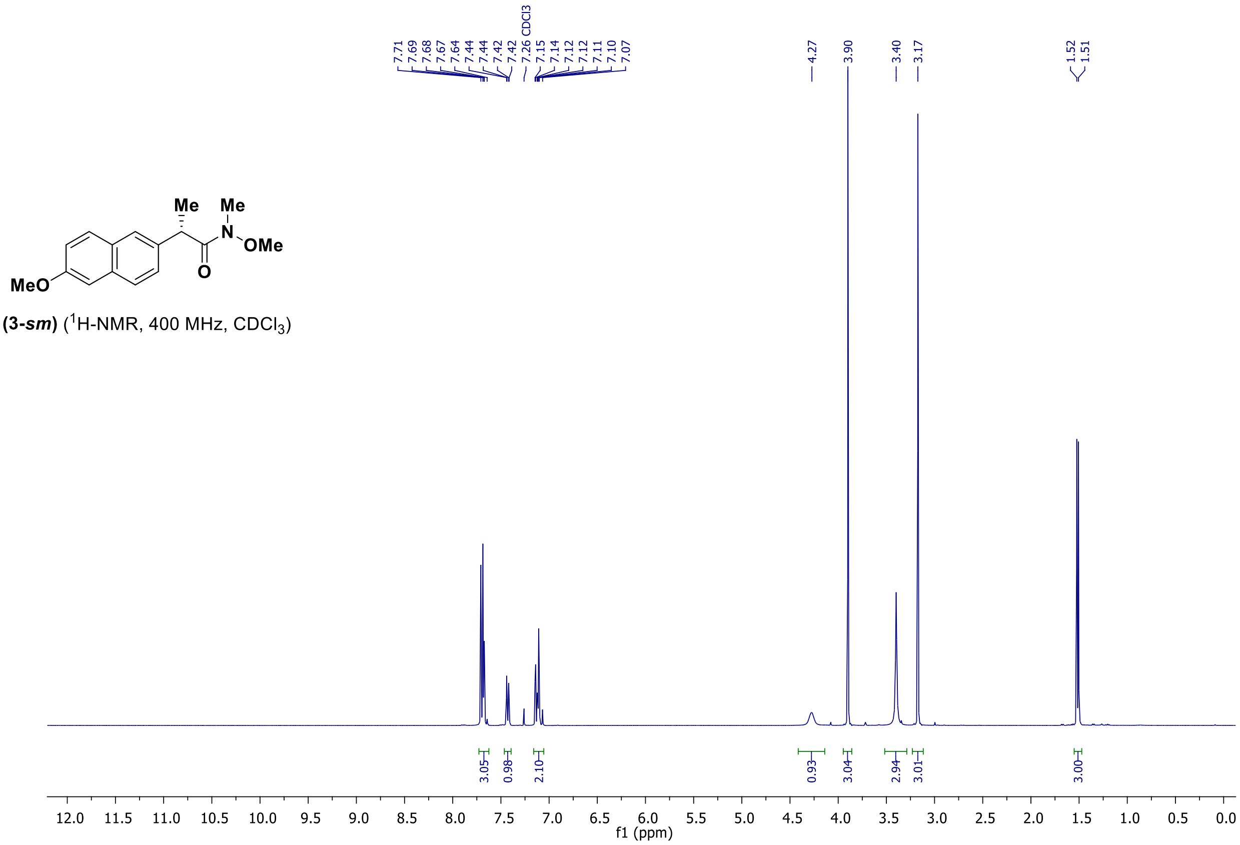

(3-*sm*) (^{1}H-NMR, 400 MHz, $CDCl_3$)

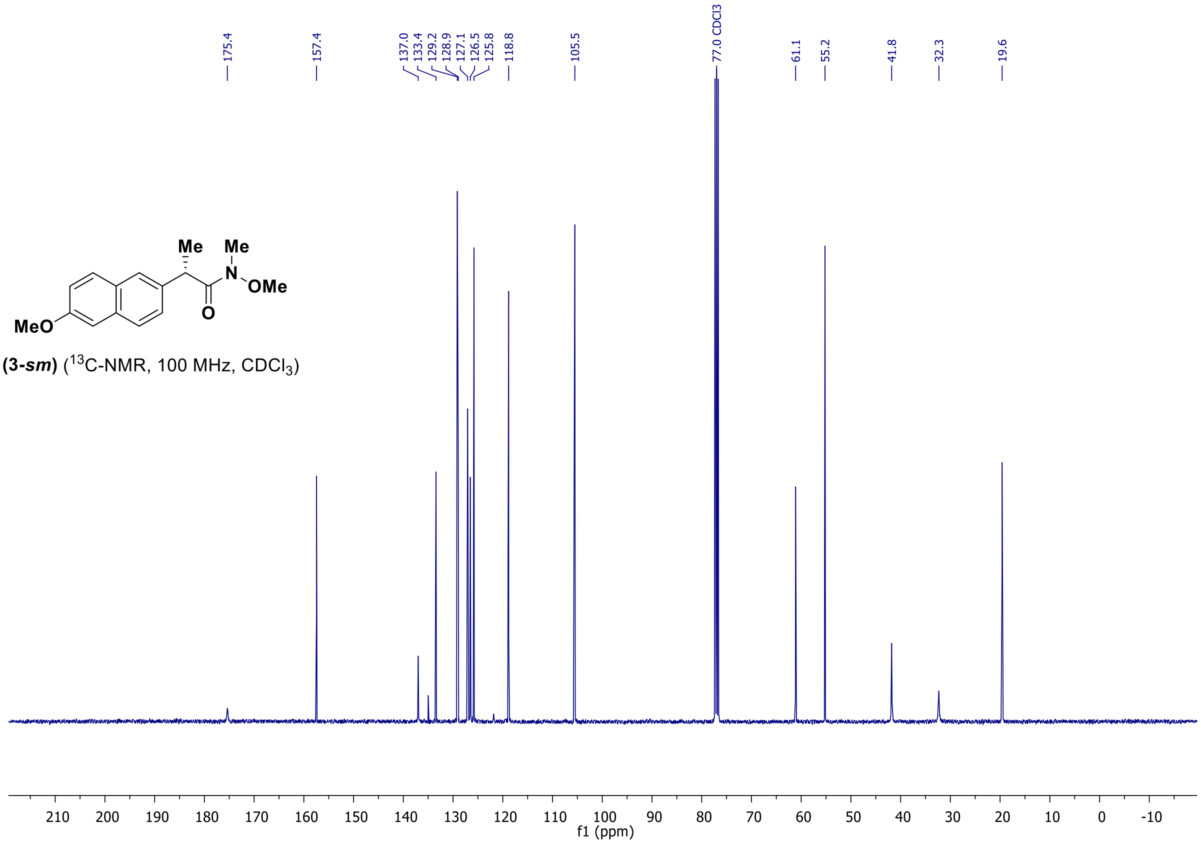

(3-*sm*) (^{13}C-NMR, 100 MHz, $CDCl_3$)

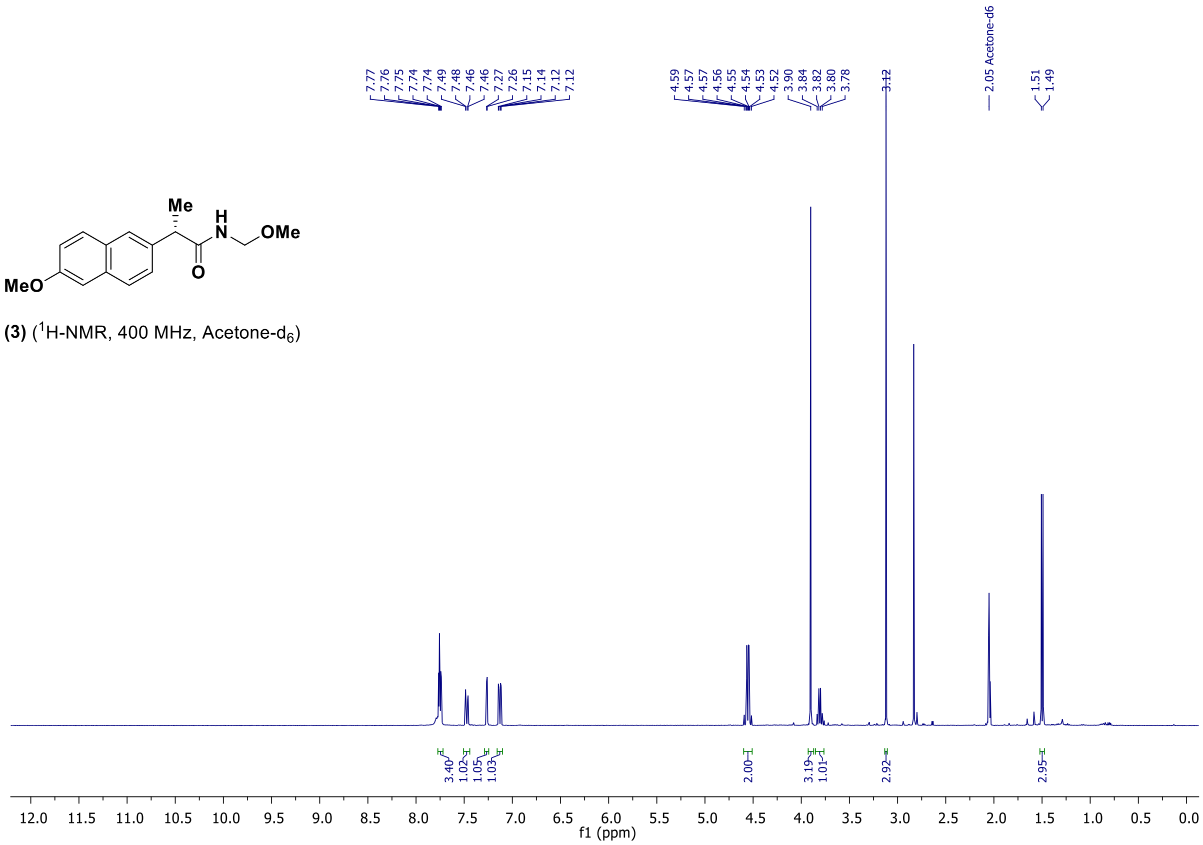

(3) (^{1}H-NMR, 400 MHz, Acetone-d$_6$)

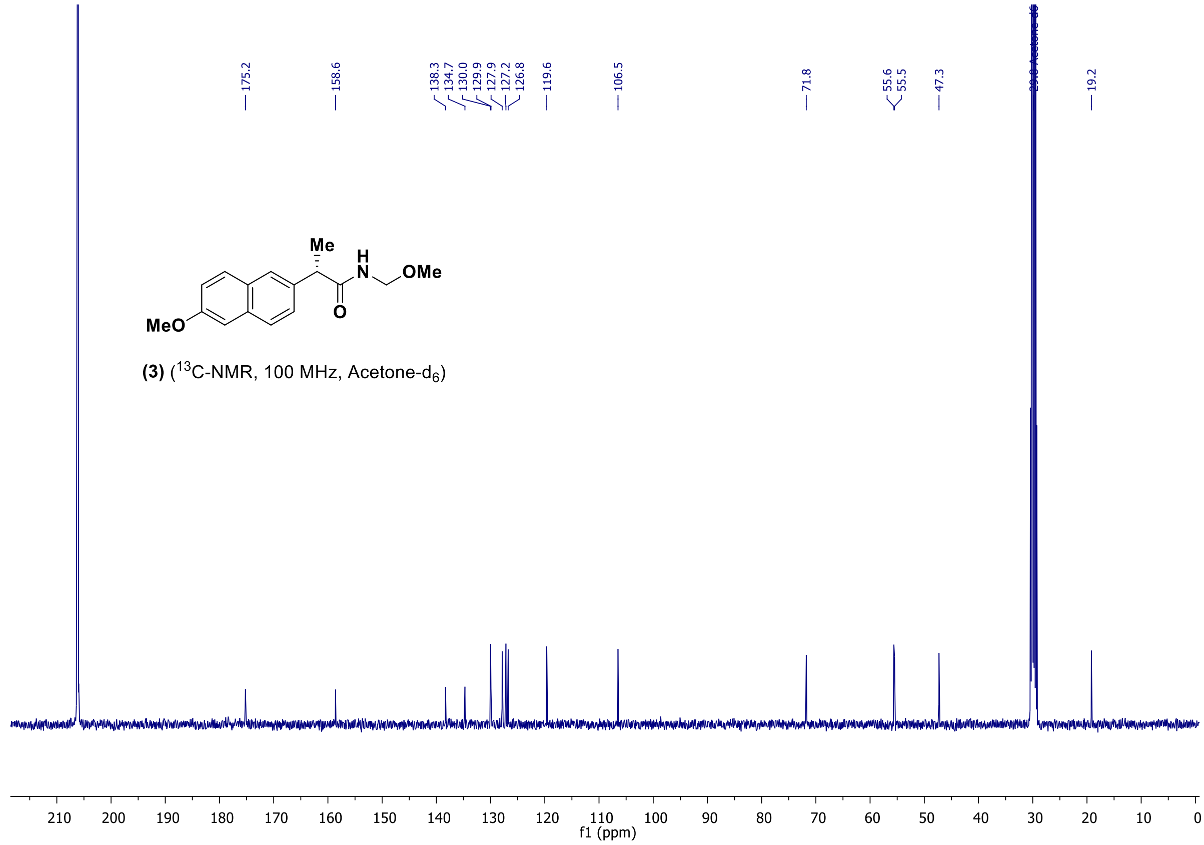

(3) (^{13}C-NMR, 100 MHz, Acetone-d$_6$)

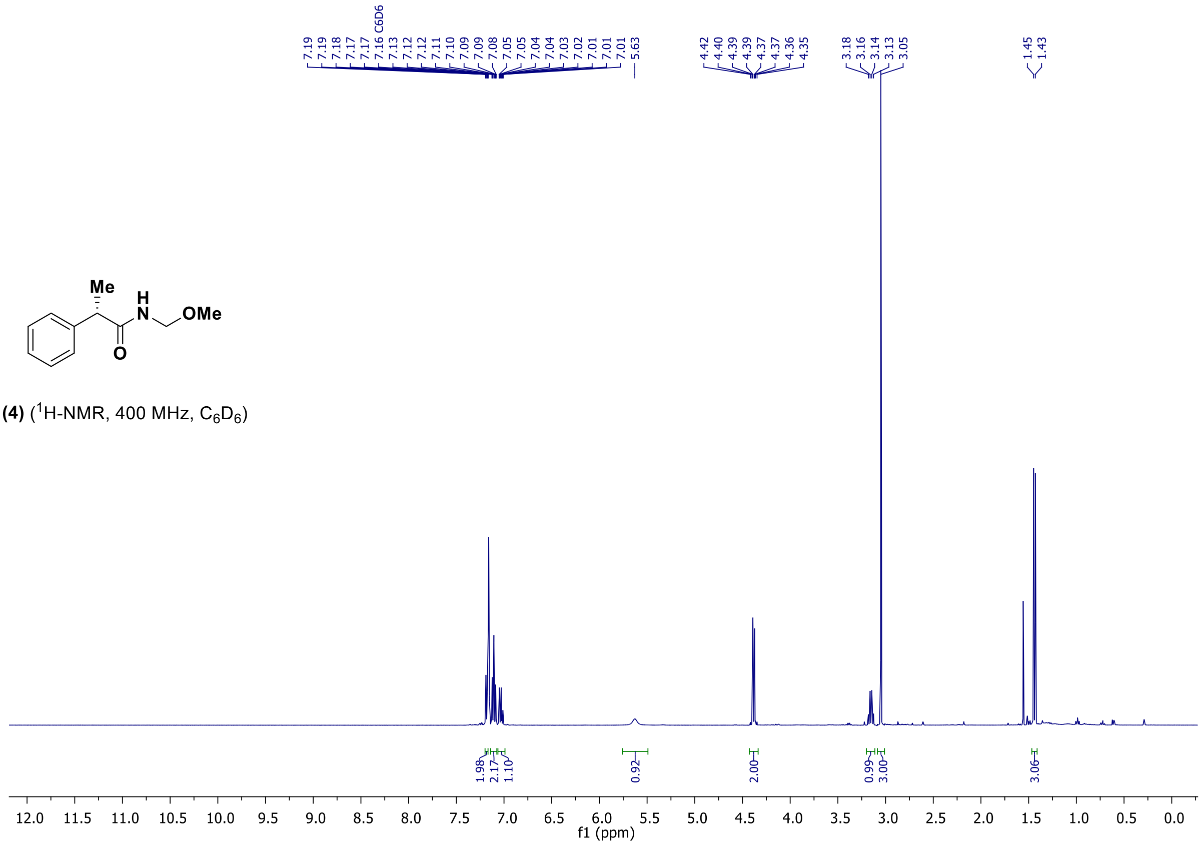

(4) (^{1}H-NMR, 400 MHz, C_6D_6)

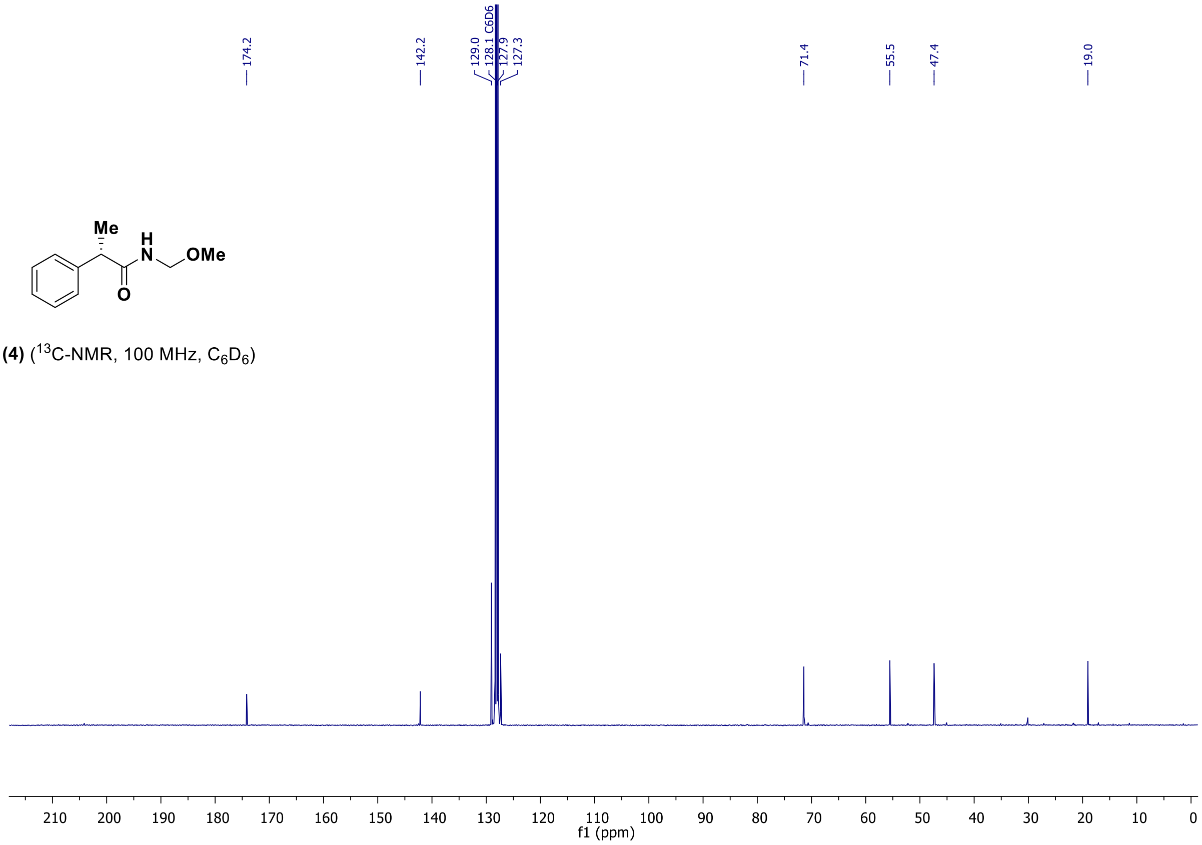

(4) (^{13}C-NMR, 100 MHz, C_6D_6)

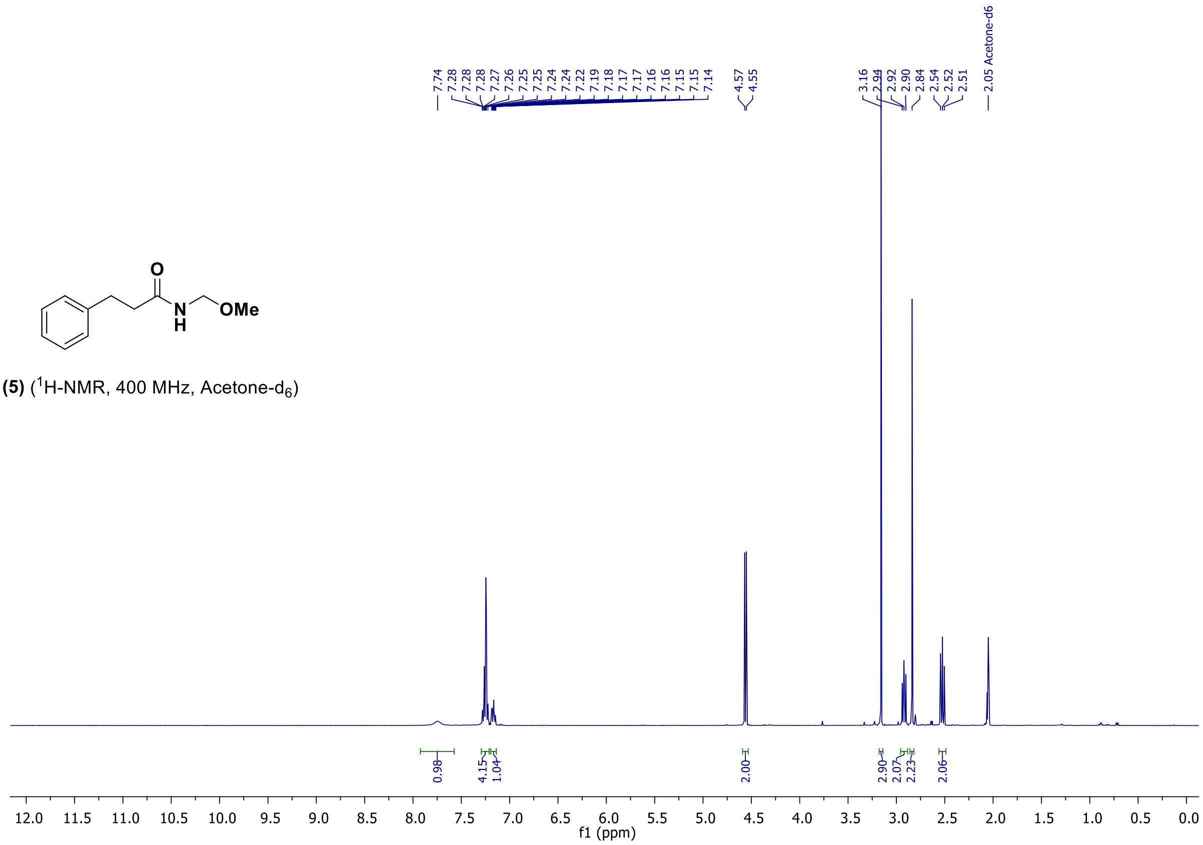

(5) (^{1}H-NMR, 400 MHz, Acetone-d_6)

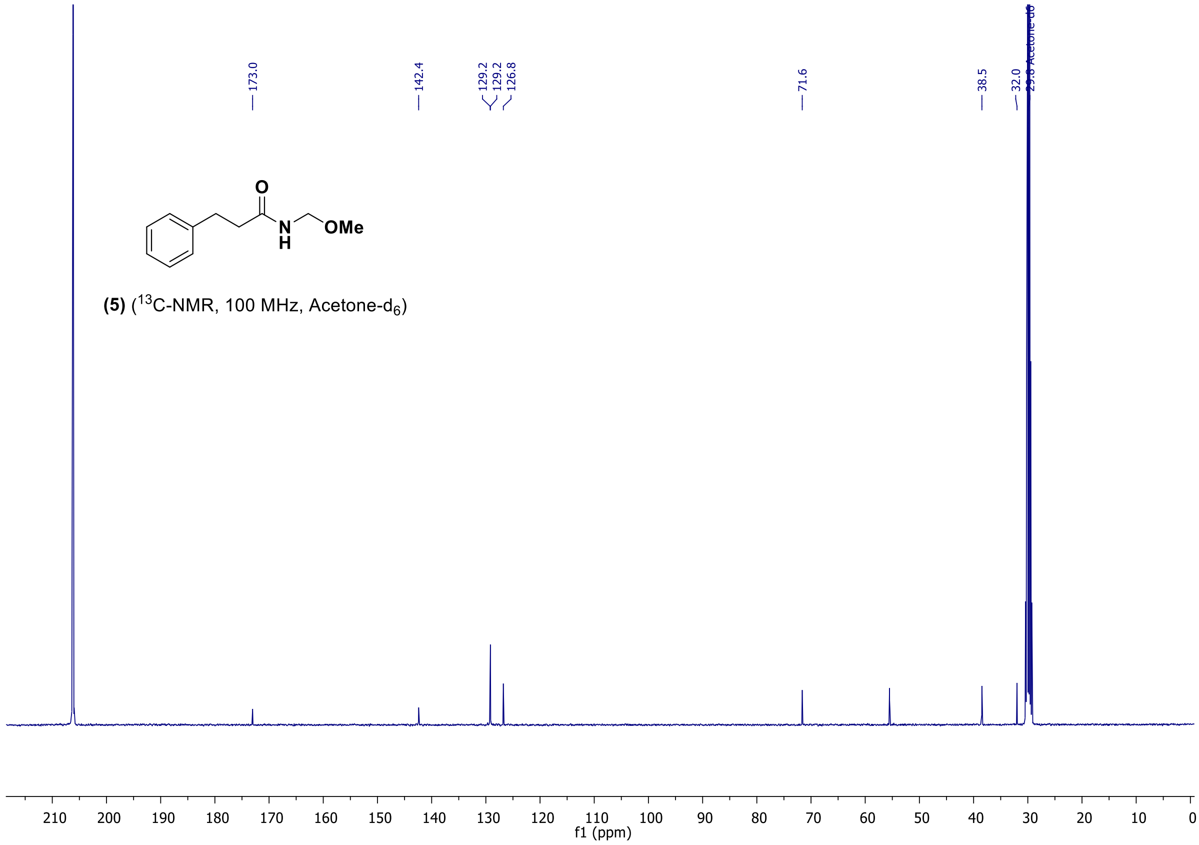

(5) (^{13}C-NMR, 100 MHz, Acetone-d_6)

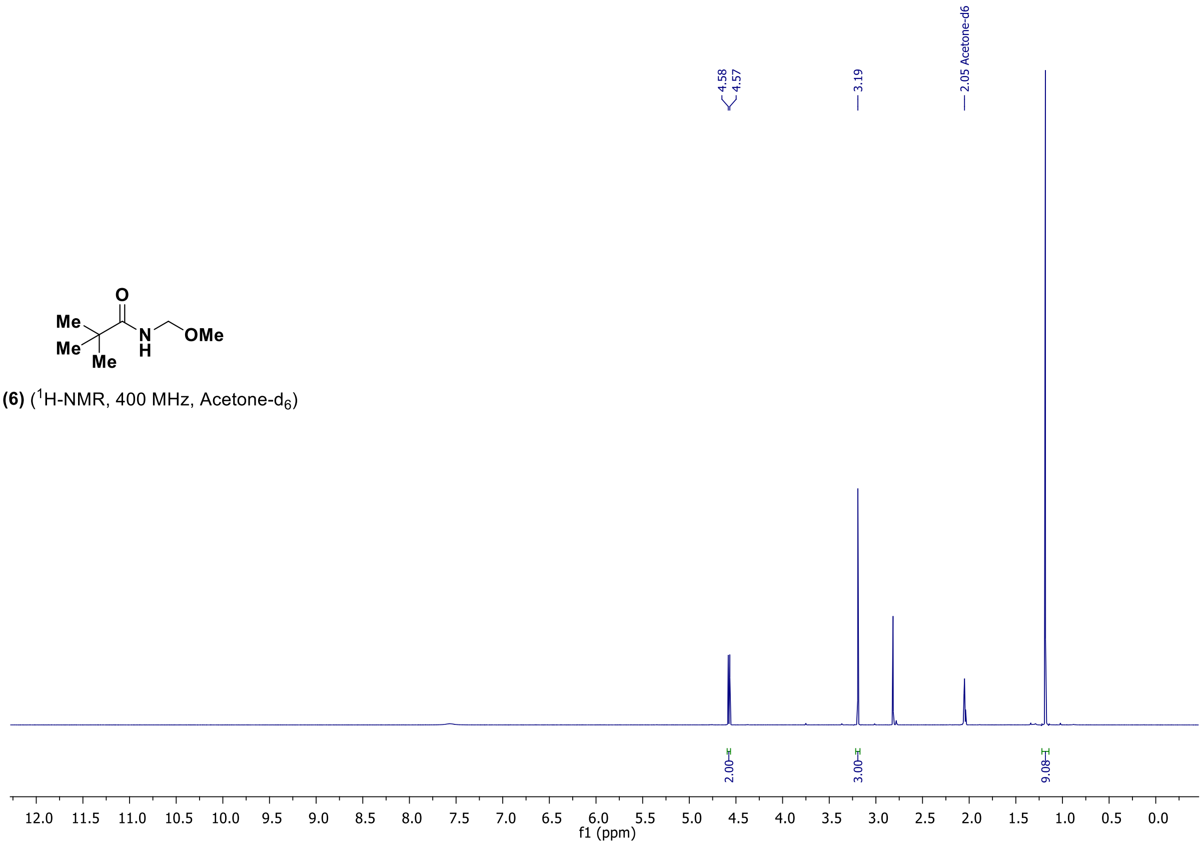

(6) (^{1}H-NMR, 400 MHz, Acetone-d$_6$)

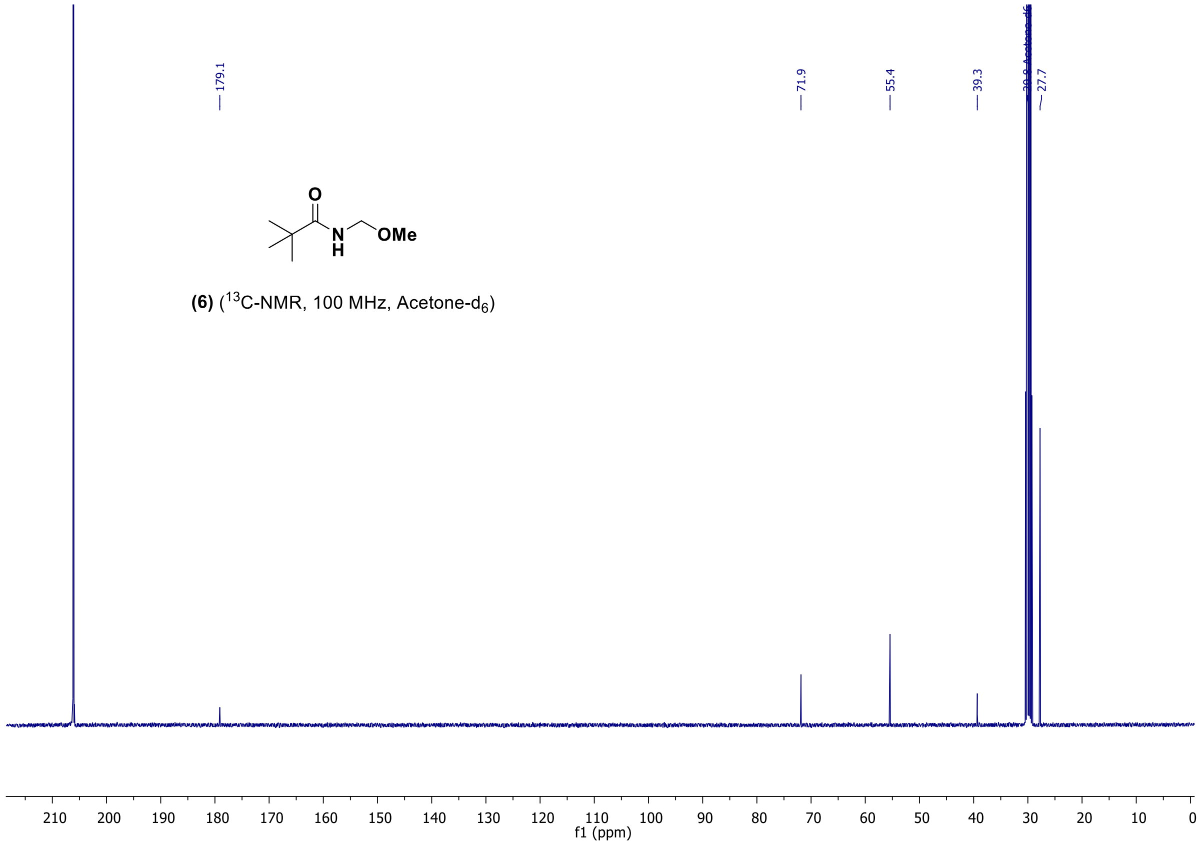

(6) (^{13}C-NMR, 100 MHz, Acetone-d$_6$)

(7) (^{1}H-NMR, 400 MHz, C_6D_6)

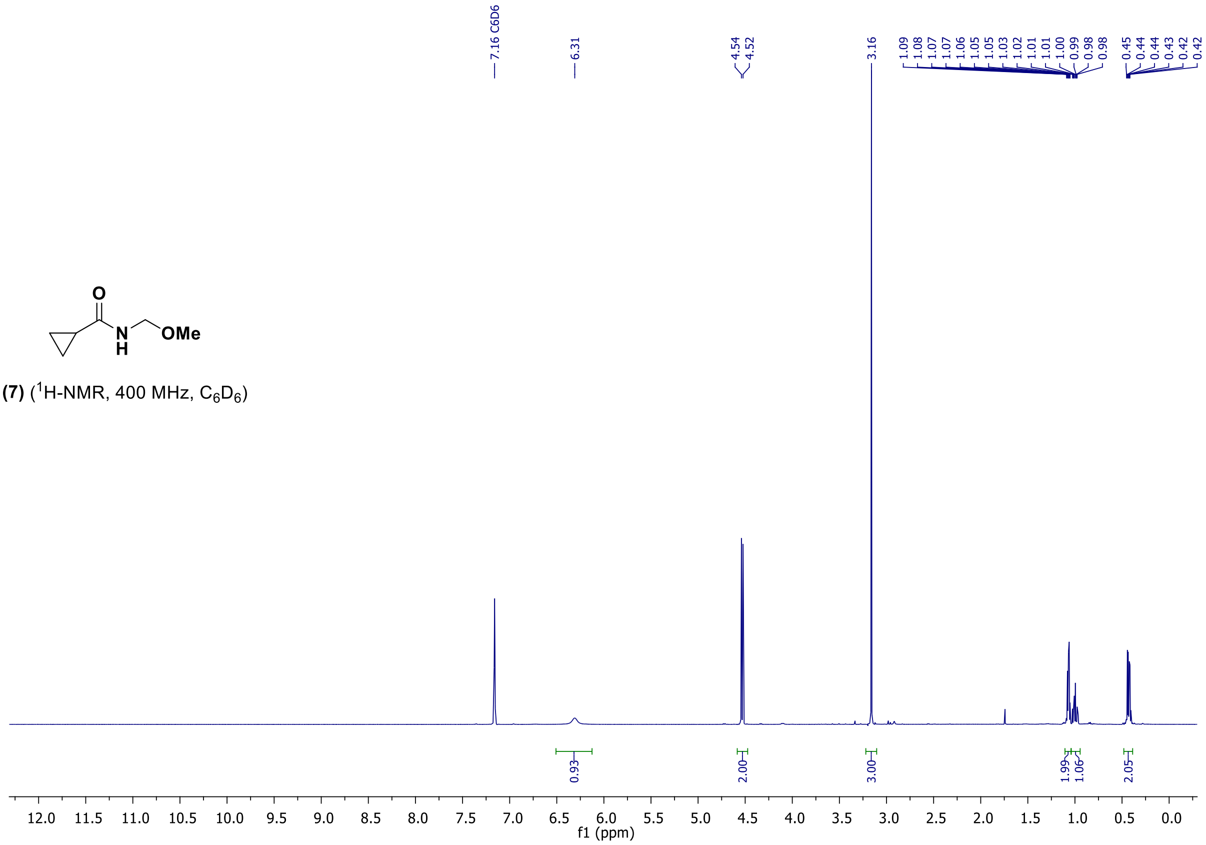

(7) (^{13}C-NMR, 100 MHz, C_6D_6)

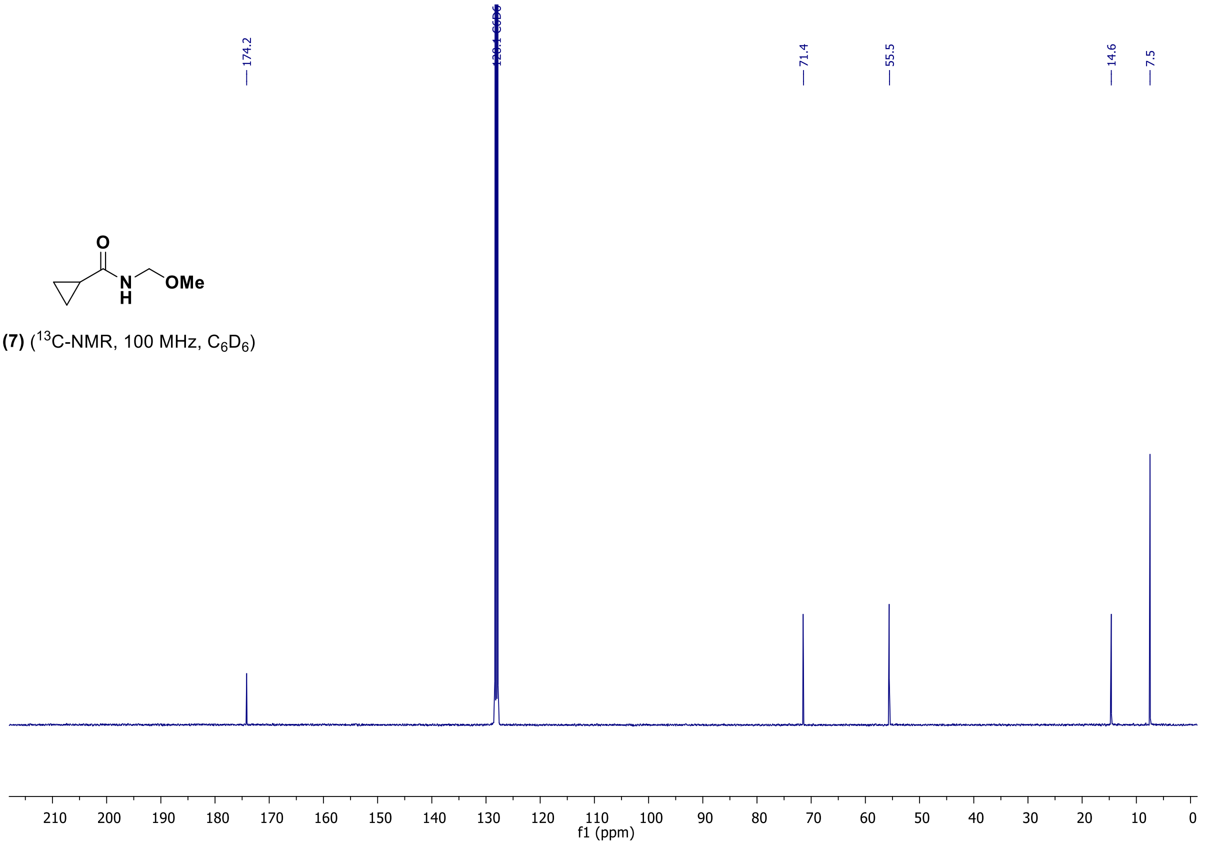

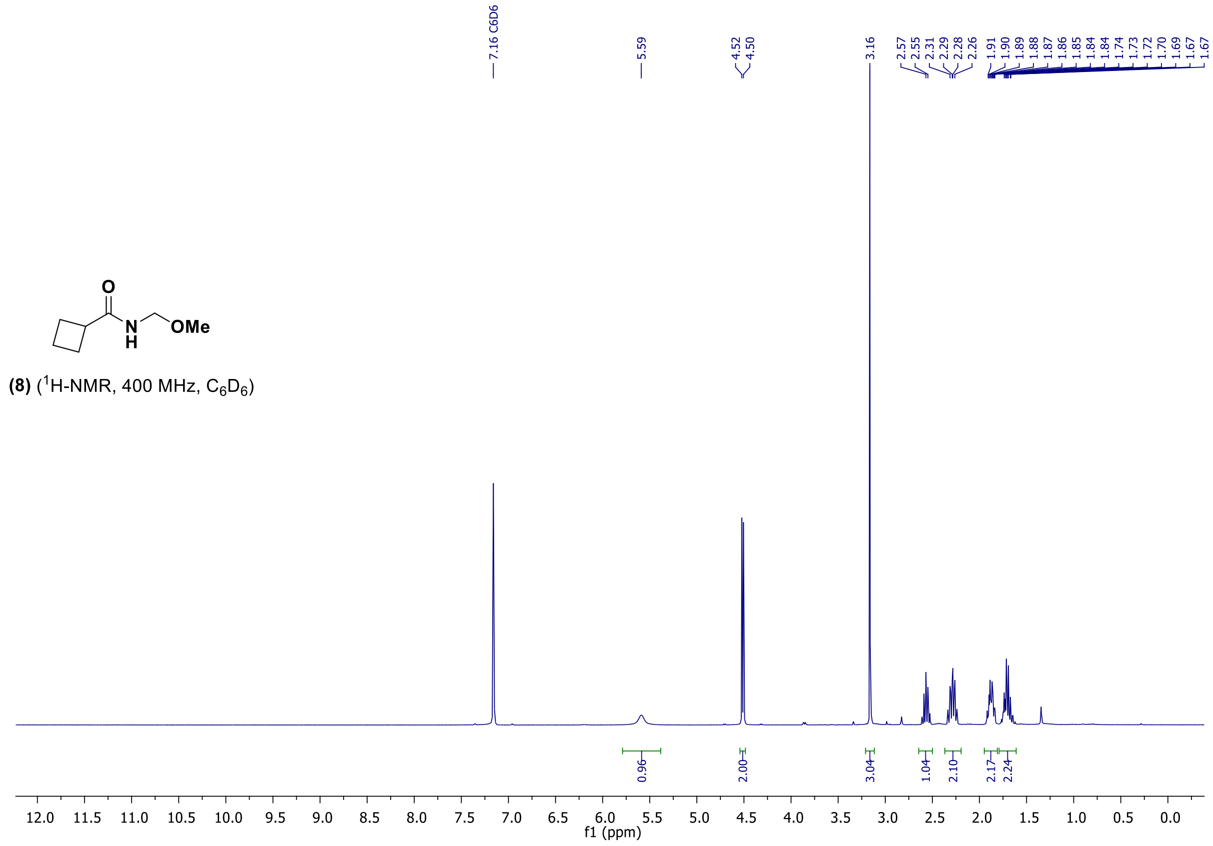

(8) (^{1}H-NMR, 400 MHz, C_6D_6)

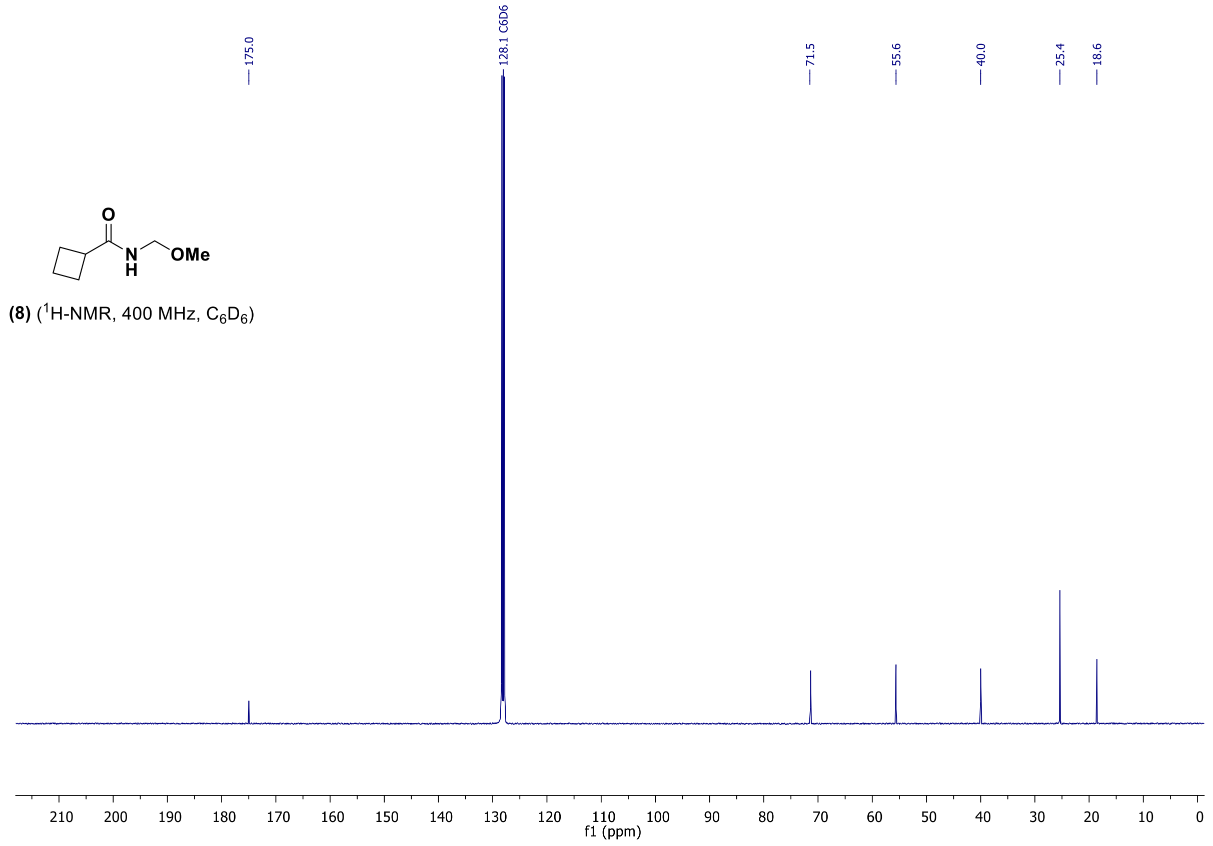

(8) (^{1}H-NMR, 400 MHz, C_6D_6)

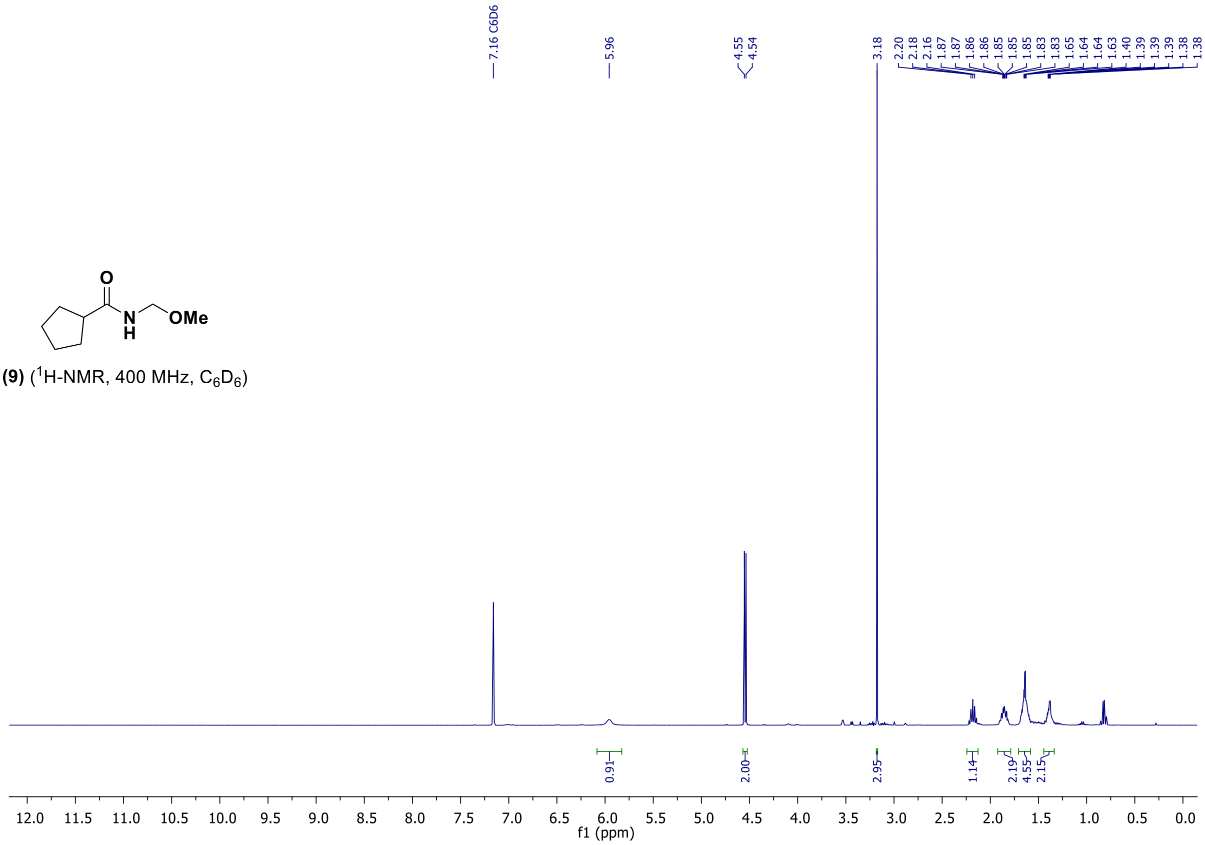

(9) (^{1}H-NMR, 400 MHz, C_6D_6)

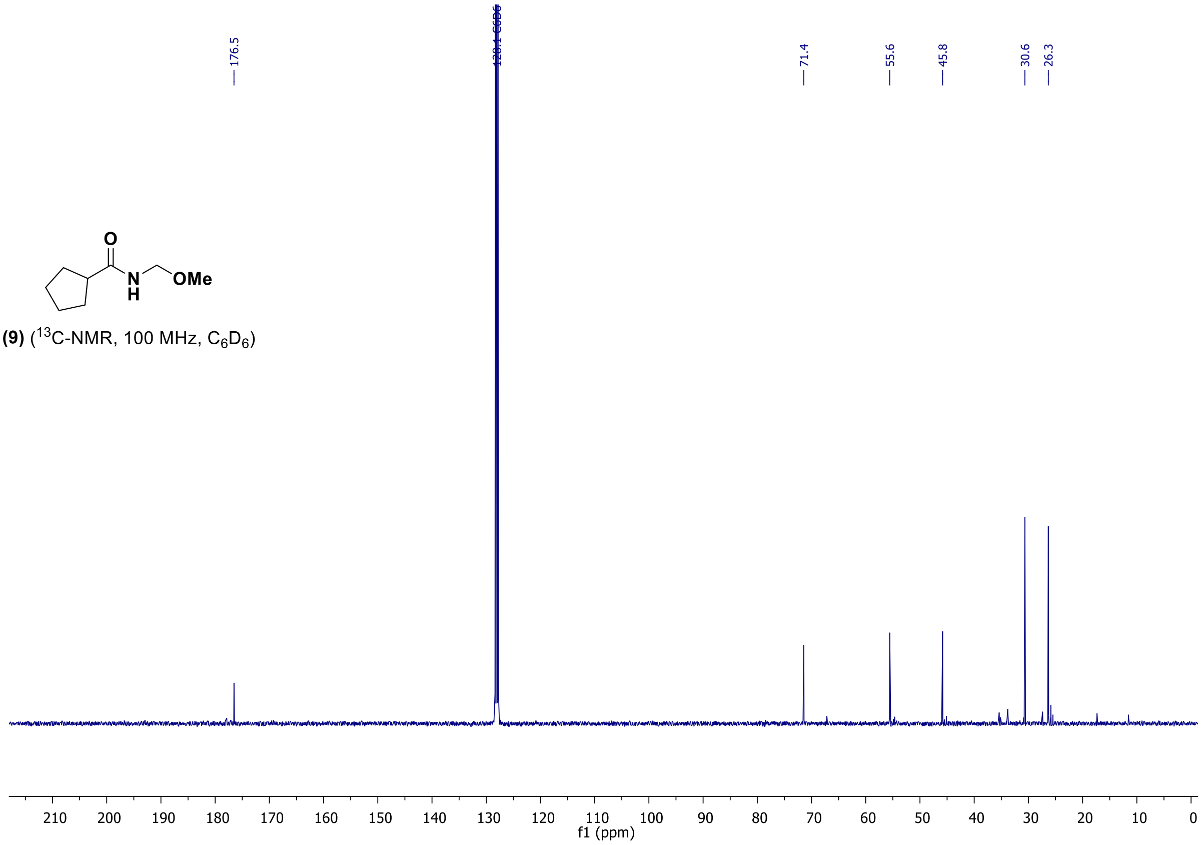

(9) (^{13}C-NMR, 100 MHz, C_6D_6)

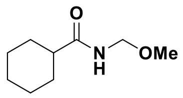

(10) (^{1}H-NMR, 400 MHz, C_6D_6)

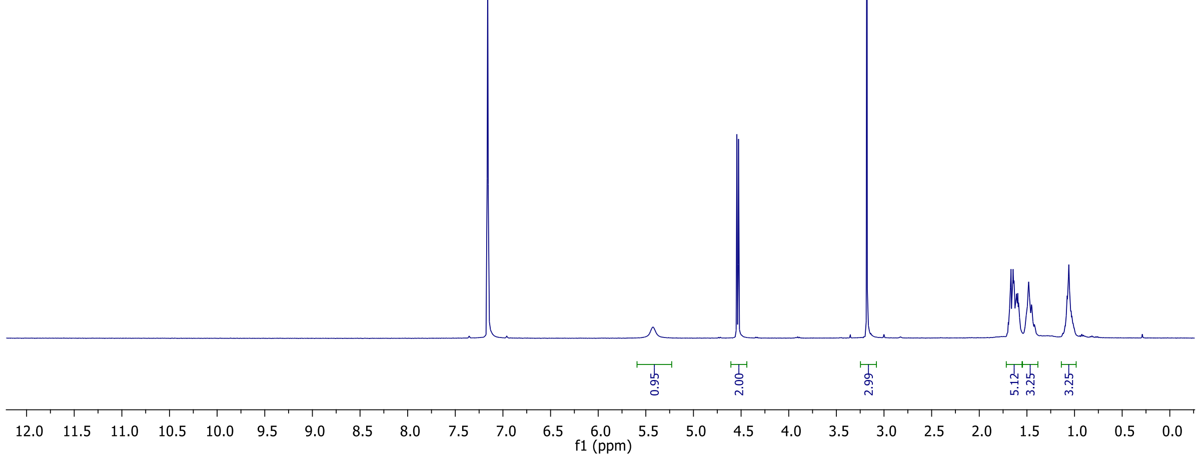

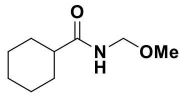

(10) (^{13}C-NMR, 100 MHz, C_6D_6)

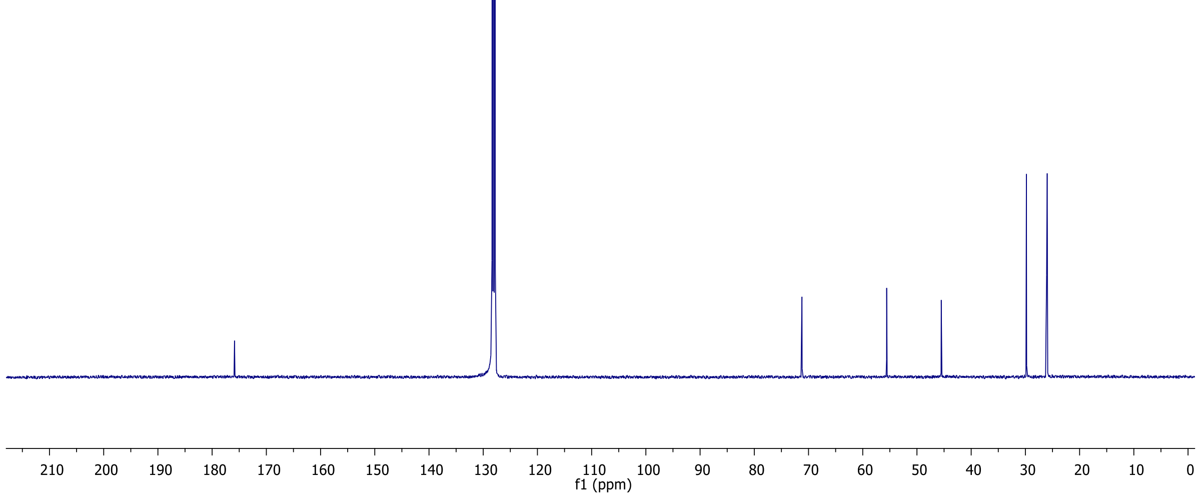

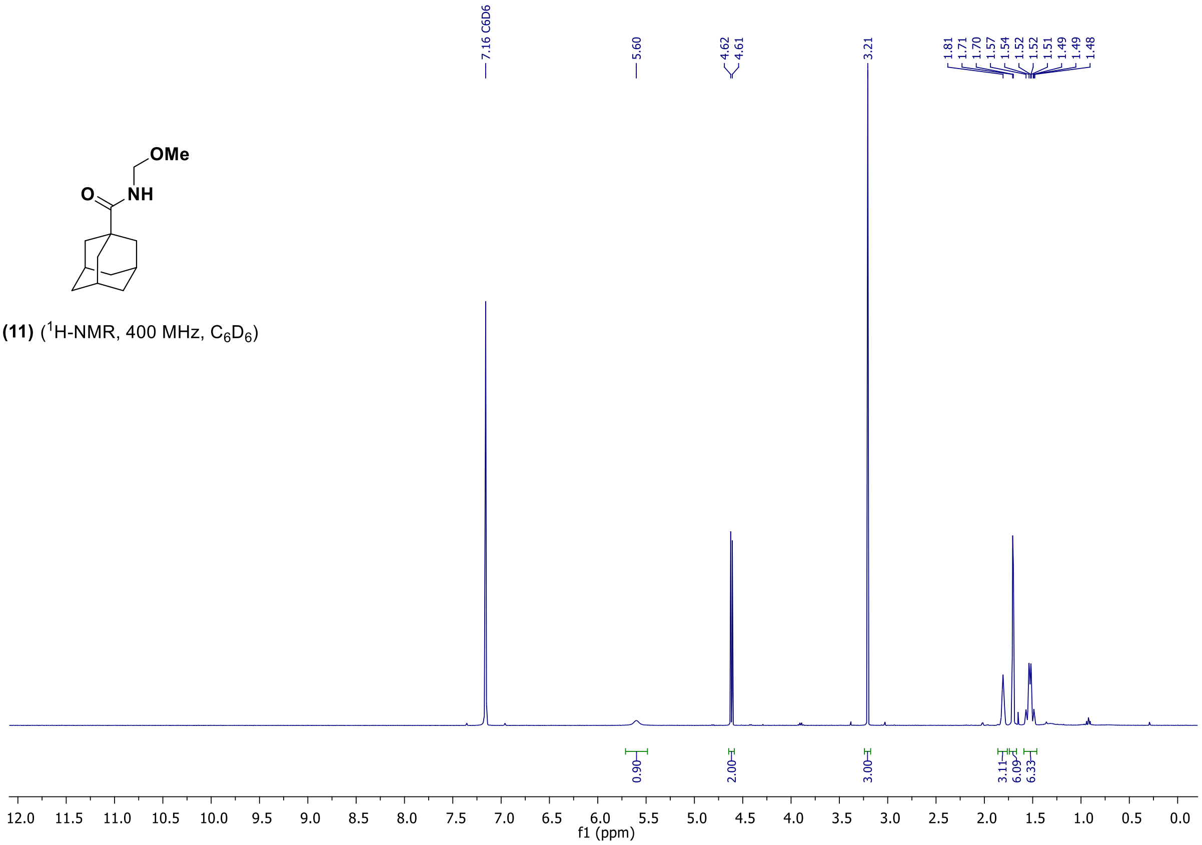

(11) (^{1}H-NMR, 400 MHz, C_6D_6)

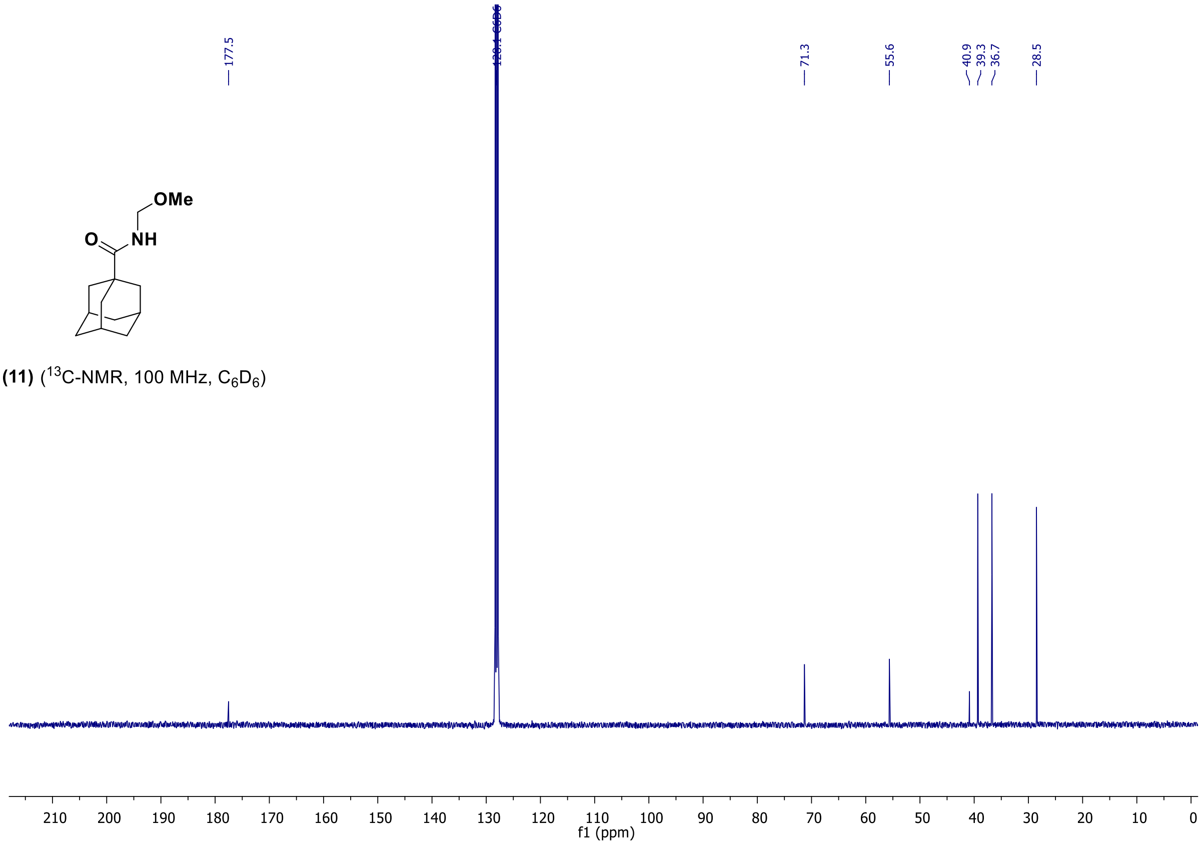

(11) (^{13}C-NMR, 100 MHz, C_6D_6)

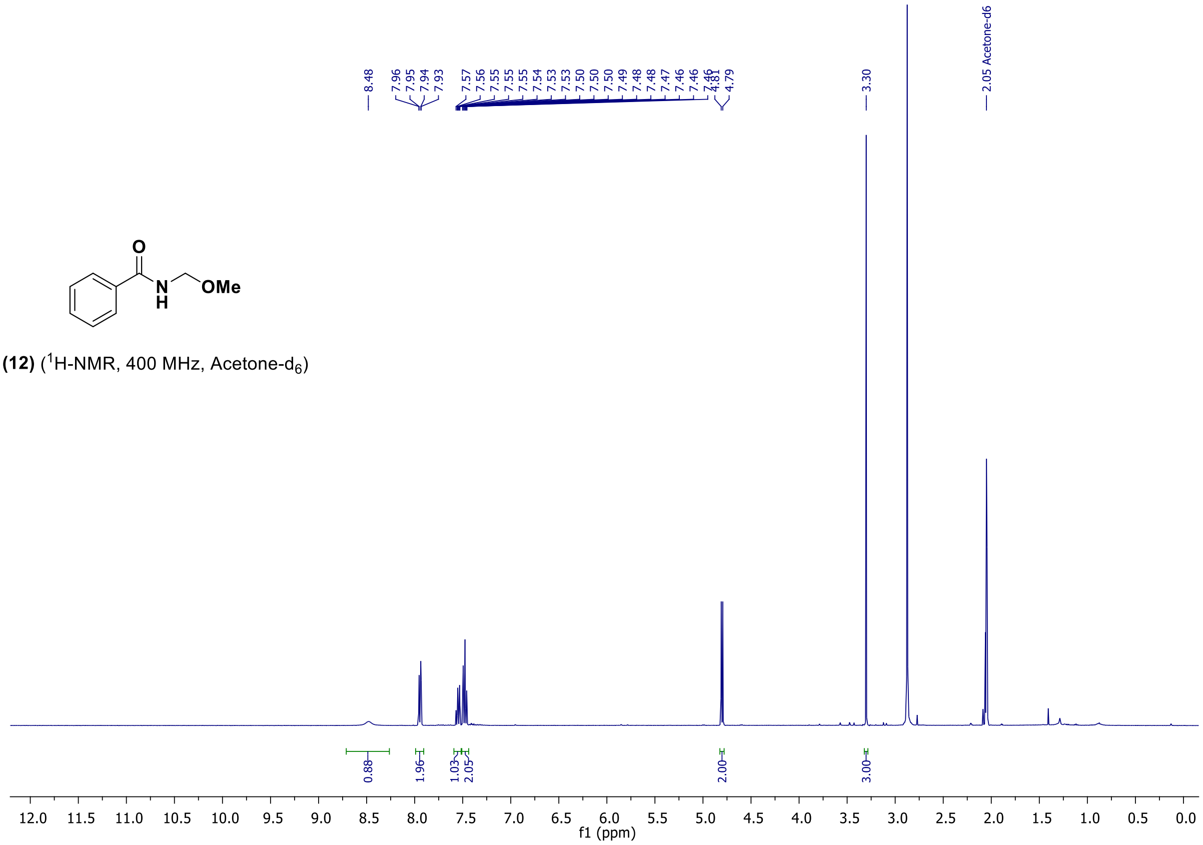

(12) (^{1}H-NMR, 400 MHz, Acetone-d_6)

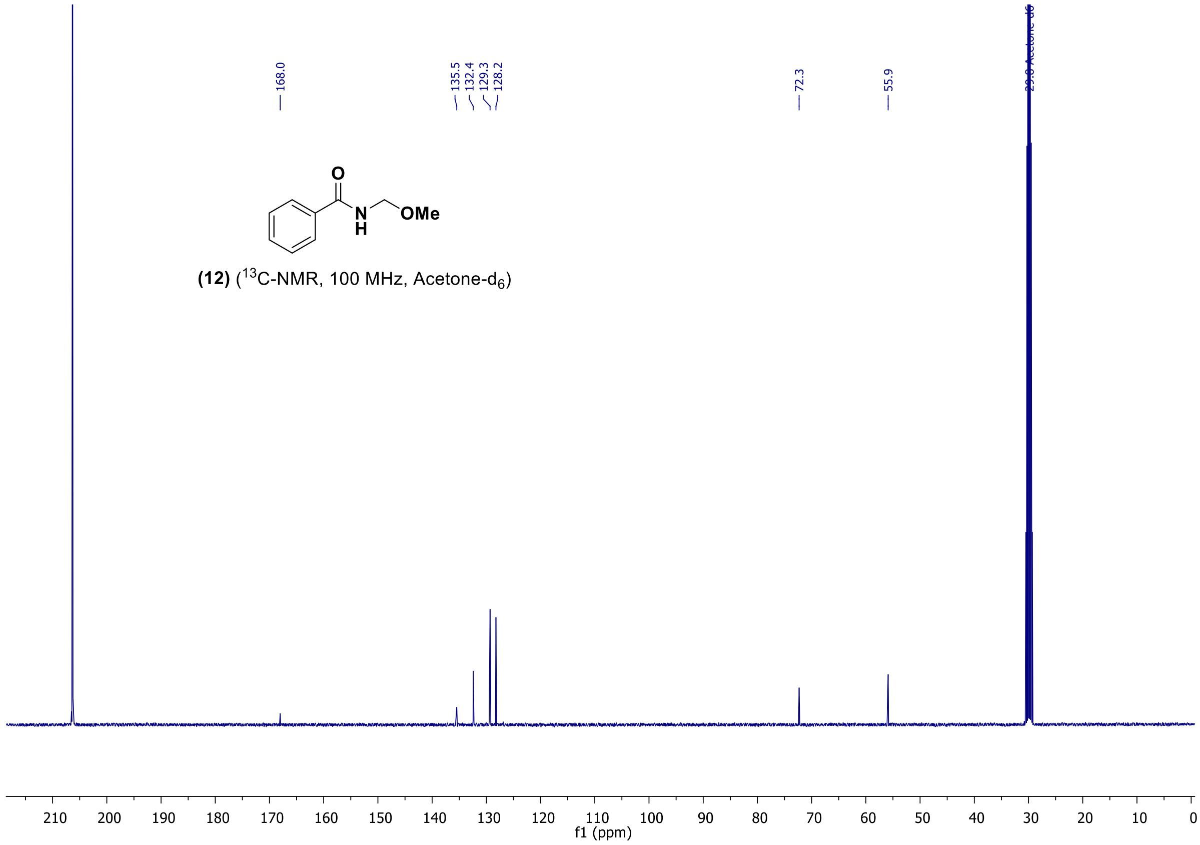

(12) (^{13}C-NMR, 100 MHz, Acetone-d_6)

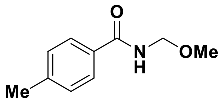

(13) (^{1}H-NMR, 400 MHz, C_6D_6)

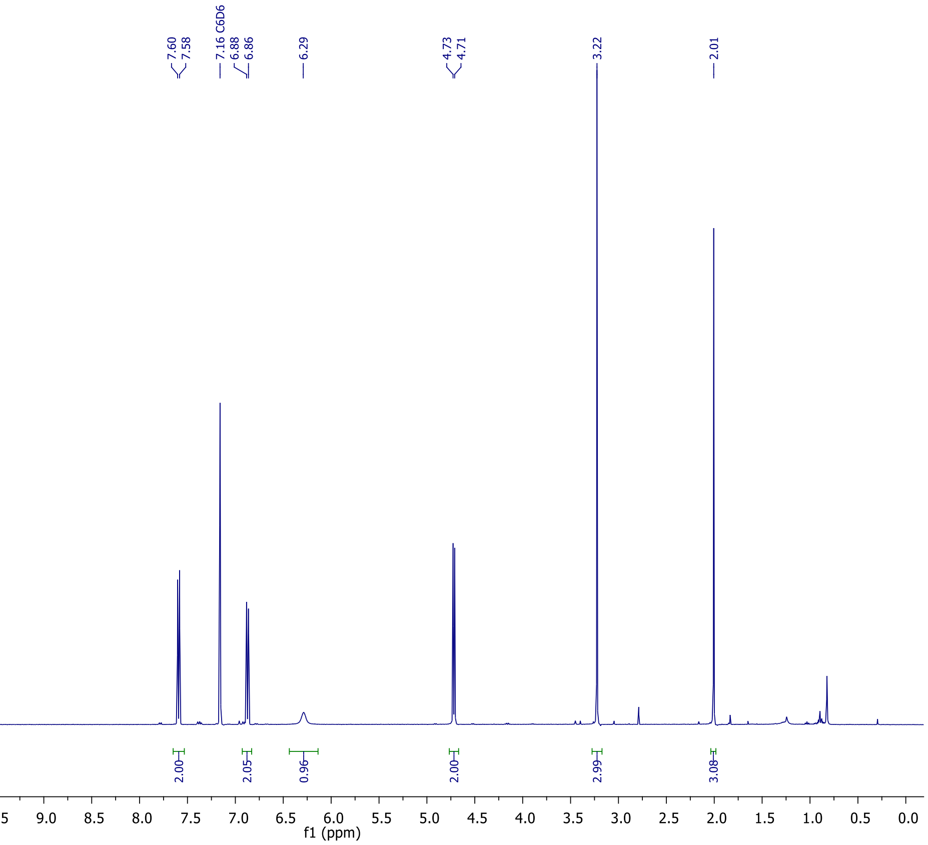

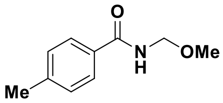

(13) (^{13}C-NMR, 100 MHz, C_6D_6)

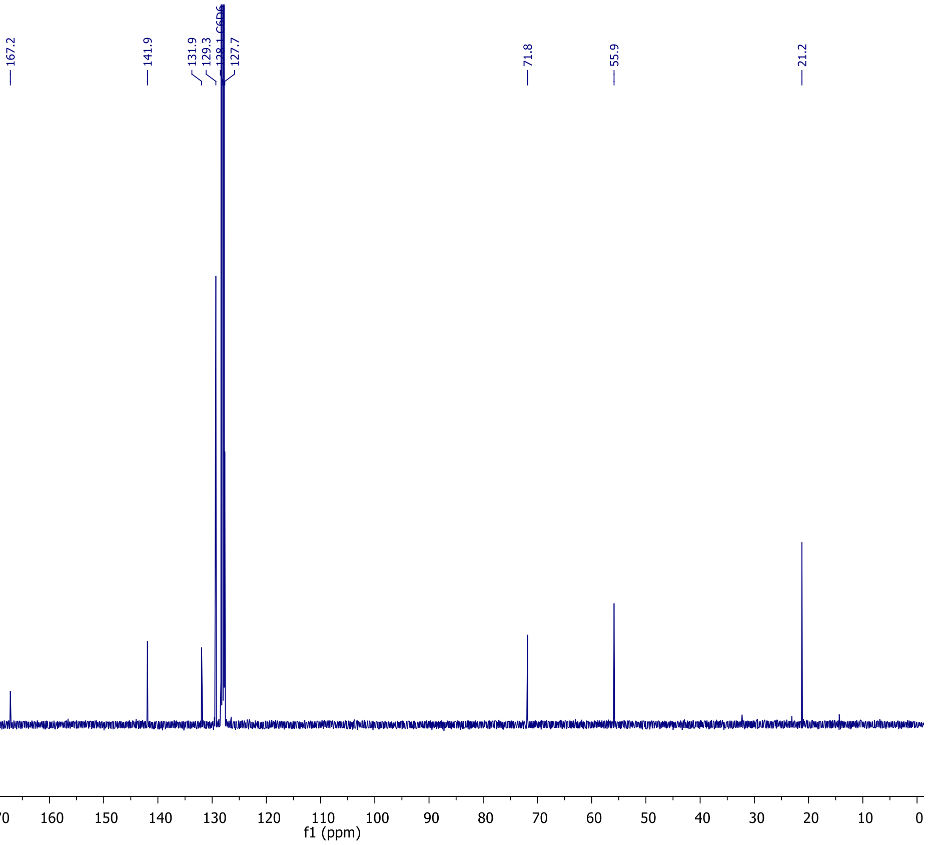

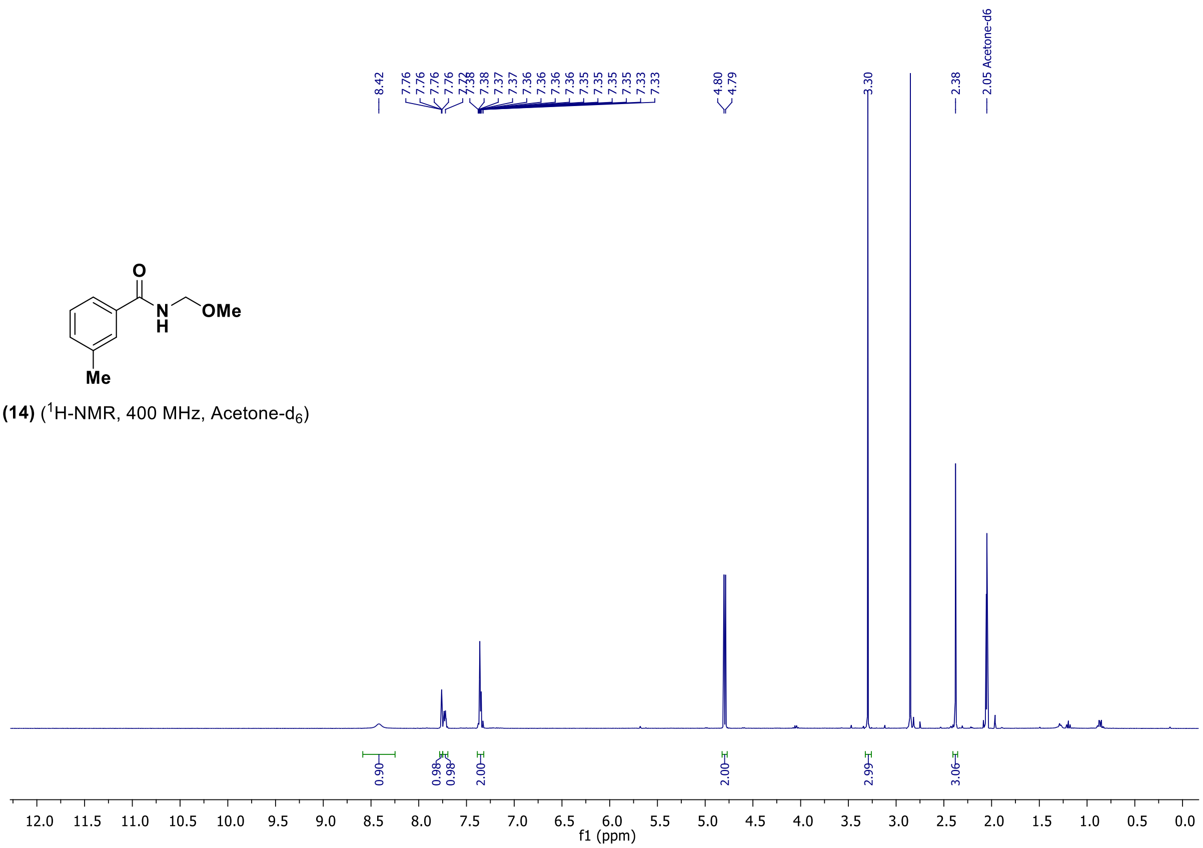

(14) (^{1}H-NMR, 400 MHz, Acetone-d$_6$)

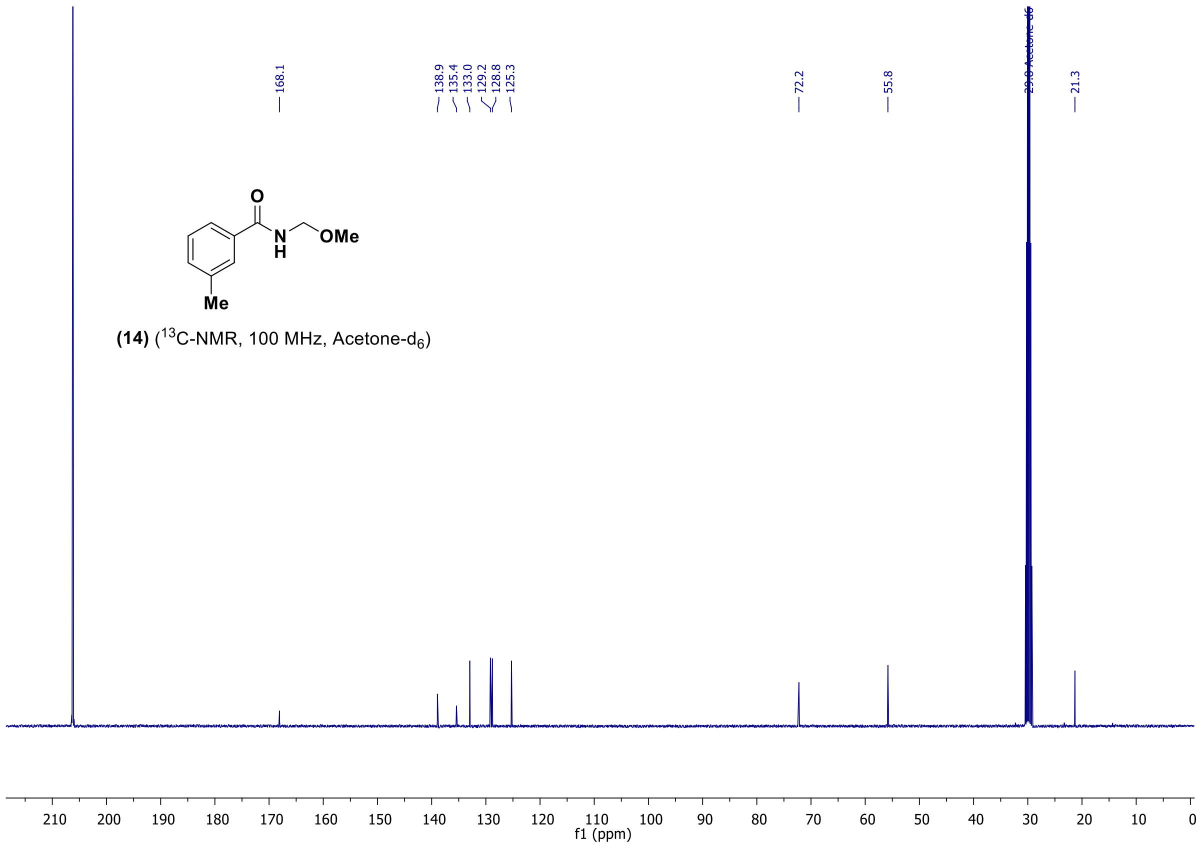

(14) (^{13}C-NMR, 100 MHz, Acetone-d$_6$)

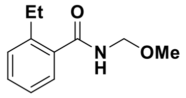

(15) (^{1}H-NMR, 400 MHz, $CDCl_3$)

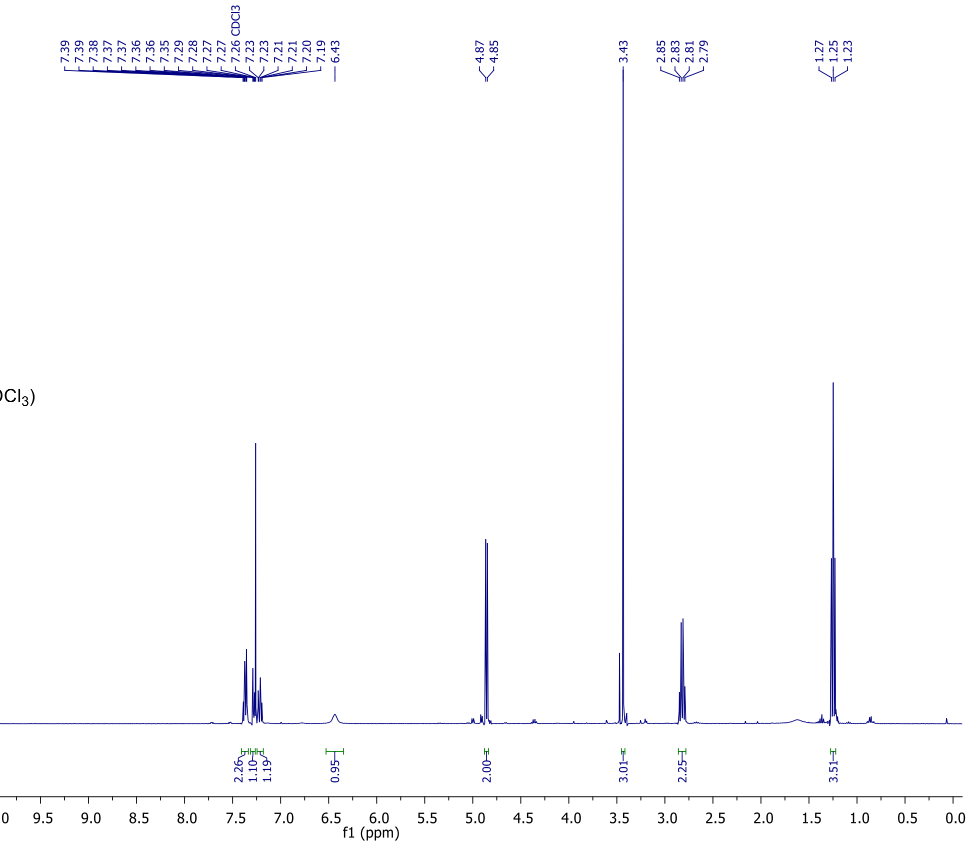

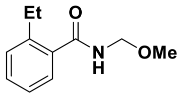

(15) (^{13}C-NMR, 100 MHz, $CDCl_3$)

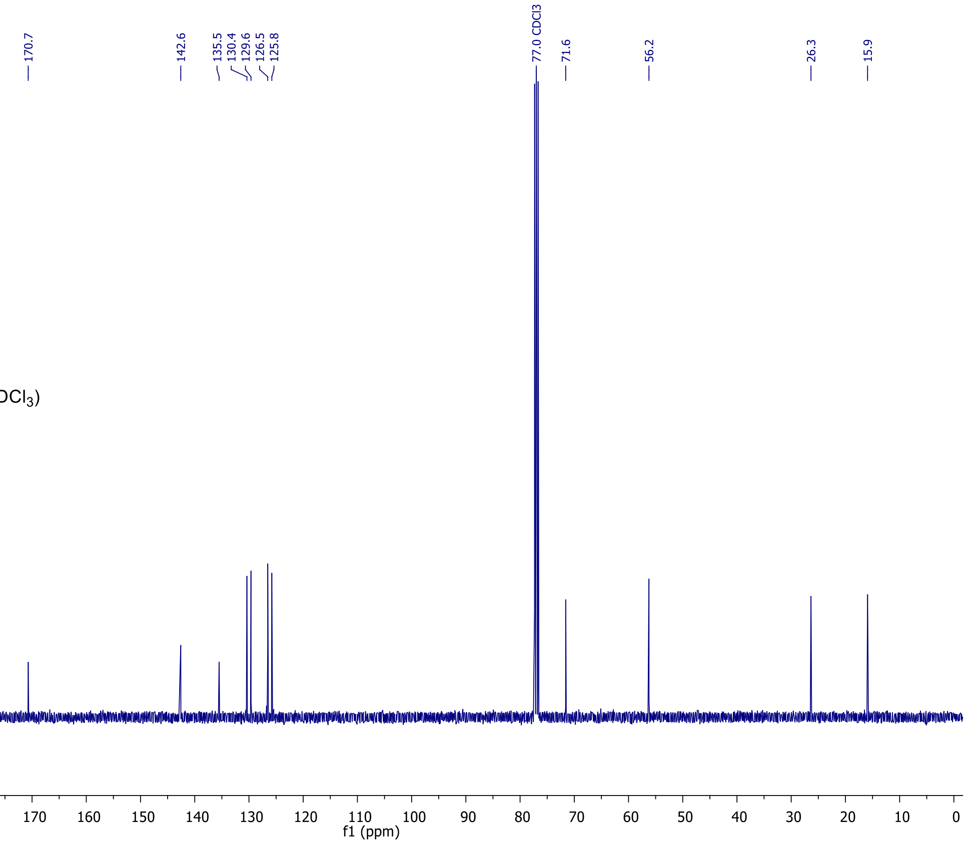

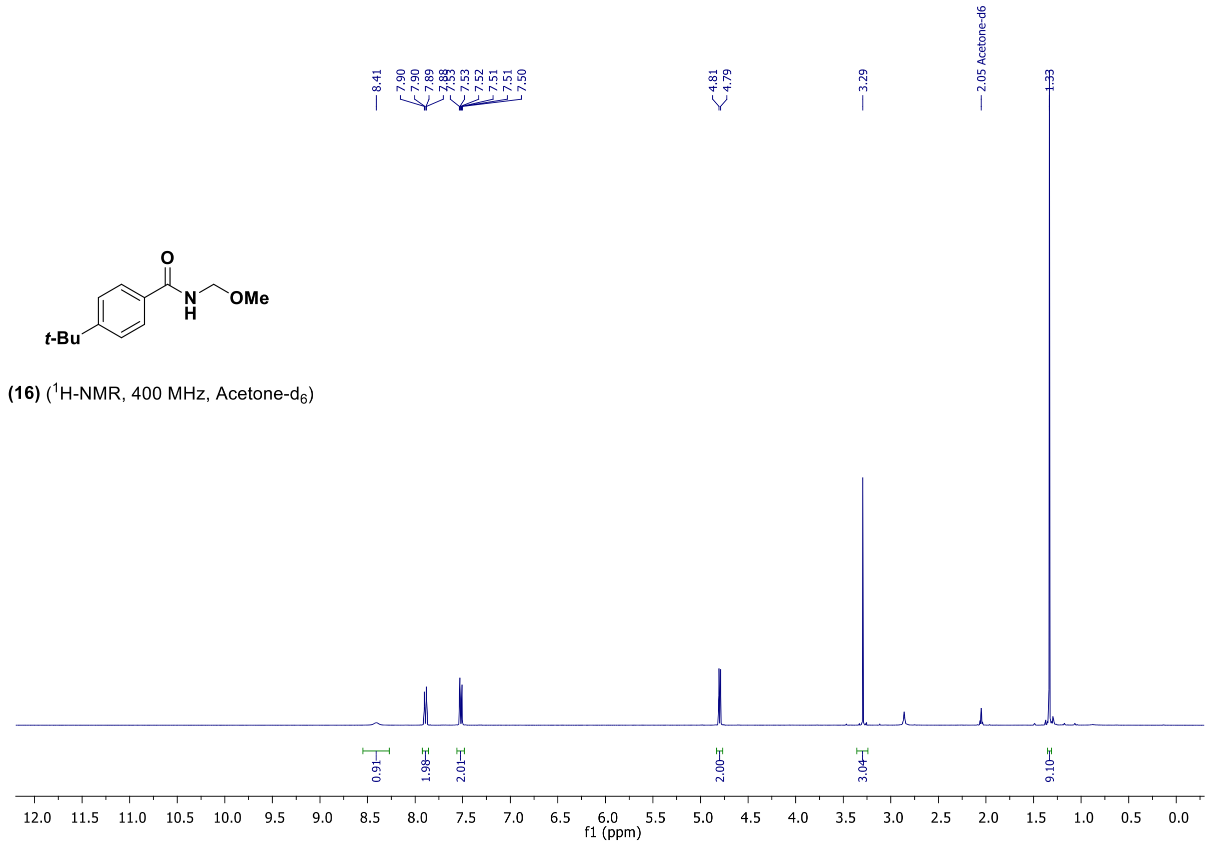

(16) (^{1}H-NMR, 400 MHz, Acetone-d_6)

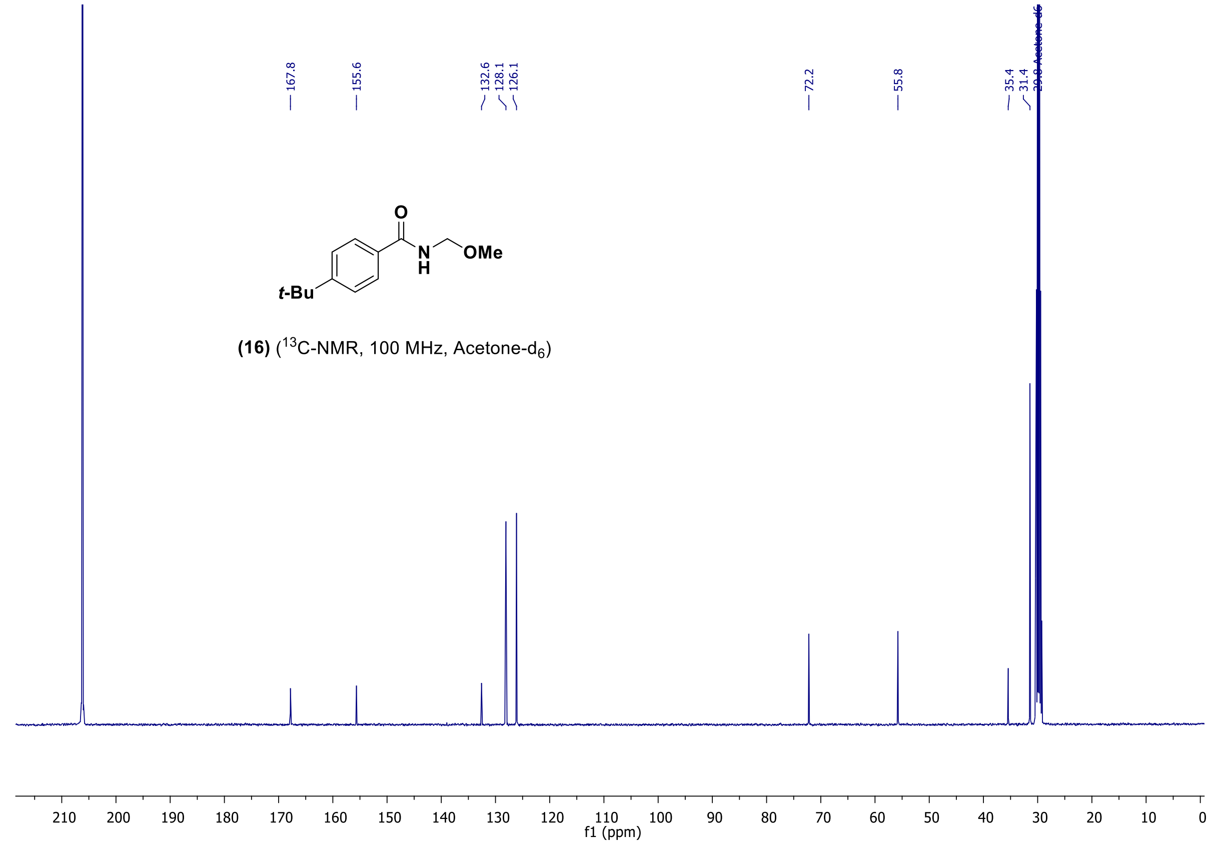

(16) (^{13}C-NMR, 100 MHz, Acetone-d_6)

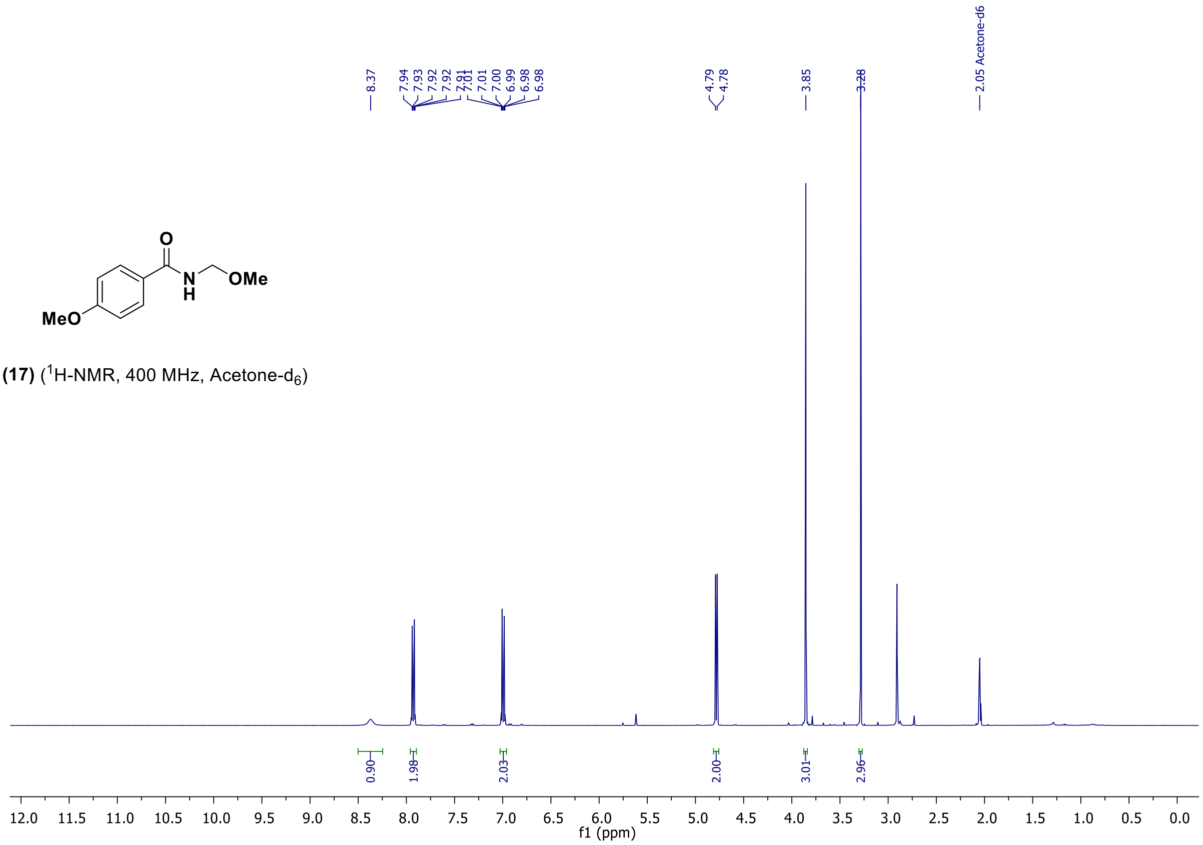

(17) (^{1}H-NMR, 400 MHz, Acetone-d_6)

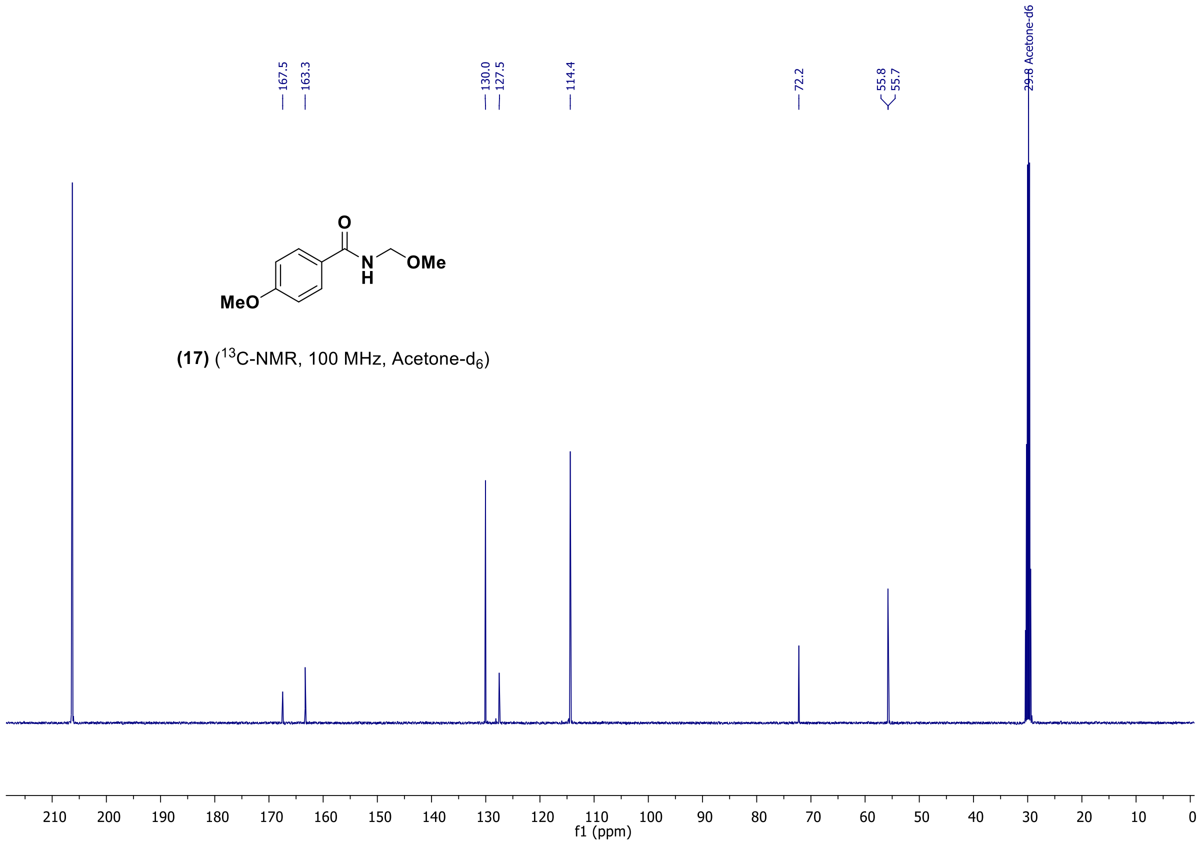

(17) (^{13}C-NMR, 100 MHz, Acetone-d_6)

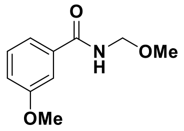

(18) (^{1}H-NMR, 400 MHz, $CDCl_3$)

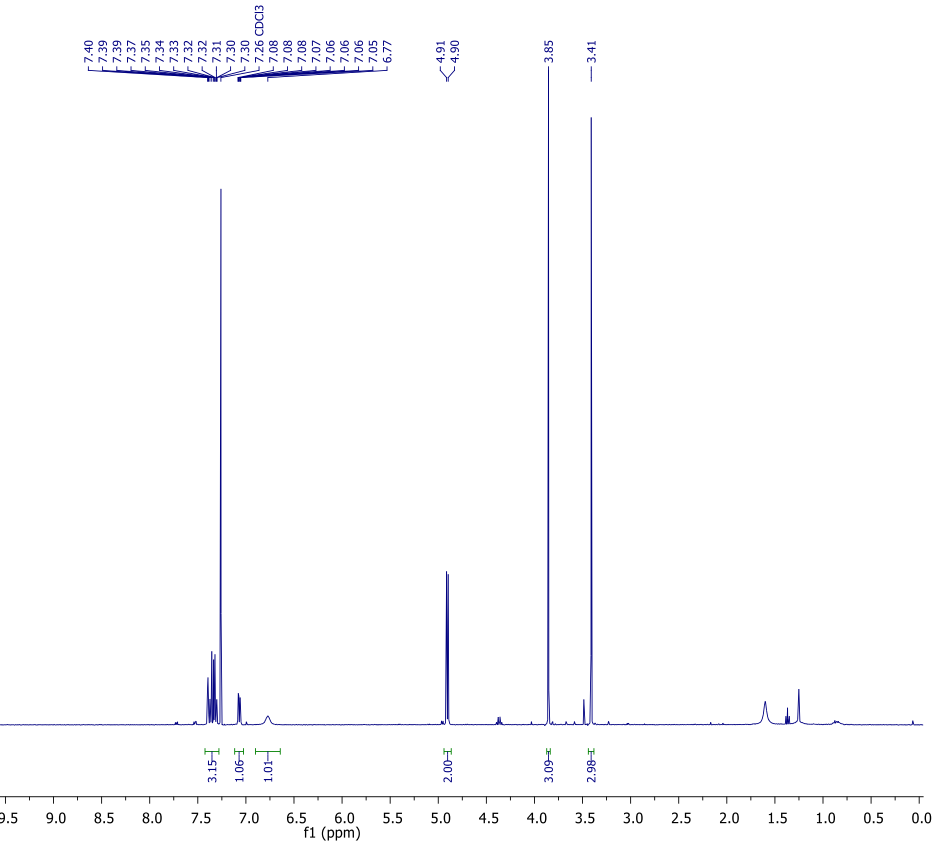

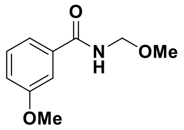

(18) (^{13}C-NMR, 100 MHz, $CDCl_3$)

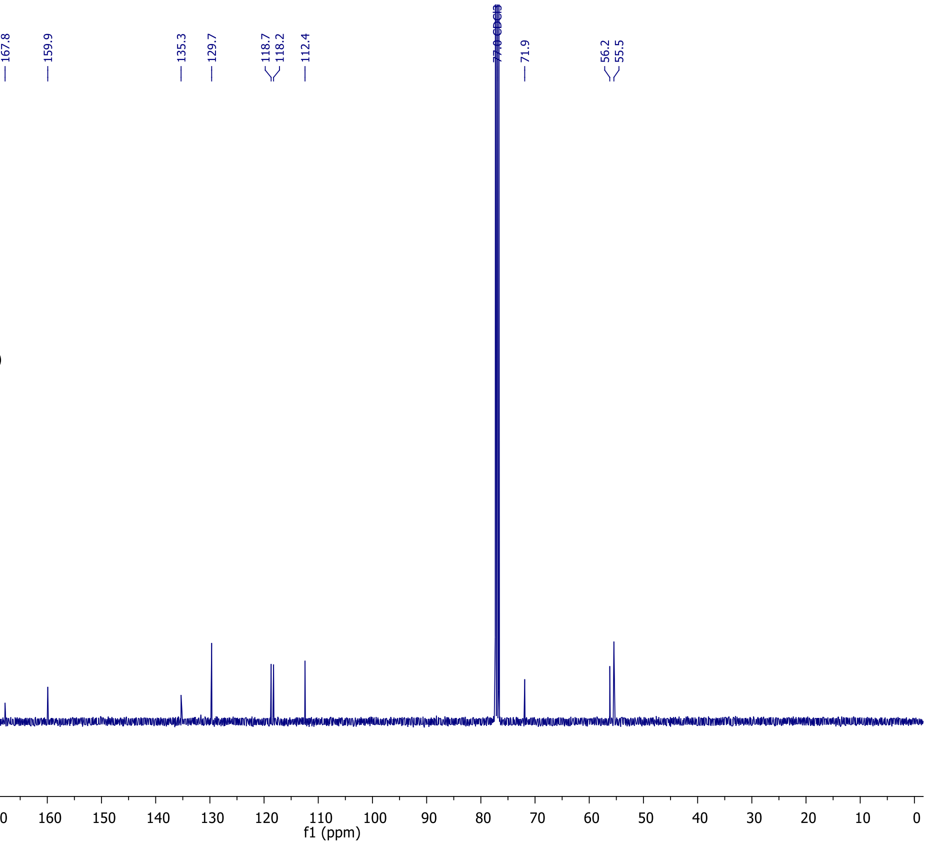

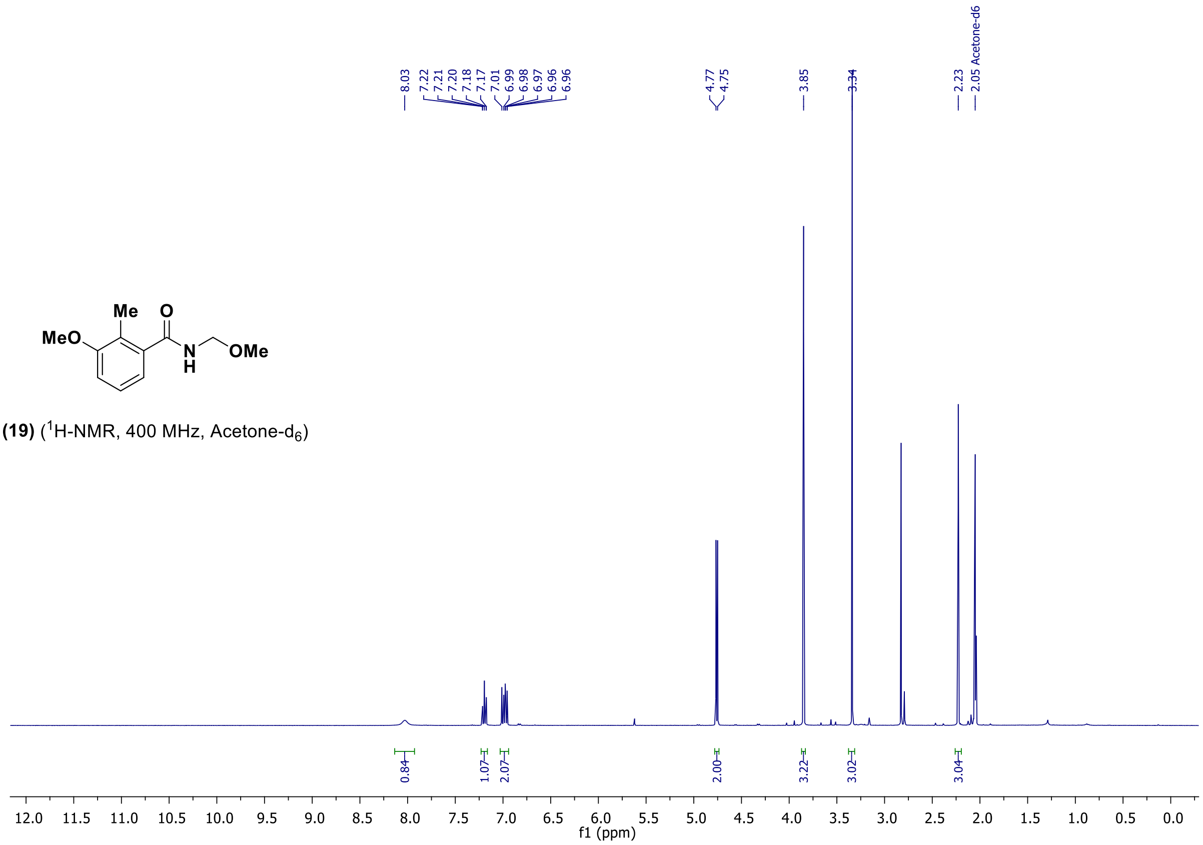

(19) (^{1}H-NMR, 400 MHz, Acetone-d_6)

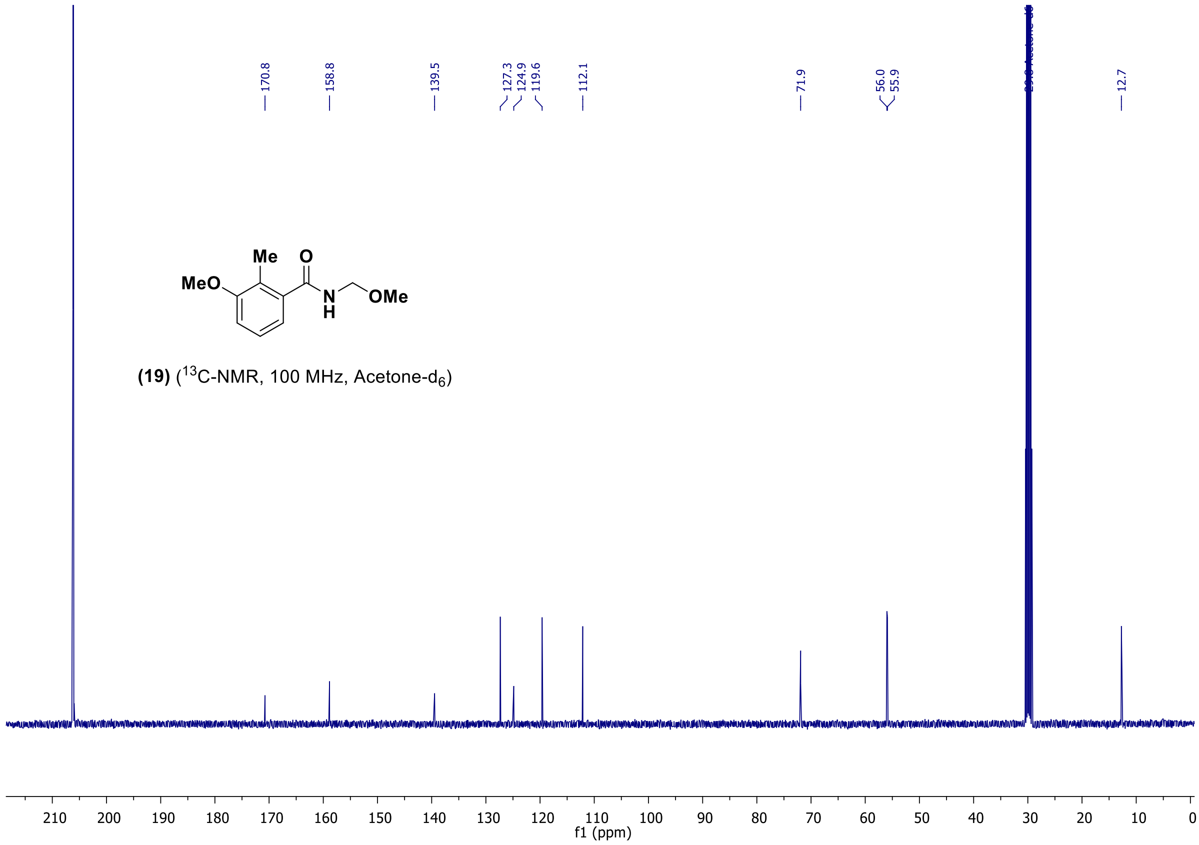

(19) (^{13}C-NMR, 100 MHz, Acetone-d_6)

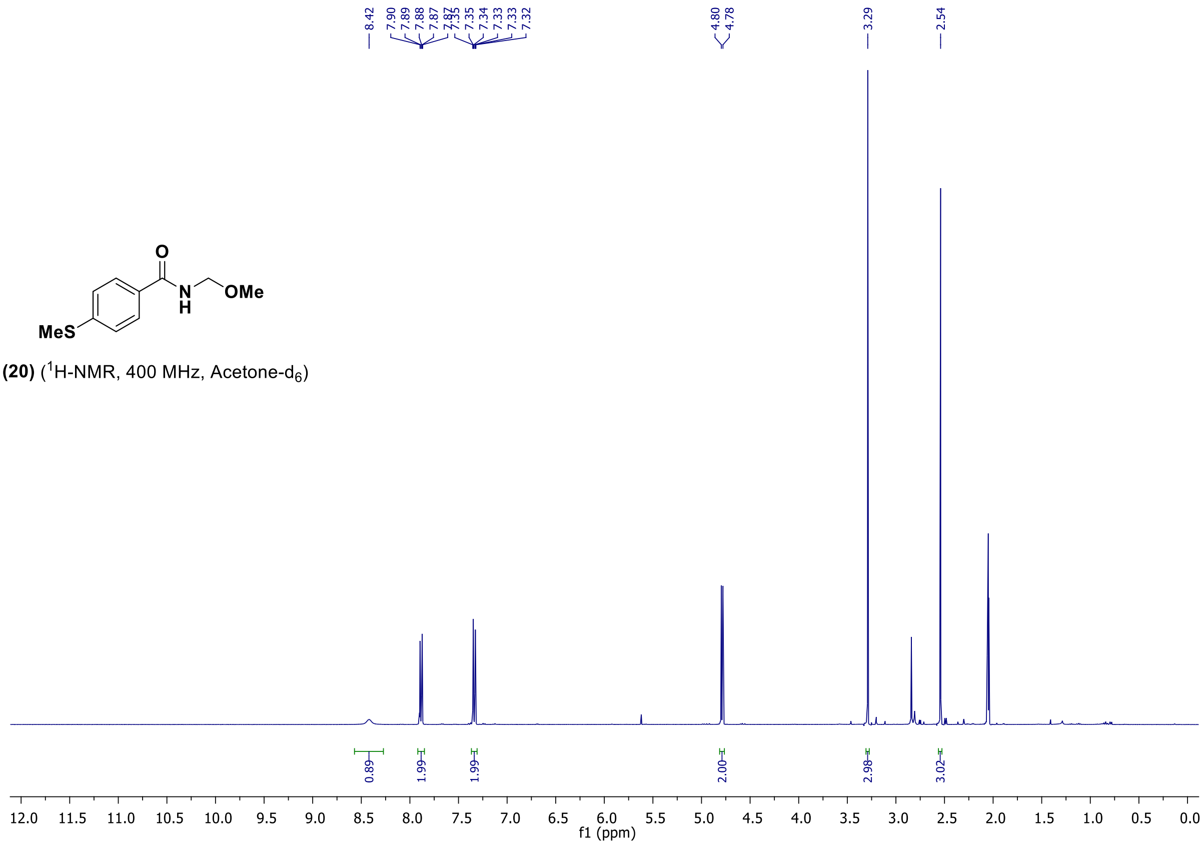

(20) (^{1}H-NMR, 400 MHz, Acetone-d_6)

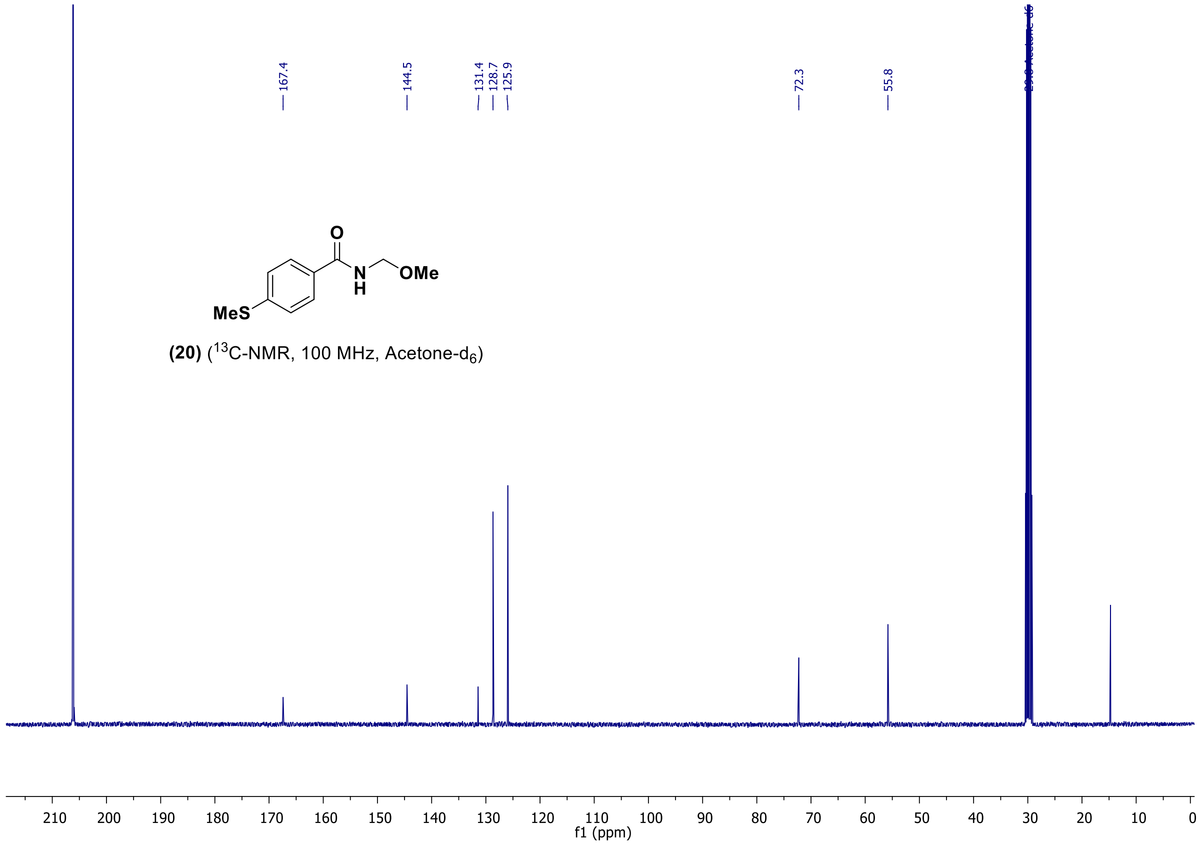

(20) (^{13}C-NMR, 100 MHz, Acetone-d_6)

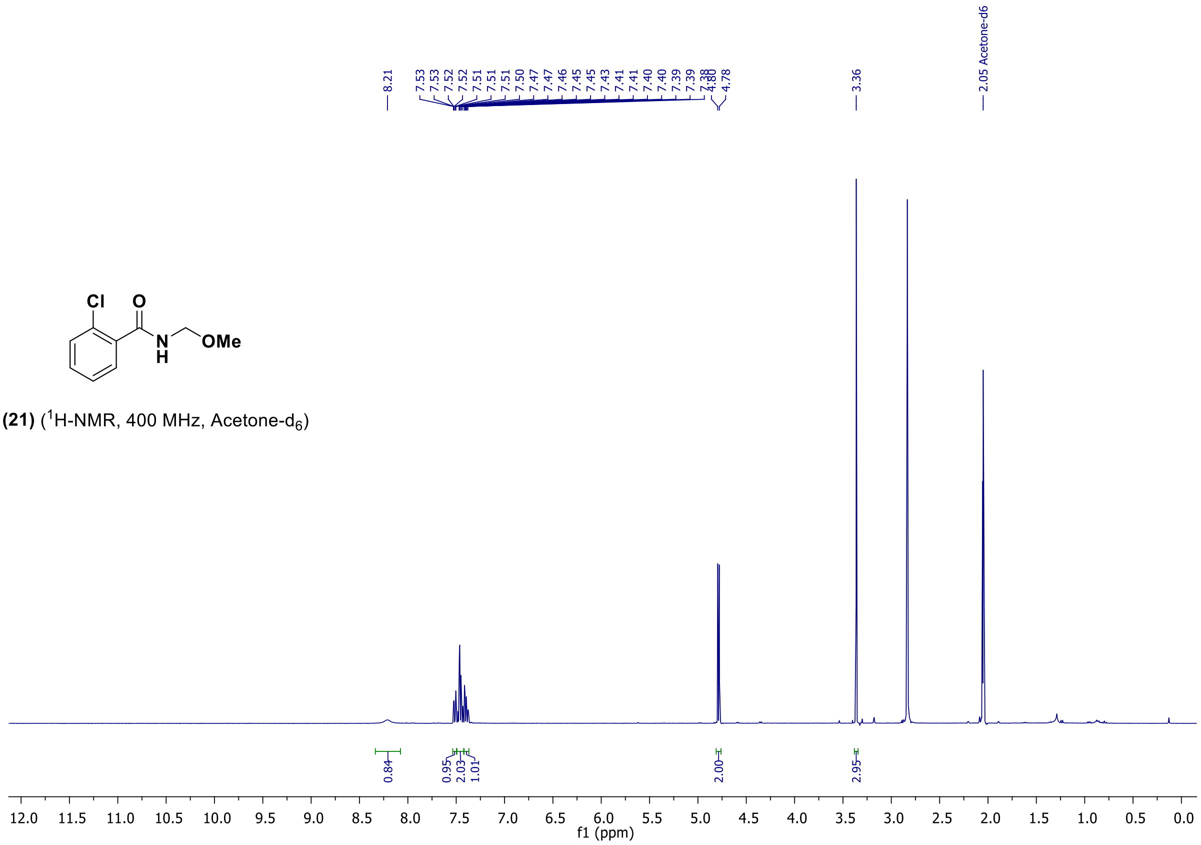

(21) (^{1}H-NMR, 400 MHz, Acetone-d_6)

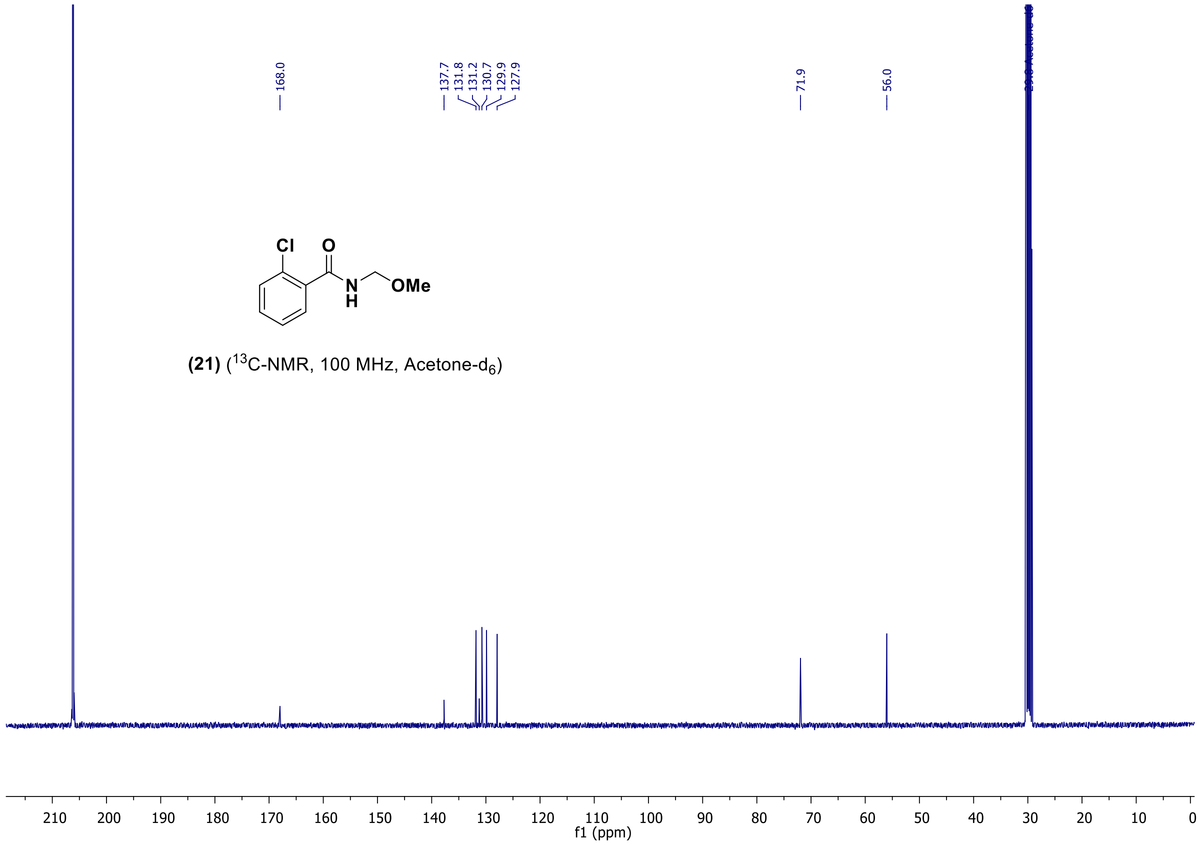

(21) (^{13}C-NMR, 100 MHz, Acetone-d_6)

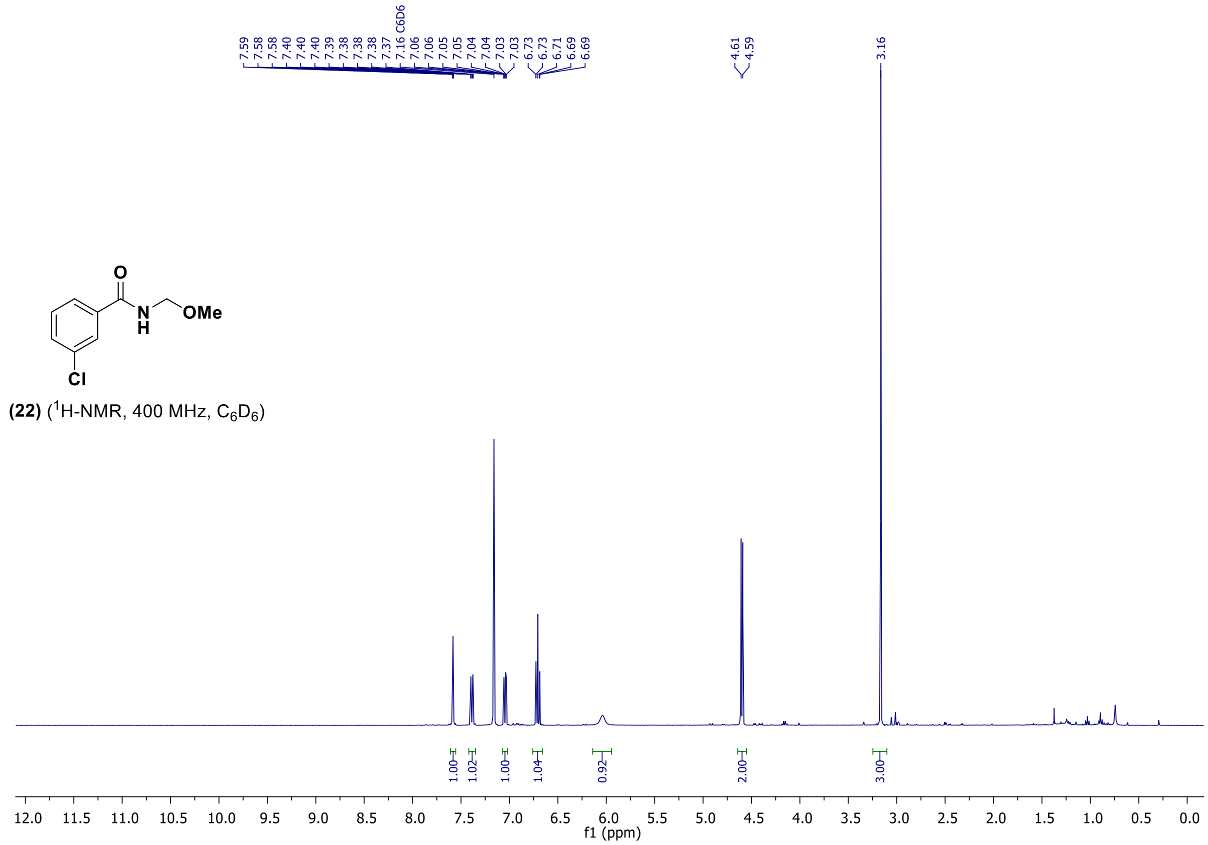

(22) (^{1}H-NMR, 400 MHz, C_6D_6)

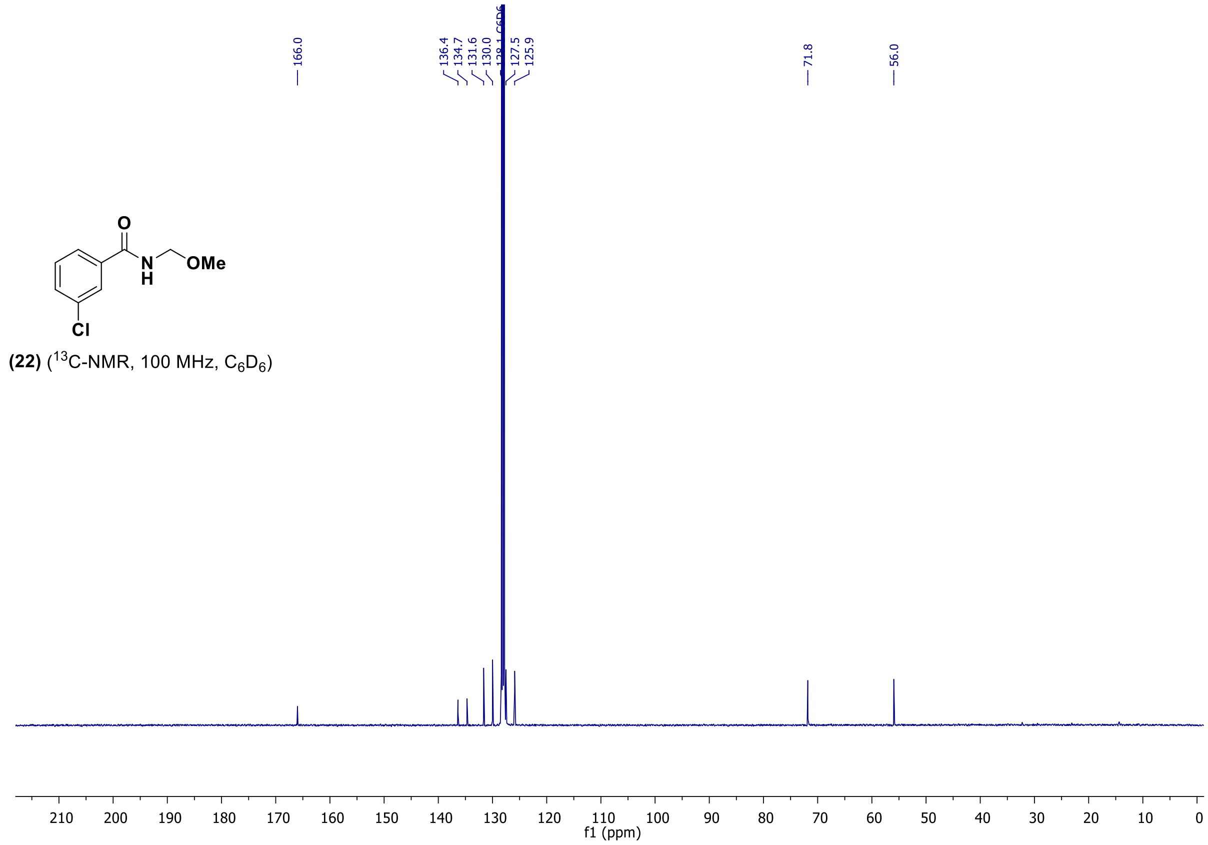

(22) (^{13}C-NMR, 100 MHz, C_6D_6)

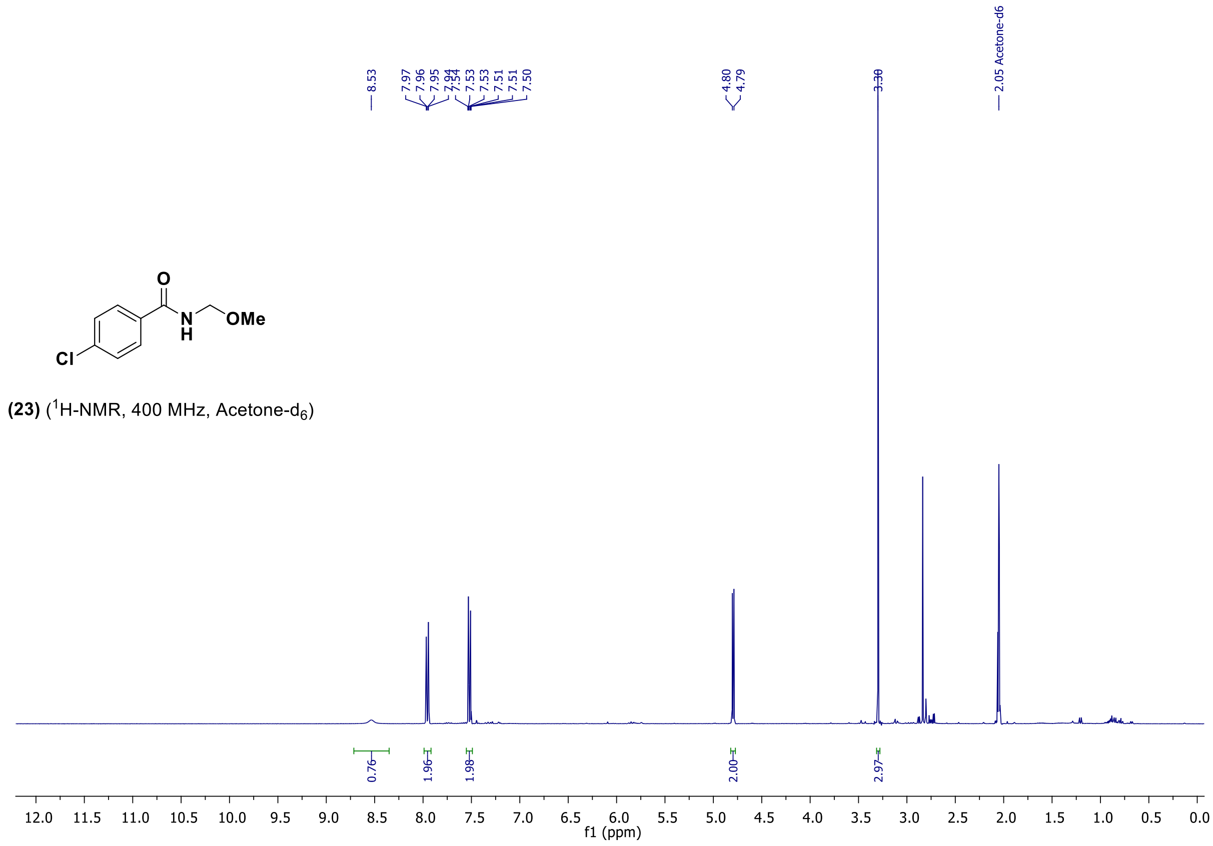

(23) (^{1}H-NMR, 400 MHz, Acetone-d$_6$)

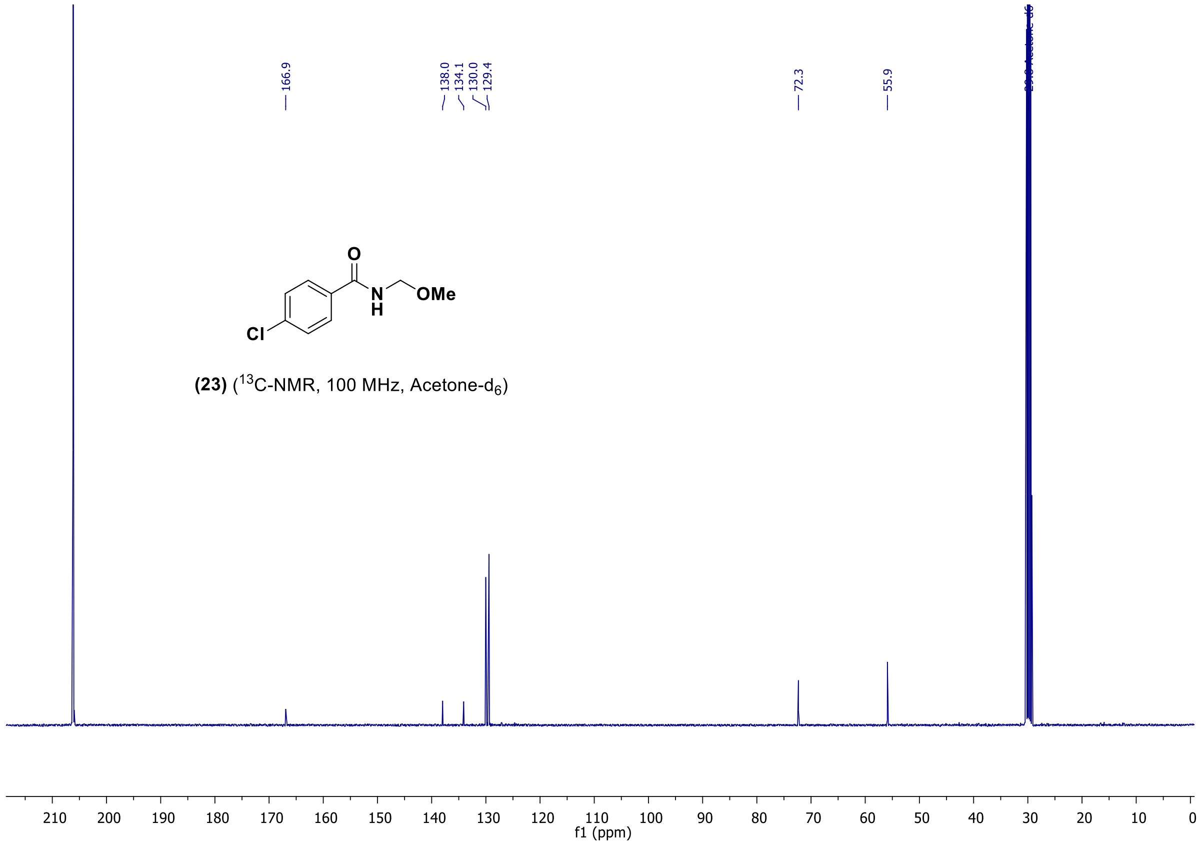

(23) (^{13}C-NMR, 100 MHz, Acetone-d$_6$)

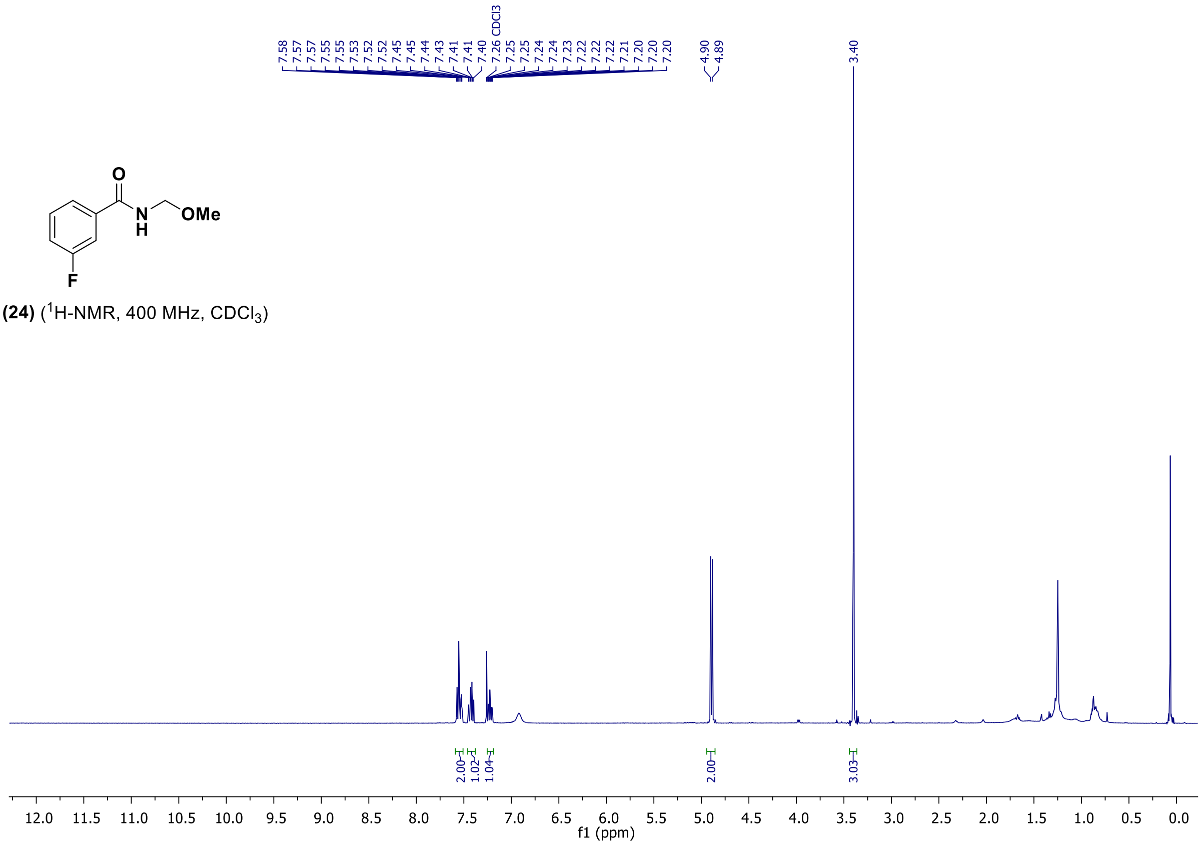

(24) (^{1}H-NMR, 400 MHz, $CDCl_3$)

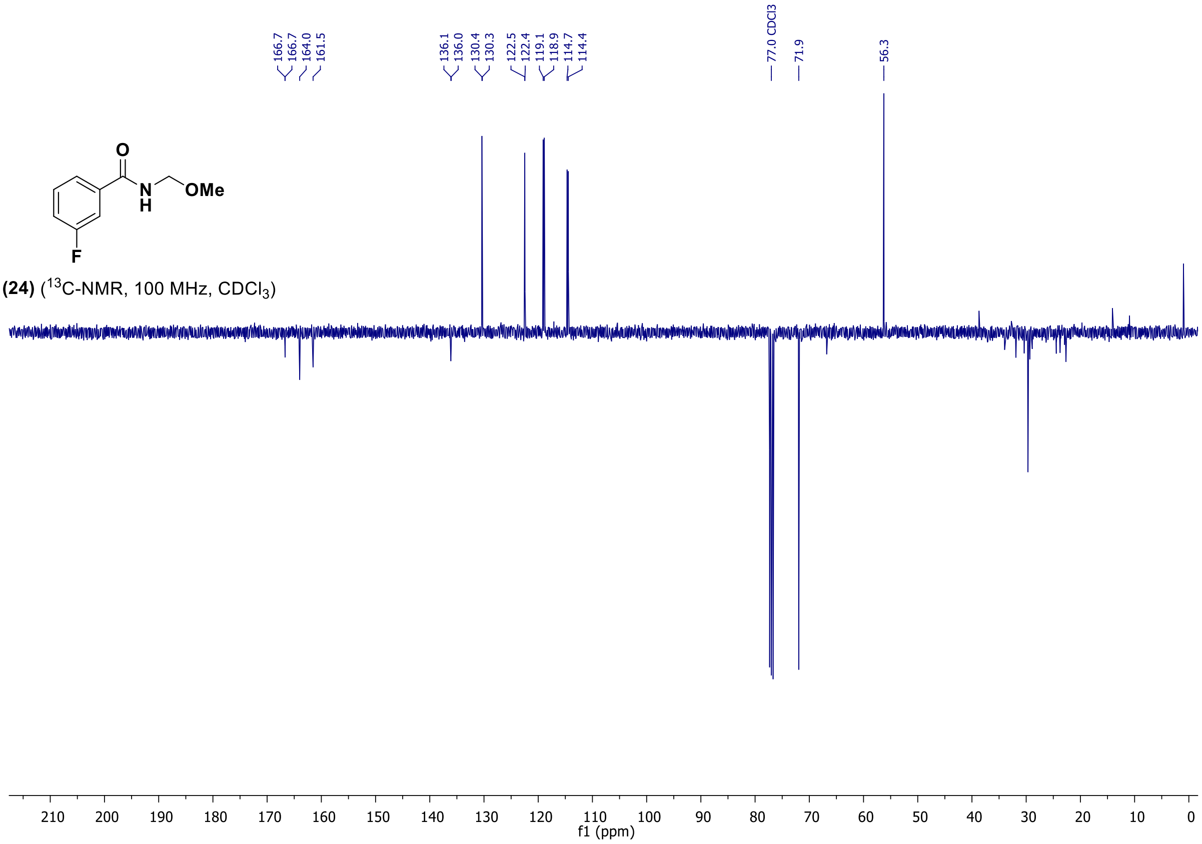

(24) (^{13}C-NMR, 100 MHz, $CDCl_3$)

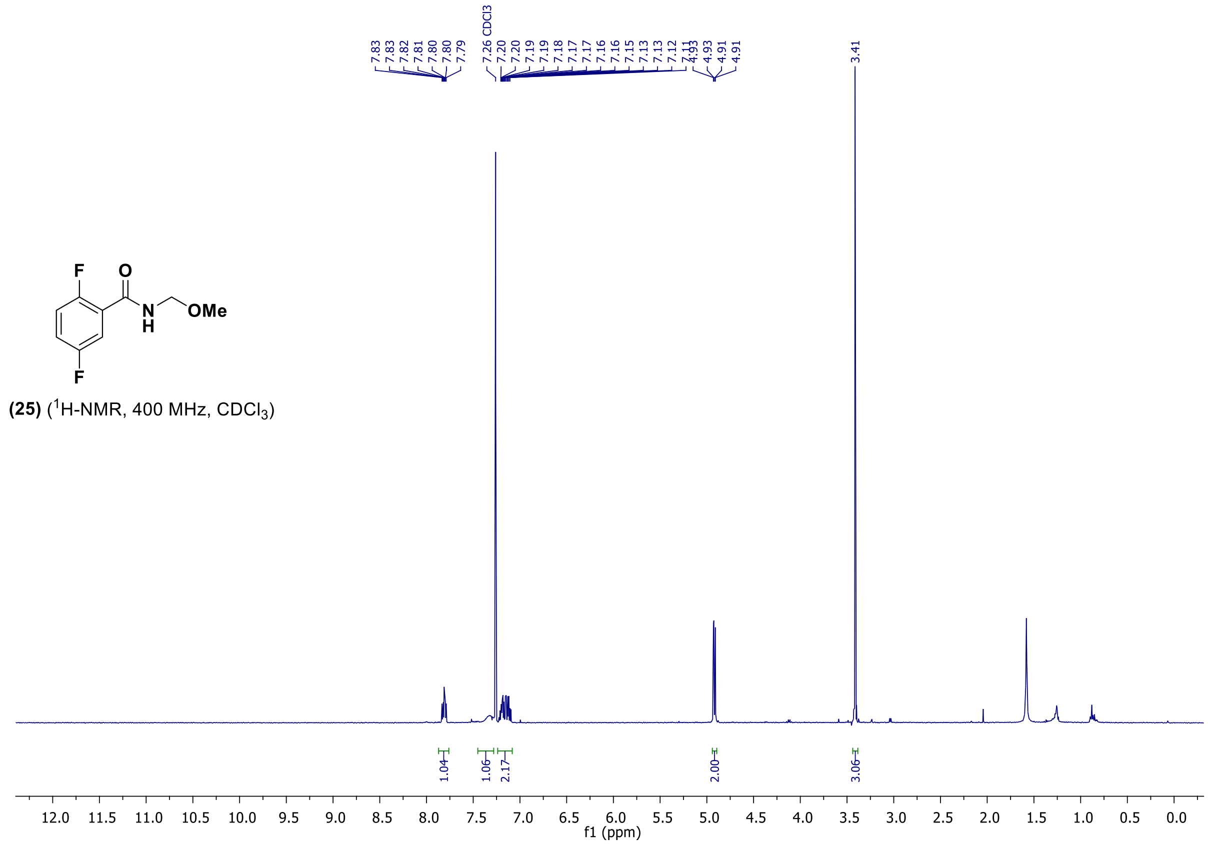

(25) (^{1}H-NMR, 400 MHz, $CDCl_3$)

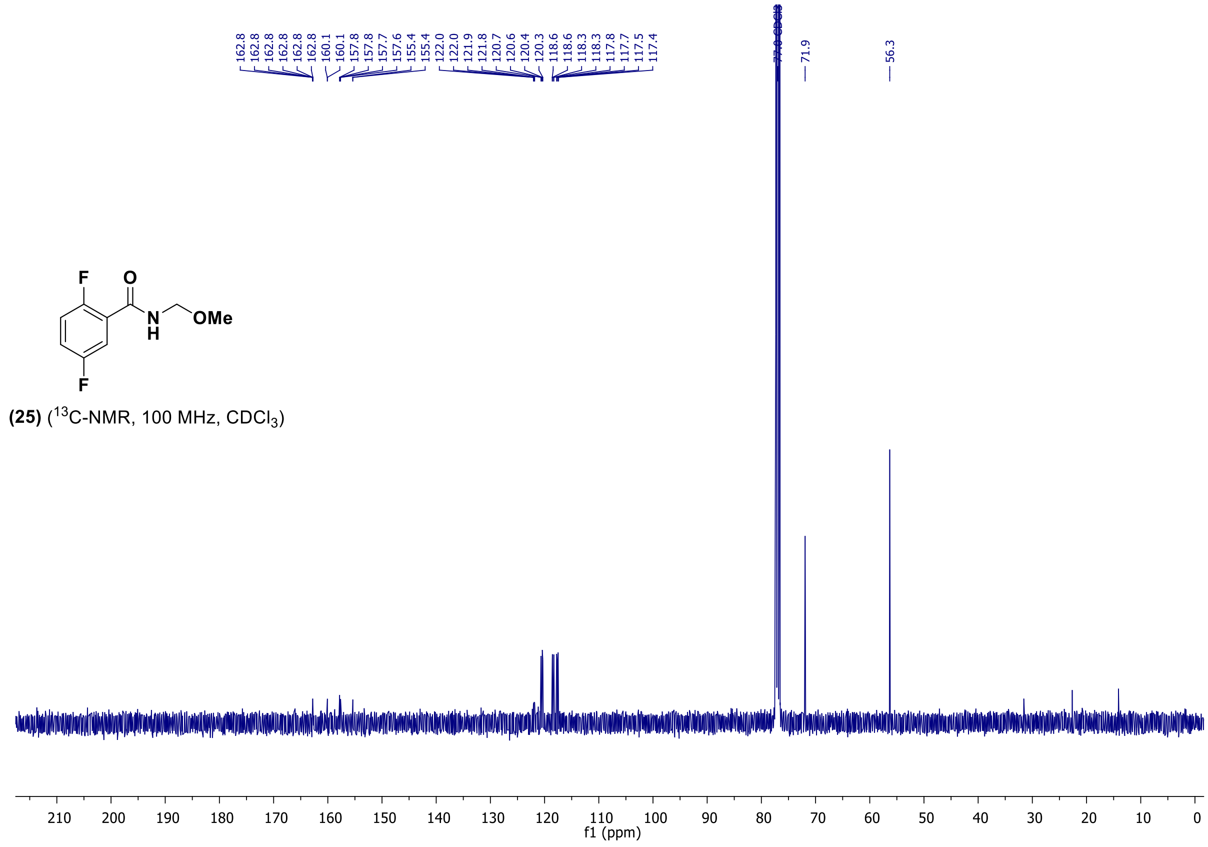

(25) (^{13}C-NMR, 100 MHz, $CDCl_3$)

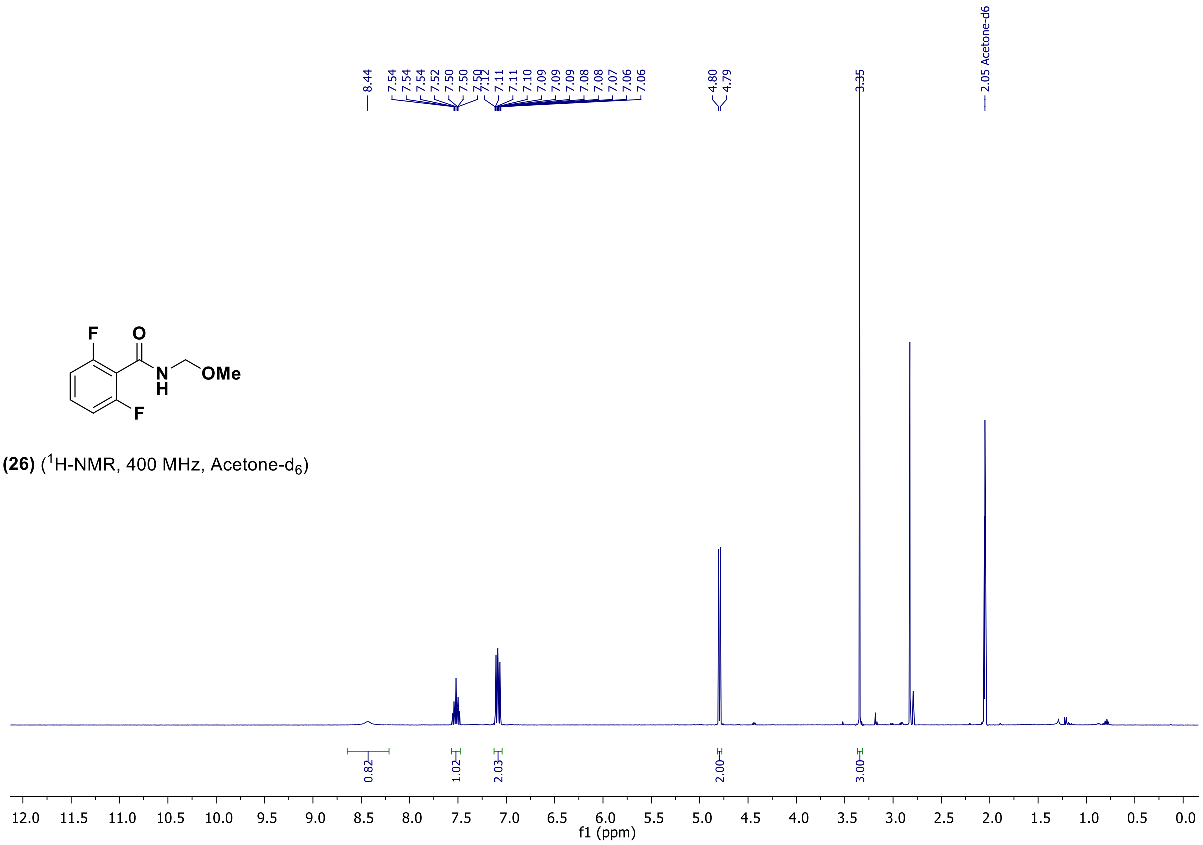

(26) (^{1}H-NMR, 400 MHz, Acetone-d_6)

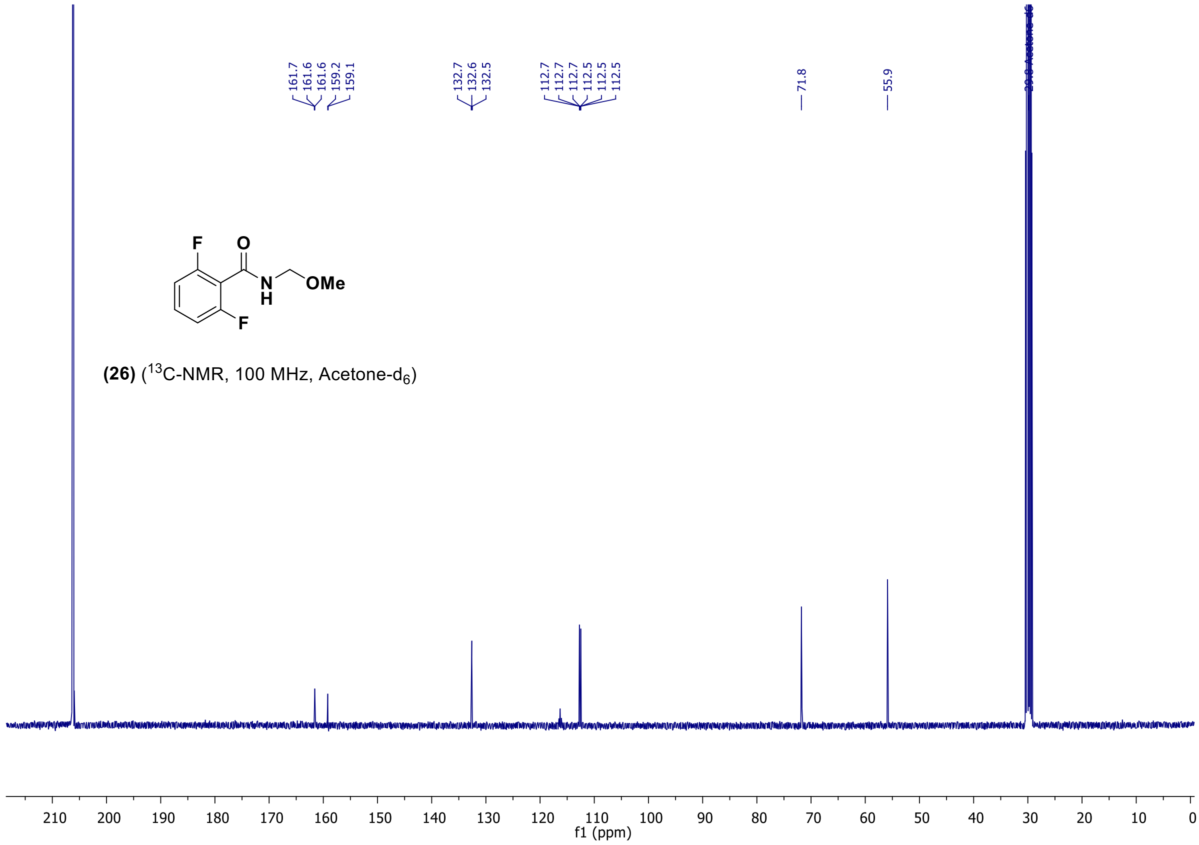

(26) (^{13}C-NMR, 100 MHz, Acetone-d_6)

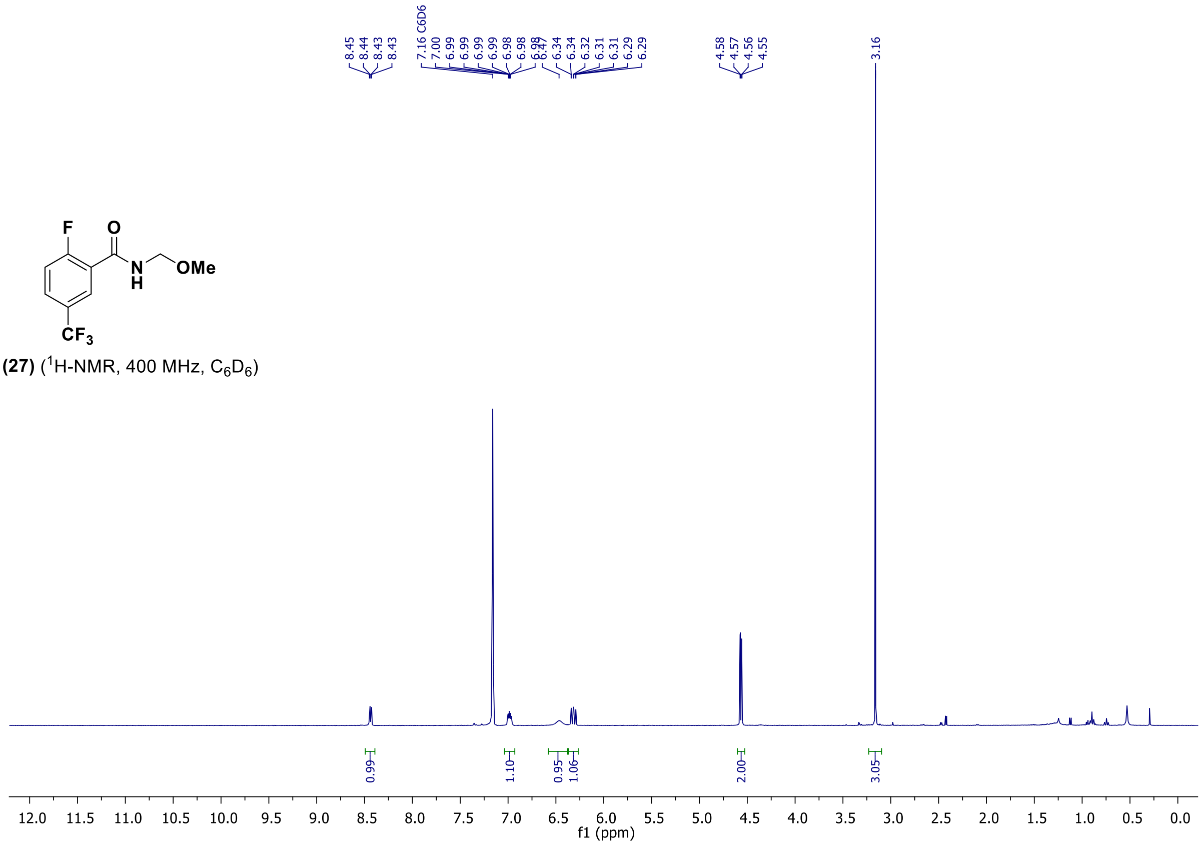

(27) (^{1}H-NMR, 400 MHz, C_6D_6)

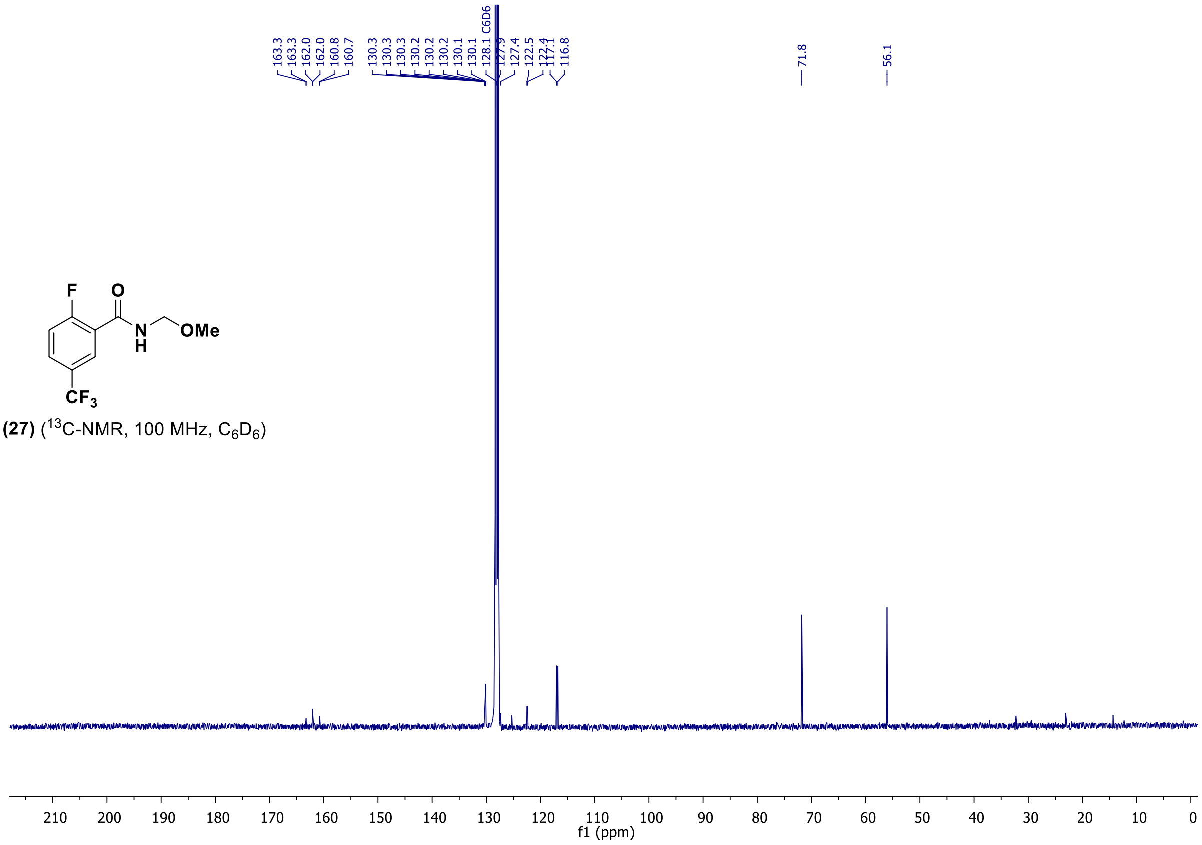

(27) (^{13}C-NMR, 100 MHz, C_6D_6)

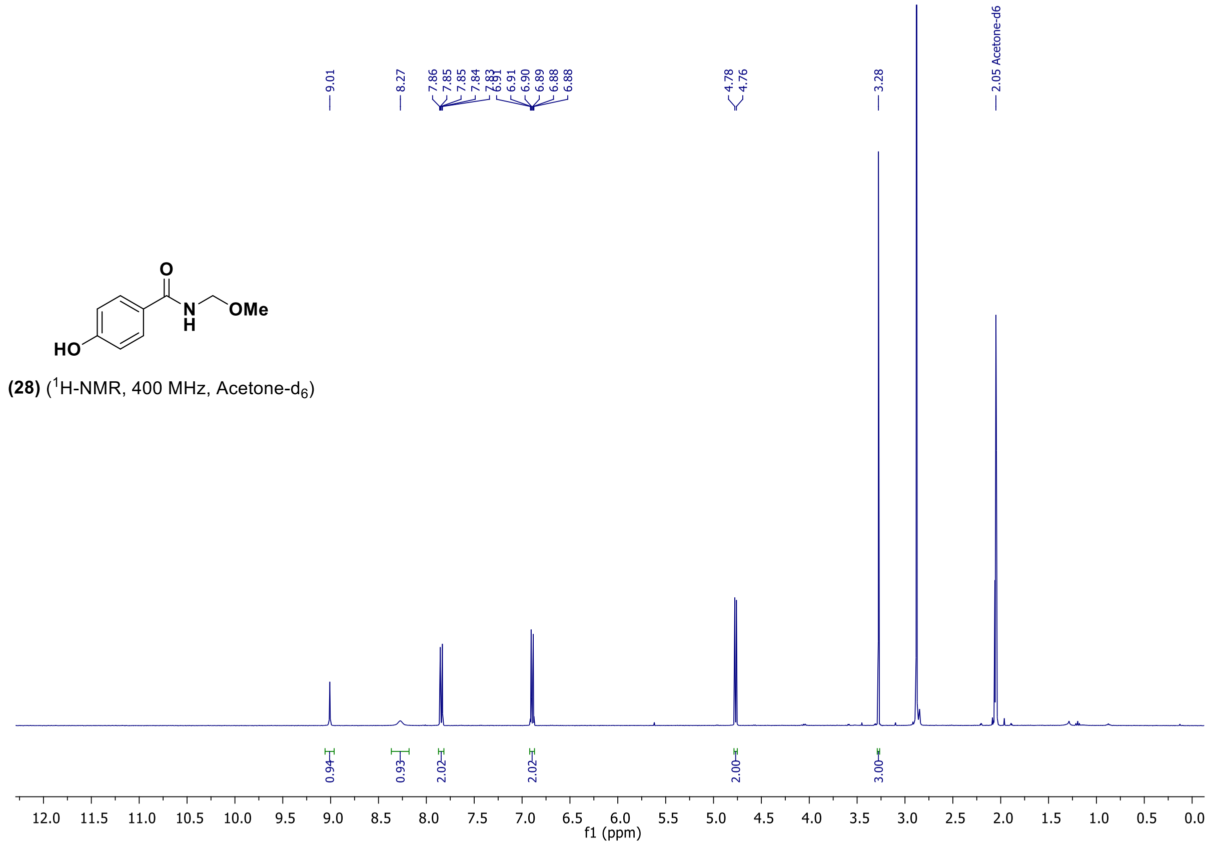

(28) (^{1}H-NMR, 400 MHz, Acetone-d_6)

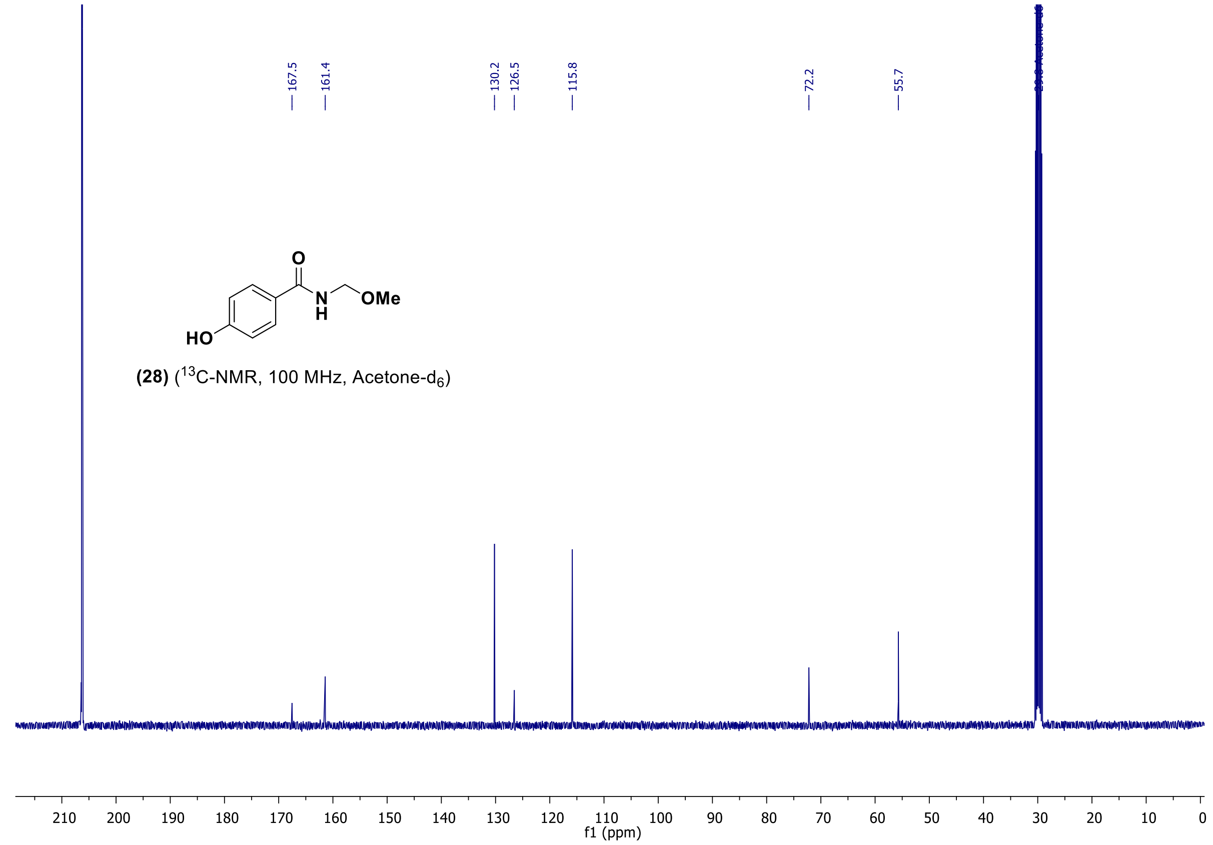

(28) (^{13}C-NMR, 100 MHz, Acetone-d_6)

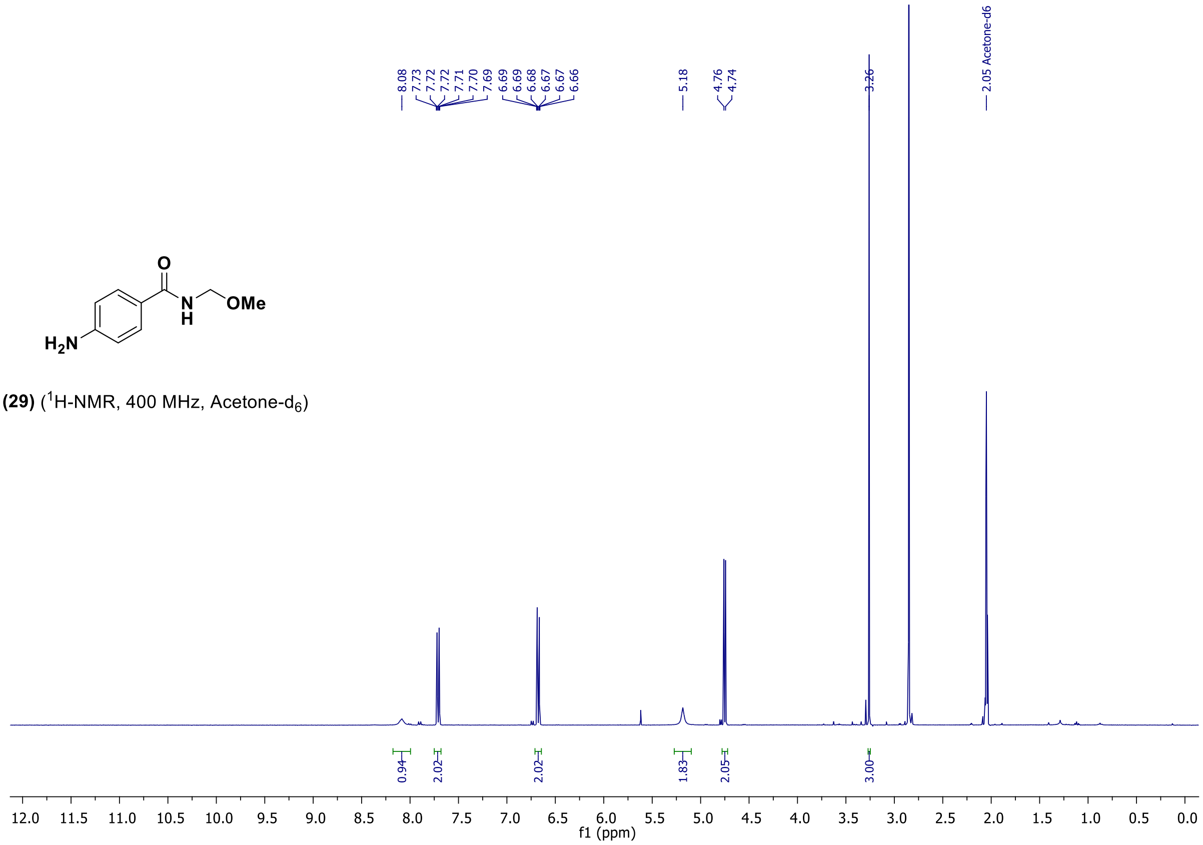

(29) (^{1}H-NMR, 400 MHz, Acetone-d_6)

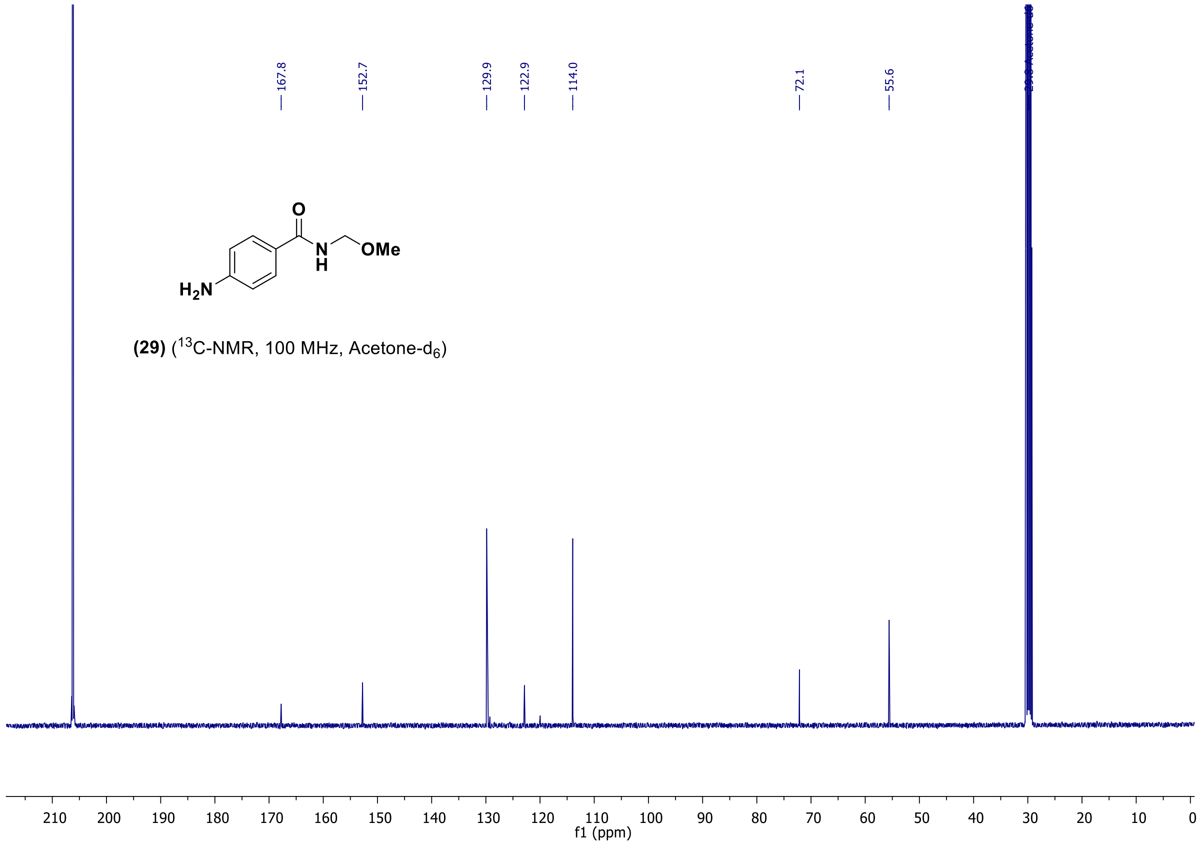

(29) (^{13}C-NMR, 100 MHz, Acetone-d_6)

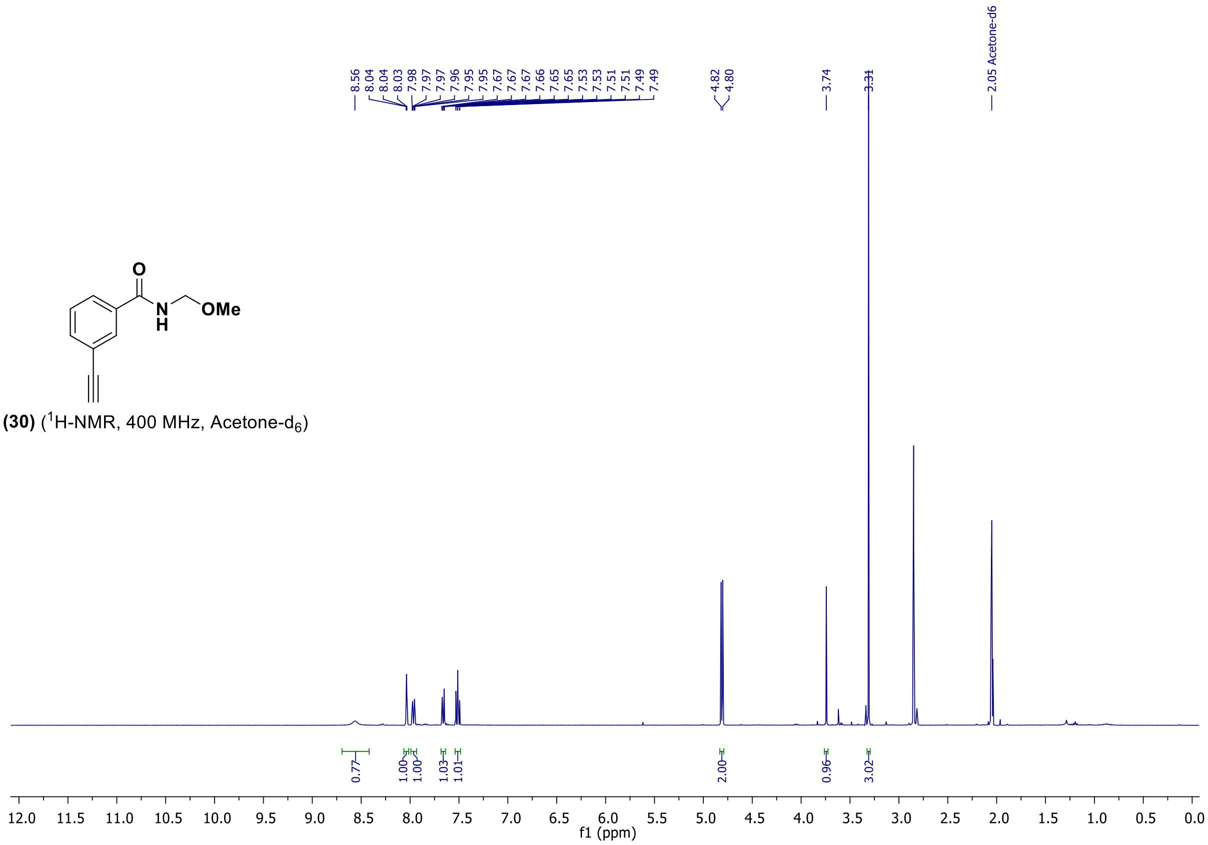

(30) (^{1}H-NMR, 400 MHz, Acetone-d_6)

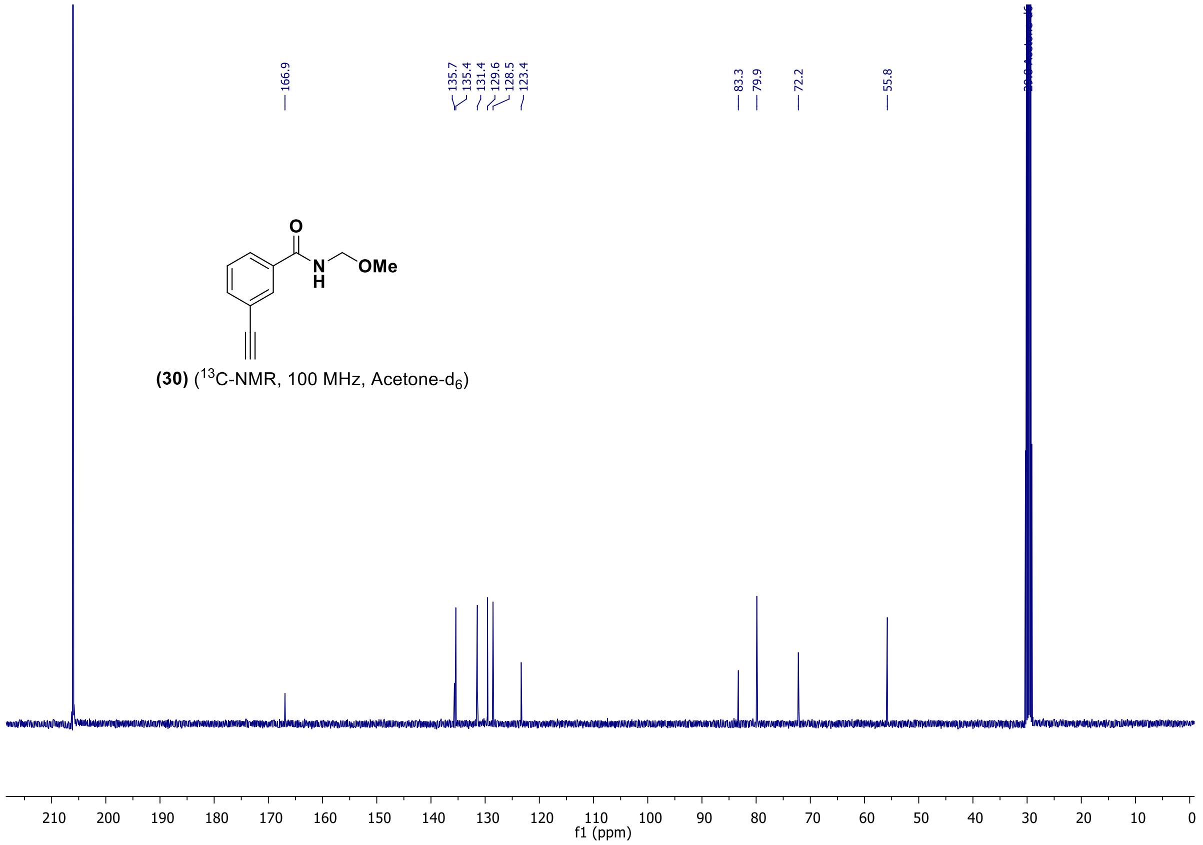

(30) (^{13}C-NMR, 100 MHz, Acetone-d_6)

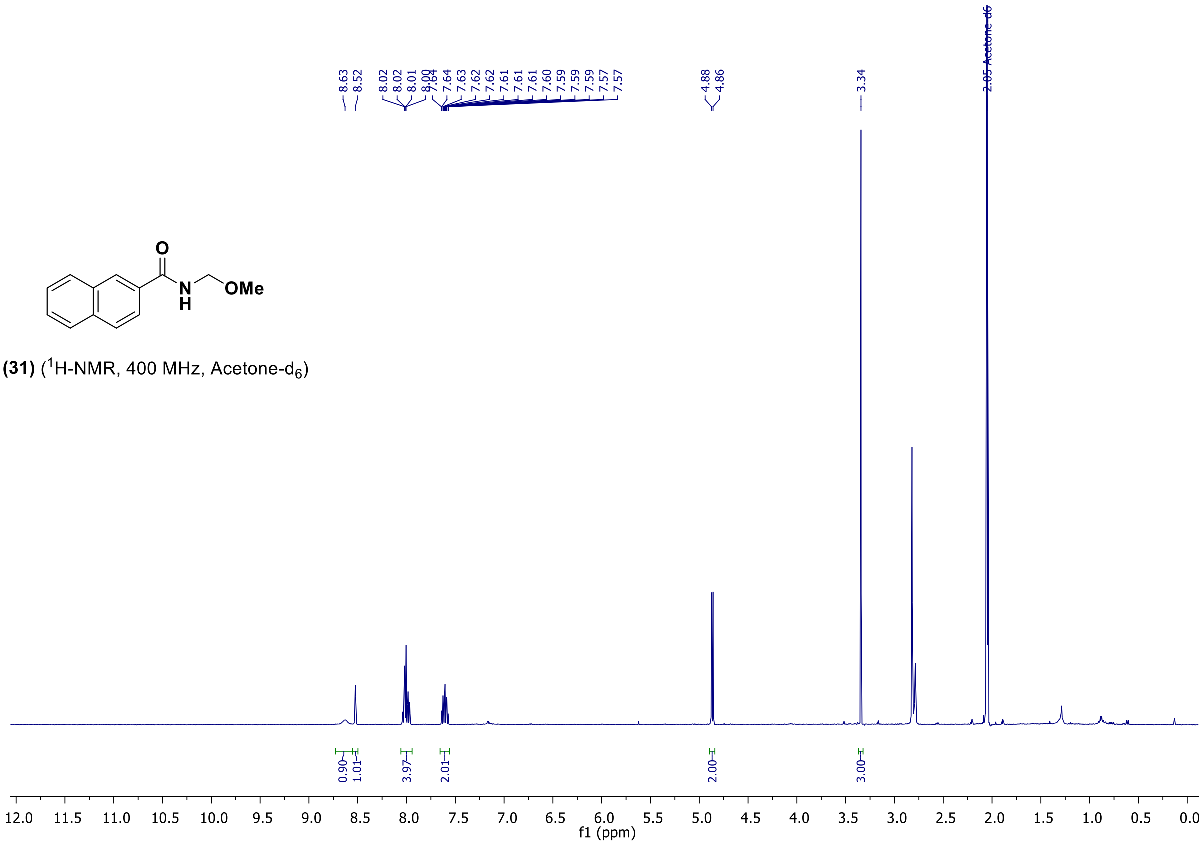

(31) (^{1}H-NMR, 400 MHz, Acetone-d$_6$)

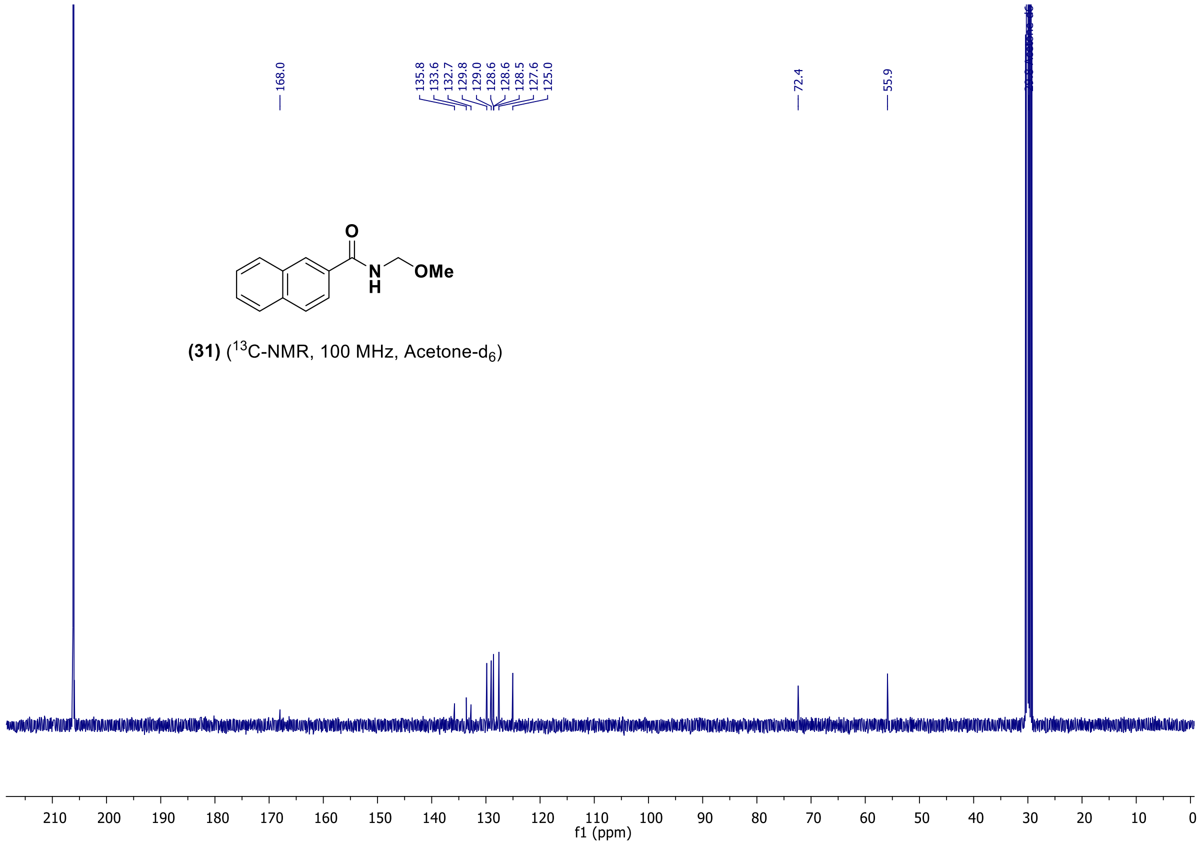

(31) (^{13}C-NMR, 100 MHz, Acetone-d$_6$)

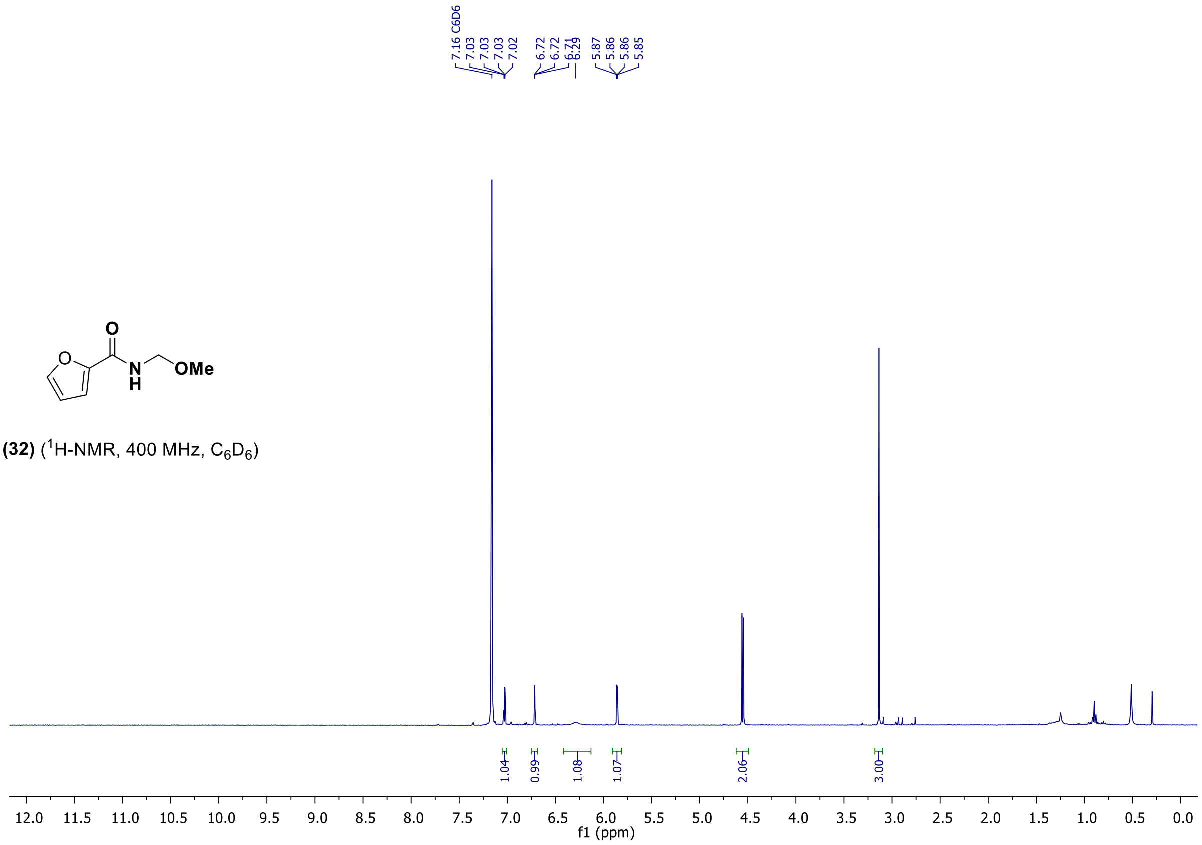

(32) (^{1}H-NMR, 400 MHz, C_6D_6)

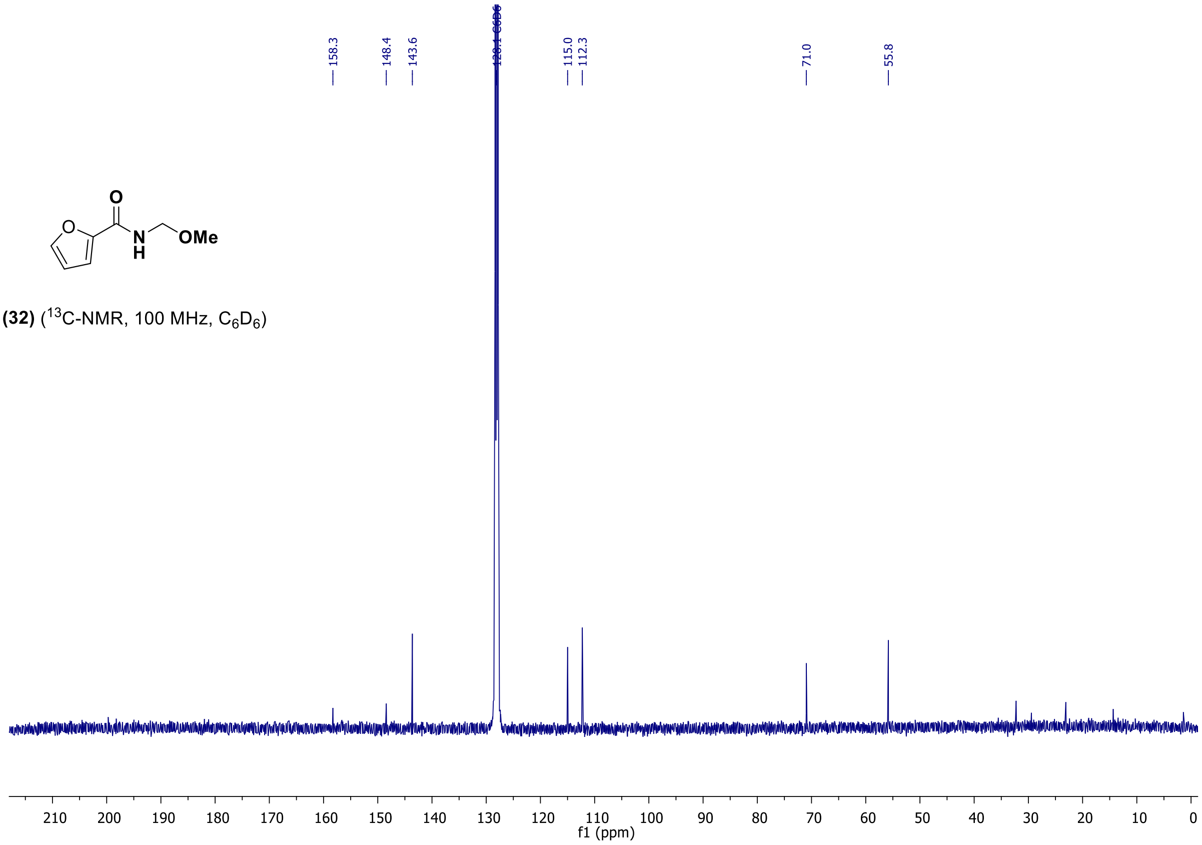

(32) (^{13}C-NMR, 100 MHz, C_6D_6)

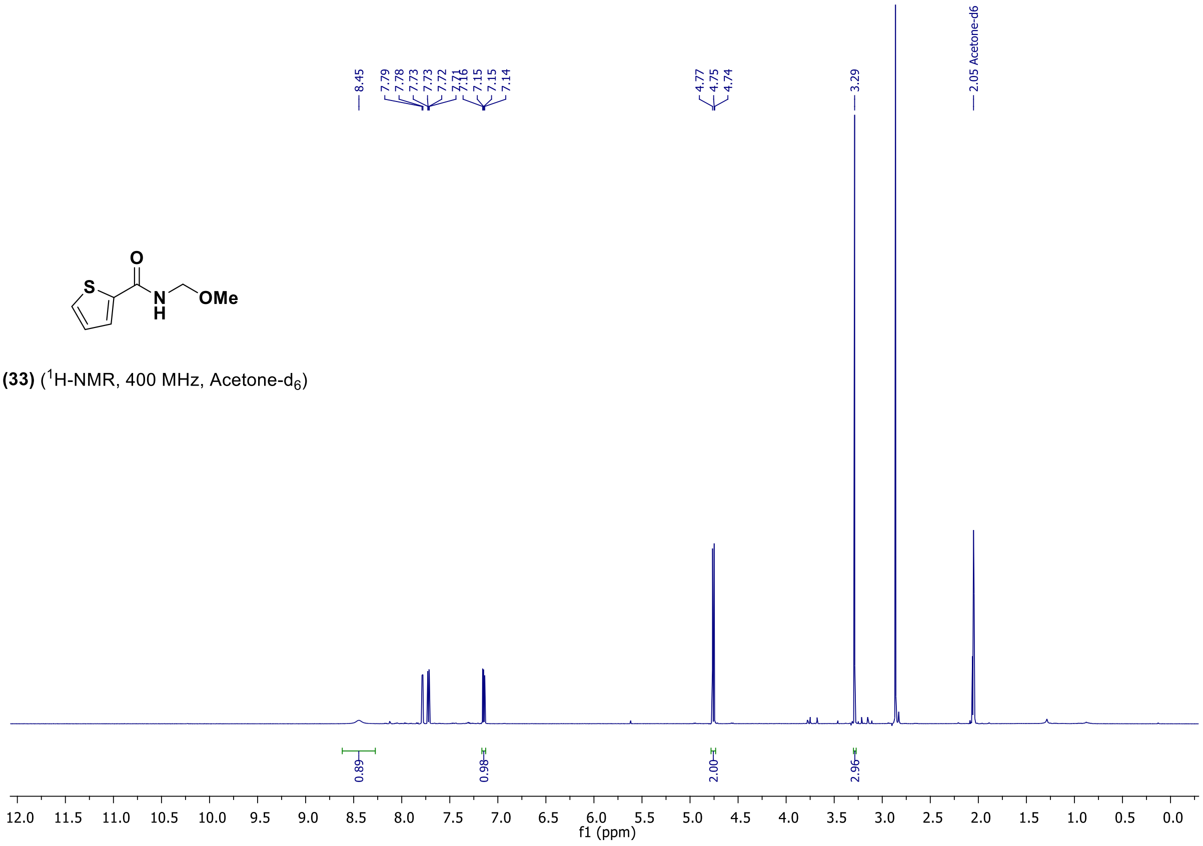

(33) (^{1}H-NMR, 400 MHz, Acetone-d_6)

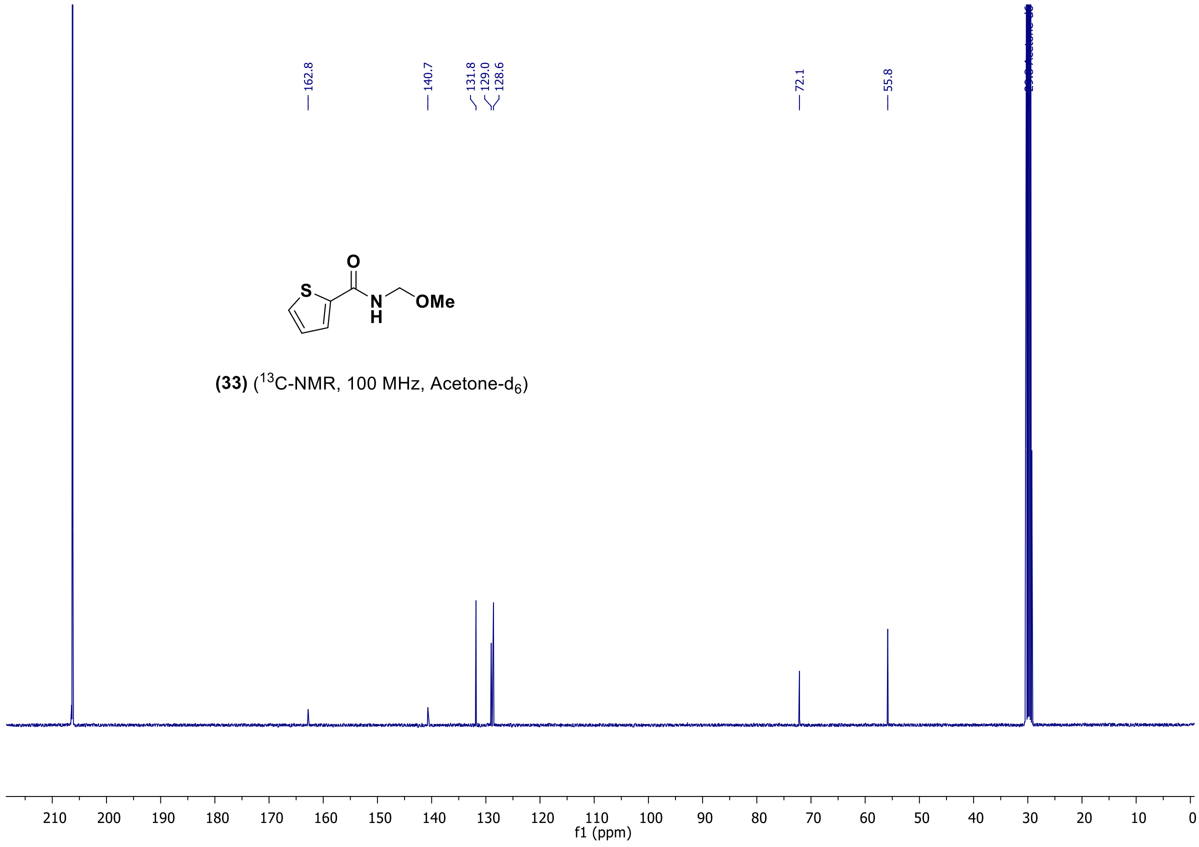

(33) (^{13}C-NMR, 100 MHz, Acetone-d_6)

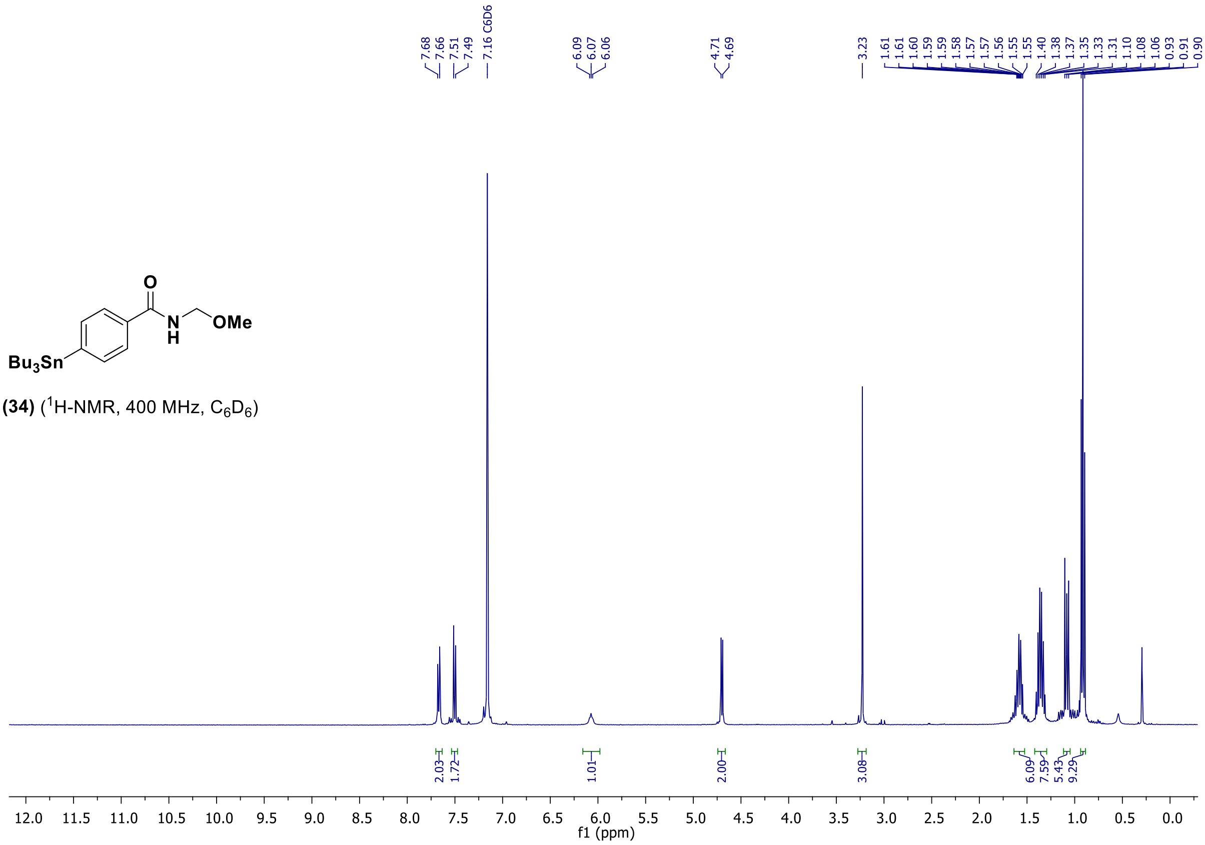

(34) (^{1}H-NMR, 400 MHz, C_6D_6)

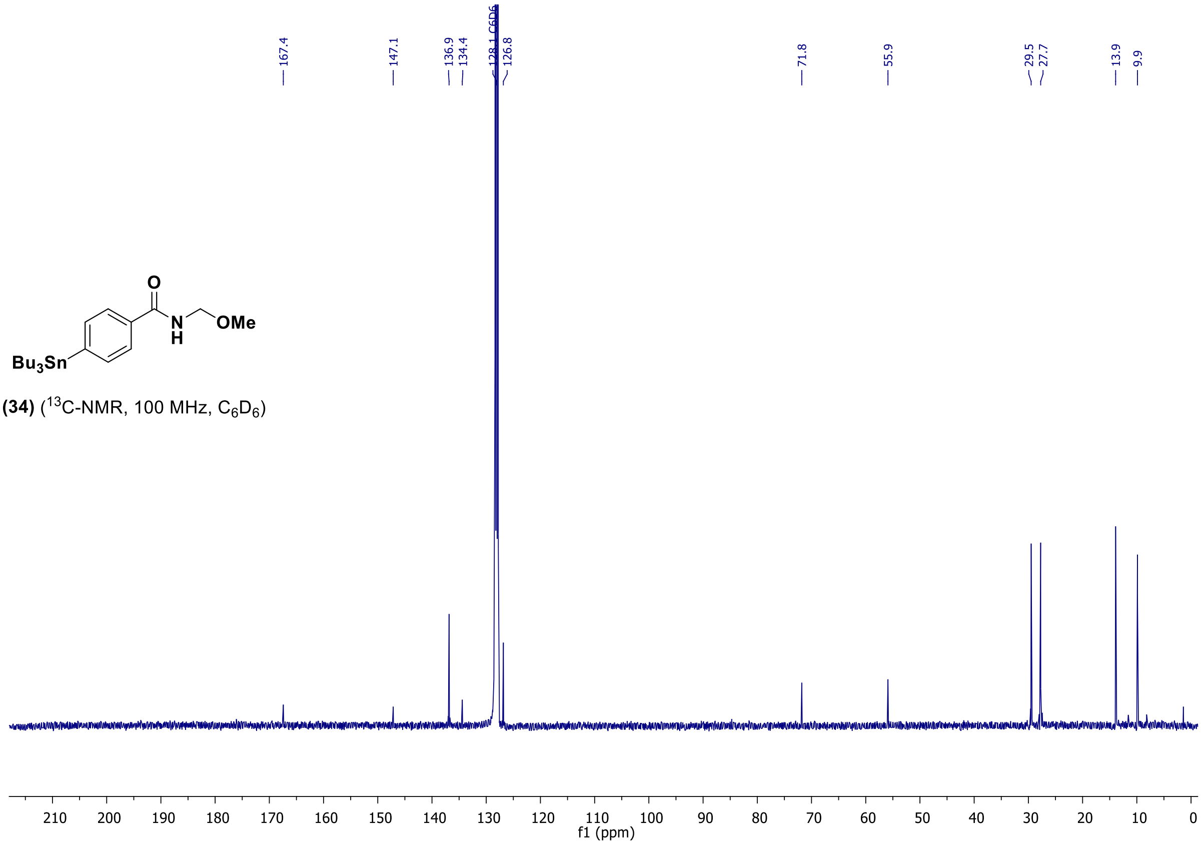

(34) (^{13}C-NMR, 100 MHz, C_6D_6)

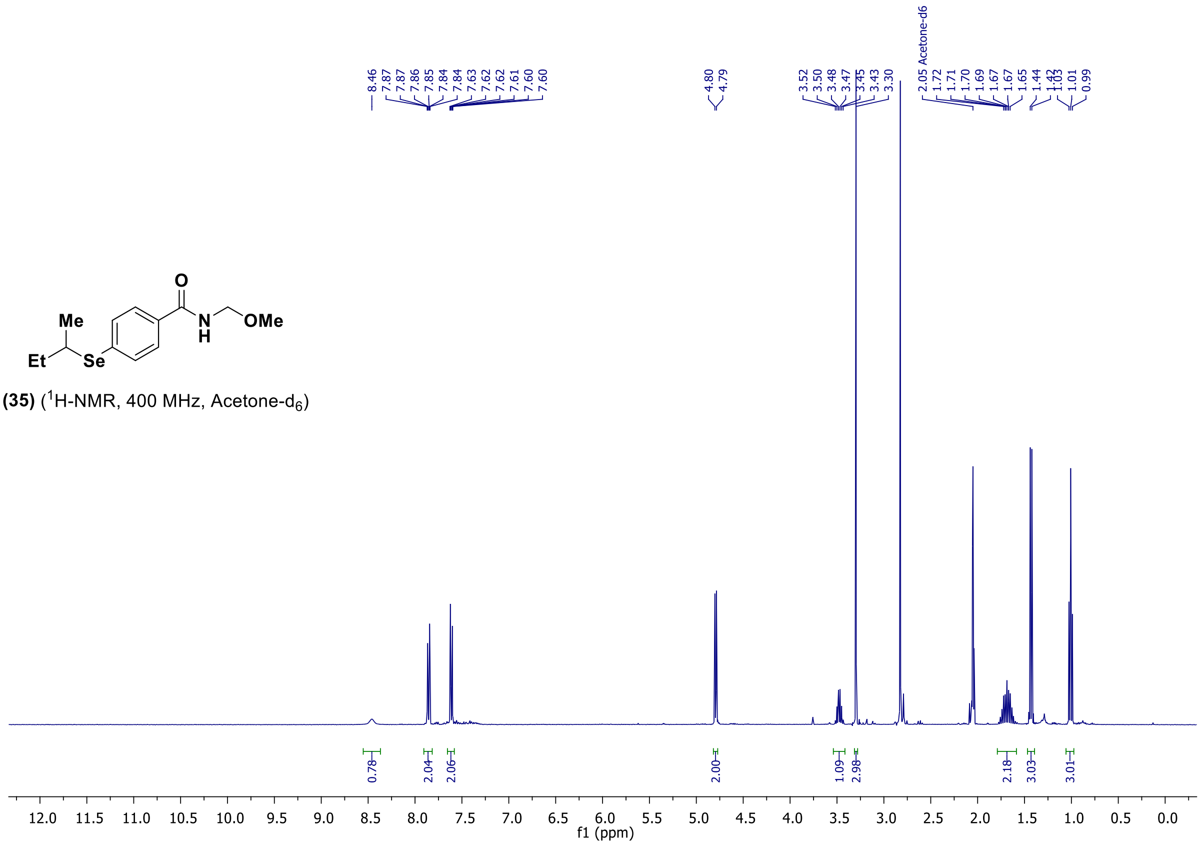

(35) (^{1}H-NMR, 400 MHz, Acetone-d_6)

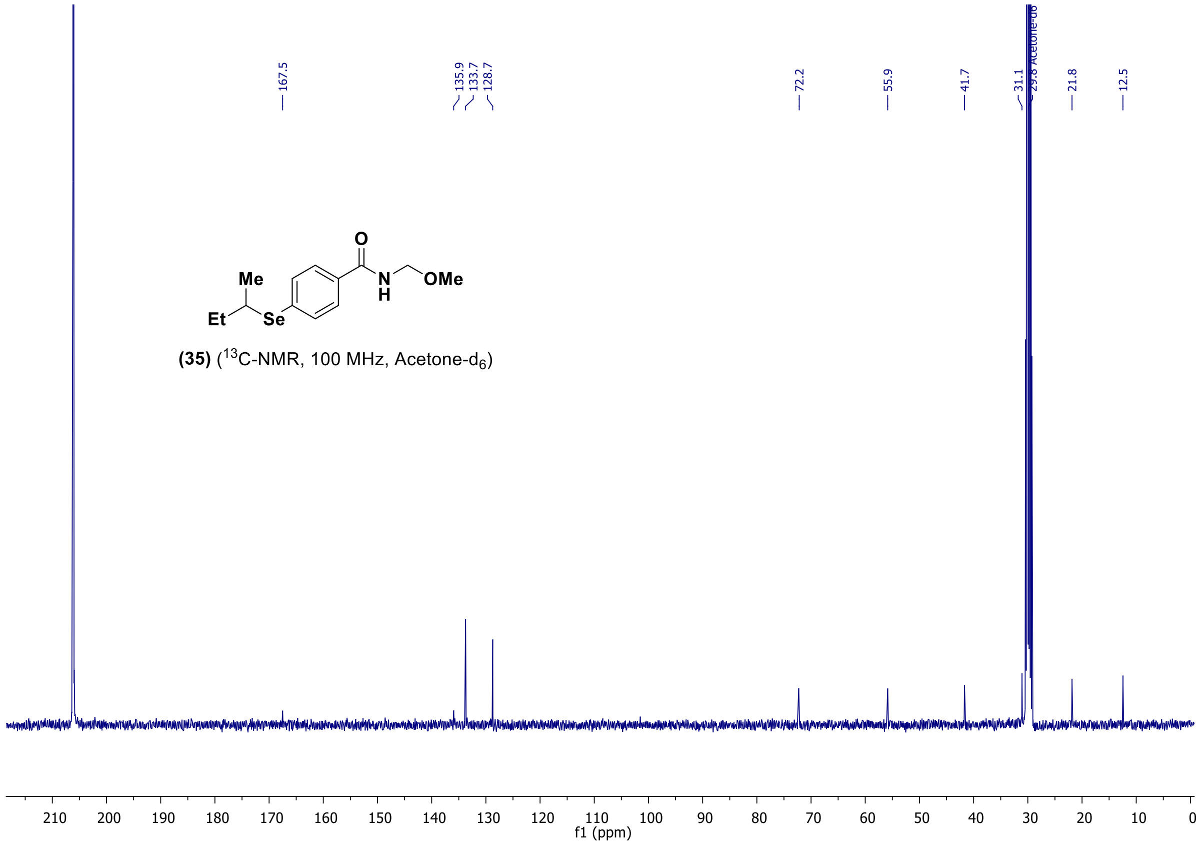

(35) (^{13}C-NMR, 100 MHz, Acetone-d_6)

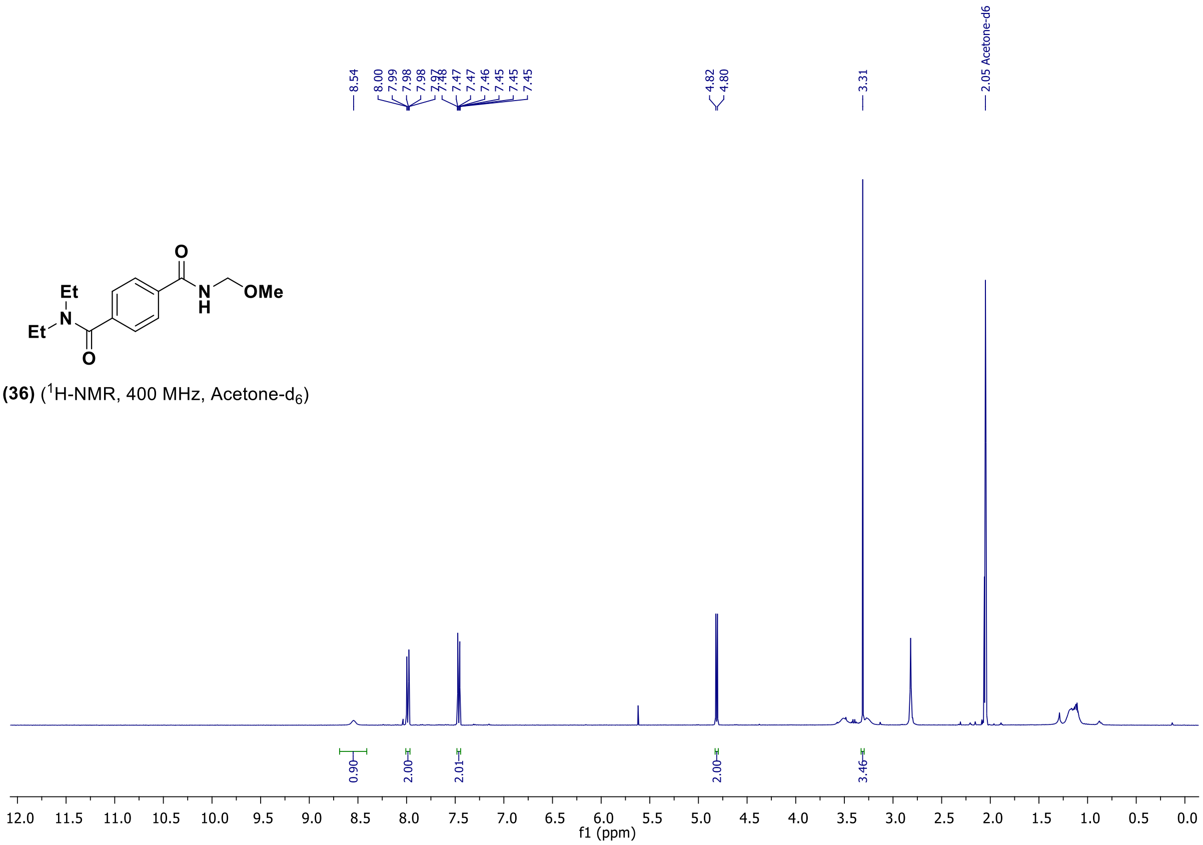

(36) (^{1}H-NMR, 400 MHz, Acetone-d_6)

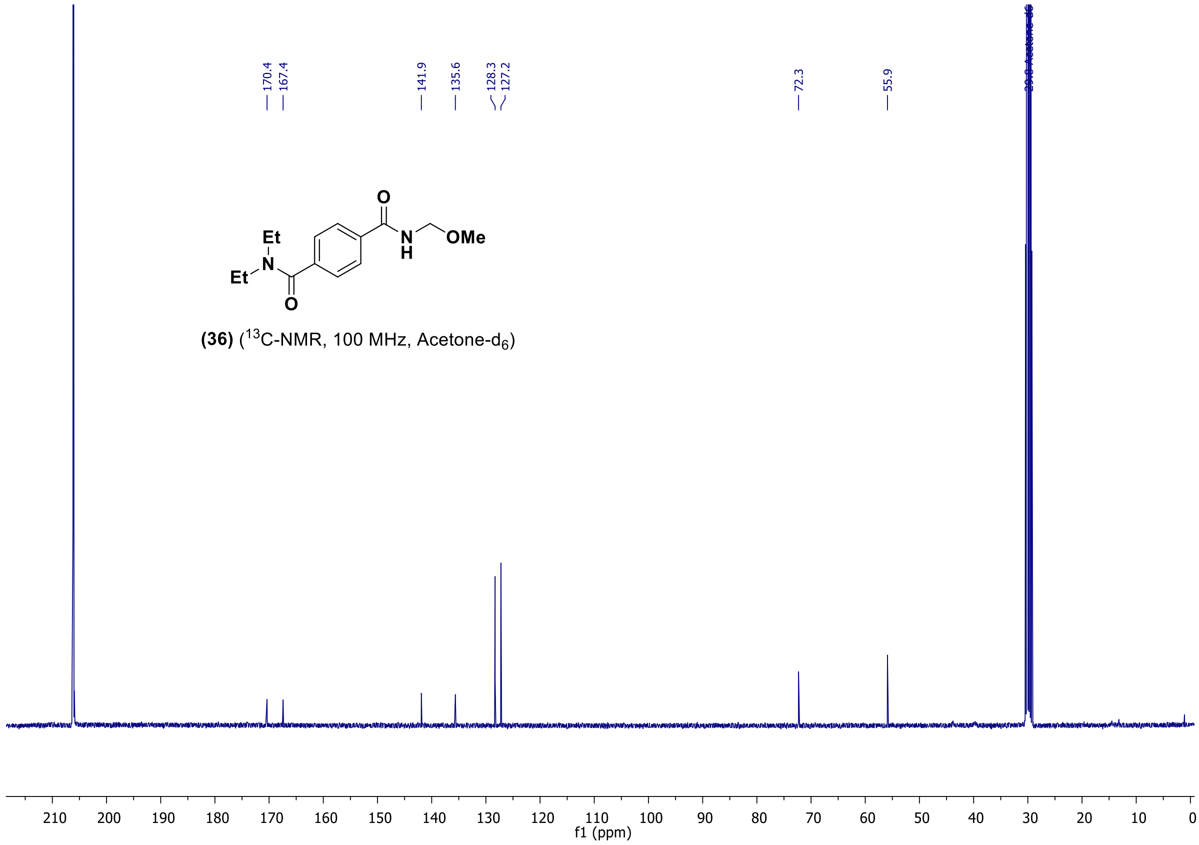

(36) (^{13}C-NMR, 100 MHz, Acetone-d_6)

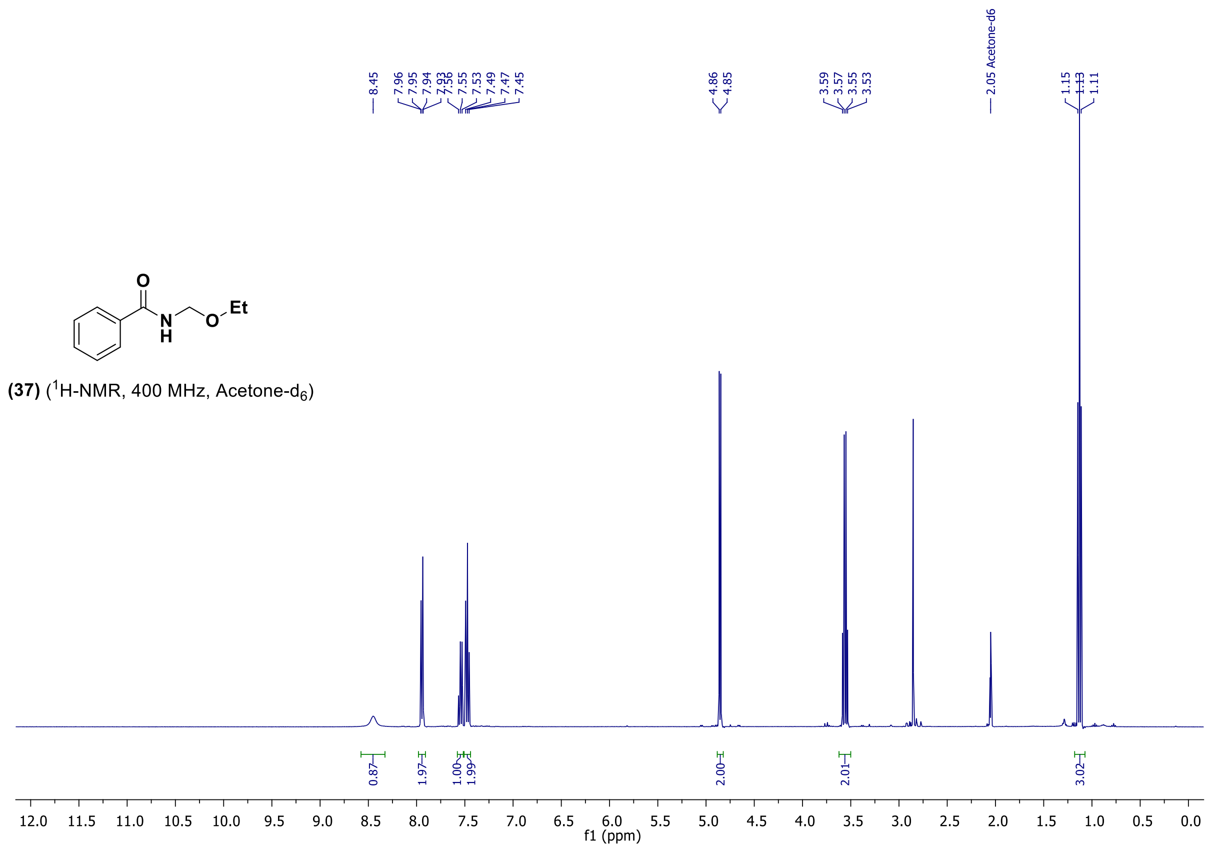

(37) (^{1}H-NMR, 400 MHz, Acetone-d_6)

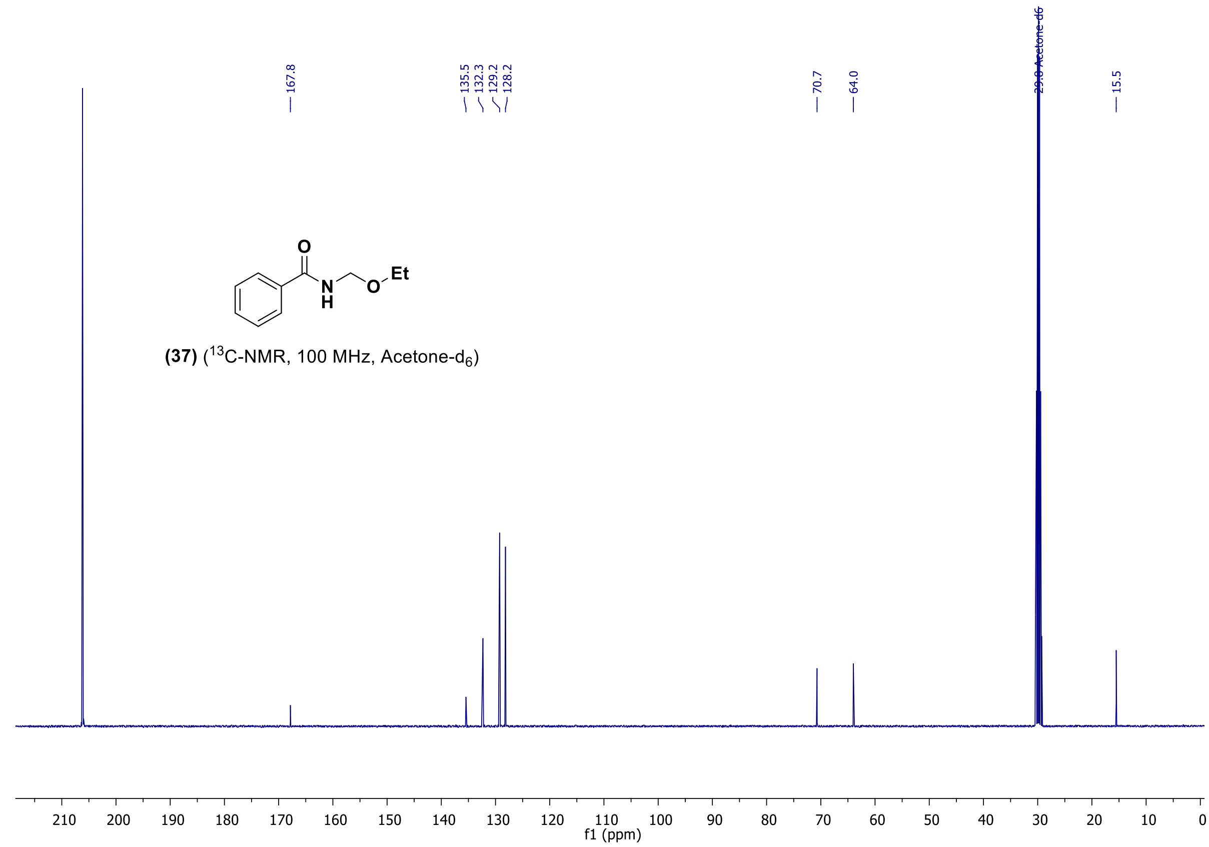

(37) (^{13}C-NMR, 100 MHz, Acetone-d_6)

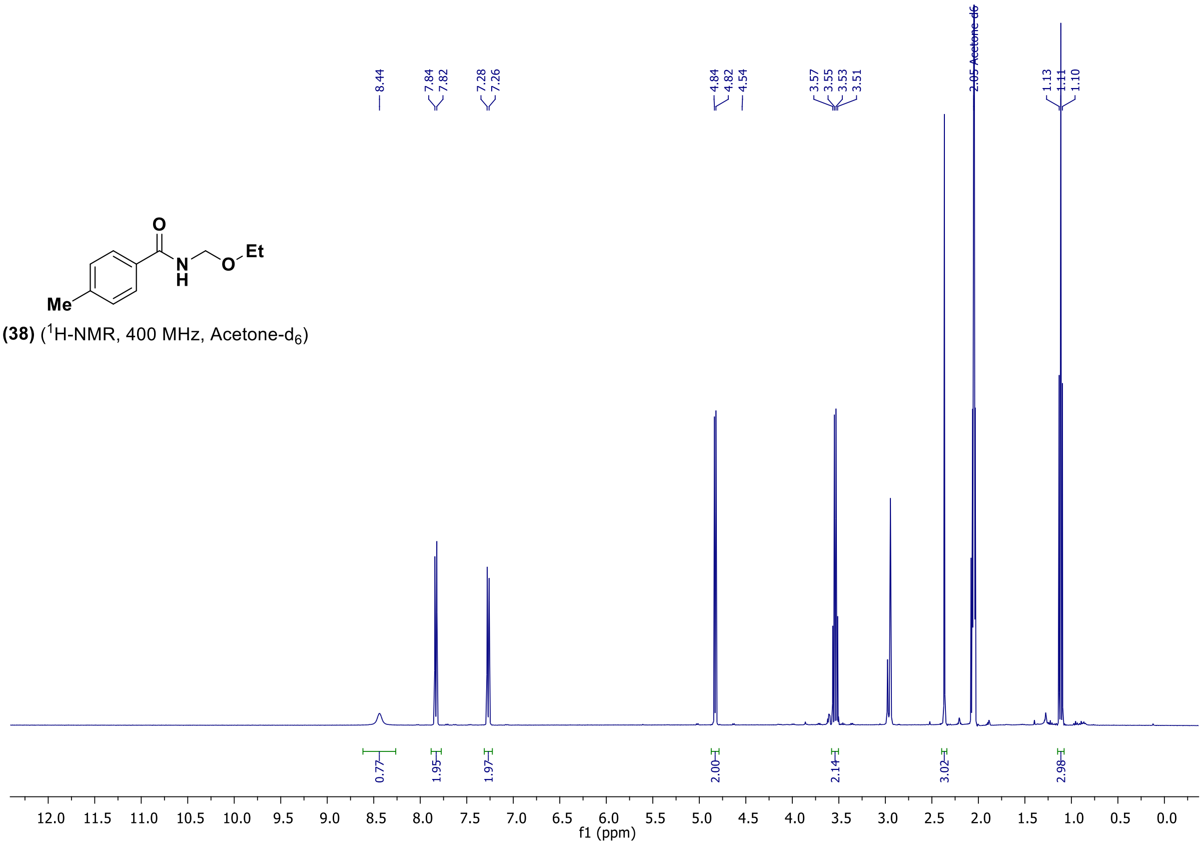

(38) (^{1}H-NMR, 400 MHz, Acetone-d_6)

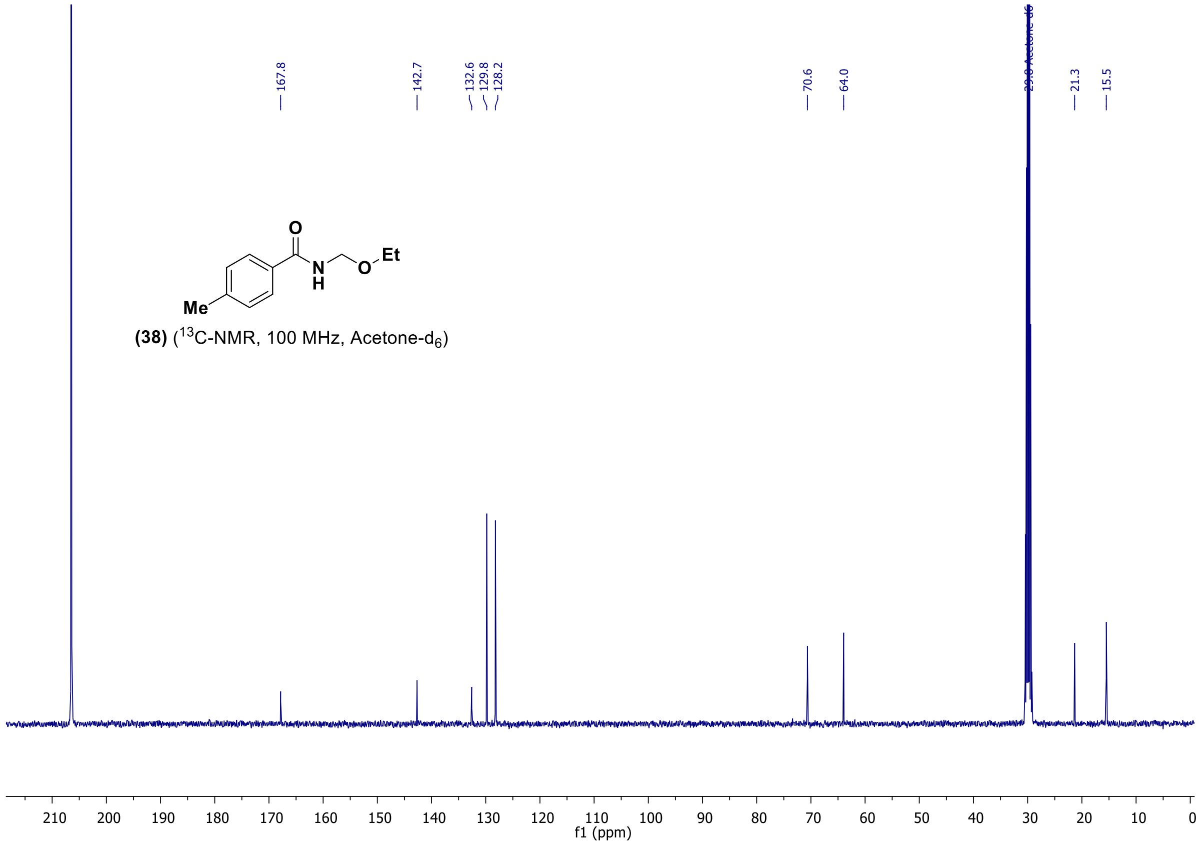

(38) (^{13}C-NMR, 100 MHz, Acetone-d_6)

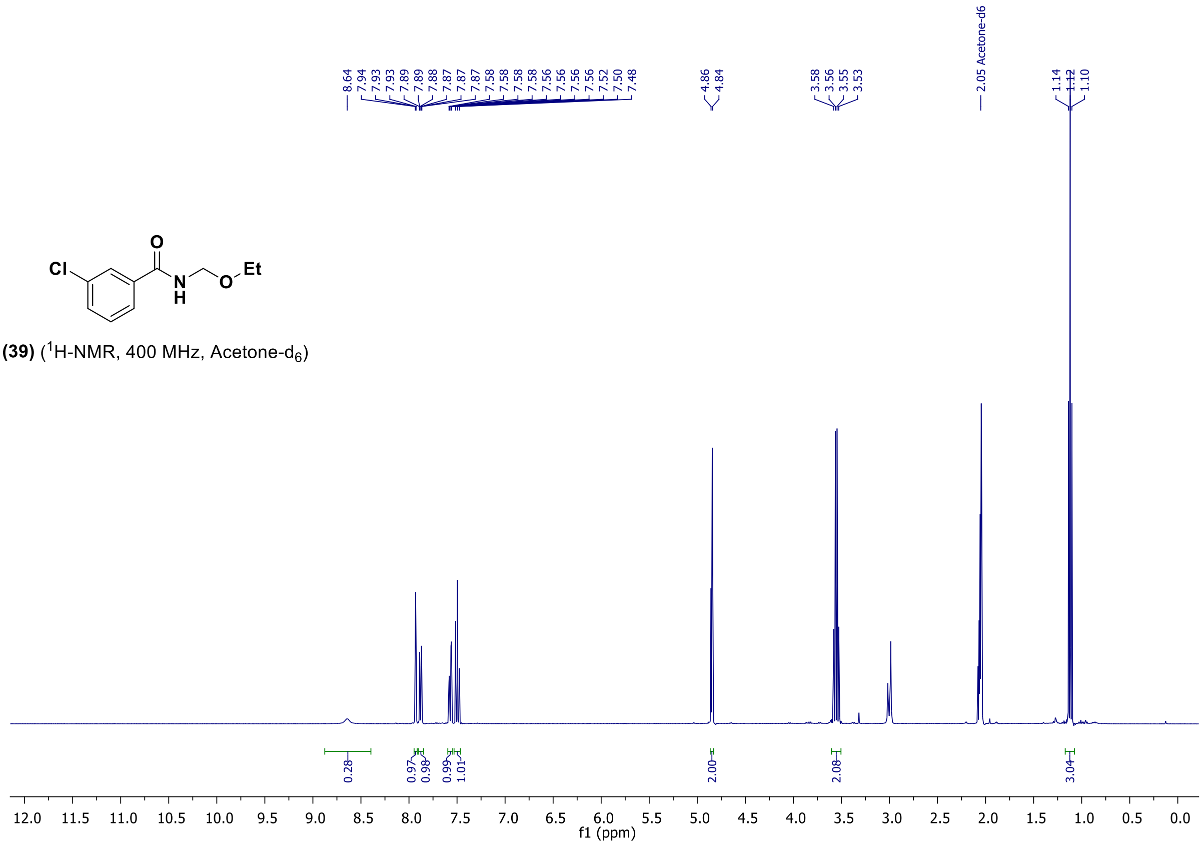

(39) (^{1}H-NMR, 400 MHz, Acetone-d_6)

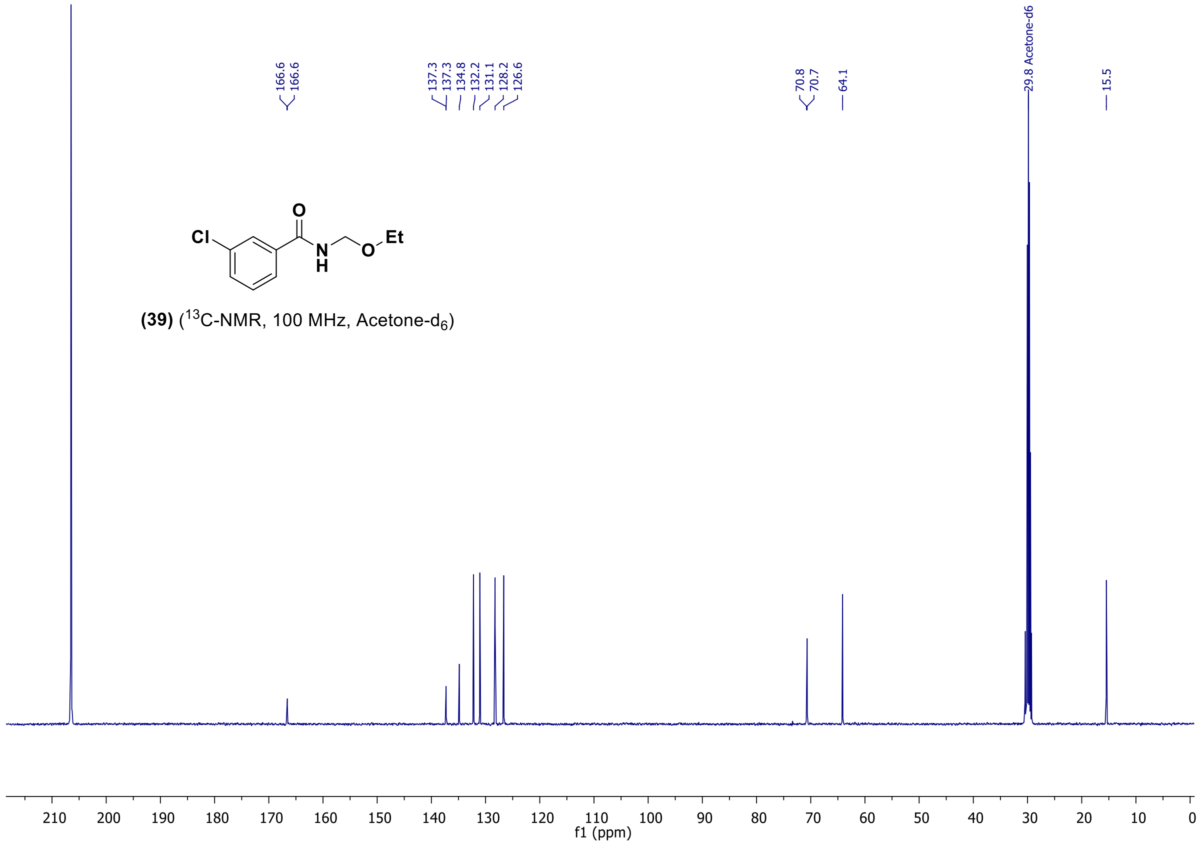

(39) (^{13}C-NMR, 100 MHz, Acetone-d_6)

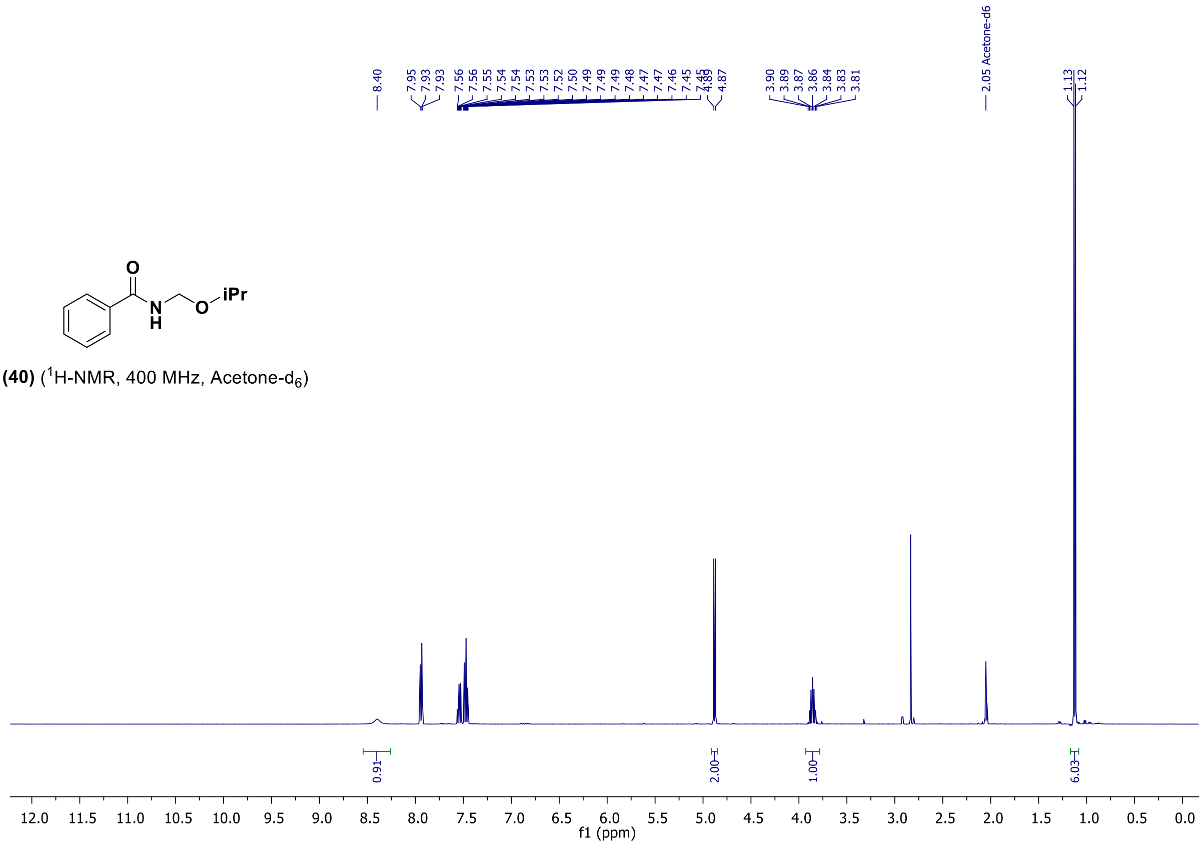

(40) (^{1}H-NMR, 400 MHz, Acetone-d_6)

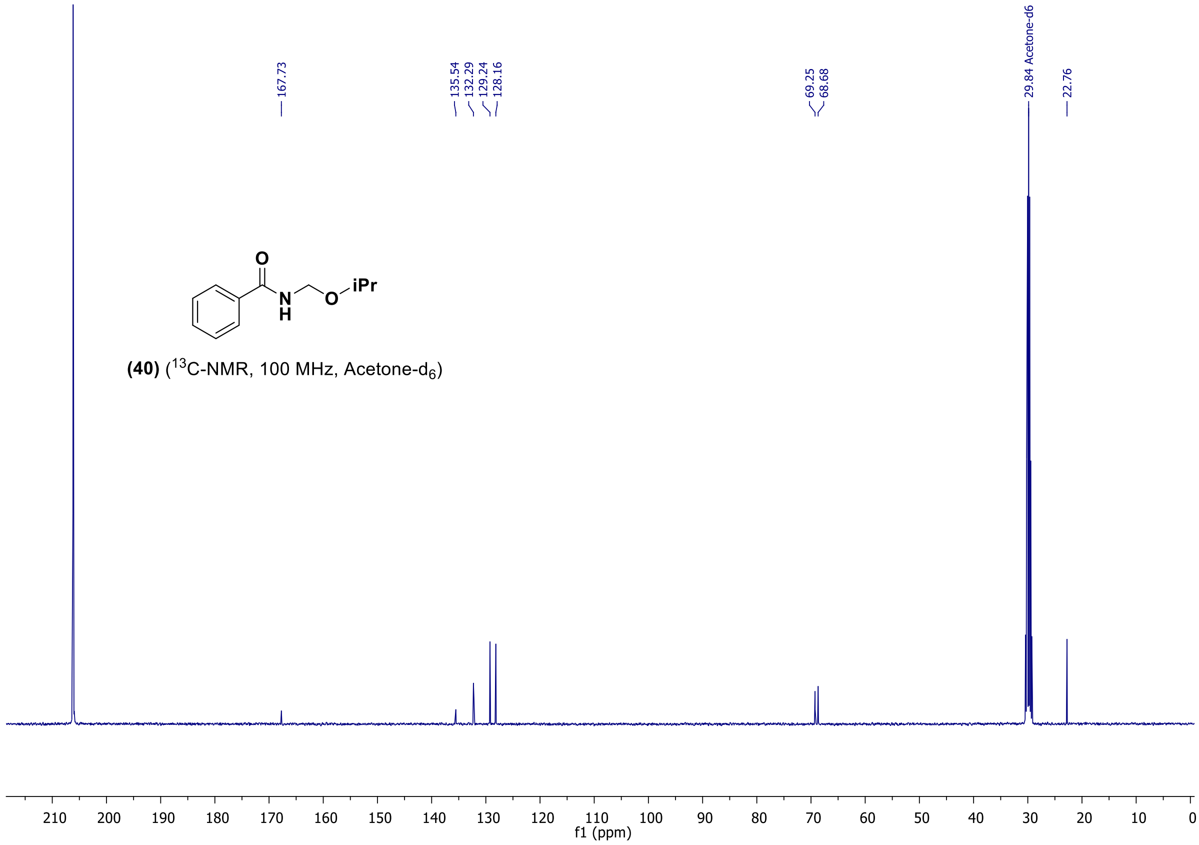

(40) (^{13}C-NMR, 100 MHz, Acetone-d_6)

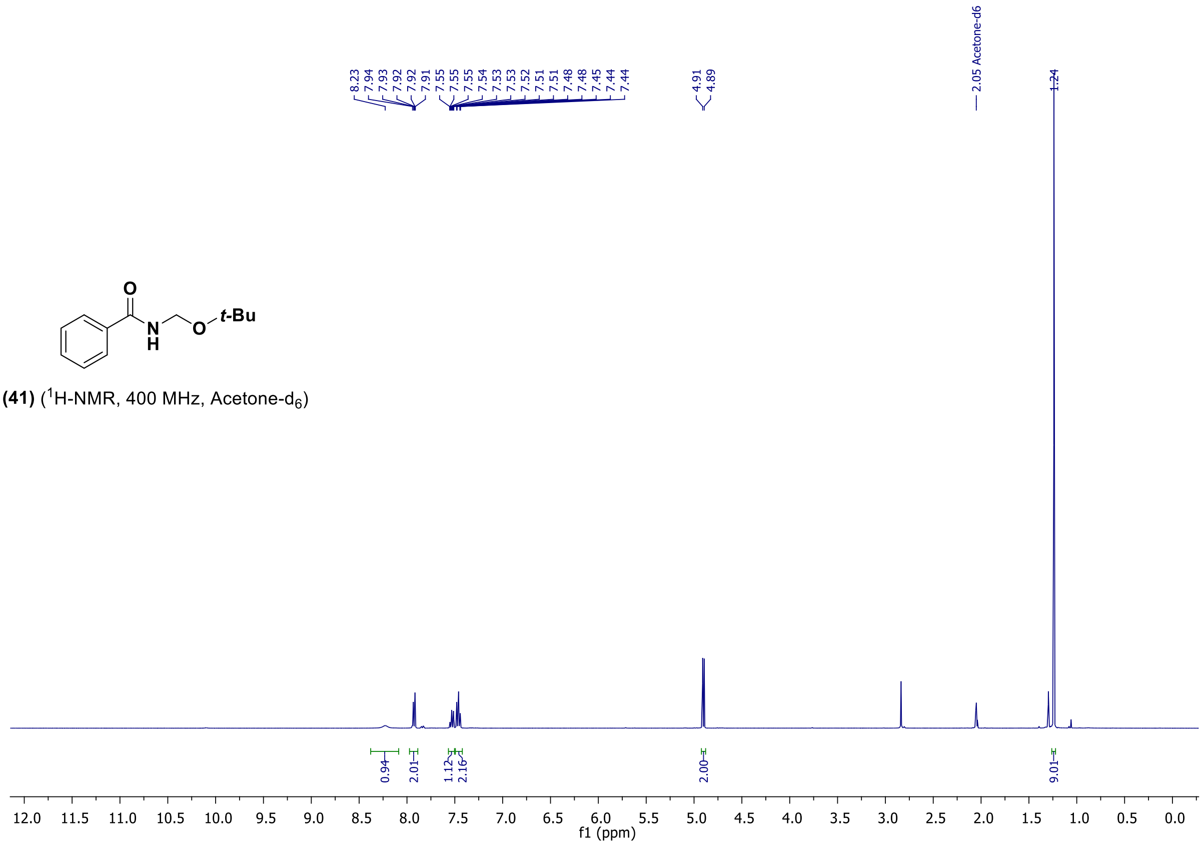

(41) (^{1}H-NMR, 400 MHz, Acetone-d$_6$)

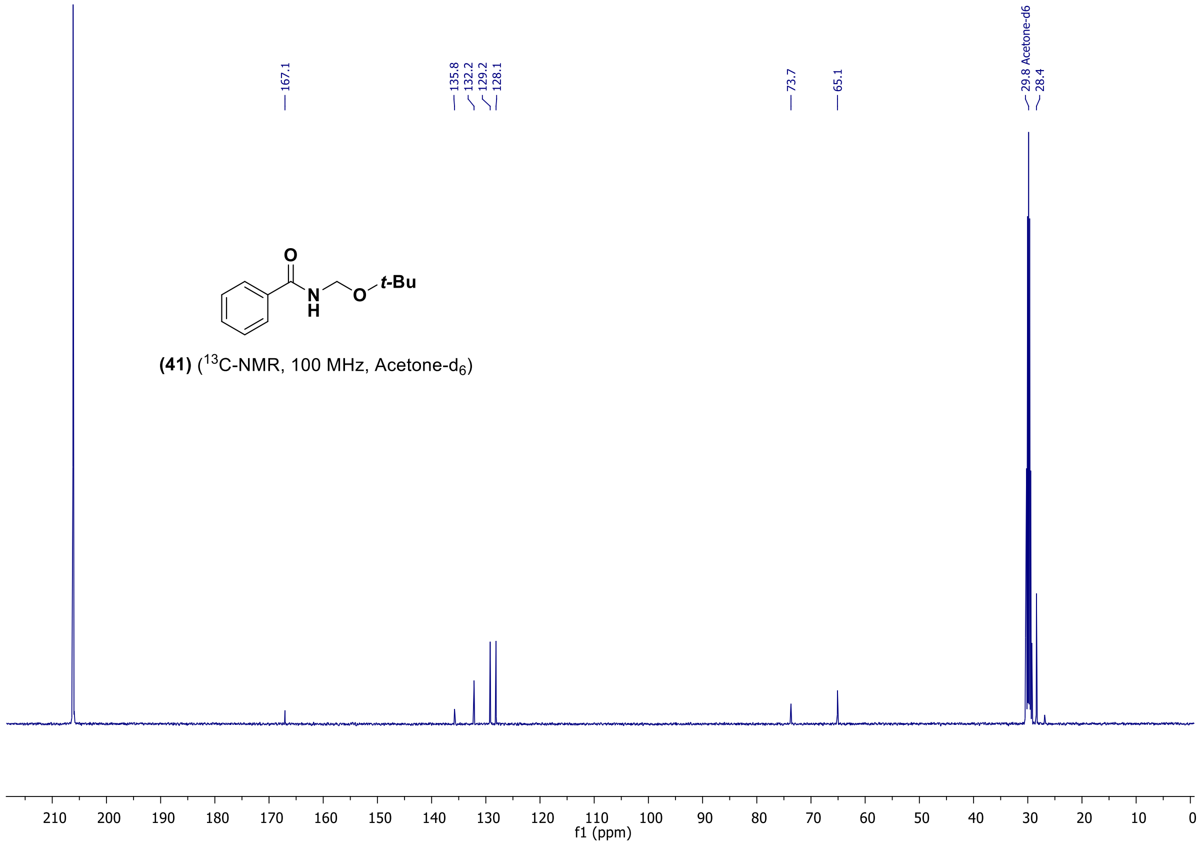

(41) (^{13}C-NMR, 100 MHz, Acetone-d$_6$)

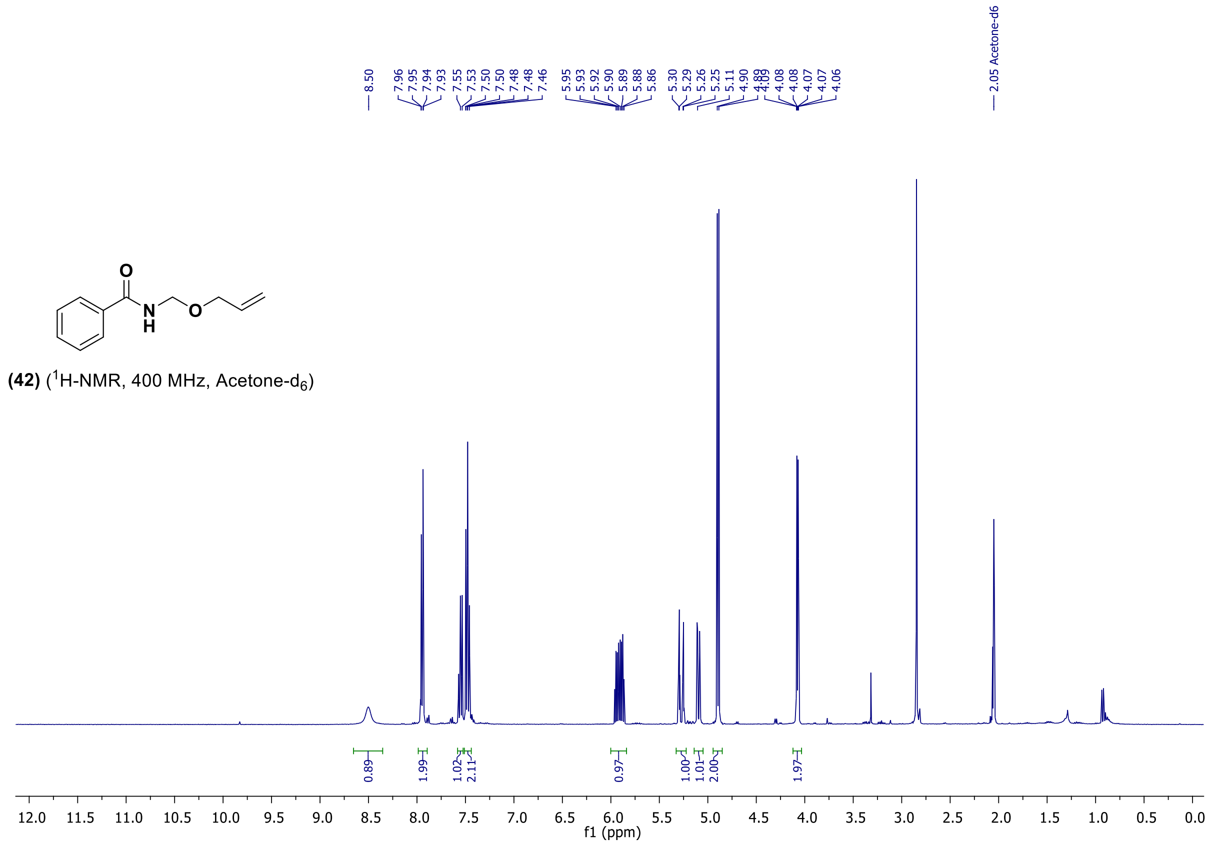

(42) (^{1}H-NMR, 400 MHz, Acetone-d_6)

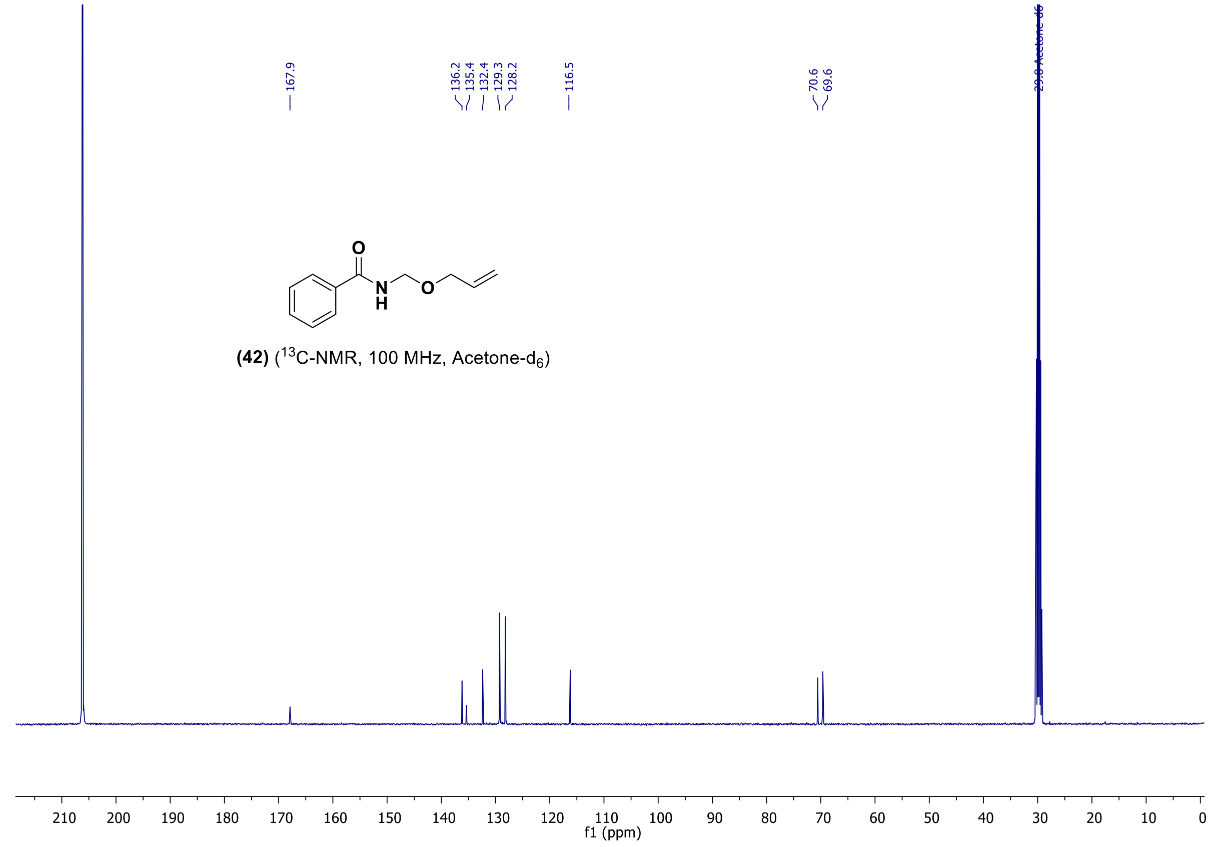

(42) (^{13}C-NMR, 100 MHz, Acetone-d_6)

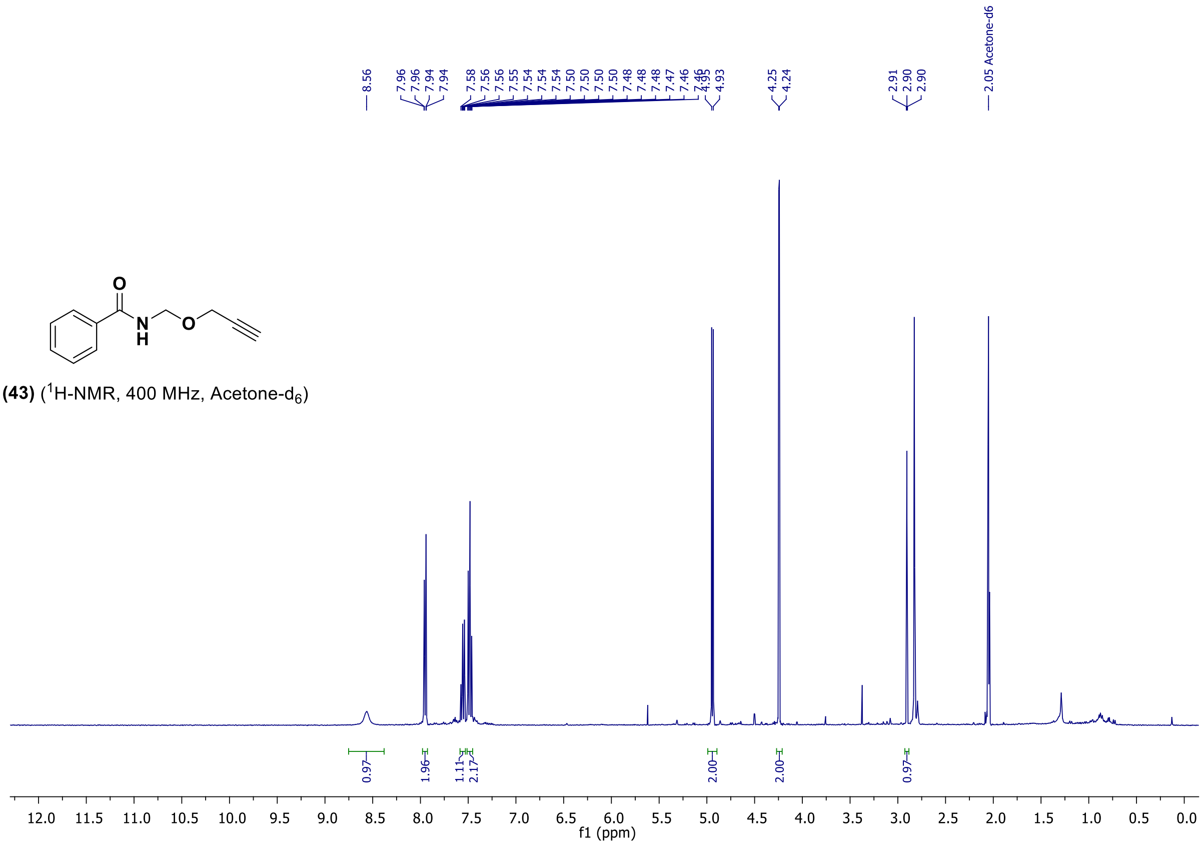

(43) (^{1}H-NMR, 400 MHz, Acetone-d$_6$)

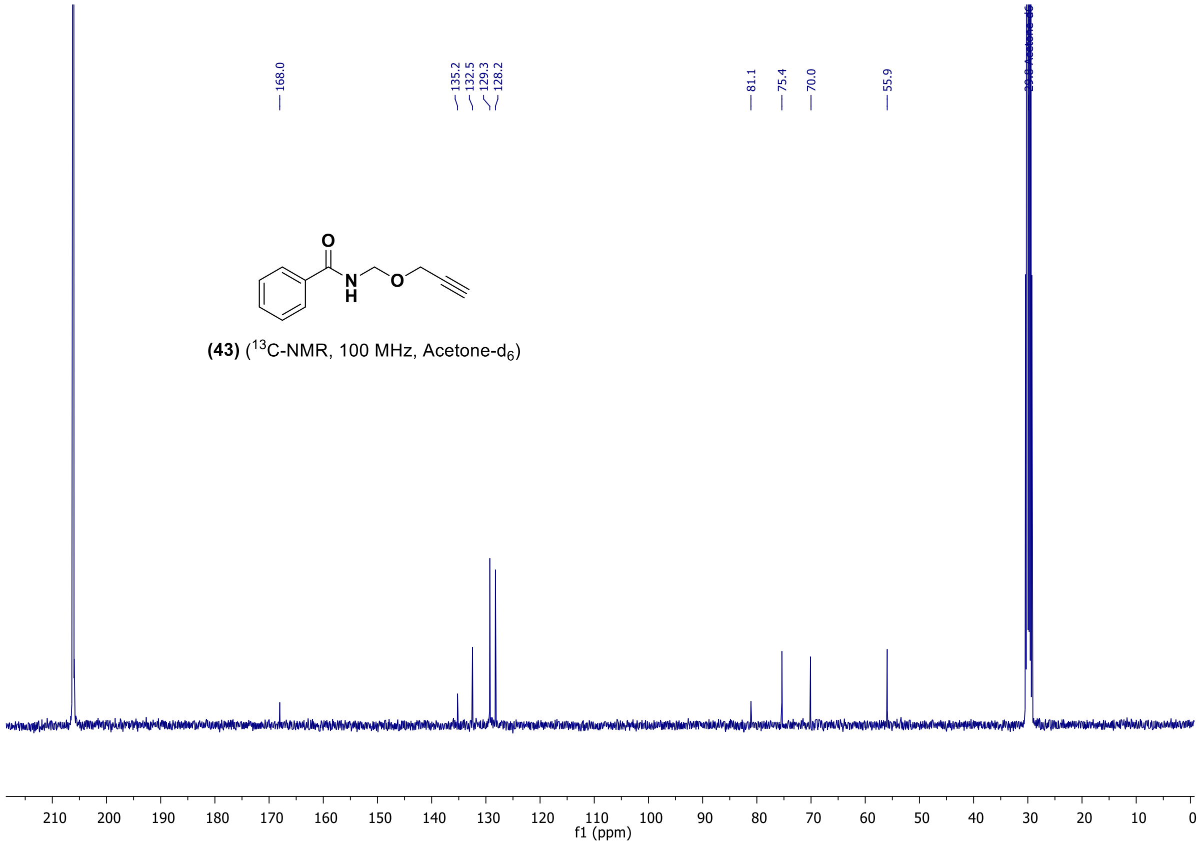

(43) (^{13}C-NMR, 100 MHz, Acetone-d$_6$)

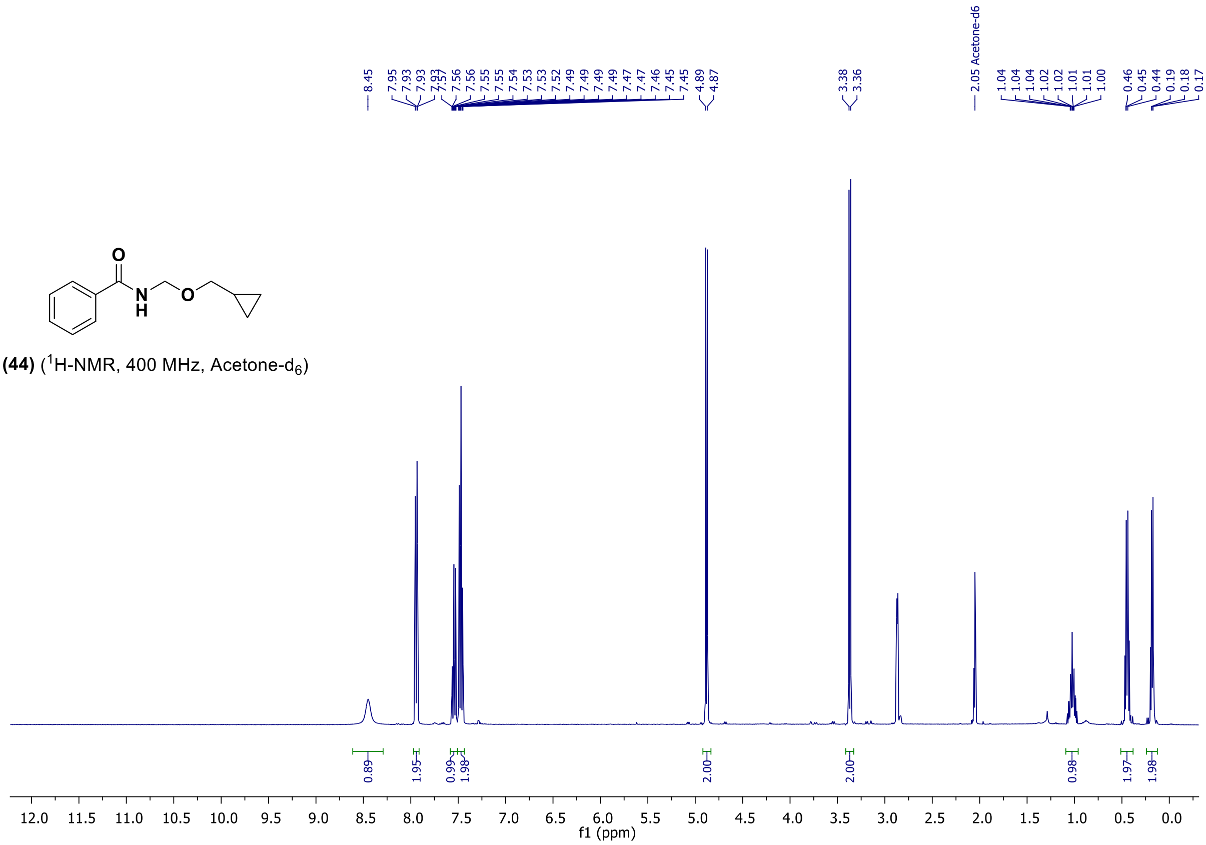

(44) (^{1}H-NMR, 400 MHz, Acetone-d_6)

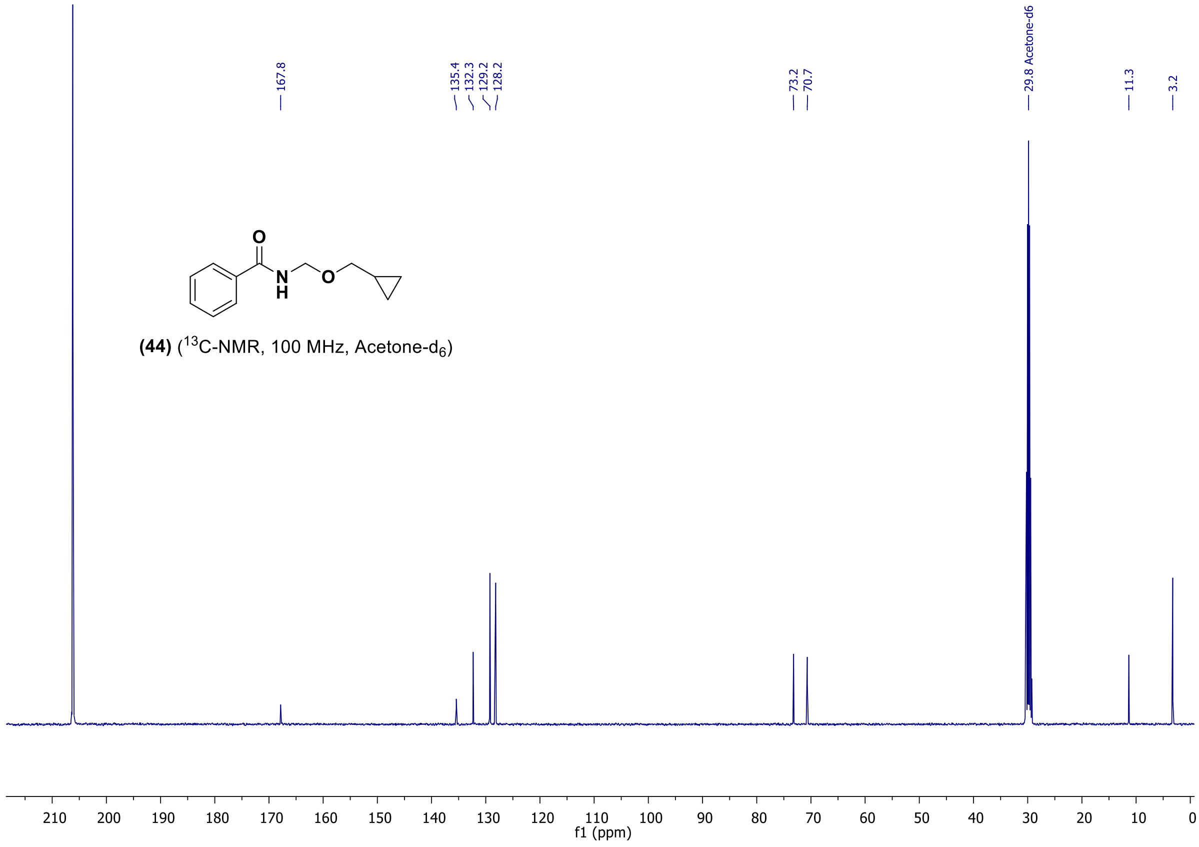

(44) (^{13}C-NMR, 100 MHz, Acetone-d_6)

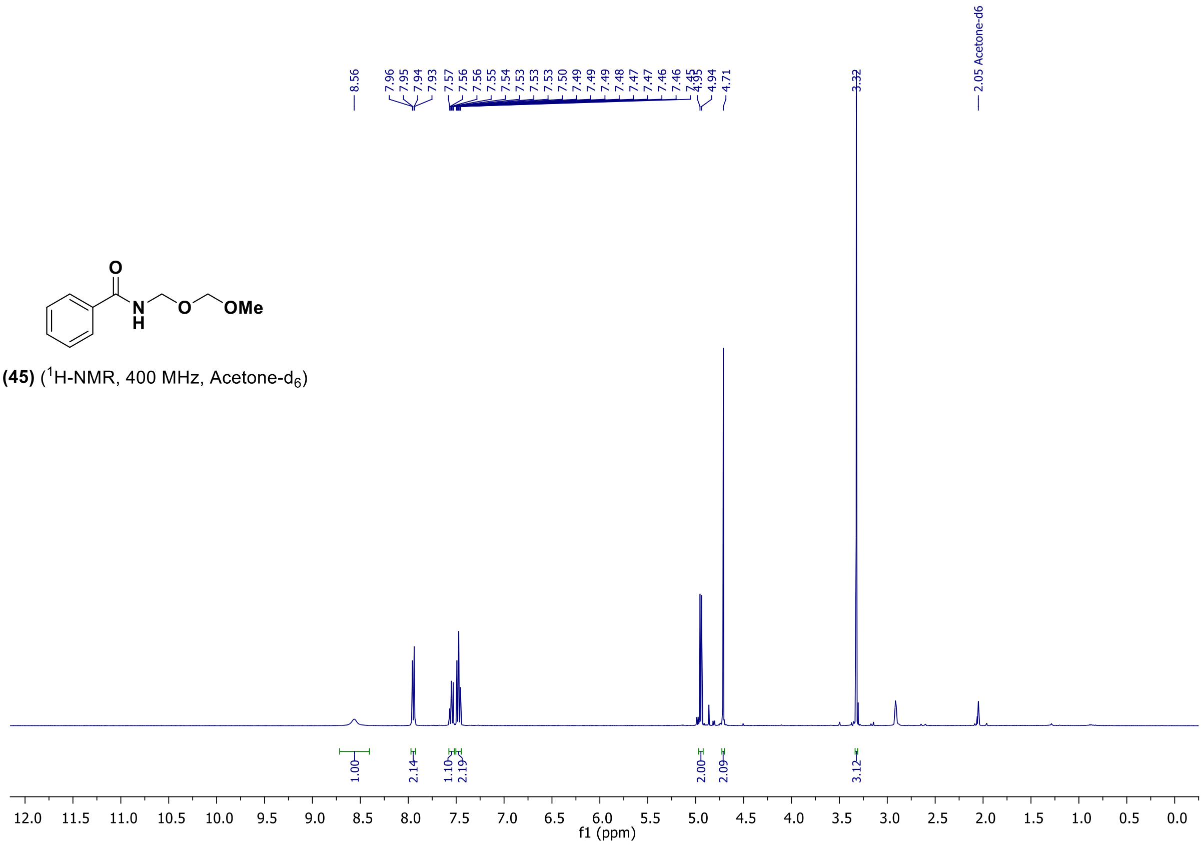

(45) (^{1}H-NMR, 400 MHz, Acetone-d_6)

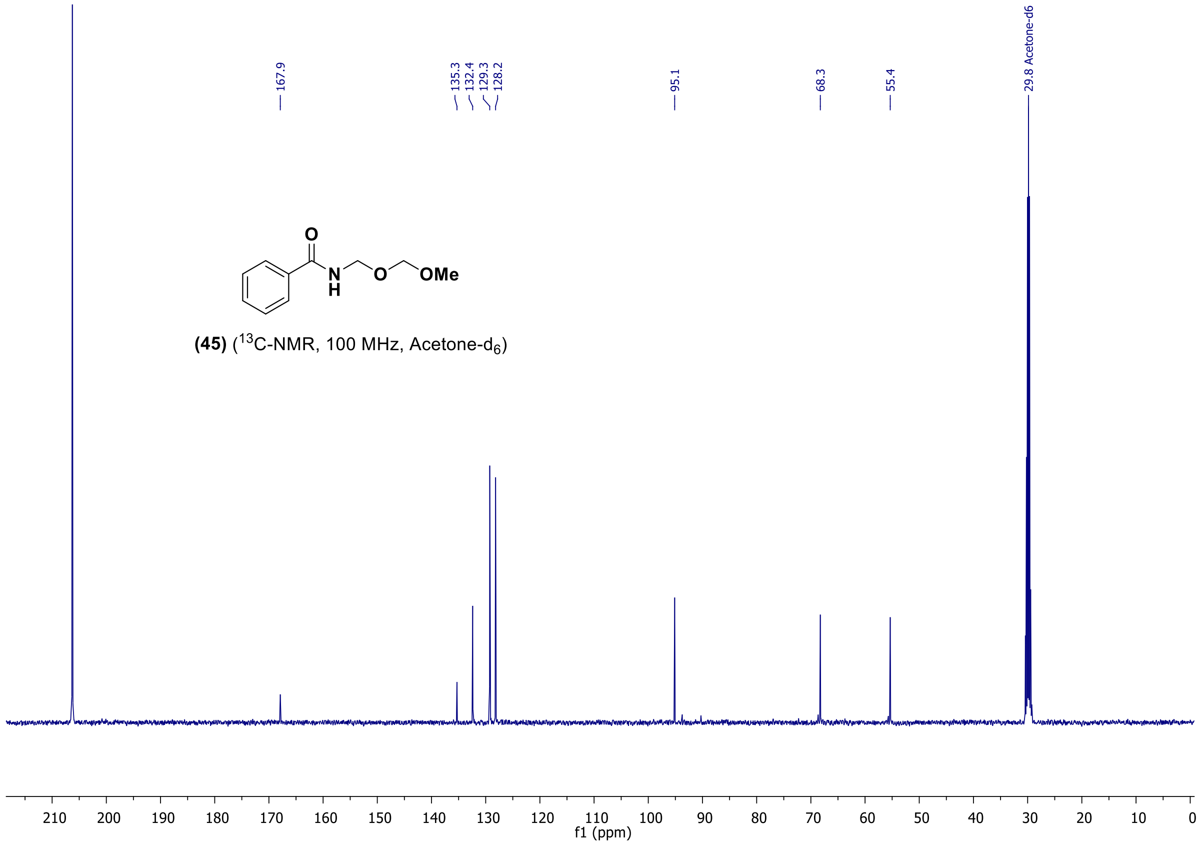

(45) (^{13}C-NMR, 100 MHz, Acetone-d_6)

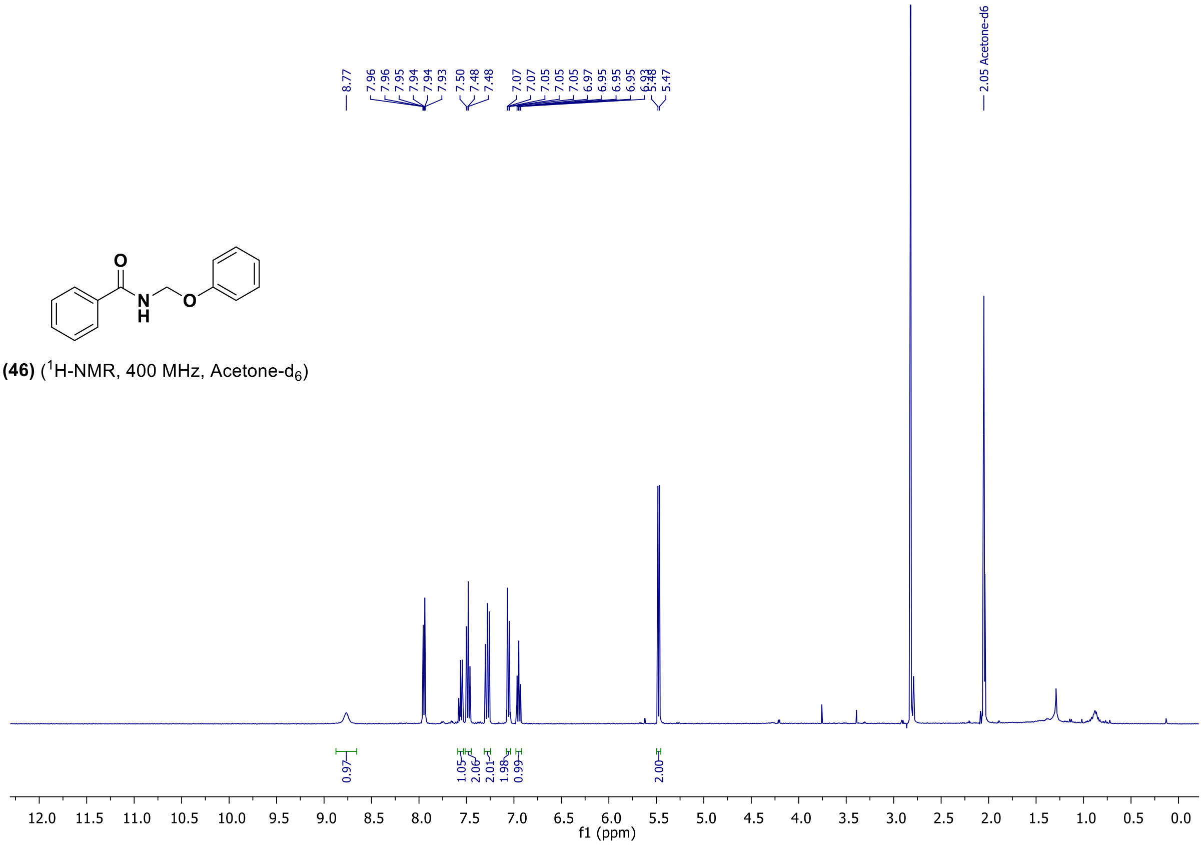

(46) (^{1}H-NMR, 400 MHz, Acetone-d_6)

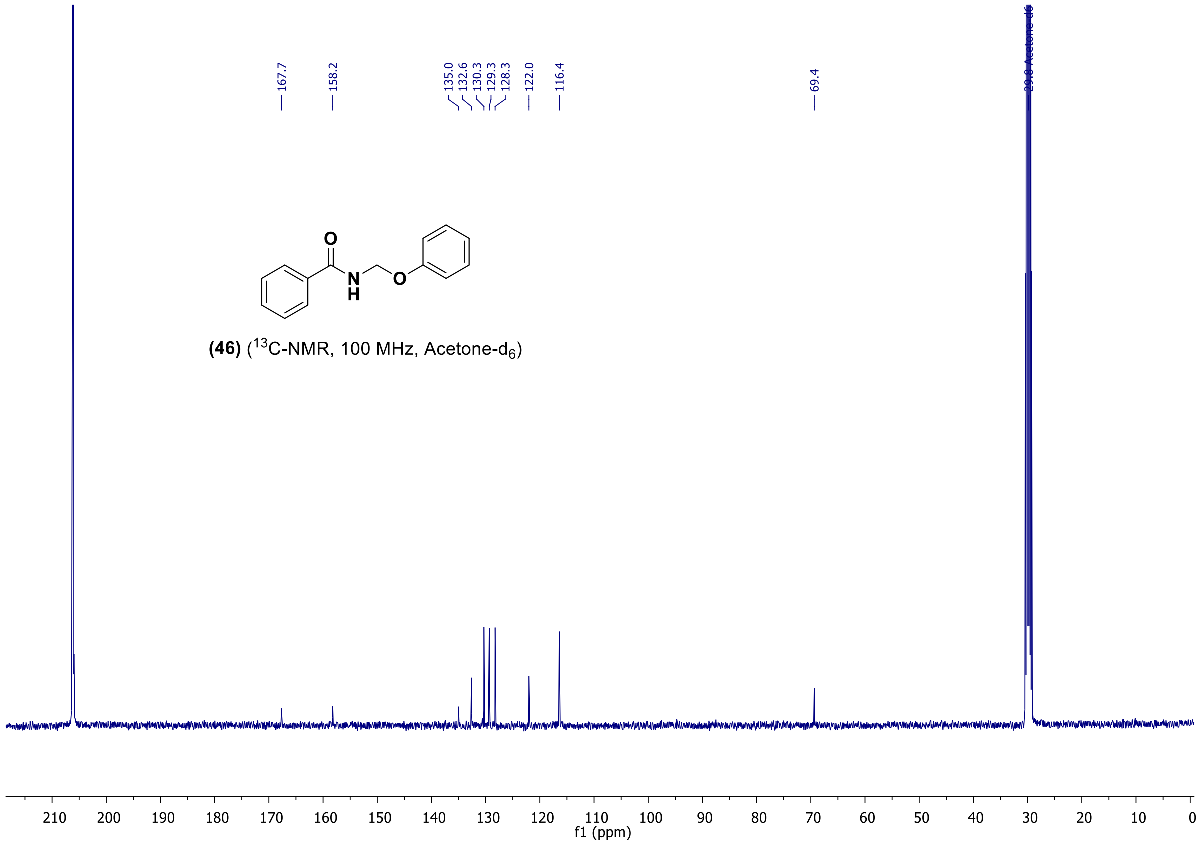

(46) (^{13}C-NMR, 100 MHz, Acetone-d_6)

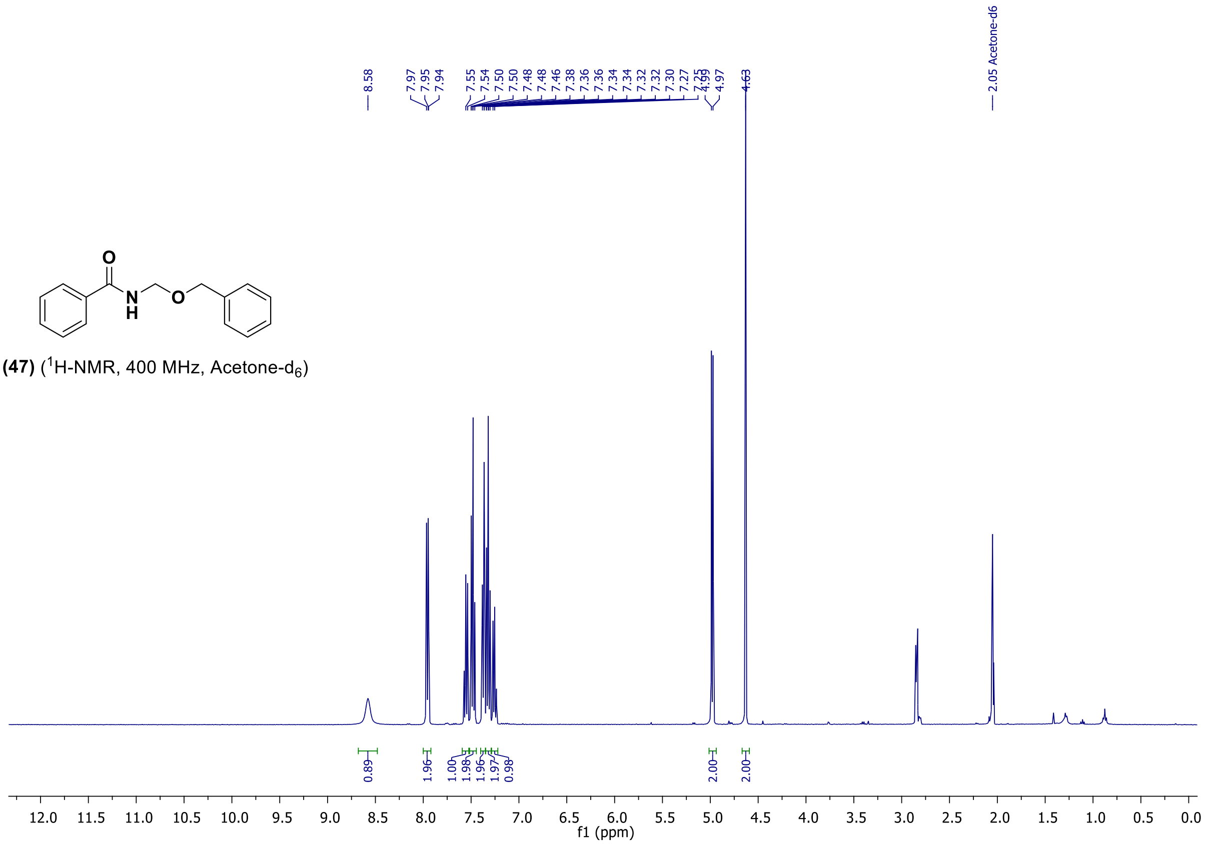

(47) (^{1}H-NMR, 400 MHz, Acetone-d$_6$)

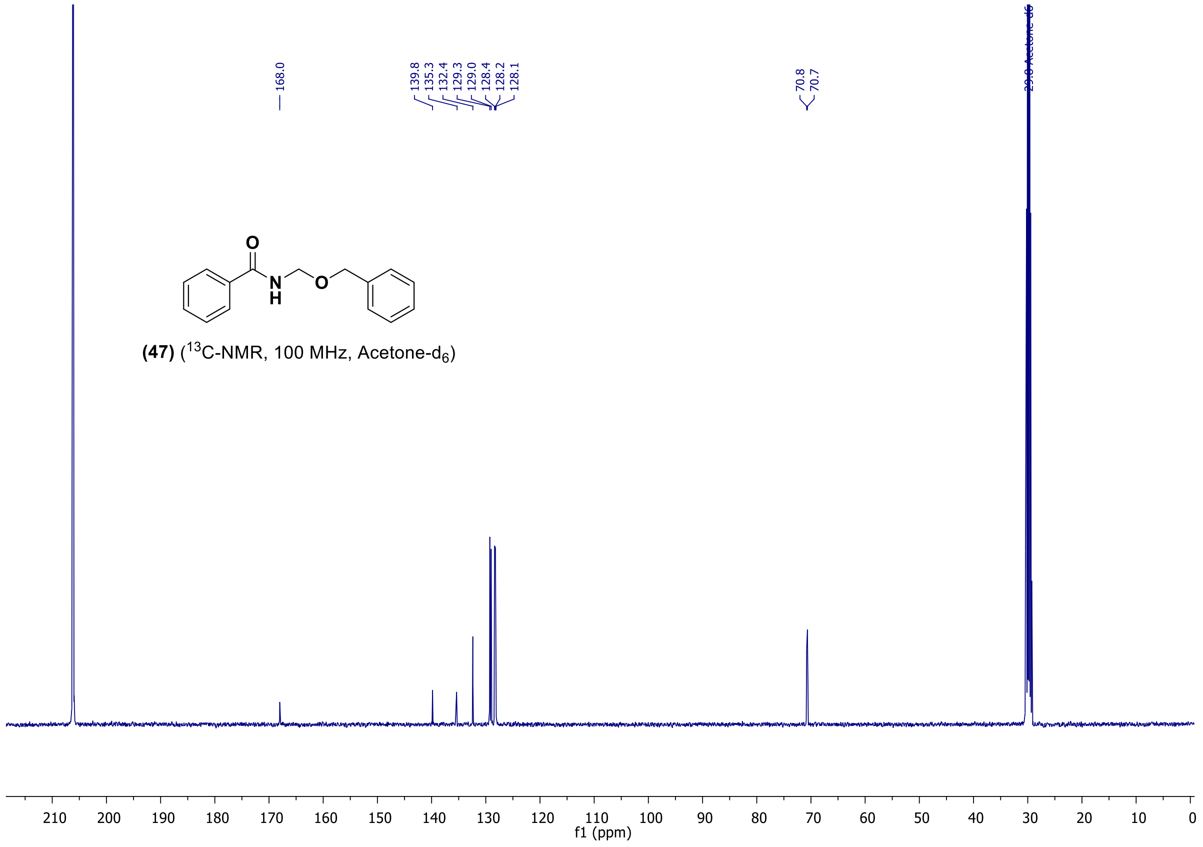

(47) (^{13}C-NMR, 100 MHz, Acetone-d$_6$)

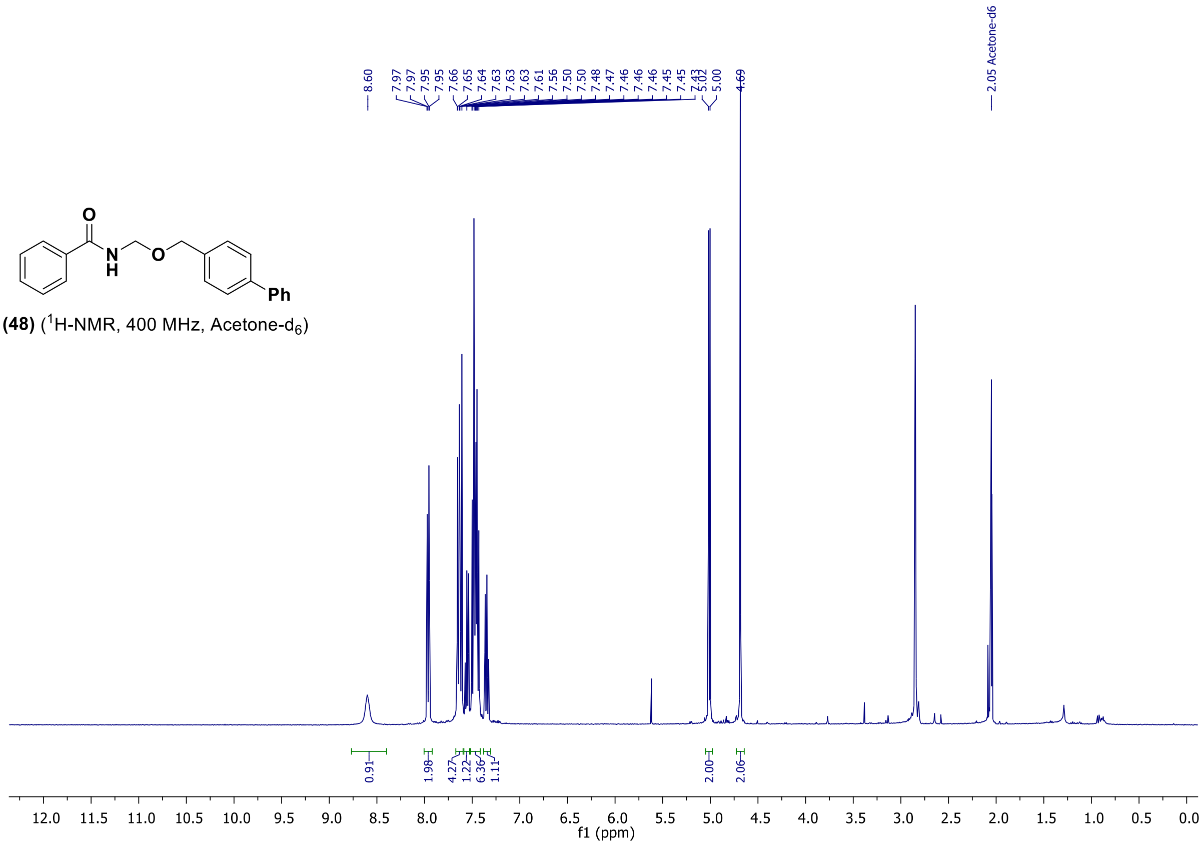

(48) (^{1}H-NMR, 400 MHz, Acetone-d_6)

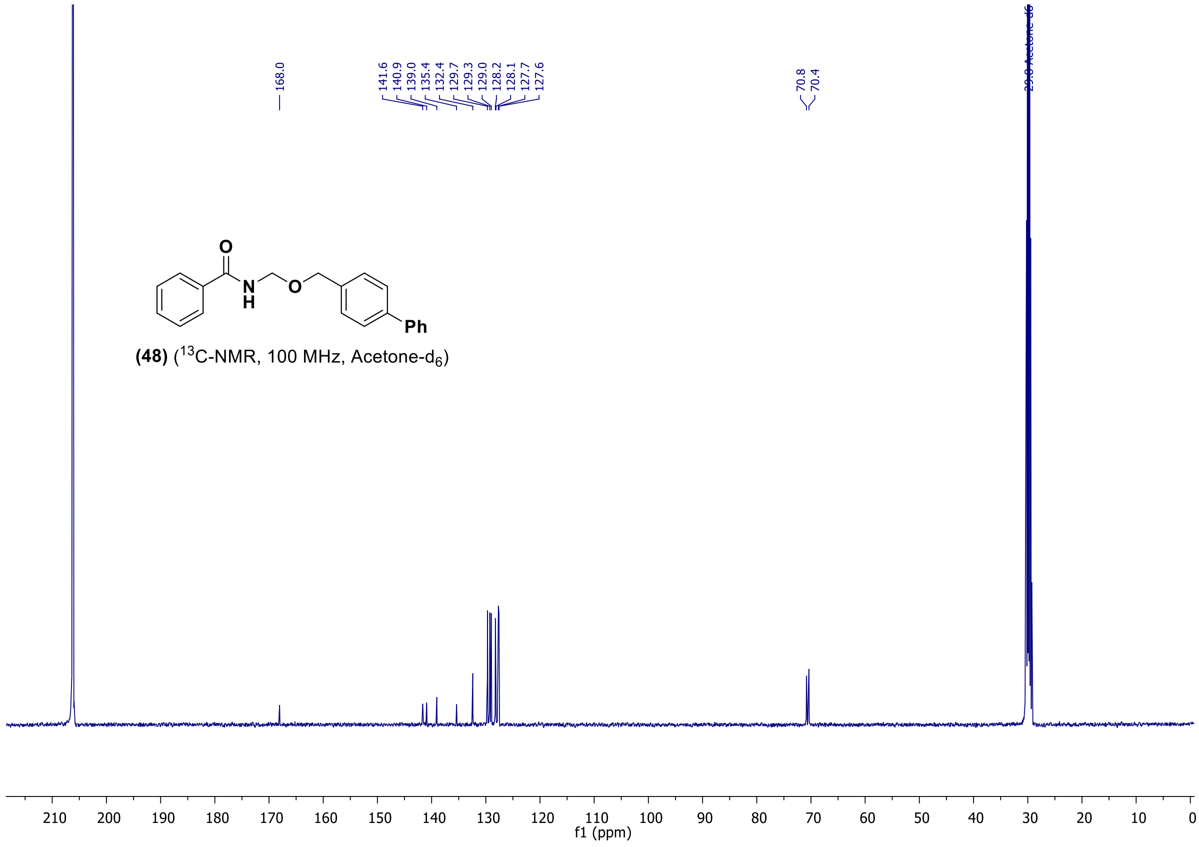

(48) (^{13}C-NMR, 100 MHz, Acetone-d_6)

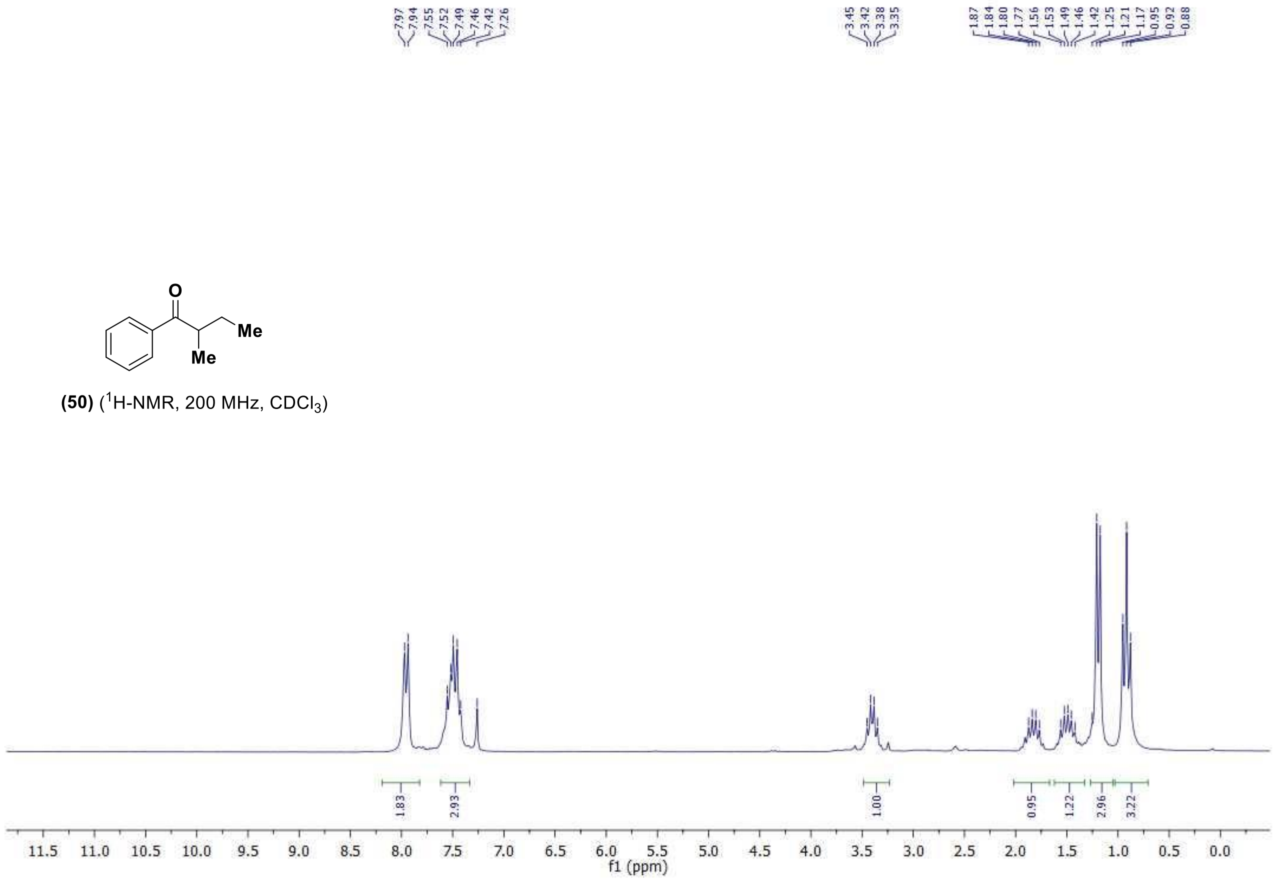

(50) (^{1}H-NMR, 200 MHz, $CDCl_3$)

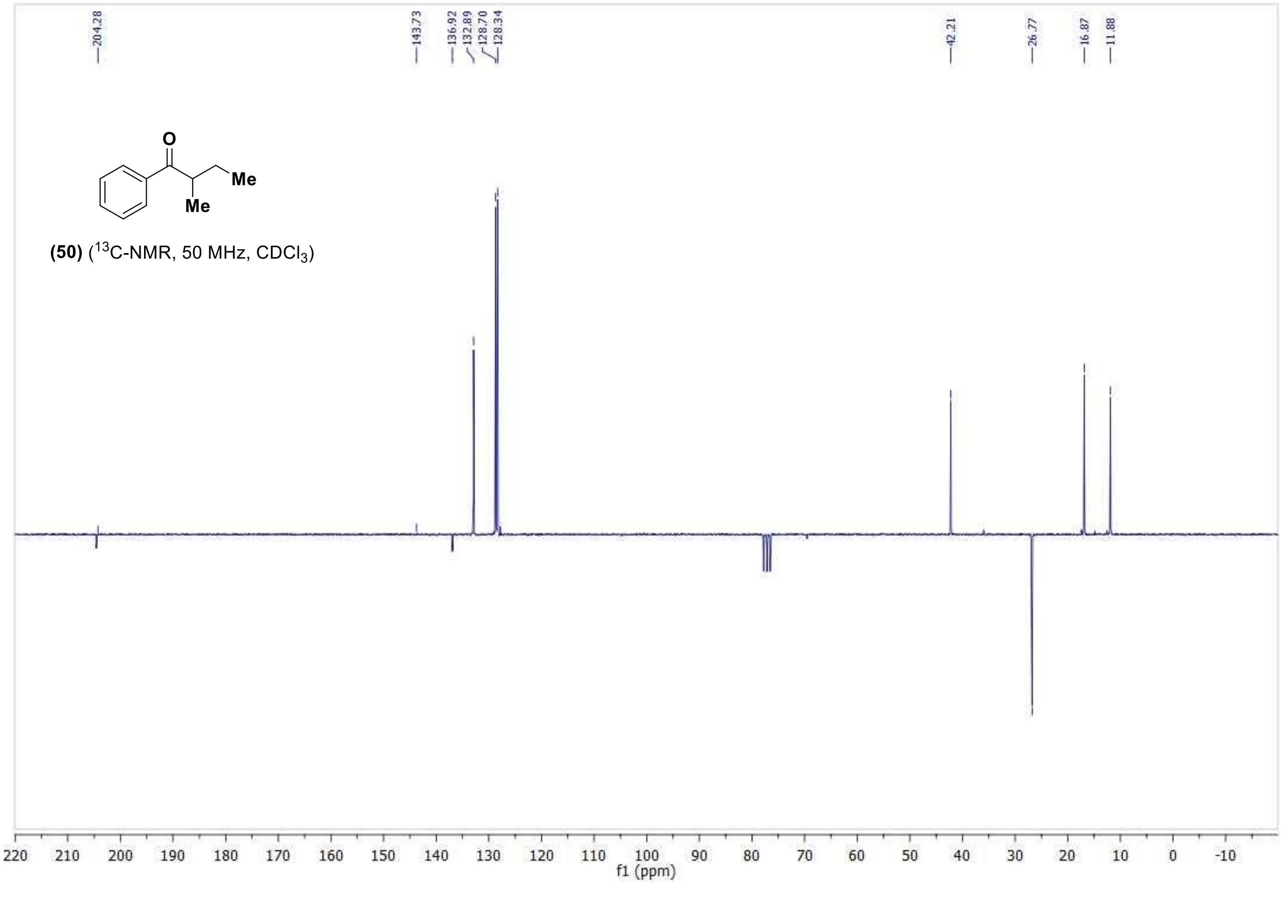

(50) (^{13}C-NMR, 50 MHz, $CDCl_3$)

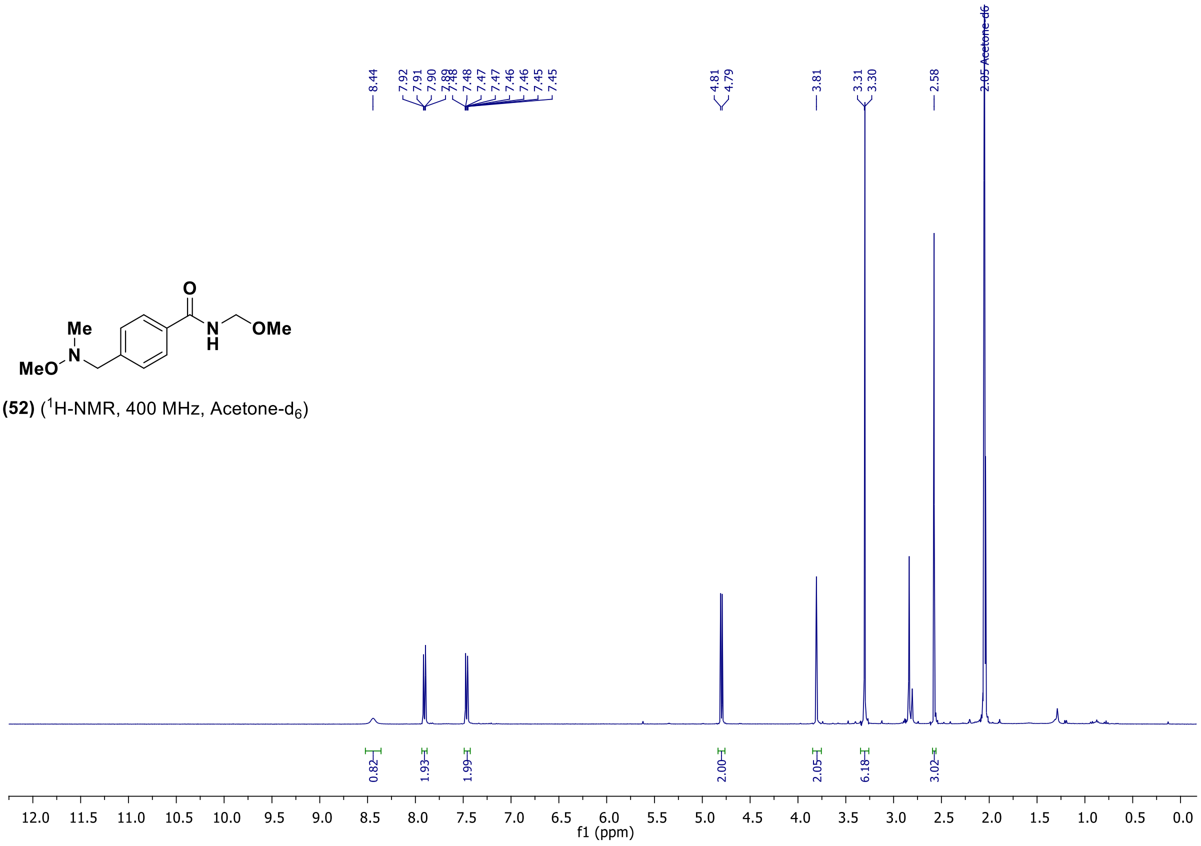

(52) (^{1}H-NMR, 400 MHz, Acetone-d$_6$)

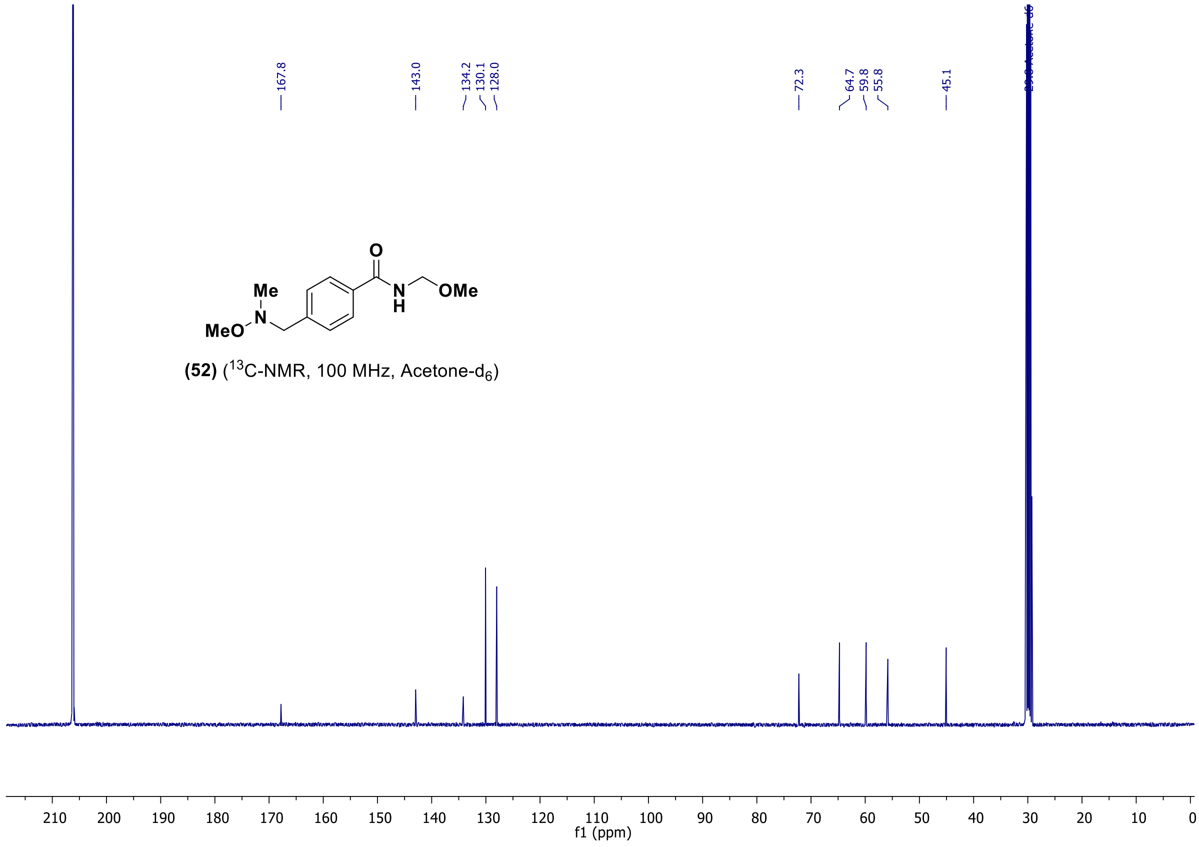

(52) (^{13}C-NMR, 100 MHz, Acetone-d$_6$)

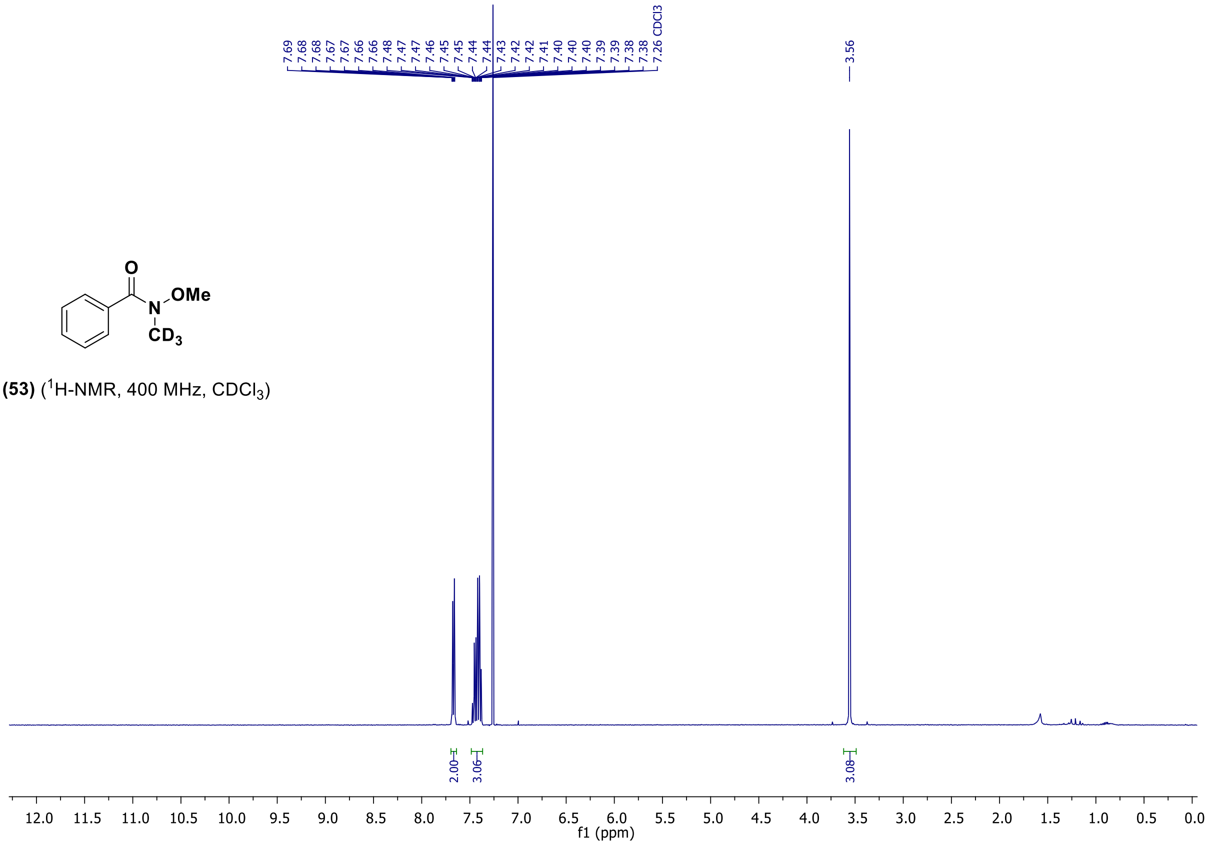

(53) (^{1}H-NMR, 400 MHz, $CDCl_3$)

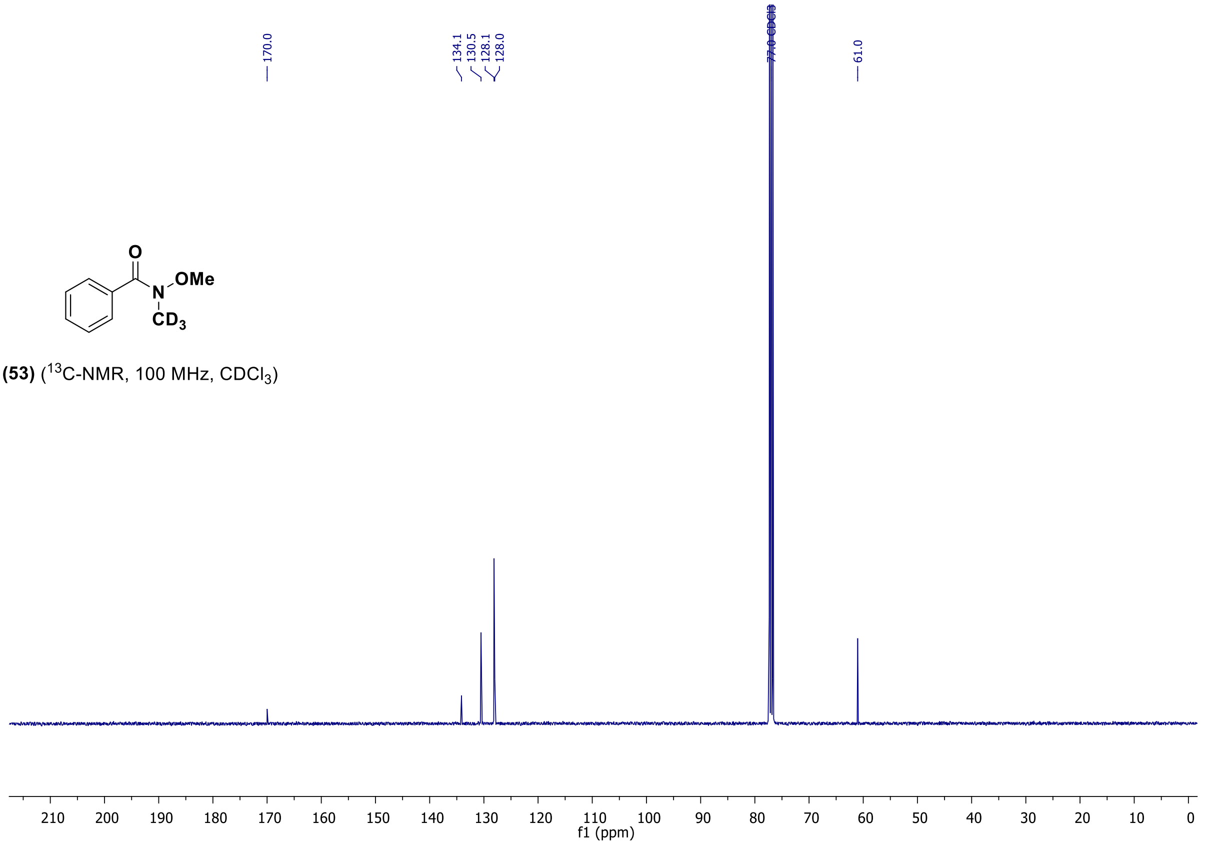

(53) (^{13}C-NMR, 100 MHz, $CDCl_3$)

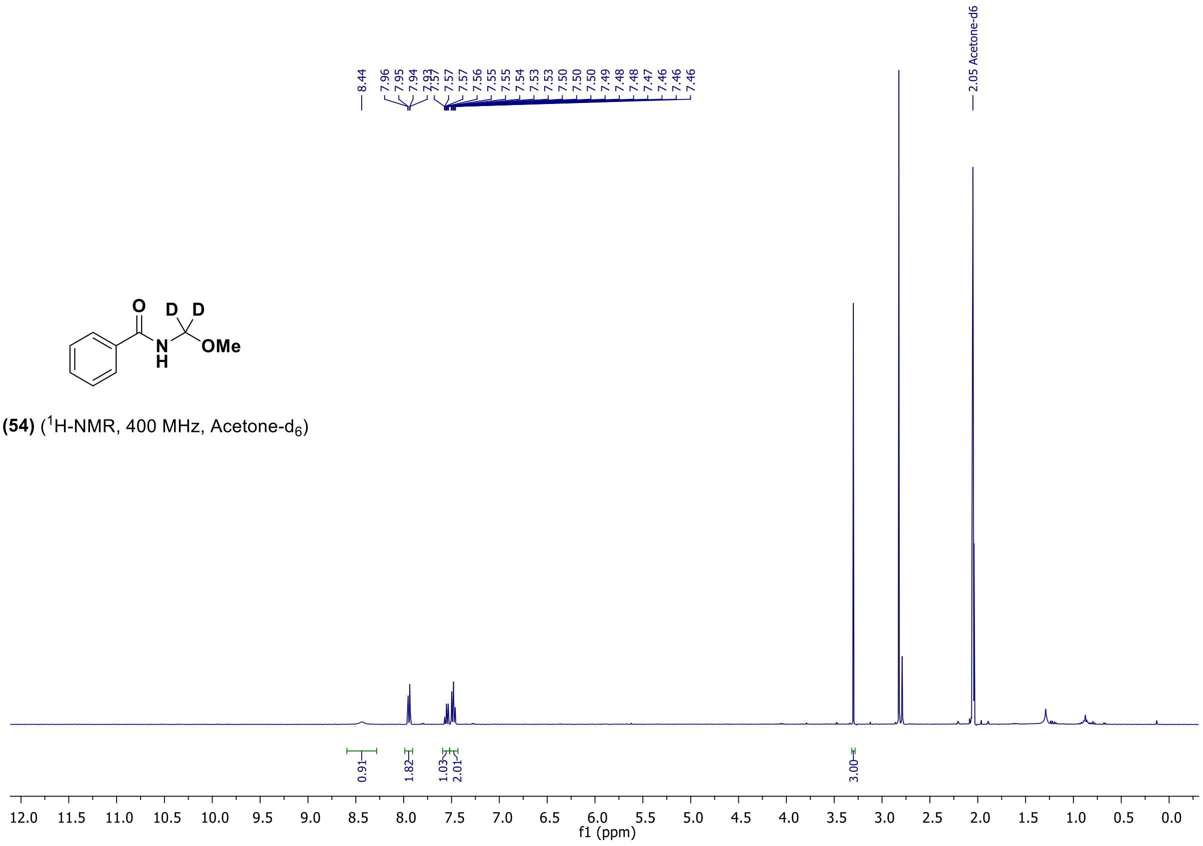

(54) (^{1}H-NMR, 400 MHz, Acetone-d_6)

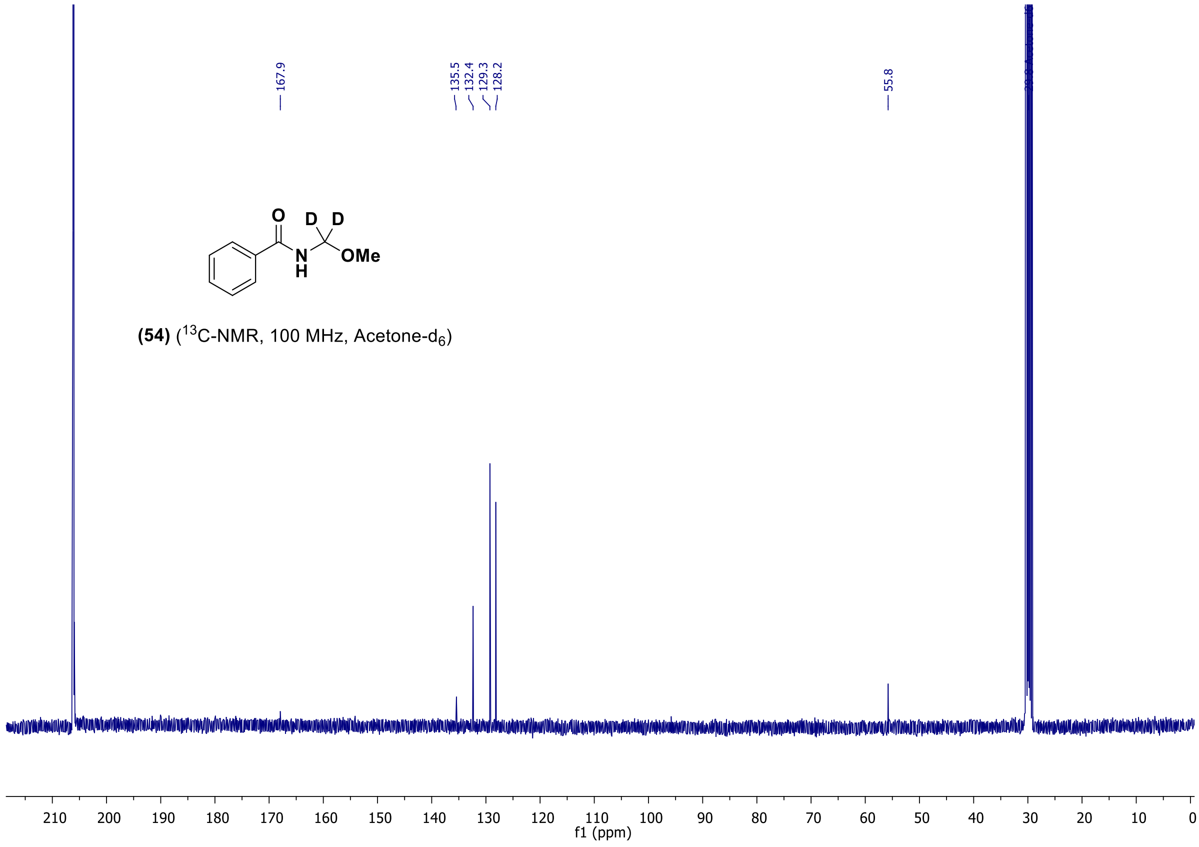

(54) (^{13}C-NMR, 100 MHz, Acetone-d_6)

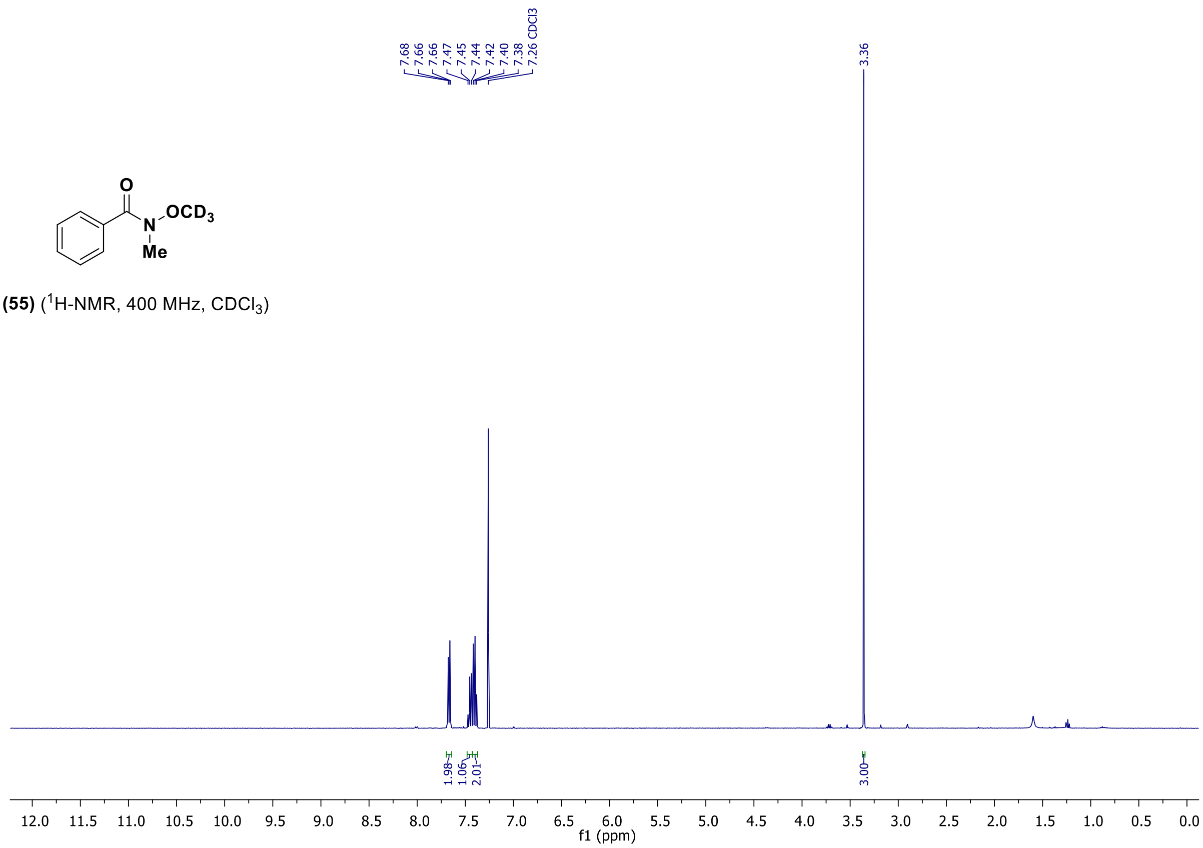

(55) (^{1}H-NMR, 400 MHz, $CDCl_3$)

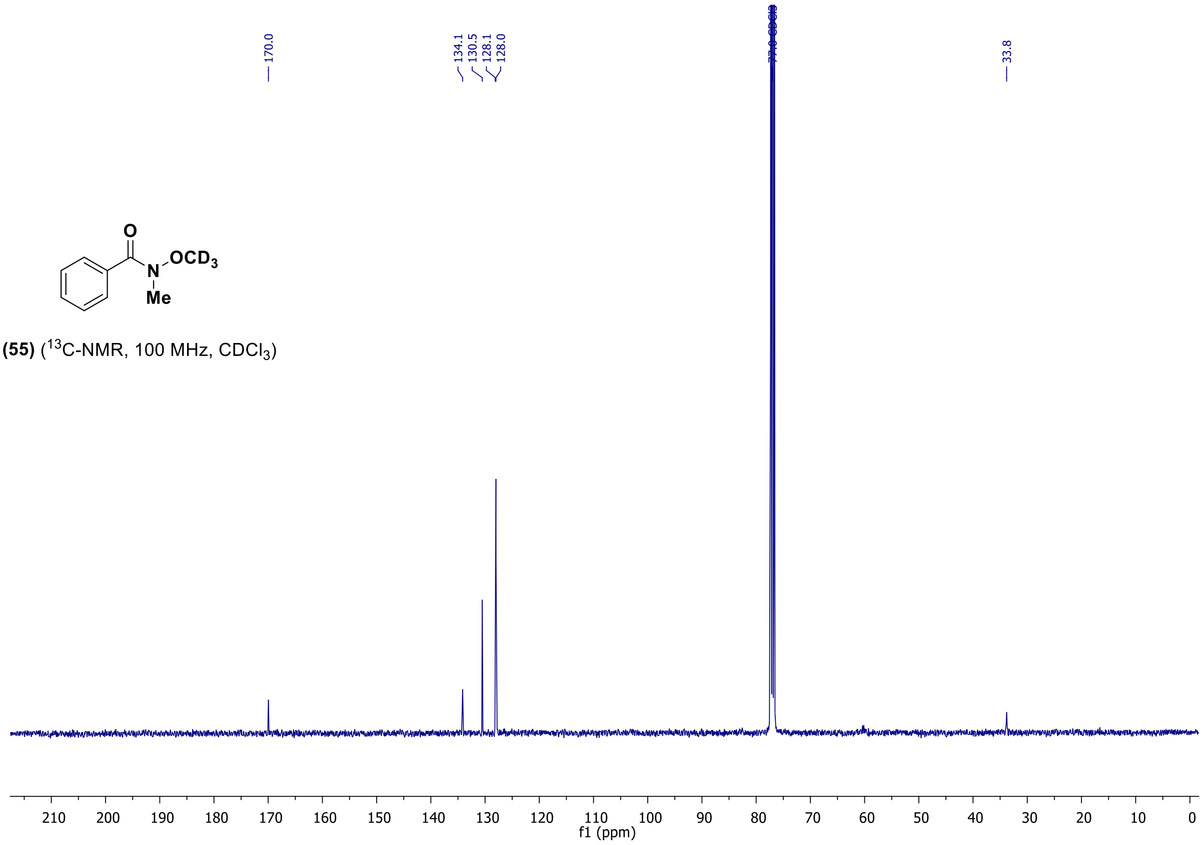

(55) (^{13}C-NMR, 100 MHz, $CDCl_3$)

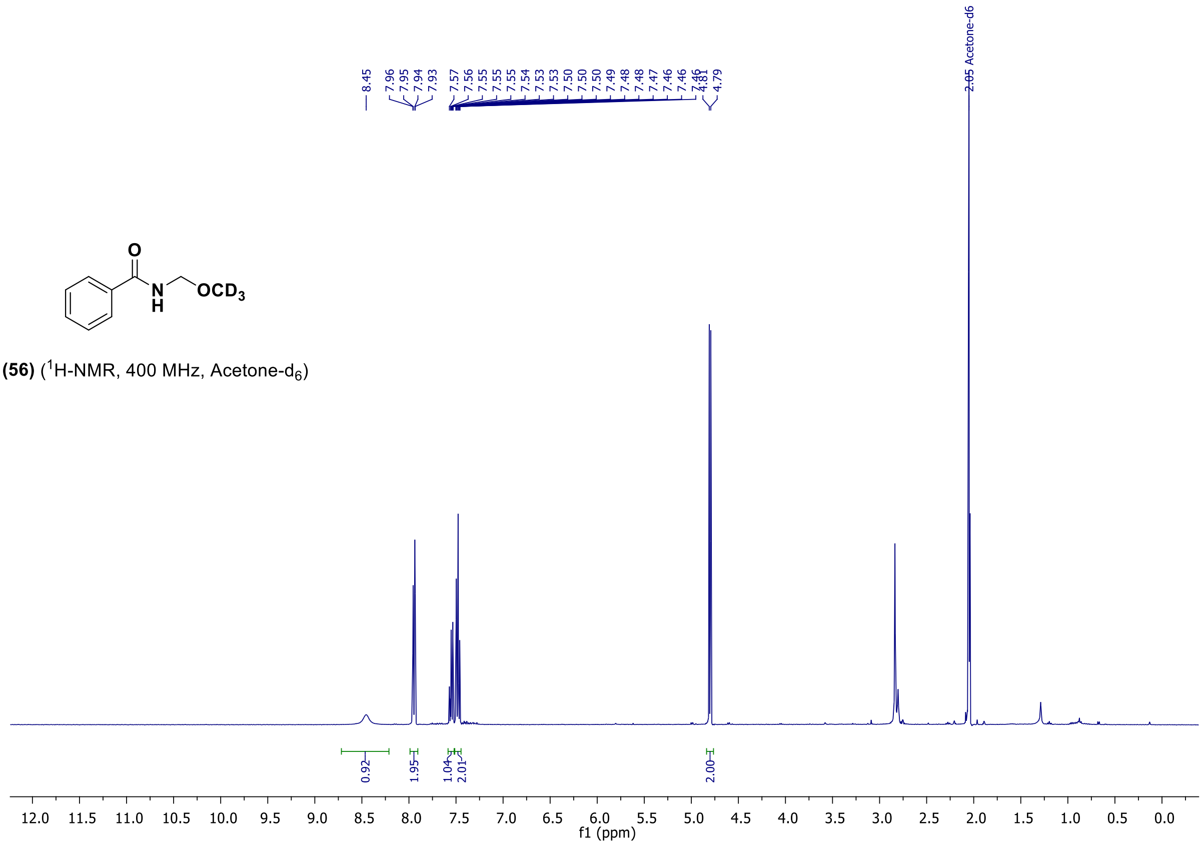

(56) (^{1}H-NMR, 400 MHz, Acetone-d$_6$)

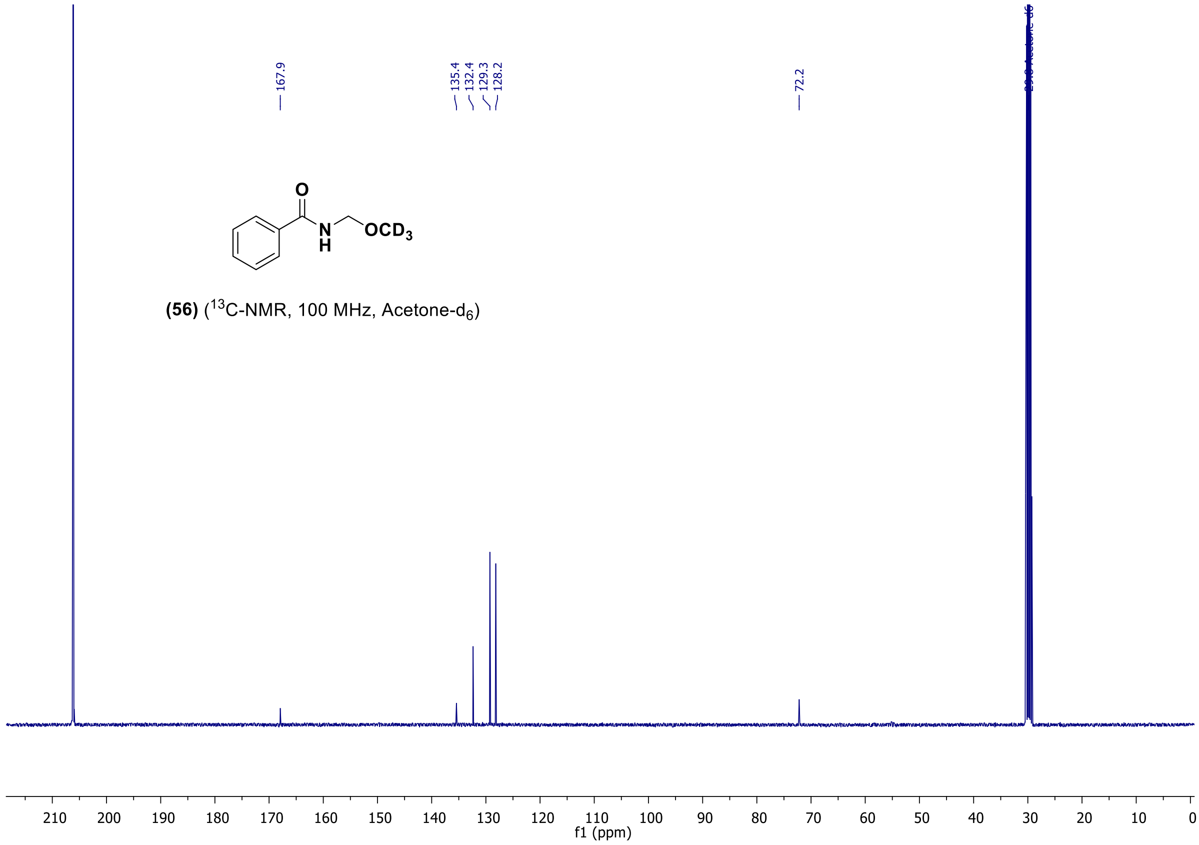

(56) (^{13}C-NMR, 100 MHz, Acetone-d$_6$)

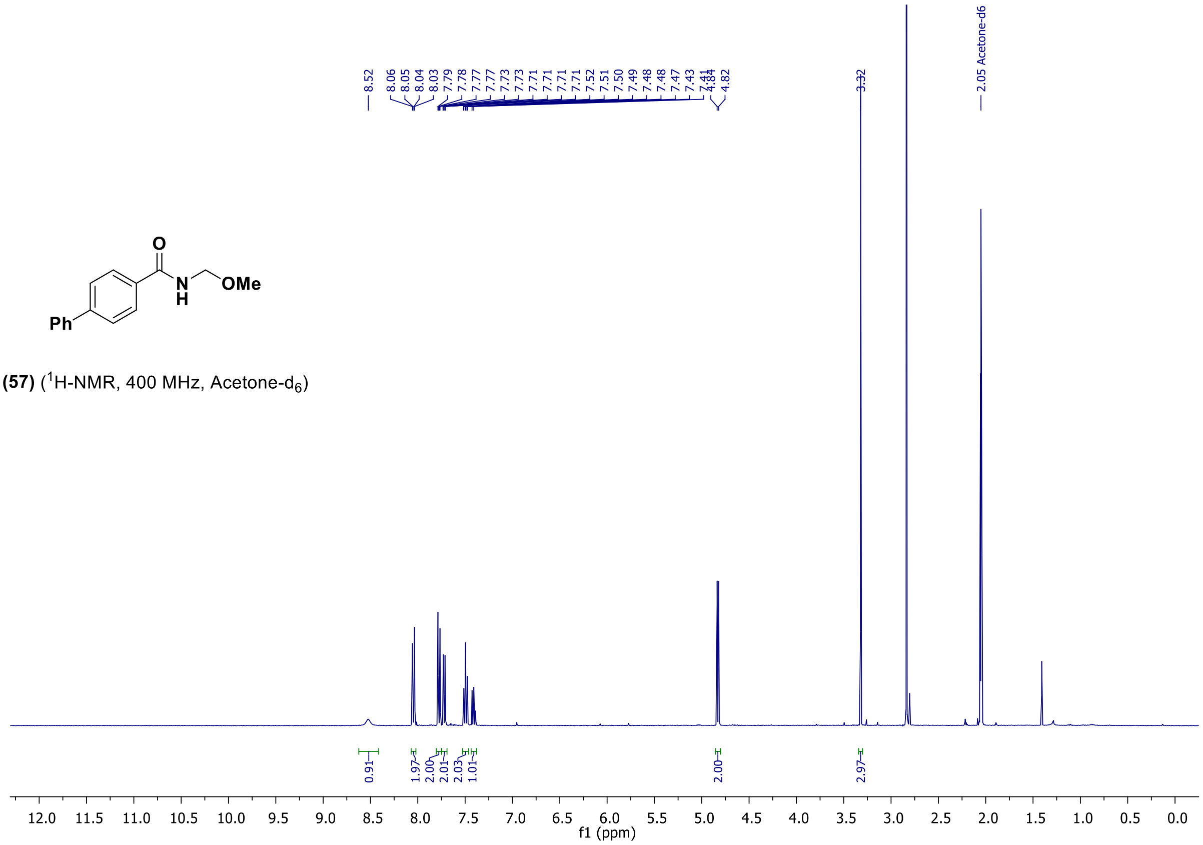

(57) (^{1}H-NMR, 400 MHz, Acetone-d_6)

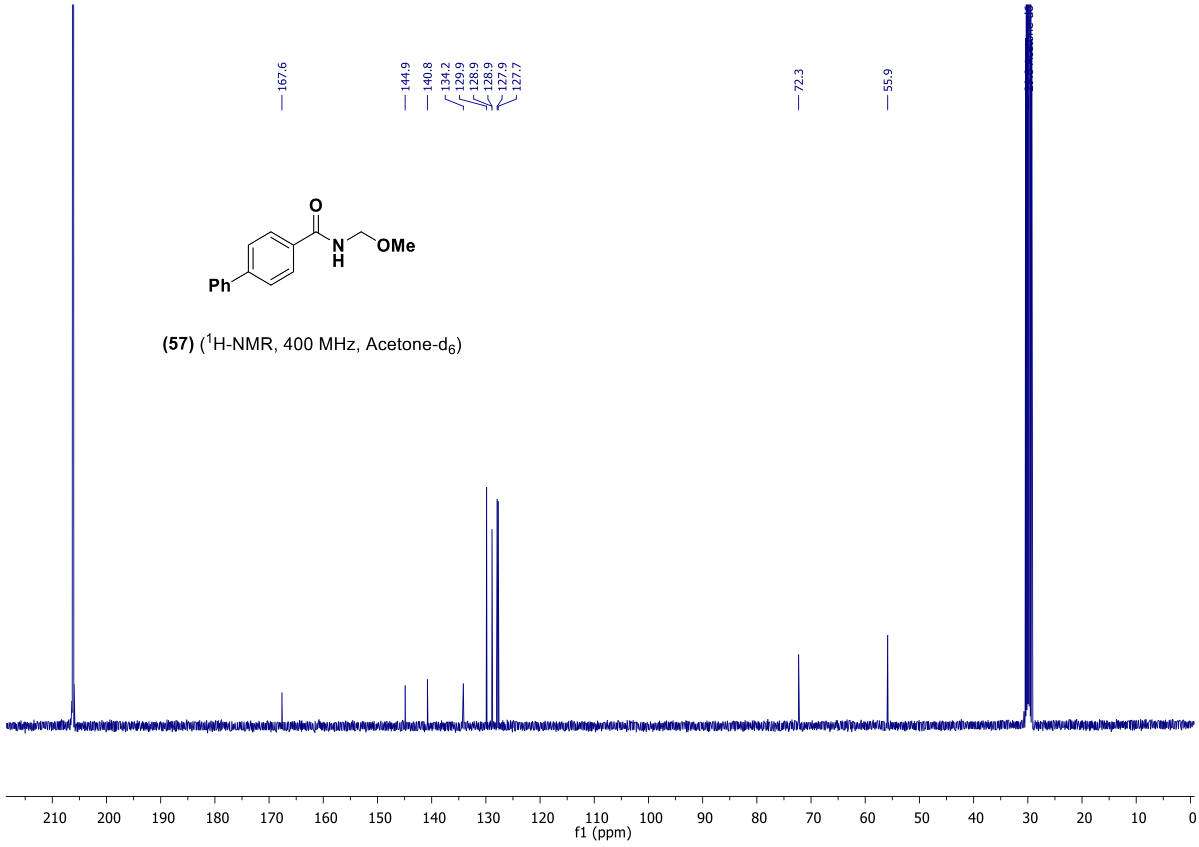

(57) (^{1}H-NMR, 400 MHz, Acetone-d_6)

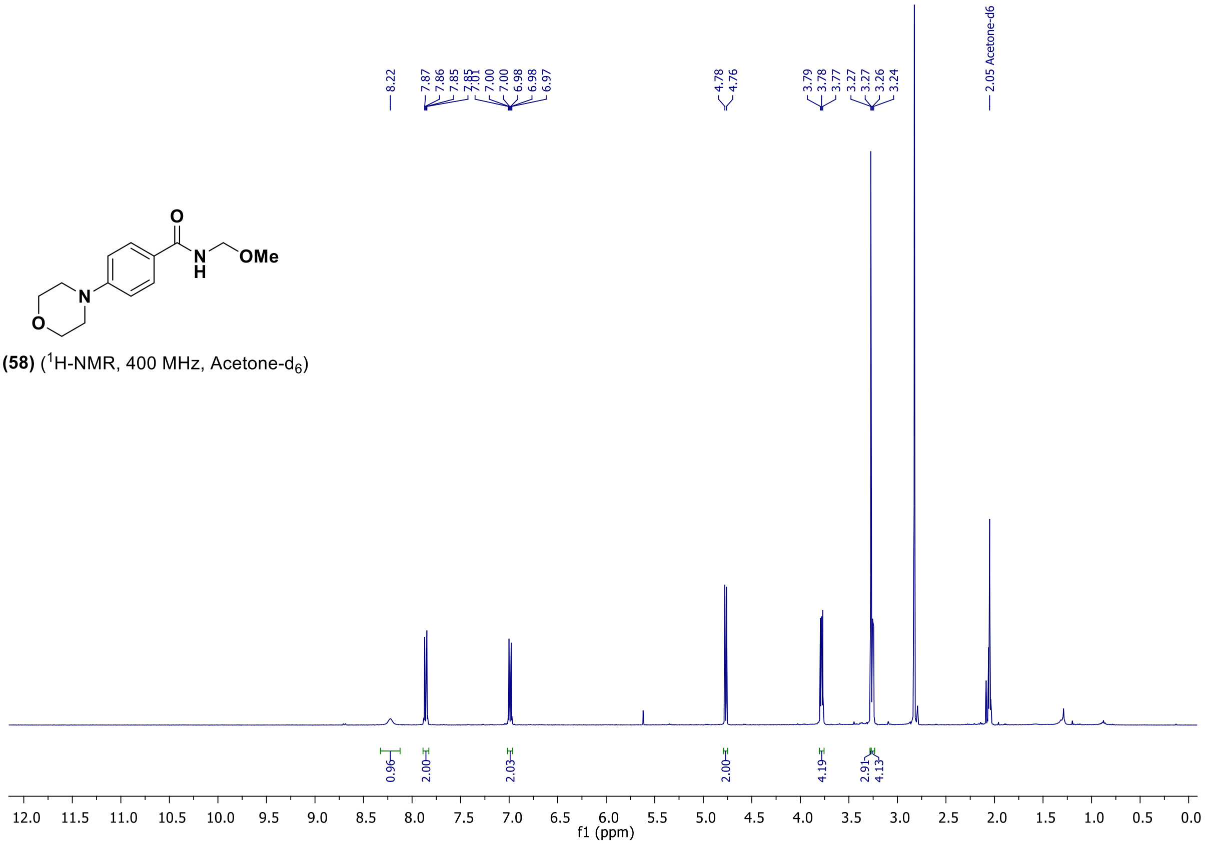

(58) (^{1}H-NMR, 400 MHz, Acetone-d$_6$)

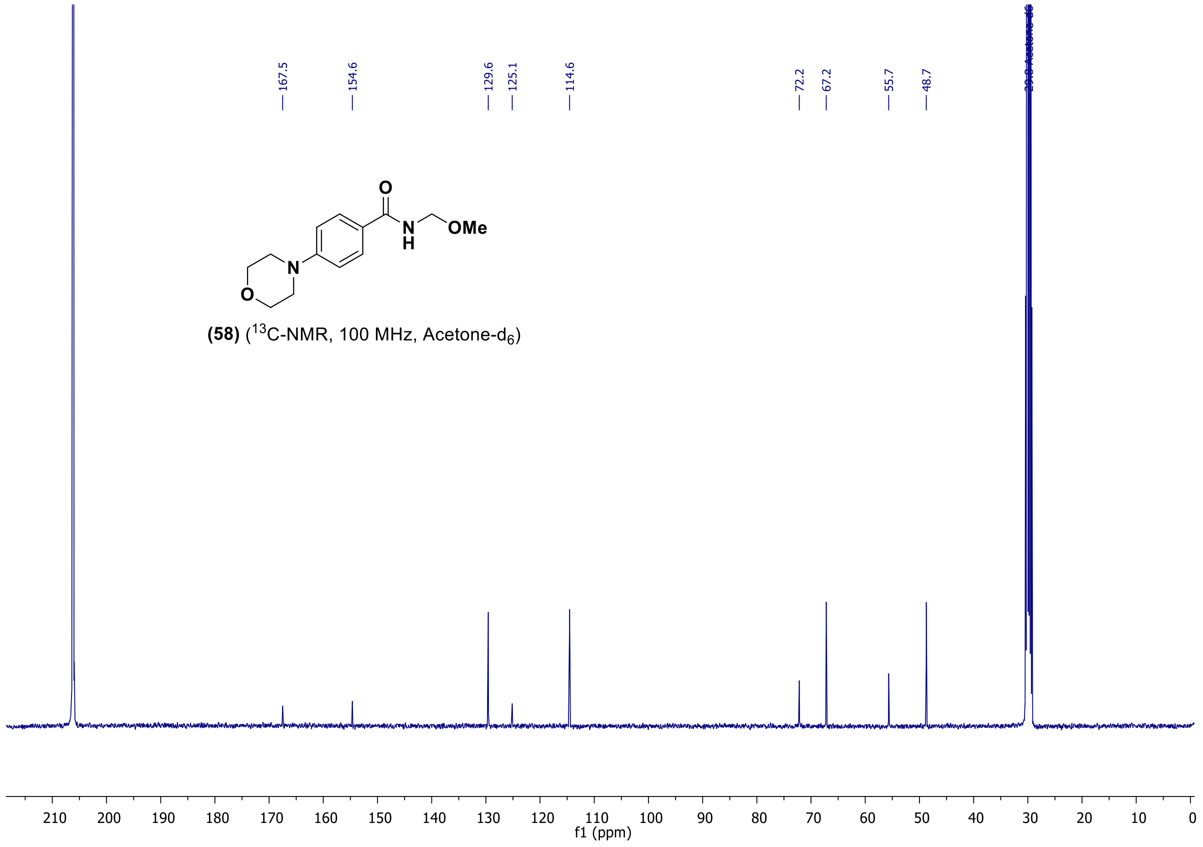

(58) (^{13}C-NMR, 100 MHz, Acetone-d$_6$)

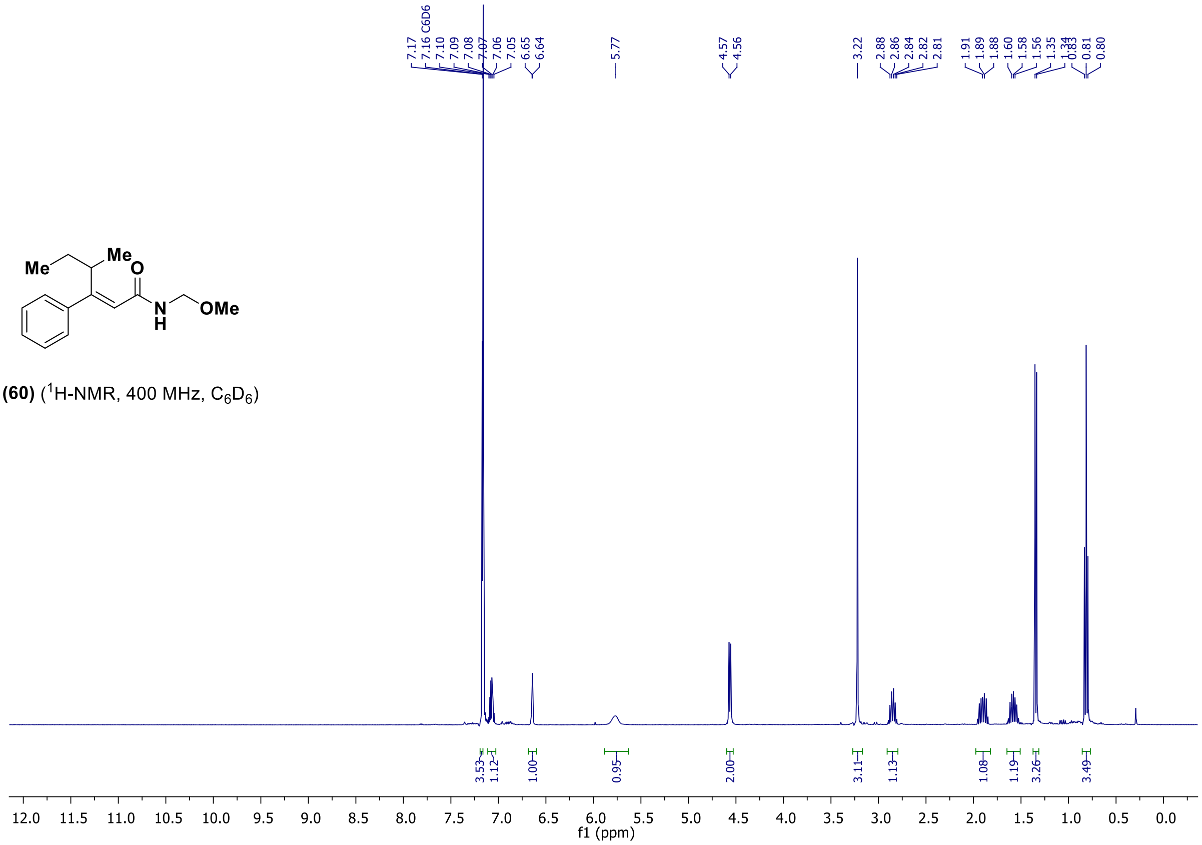

(60) (^{1}H-NMR, 400 MHz, C_6D_6)

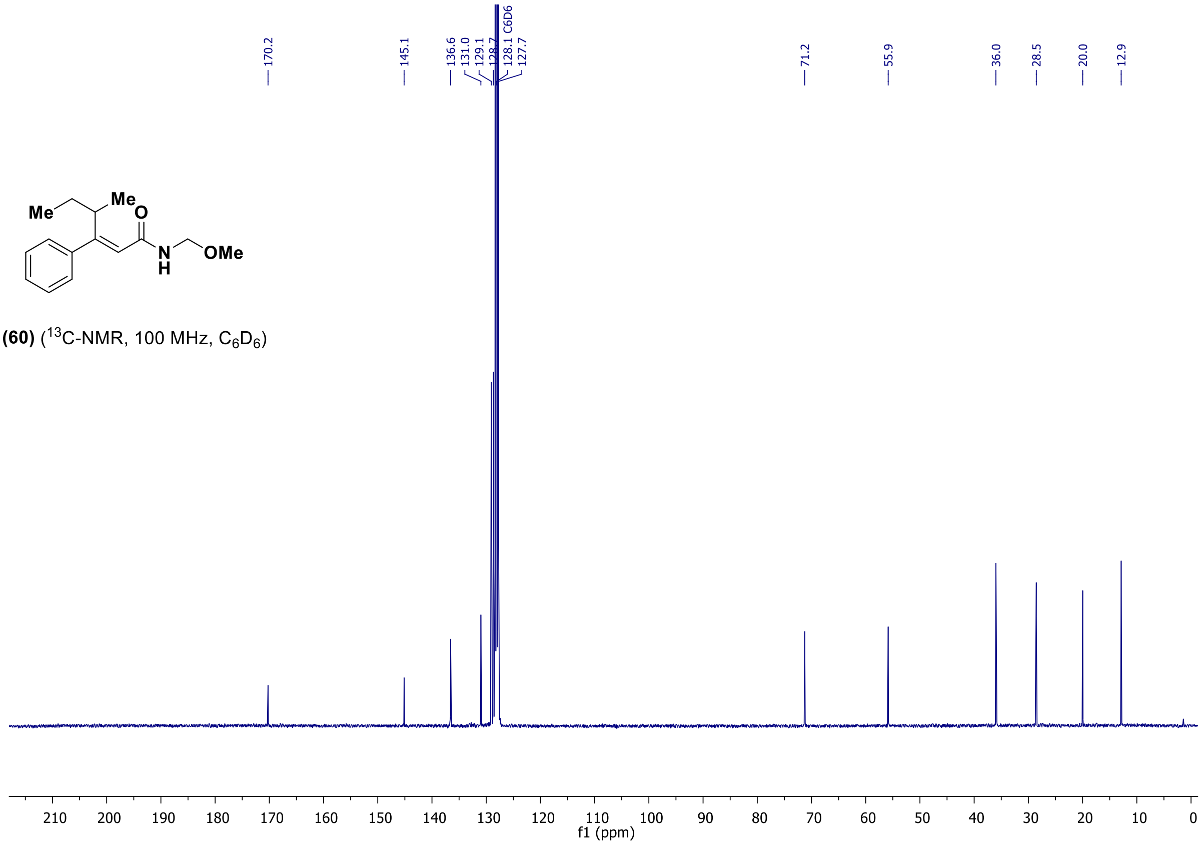

(60) (^{13}C-NMR, 100 MHz, C_6D_6)

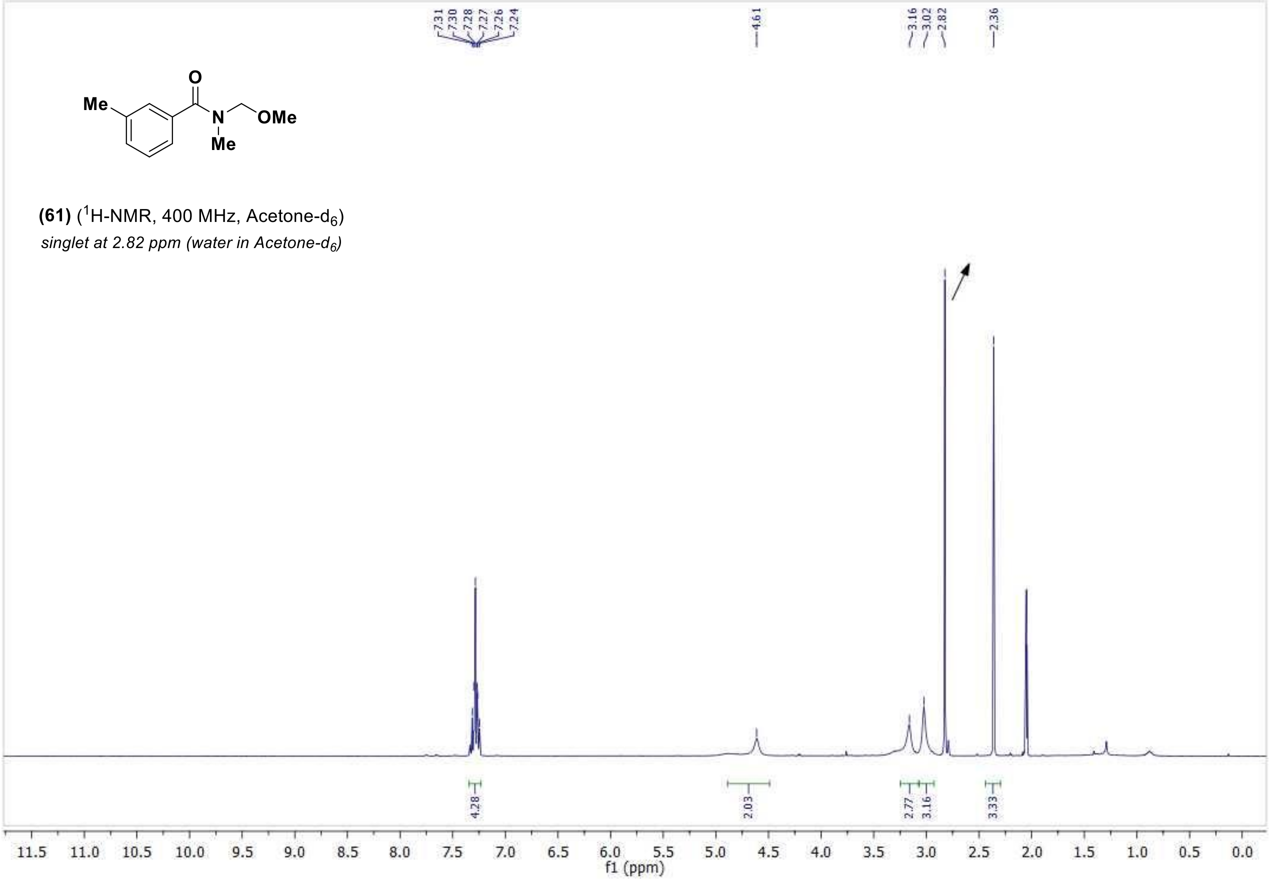
7.31
7.30
7.28
7.27
7.26
7.24
4.61
3.16
3.02
2.82
2.36
Me
O
N
OMe
Me
(61) (^{1}H-NMR, 400 MHz, Acetone-d$_6$)
singlet at 2.82 ppm (water in Acetone-d$_6$)
4.28
2.03
2.77
3.16
3.33
11.5 11.0 10.5 10.0 9.5 9.0 8.5 8.0 7.5 7.0 6.5 6.0 5.5 5.0 4.5 4.0 3.5 3.0 2.5 2.0 1.5 1.0 0.5 0.0
f1 (ppm)

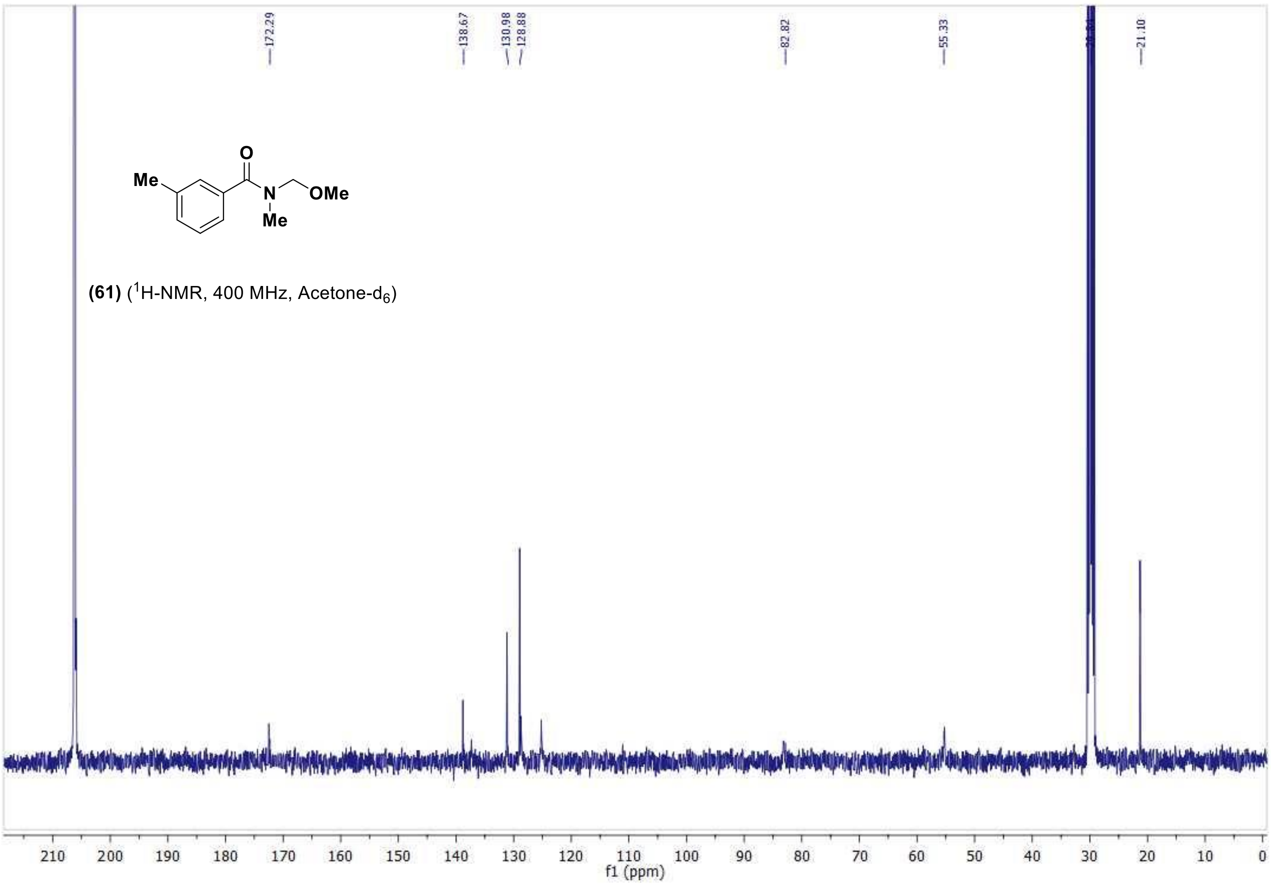
172.29
138.67
130.98
128.88
82.82
55.33
21.10
Me
O
N
OMe
Me
(61) (^{1}H-NMR, 400 MHz, Acetone-d$_6$)
210 200 190 180 170 160 150 140 130 120 110 100 90 80 70 60 50 40 30 20 10 0
f1 (ppm)

Copies of heteronuclei NMR (^{19}F-, ^{77}Se- and ^{119}Sn) spectra

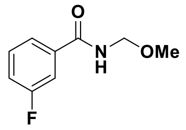

(24) (^{19}F-NMR, 376 MHz, $CDCl_3$)

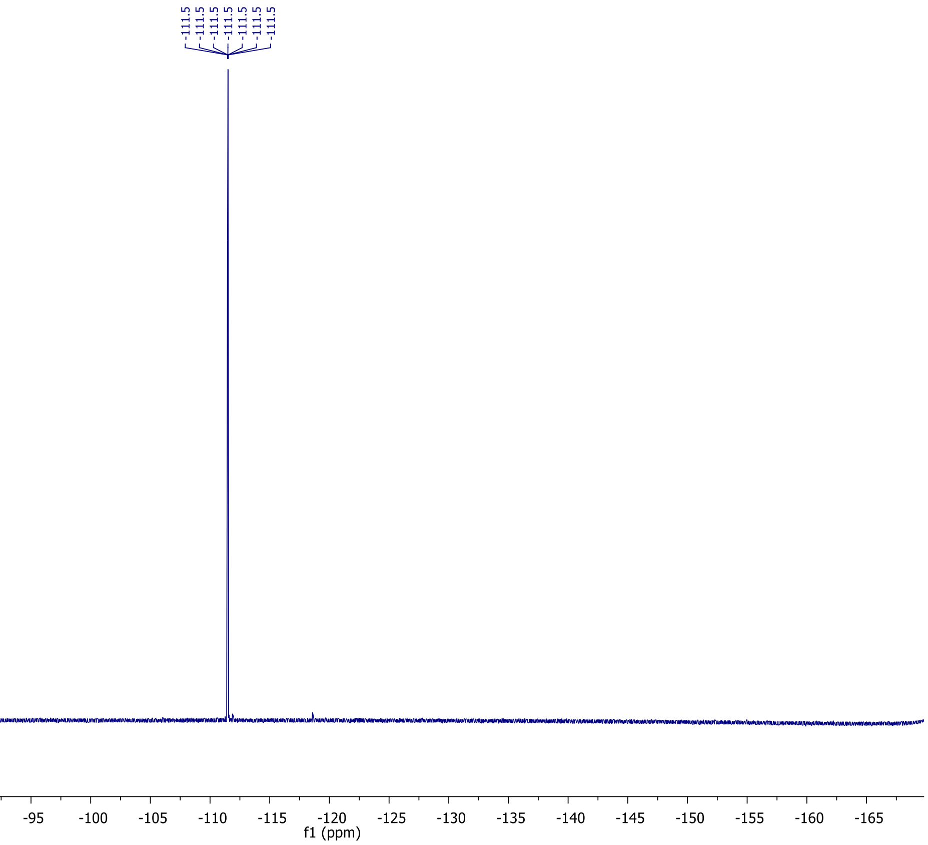

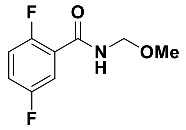

(25) (^{19}F-NMR, 376 MHz, $CDCl_3$)

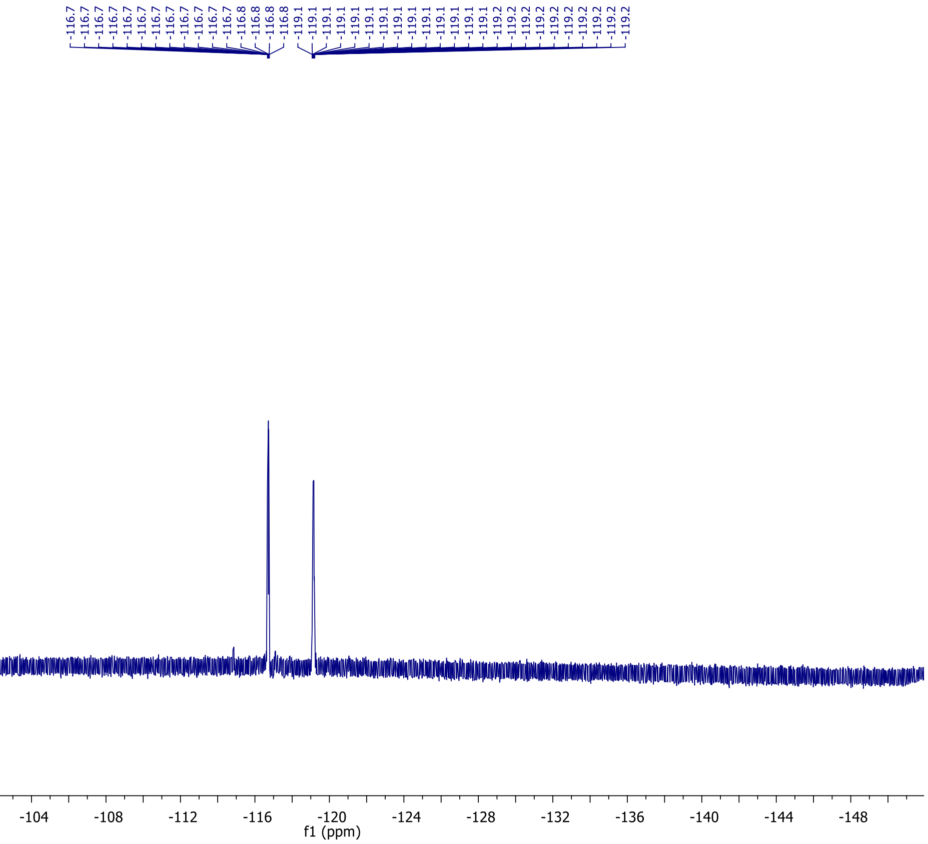

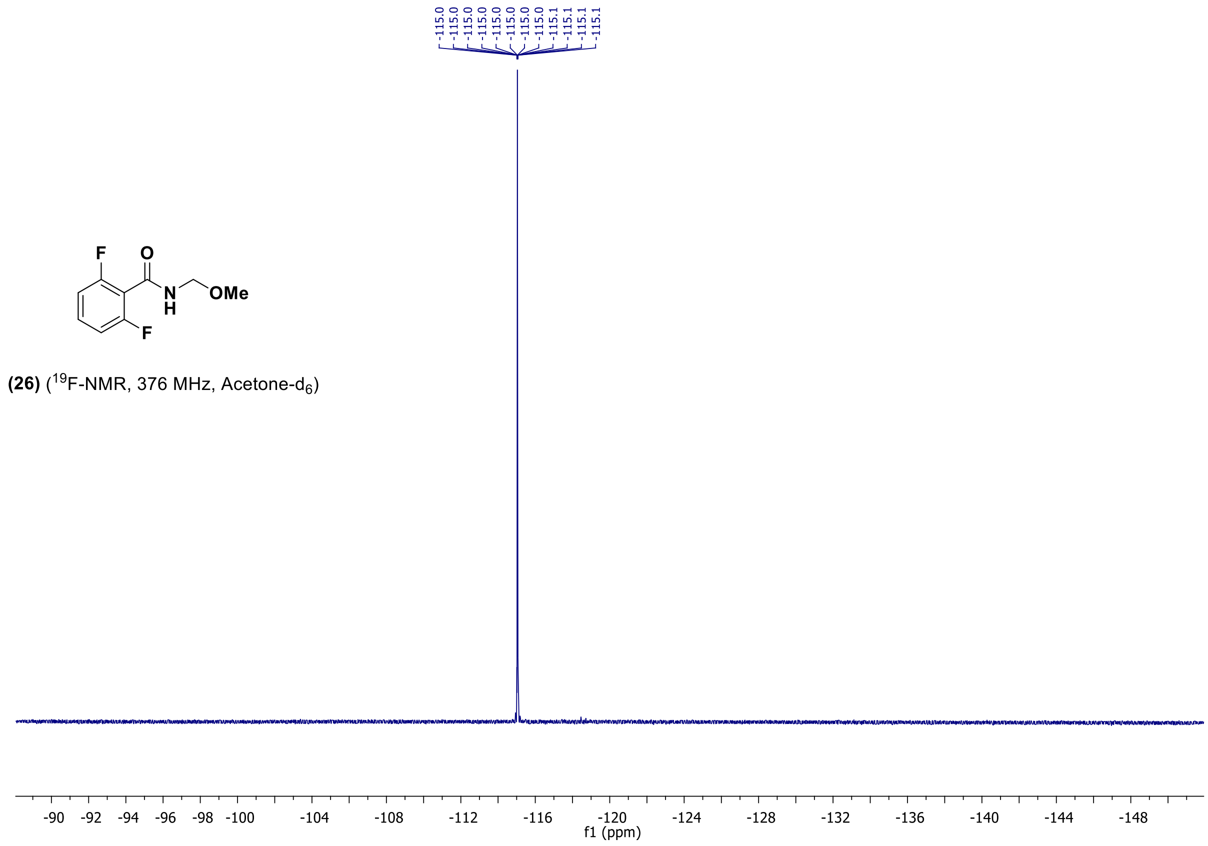

(26) (^{19}F-NMR, 376 MHz, Acetone-d_6)

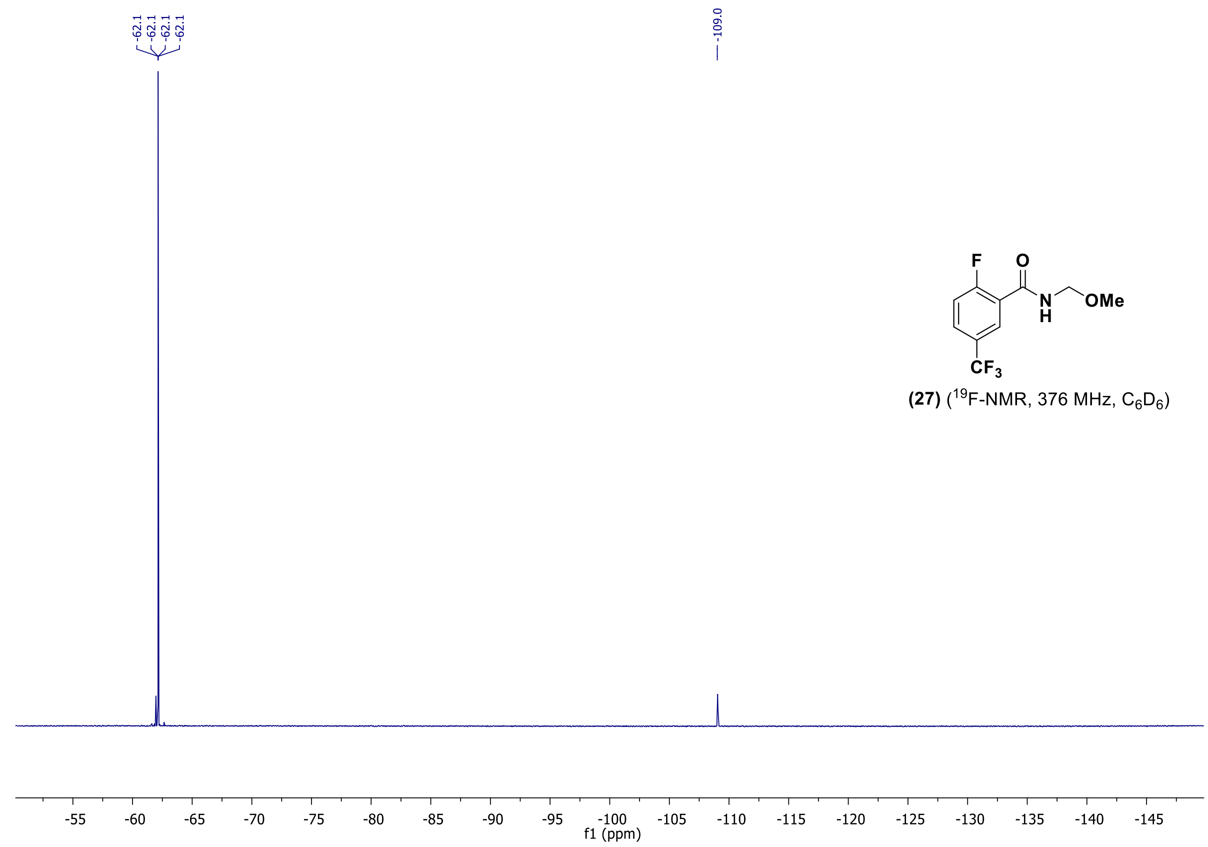

(27) (^{19}F-NMR, 376 MHz, C_6D_6)

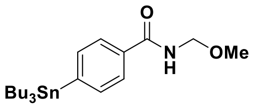

(34) (^{119}Sn-NMR, 149 MHz, C_6D_6)

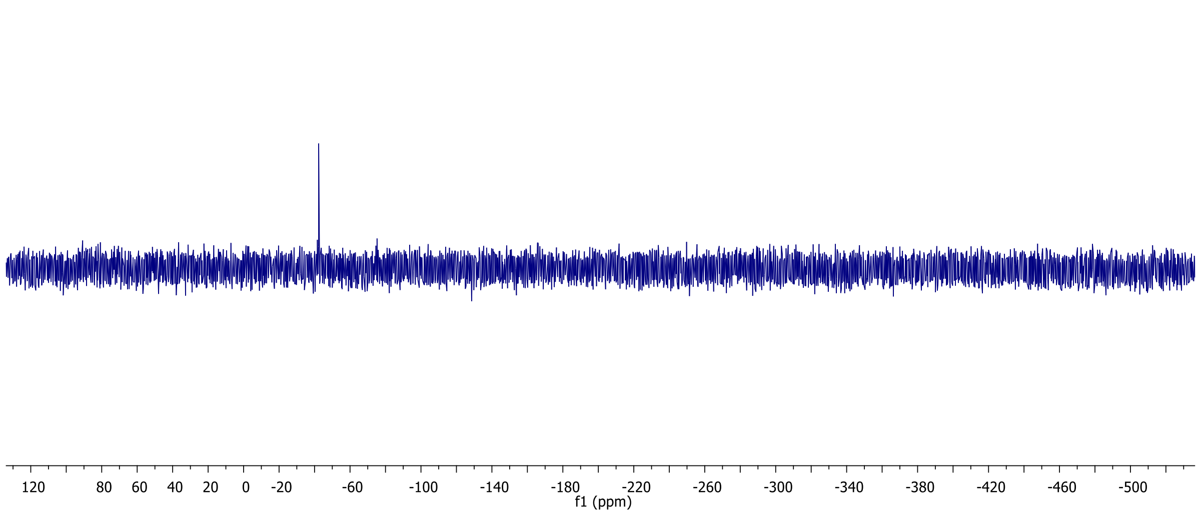

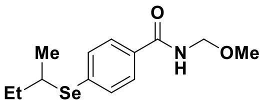

(35) (^{77}Se-NMR, 76 MHz, Acetone-d_6)

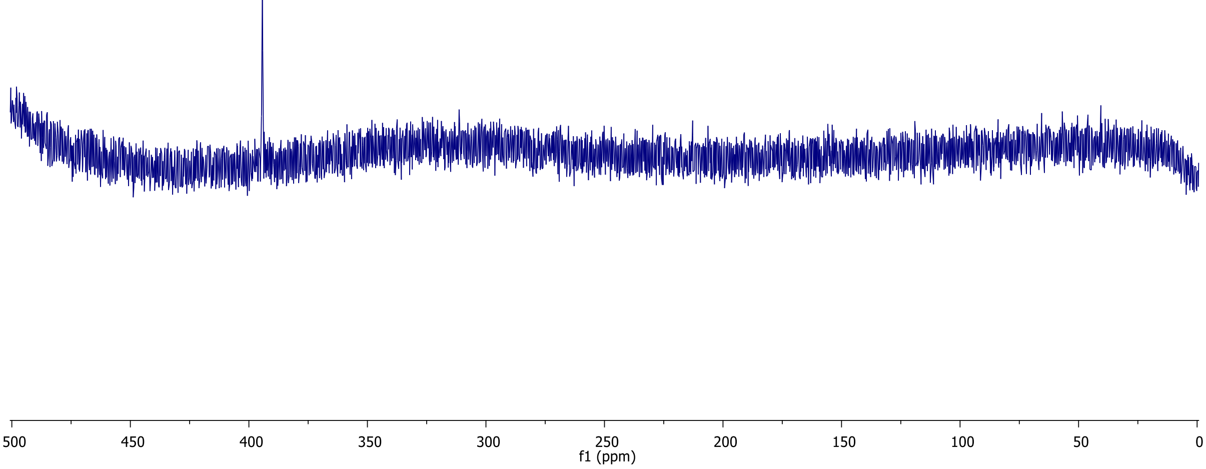

X-ray analysis for compound 17

The X-ray intensity data were measured on STOE STADIVARI diffractometer equipped with multilayer monochromator, Cu K/α Primux 100 micro, micro focus sealed tube and Oxford cooling system. The structure solved by *charge flipping*. Non-hydrogen atoms were refined with *anisotropic displacement parameters*. Hydrogen atoms were inserted at calculated positions and refined with riding model. The following softwares were used: X-Area Integrate Software *package*, *X-Area LANA* for scaling, *OLEX2*[15] for structure solution, refinement, molecular diagrams and graphical user-interface, *Shelxl* for refinement and graphical user-interface *olex2.solve 1.5* for structure solution, *SHELXL-2015* for refinement, *Platon*[i] for symmetry check. Experimental data and CCDC-Codes Experimental data (Available online: http://www.ccdc.cam.ac.uk/conts/retrieving.html) can be found in Table 1. Crystal data, data collection parameters, and structure refinement details given in Table 2. Asymmetric Units visualized in Figure 1, intermolecular H-Bond in Figure 4. Data quality visualizations in Figure 2 and 3.

Table 1

Compound	Machine	Source	Temp.	Detector Distance	Time/ Frame	#Frames	Frame width	CCDC
			[K]	[mm]	[s]		[°]	
**17 (MAL261)**	Stoe Stadivari	Cu	100	40	2	2174	1	2191936

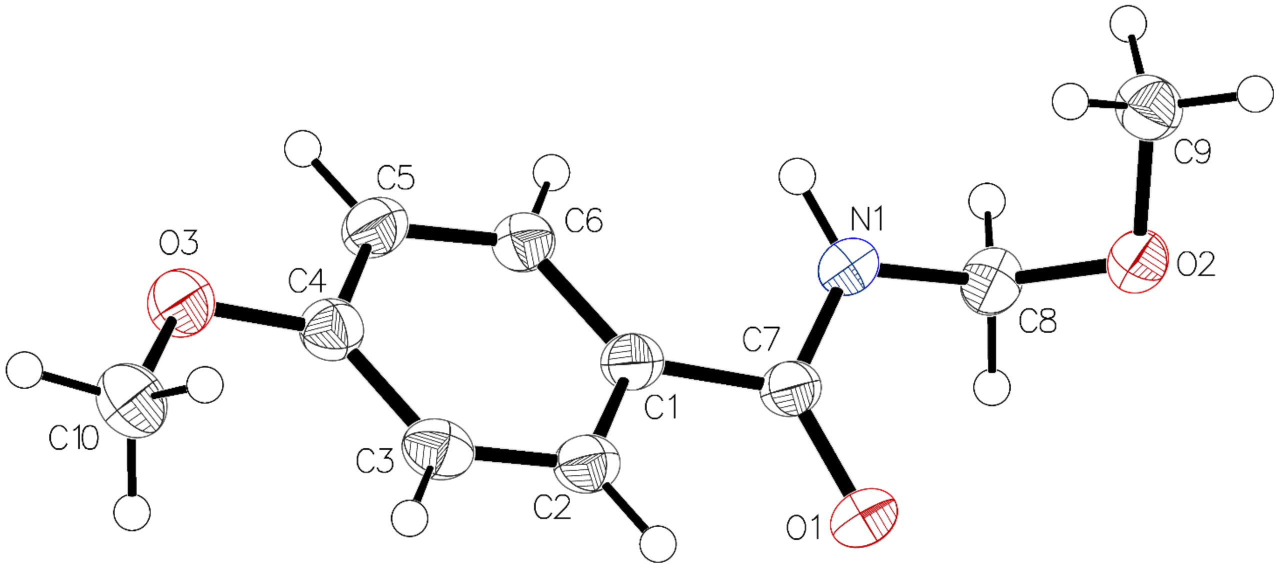

Figure 1 Asymmetric Unit (compound **17**) drawn with 50% displacement ellipsoid. The bond precision for C-C single bonds is 0.0020Å.

i_over_sigma_vs_resolution.htm

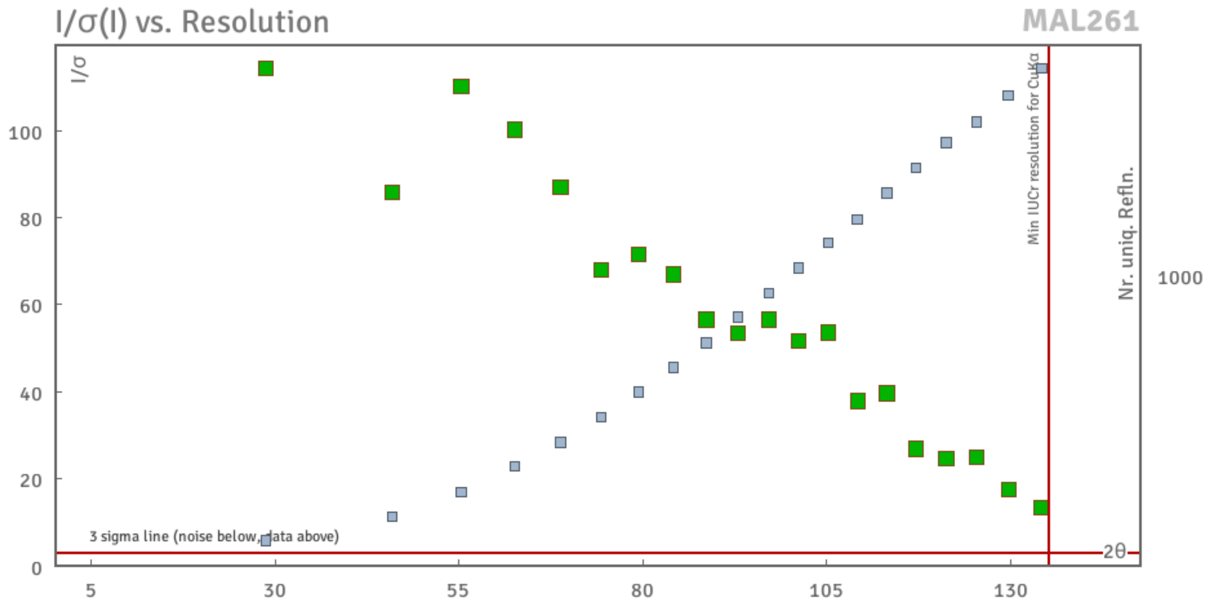

Figure 2 Data Quality – I/Sigma (I) level view

cc_half_vs_resolution.htm

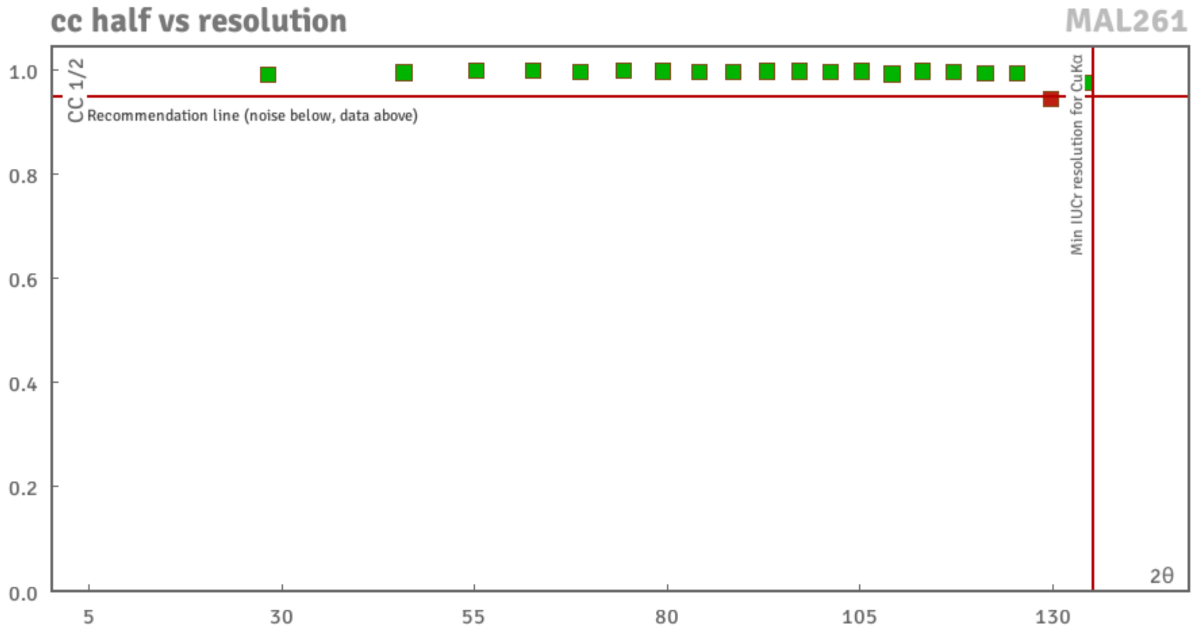

Figure 3 Data Quality – CC 1/2 level view

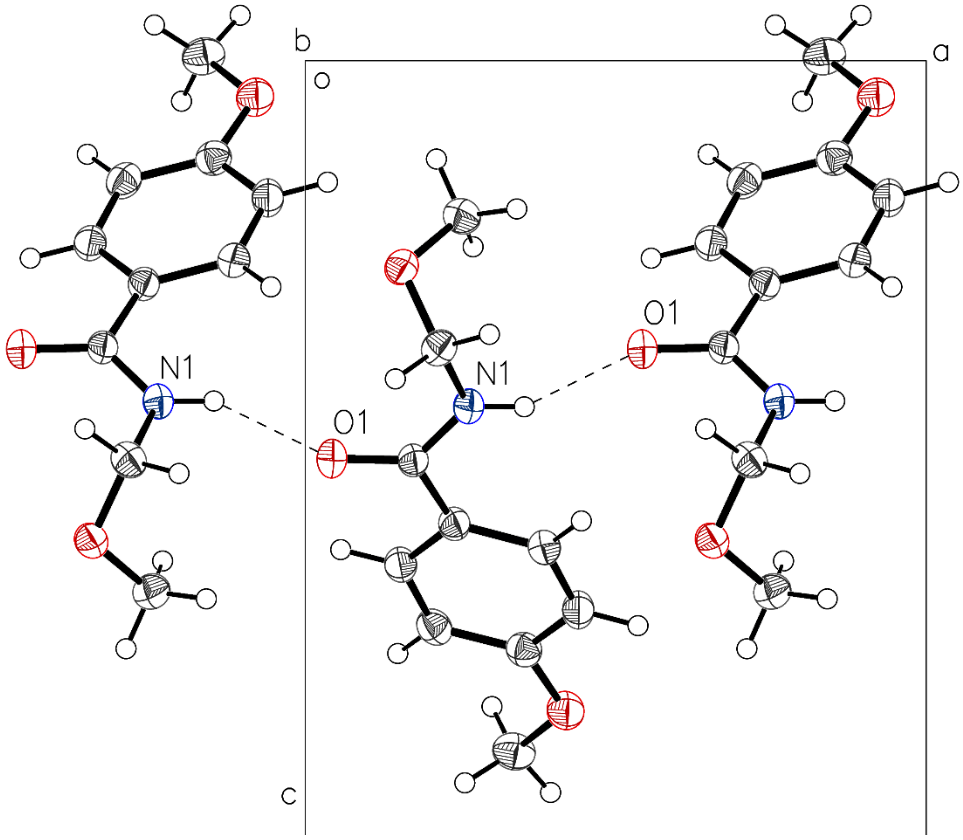

Figure 4 Intermolecular H-Bonds of moderate character in direction of axis “a” influence the packing.

Table 2 Sample and crystal data, data collection and structure refinement. Detailed information can found in the Cif Code of CCDC: 2191936

Identification code	MAL261
Empirical formula	$C_{10}H_{13}NO_3$
Formula weight	195.21
Temperature/K	100
Crystal system	orthorhombic
Space group	Pbca
a/Å	9.3359(13)
b/Å	10.169(3)
c/Å	20.658(3)
α/°	90
β/°	90

γ/°	90
Volume/Å^3	1961.2(7)
Z	8
ρ_{calc}g/cm^3	1.322
μ/mm^{-1}	0.813
F(000)	832.0
Crystal size/mm^3	0.3 × 0.217 × 0.1
Radiation	Cu Kα (λ = 1.54186)
2Θ range for data collection/°	8.56 to 136.55
Index ranges	-11 ≤ h ≤ 3, -12 ≤ k ≤ 11, -23 ≤ l ≤ 24
Reflections collected	11452
Independent reflections	3563 [R_{int} = 0.0375, R_{sigma} = 0.0212]
Data/restraints/parameters	3563/0/130
Goodness-of-fit on F^2	1.051
Final R indexes [I>=2σ (I)]	R_1 = 0.0549, wR_2 = 0.1523
Final R indexes [all data]	R_1 = 0.0560, wR_2 = 0.1541
Largest diff. peak/hole / e Å^{-3}	0.28/-0.24

References

[1] H. E. Gottlieb, V. Kotlyar, A. Nudelman, *J. Org. Chem.* **1997**, *62*, 7512-7515.
[2] S. Touqeer, R. Senatore, M. Malik, E. Urban, V. Pace, *Adv. Synth. Catal.* **2020**, *362*, 5056-5061.
[3] H. A. Duong, R. E. Gilligan, M. L. Cooke, R. J. Phipps, M. J. Gaunt, *Angew. Chem. Int. Ed.* **2011**, *50*, 463-466.
[4] X. Wu, B. Wang, Y. Zhou, H. Liu, *Org. Lett.* **2017**, *19*, 1294-1297.
[5] A. Nasier, X. Chang, C. Guo, *J. Org. Chem.* **2021**, *86*, 16068-16076.
[6] G. X. Ortiz, Jr., B. N. Hemric, Q. Wang, *Org. Lett.* **2017**, *19*, 1314-1317.
[7] N. Zhang, Q. Yu, R. Chen, J. Huang, Y. Xia, K. Zhao, *Chem. Commun.* **2013**, *49*, 9464-9466.
[8] N. Guimond, S. I. Gorelsky, K. Fagnou, *J. Am. Chem. Soc.* **2011**, *133*, 6449-6457.
[9] T. Sheradsky, E. Nov, *J. Chem. Soc., Perkin Trans. 1* **1980**, 2781-2786.
[10] M. Allegretti, R. Bertini, M. C. Cesta, C. Bizzarri, R. Di Bitondo, V. Di Cioccio, E. Galliera, V. Berdini, A. Topai, G. Zampella, V. Russo, N. Di Bello, G. Nano, L. Nicolini, M. Locati, P. Fantucci, S. Florio, F. Colotta, *J. Med. Chem.* **2005**, *48*, 4312-4331.
[11] F. Yang, L. Ackermann, *Org. Lett.* **2013**, *15*, 718-720.
[12] F. Alonso, E. Lorenzo, M. Yus, *J. Org. Chem.* **1996**, *61*, 6058-6059.
[13] Y. Fukui, P. Liu, Q. Liu, Z.-T. He, N.-Y. Wu, P. Tian, G.-Q. Lin, *J. Am. Chem. Soc.* **2014**, *136*, 15607-15614.
[14] S. P. H. Mee, V. Lee, J. E. Baldwin, *Angew. Chem. Int. Ed.* **2004**, *43*, 1132-1136.
[15] O. V. Dolomanov, L. J. Bourhis, R. J. Gildea, J. A. K. Howard, H. Puschmann, *J. Appl. Crystallogr.* **2009**, *42*, 339-341.
